# Photoredox-catalyzed aminofluorosulfonylation of unactivated olefins†

**DOI:** 10.1039/d1sc02503a

**Published:** 2021-06-07

**Authors:** Tao Zhong, Ji-Tao Yi, Zhi-Da Chen, Quan-Can Zhuang, Yong-Zhao Li, Gui Lu, Jiang Weng

**Affiliations:** Guangdong Provincial Key Laboratory of Chiral Molecule and Drug Discovery, School of Pharmaceutical Sciences, Sun Yat-sen University Guangzhou 510006 P. R. China wengj2@mail.sysu.edu.cn

## Abstract

The development of efficient approaches to access sulfonyl fluorides is of great significance because of the widespread applications of these structural motifs in many areas, among which the emerging sulfur(vi) fluoride exchange (SuFEx) click chemistry is the most prominent. Here, we report the first three-component aminofluorosulfonylation of unactivated olefins by merging photoredox-catalyzed proton-coupled electron transfer (PCET) activation with radical relay processes. Various aliphatic sulfonyl fluorides featuring a privileged 5-membered heterocyclic core have been efficiently afforded under mild conditions with good functional group tolerance. The synthetic potential of the sulfonyl fluoride products has been examined by diverse transformations including SuFEx reactions and transition metal-catalyzed cross-coupling reactions. Mechanistic studies demonstrate that amidyl radicals, alkyl radicals and sulfonyl radicals are involved in this difunctionalization transformation.

## Abstract 2

A three-component aminofluorosulfonylation of unactivated alkenes has been developed by merging photocatalytic PCET with radical relay processes, affording various aliphatic sulfonyl fluorides featuring medicinally privileged heterocyclic scaffolds.

## Graphical abstract

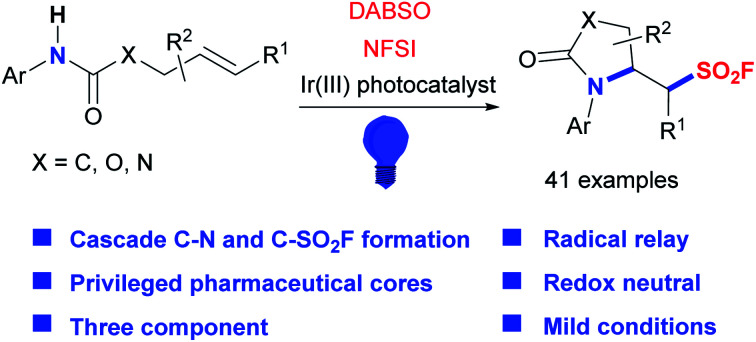

## Introduction

**Fig. 1:**
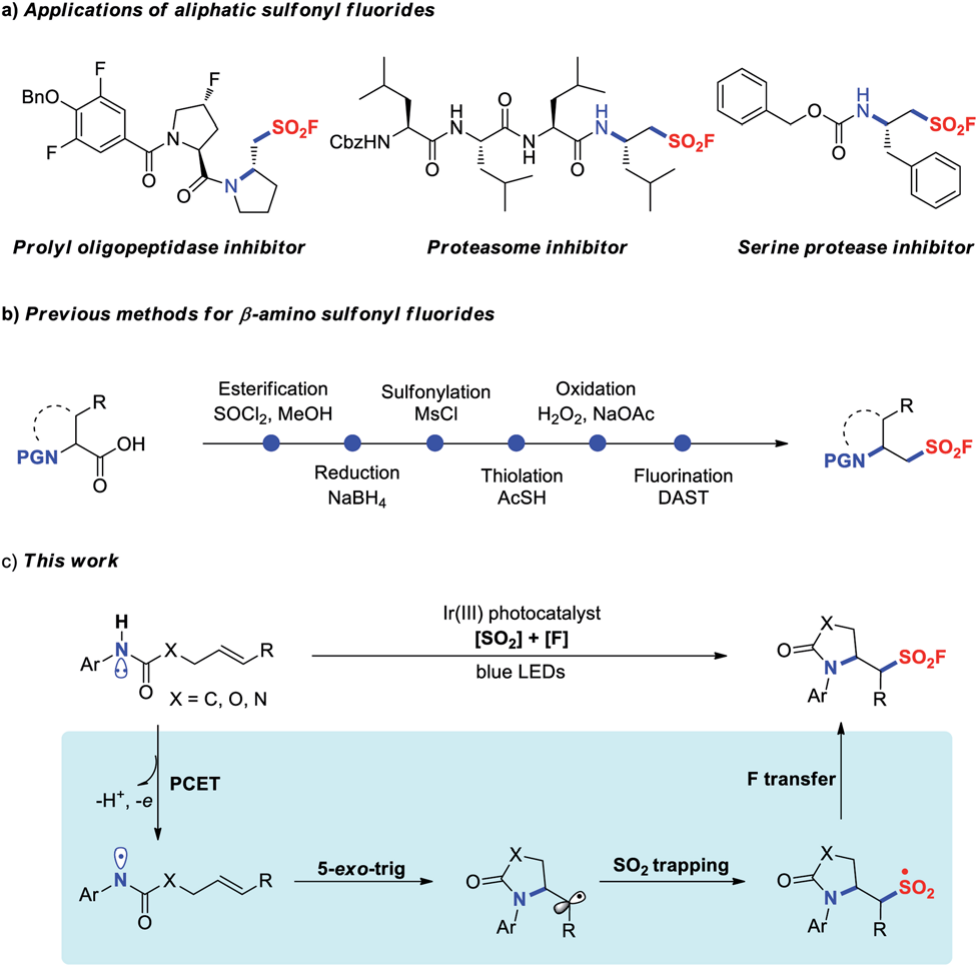
The applications and synthetic methods of β-amino sulfonyl fluorides.

The sulfur(vi) fluoride exchange (SuFEx) reaction revived by Sharpless and co-workers in 2014 is an emerging and promising click reaction that rests on the unique reactivity–stability balance of higher organosulfur fluorides.^1^ Sulfonyl fluorides, some of the most widely used connective hubs for SuFEx click chemistry, have attracted enormous attention and find widespread applications in fields as diverse as organic synthesis,^2^ materials science,^3^ chemical biology and drug discovery.^4^ As a result, the development of efficient approaches for preparing sulfonyl fluorides is undoubtedly in high demand and has become of special interest in synthetic chemistry.^5^ However, compared to the tremendous progress made in the synthesis of aryl sulfonyl fluorides, methods for accessing aliphatic sulfonyl fluorides remain less explored. Conventionally, aliphatic sulfonyl fluorides are prepared *via* fluoride–chloride exchange of corresponding sulfonyl chlorides with fluoride salts.^1^ Alternatively, conversion of alkyl halides, thiols, or sultones into aliphatic sulfonyl fluorides has also been achieved through multistep sequences.^6^ Moreover, ethene sulfonyl fluoride (ESF) has been used as a versatile building block for the synthesis of ethyl sulfonyl fluoride derivatives.^5*a*,*b*,7^ Despite these significant advances, the development of more efficient methods to access aliphatic sulfonyl fluorides is of high interest, because many pharmaceutical agents contain these structural motifs (Fig. 1a).

The direct difunctionalization of alkenes is a powerful strategy for the rapid assembly of molecular complexity and diversity.^8^ With our continuous research interest in sulfonyl fluoride synthesis,^5*a*,9^ we intended to achieve the radical 1,2-difunctionalization of unactivated alkenes providing functionalized aliphatic sulfonyl fluoride derivatives. To the best of our knowledge, the sole example so far is fluoroalkylation–fluorosulfonylation of alkenes recently developed by the group of Liu and Chen.^10^ However, a stoichiometric amount of metal reagent, such as a silver salt or zinc powder, was required to mediate these processes. Therefore, further endeavors to develop redox-neutral fluorosulfonylation involving difunctionalization of alkenes to enrich the structural diversity of the sulfonyl fluoride molecules are highly valuable.

β-Amino-substituted sulfonyl fluorides are unique structural motifs with biologically important activities in various pharmaceuticals, in particular the peptide-type covalent inhibitors as illustrated in Fig. 1a.^4*c*–*f*^ Typically, these compounds were prepared from α-amino acids in a multi-step manner (Fig. 1b).^4*d*–*f*^ Inspired by the significant progress in visible-light photoredox-catalyzed 1,2-difunctionalization of alkenes,^11^ we envisioned that the radical aminofluorosulfonylation might directly provide valuable β-amino sulfonyl fluoride derivatives. Recently, Knowles and co-workers reported the generation of amidyl radicals through photocatalytic proton-coupled electron transfer (PCET) activation of amides,^12^ and various transformations for difunctionalization of alkenes, including aminoalkylation,^13^ hydroamination,^14^ aminoalkynylation,^15^ aminoarylation^16^ and aminoacylation,^17^ were elegantly realized benefiting from the rapid C-centered radical formation through 5-*exo*-trig cyclization of the amidyl radical (*k* = ∼10^5^ s^−1^). Based on our previous experience on aryl sulfonyl fluoride synthesis,^9^ we questioned whether the alkyl radical could be sequentially trapped through SO_2_ insertion and subsequent fluorine transfer, enabling the introduction of both amino and fluorosulfonyl groups across alkenes to access β-amino-substituted sulfonyl fluorides (Fig. 1c).

However, in this process several challenges remain to be addressed: (1) a relatively low thermodynamic driving force for the conversion of amidyl into 1° or 2° alkyl radicals (Δ*G*^0^ ≈ −3 to −5 kcal mol^−1^) was unfavorable for the difunctionalization process;^16,18^ (2) severe competitive reactions such as hydroamination and aminofluorination might be observed;^14,19^ (3) potential incompatibility of photocatalytic conditions with redox-active SO_2_ and fluorine sources. With these challenges in mind, herein we set out to describe a three-component aminofluorosulfonylation of unactivated alkenes by merging photocatalytic PCET activation with a radical relay process.

## Results and discussion

Initially, we conducted an optimization study using *N*-phenyl pent-4-enamide (**1a**) as the model substrate and it is readily accessible from aniline and 4-pentenoic acid. Gratifyingly, when **1a** was treated with DABSO and NFSI in CH_3_CN in the presence of [Ir(dF(CF_3_)ppy)_2_(bpy)]PF_6_ (**PC-II**, *E*_1/2_(*Ir^III^/Ir^II^) = +1.32 V *vs.* SCE)^20^ and K_3_PO_4_ under irradiation with blue LEDs for 10 hours, the desired aminofluorosulfonylation product was smoothly obtained in 64% ^19^F NMR yield (Table 1, entry 1). When the photocatalyst was switched from **PC-II** to others, such as Ir-based photocatalysts [Ir(dF(CF_3_)ppy)_2_(dtbbpy)]PF_6_ (**PC-I**)^21^ and [Ir(dF(CF_3_)ppy)_2_(5,5′-dCF_3_bpy)]PF_6_ (**PC-III**),^14*a*,22^**4CzIPN**,^23^**Eosin Y**,^24^ and **[Ru(bpy)3]Cl2**,^25^ the yields decreased (entries 2–6). Substituting DABSO with other surrogates of sulfur dioxide (such as Na_2_S_2_O_5_ and Rongalite),^26^ or replacing the fluorine donor NFSI with Selectfluor led to a significantly lower conversion or no reaction (entries 7–9). Screening of the bases revealed that K_3_PO_4_ was the optimal choice, while using other inorganic or organic bases resulted in diminished yields (entries 10 and 11). Moreover, control experiments revealed that a photocatalyst and light irradiation were essential for the success of this transformation (entries 12 and 13). In the absence of a base, a lower yield was obtained (entry 14). For full details of the reaction optimization, see the ESI.†

**Table 1:** Optimization of reaction conditions

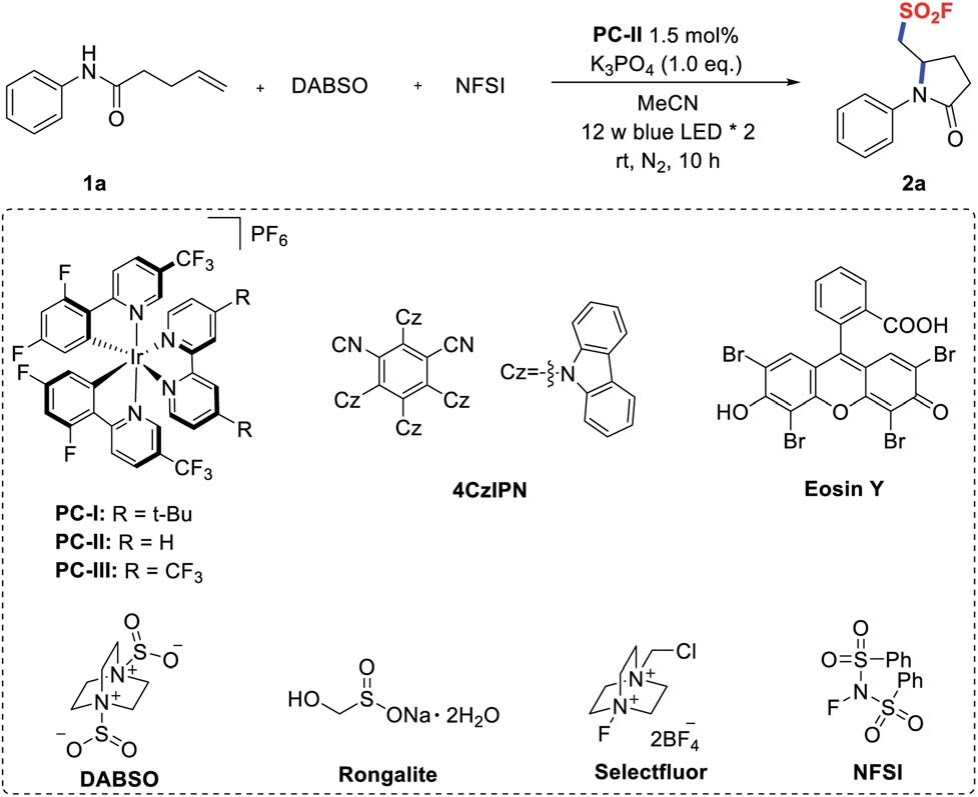		
Entry	Variation from the standard conditions^a^	Yield^b^ (%)
1	None	64 (60)^c^
2	**PC-I** instead of **PC-II**	30
3	**PC-III** instead of **PC-II**	45
4	**4CzIPN** instead of **PC-II**	50
5	**Eosin Y** instead of **PC-II**	N.D.
6	**[Ru(bpy)3]Cl2** instead of **PC-II**	Trace
7	Na_2_S_2_O_5_ instead of DABSO	Trace
8	Rongalite instead of DABSO	N.D.
9	Selectfluor instead of NFSI	Trace
10	K_2_CO_3_ instead of K_3_PO_4_	55
11	Bu_4_N[OP(O) (OMe)_2_] instead of K_3_PO_4_	Trace
12	Without **PC-II**	N.D.
13	Without light	N.D.
14	Without base	50

a Reaction conditions: **1a** (0.1 mmol), DABSO (0.15 mmol, 1.5 eq.), NFSI (0.2 mmol, 2.0 eq.), **PC-II** (1.5 mol%), and K_3_PO_4_ (0.1 mmol, 1.0 eq.) in 4.0 mL MeCN under a N_2_ atmosphere.

b ^19^F NMR yields calculated with PhCF_3_ as the internal standard.

c Isolated yields.

**Scheme 1:**
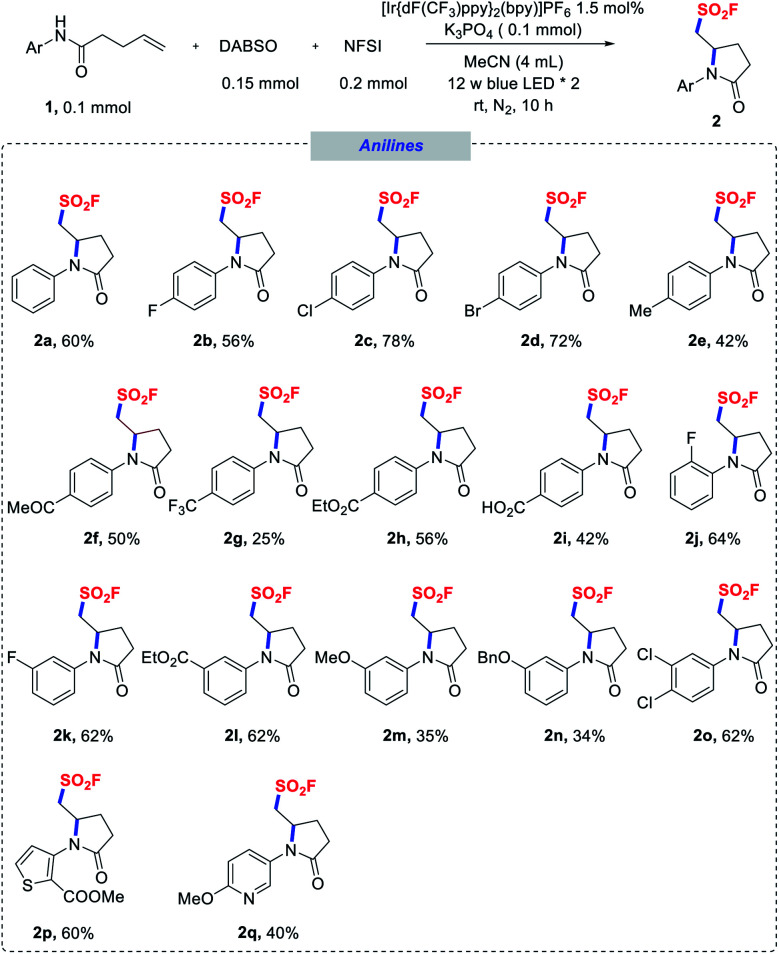
Scope of *N*-(hetero)aryl amides. Reaction conditions as stated in Table 1, entry 1.

**Scheme 2:**
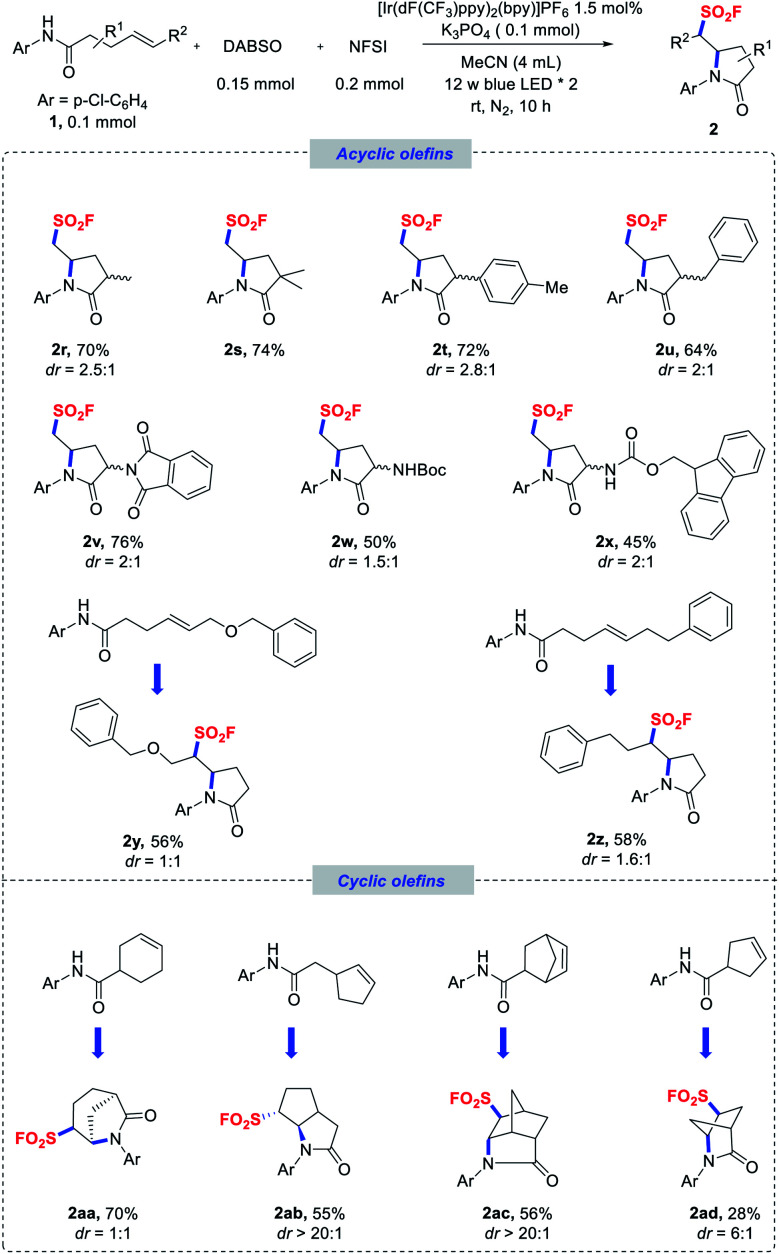
Scope of terminal and nonterminal olefins. Reaction conditions as stated in Table 1, entry 1. Diastereomeric ratios were determined by NMR analysis of the crude reaction mixtures.

**Scheme 3:**
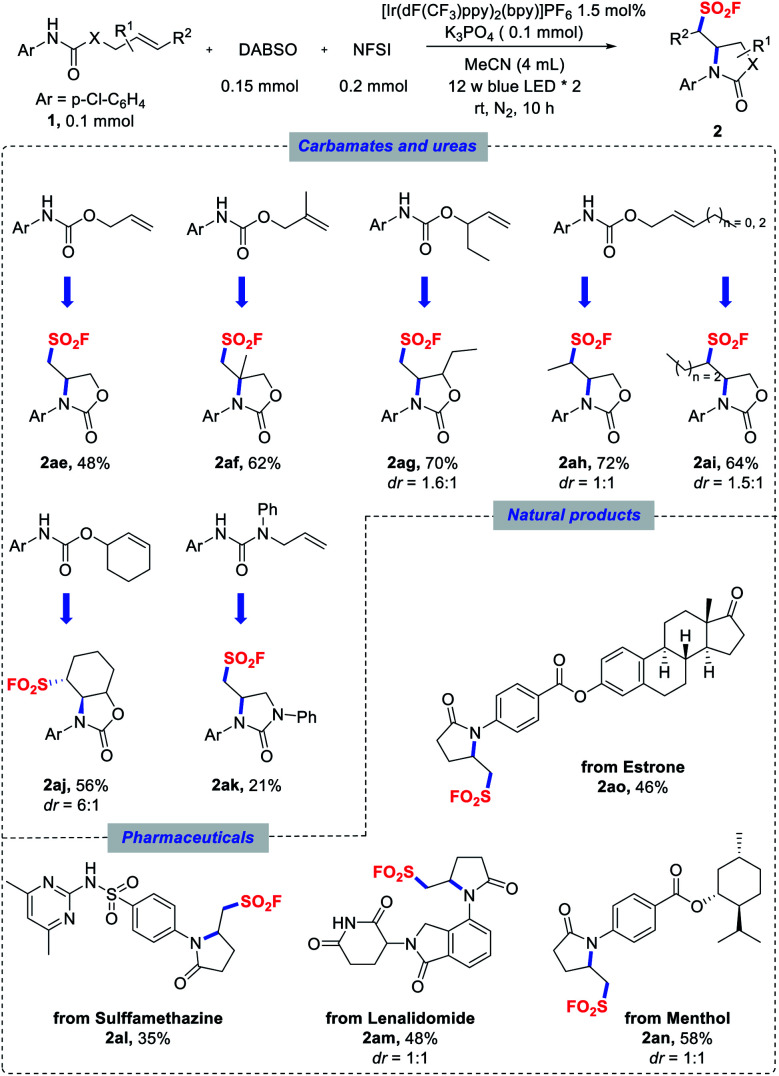
Scope of carbamates, ureas, pharmaceuticals and natural products. Reaction conditions as stated in Table 1, entry 1. Diastereomeric ratios were determined by NMR analysis of the crude reaction mixtures.

With the optimized conditions in hand, we next explored the substrate scope of the aminofluorosulfonylation reactions, and the results are summarized in Schemes 1–3. To our delight, good yields were obtained for a wide range of anilide derivatives bearing different substituents on the arylamine moiety (**2a–o**). Various substrates bearing either electron-withdrawing or electron-donating substituents at the *para* position of the *N*-aryl groups were tolerated under the reaction conditions, furnishing pyrrolidinone-derived sulfonyl fluorides **2a–i** in moderate to good yields. However, lower yields were obtained for substrates with a strongly electron-withdrawing substituent such as CF_3_. The reactions of *ortho*-, *meta*- or di-substituted *N*-aryl amides also proceeded smoothly (**2j–n**). Notably, *N*-heteroaryl amides proved to be competent substrates in this transformation as well (**2p**, **2q**).

With respect to the olefin component, a variety of olefins with different substituent patterns were successfully adapted (**2r–z**). As for terminal olefins, substrates bearing various substituents at the α-carbonyl position, such as methyl, dimethyl, aryl, benzyl, and even bulky protected amino groups, were generally compatible in the reaction and induced moderate diastereoselectivities (Scheme 2, **2r–x**). Nonterminal olefin substrates were also well tolerated to deliver the corresponding secondary alkyl sulfonyl fluorides (**2y–z**) in good yields. Remarkably, substrates bearing an endocyclic double bond could also be applicable for providing more complex fused polycyclic structures (**2aa–ad**) with excellent diastereoselectivities in some cases. It should also be mentioned that NFSI was fully consumed in most of these reactions, and phenylsulfonyl fluoride was obtained as the side product,^27^ which led to incomplete conversion of the amide substrates.

In addition to amide substrates, the aminofluorosulfonylation of carbamates and ureas was also examined under the standard conditions. Acyclic carbamates derived from substituted allylic alcohols could also undergo a cyclization cascade to provide access to sulfonyl fluorides with oxazolidinone backbones (Scheme 3, **2ae–ai**). A cyclohexenol-derivatized carbamate could also be utilized to afford fused bicyclic product **2aj** in good yield and diastereoselectivity. Similarly, β,γ-unsaturated aryl urea could be employed in this transformation to assemble the imidazolidinone scaffold (**2ak**), even though a lower yield was obtained. Furthermore, the potential of this reaction was evaluated with more challenging substrates derived from pharmaceuticals and natural products. The amides derived from sulfamethazine (antibacterial) and lenalidomide (anticancer) were successfully cyclized to deliver sulfonyl fluoride products **2al** and **2am** in moderate yields. Similarly, menthol and estrone derivatives were well tolerated to provide the desired products **2an** and **2ao**, respectively.

**Scheme 4:**
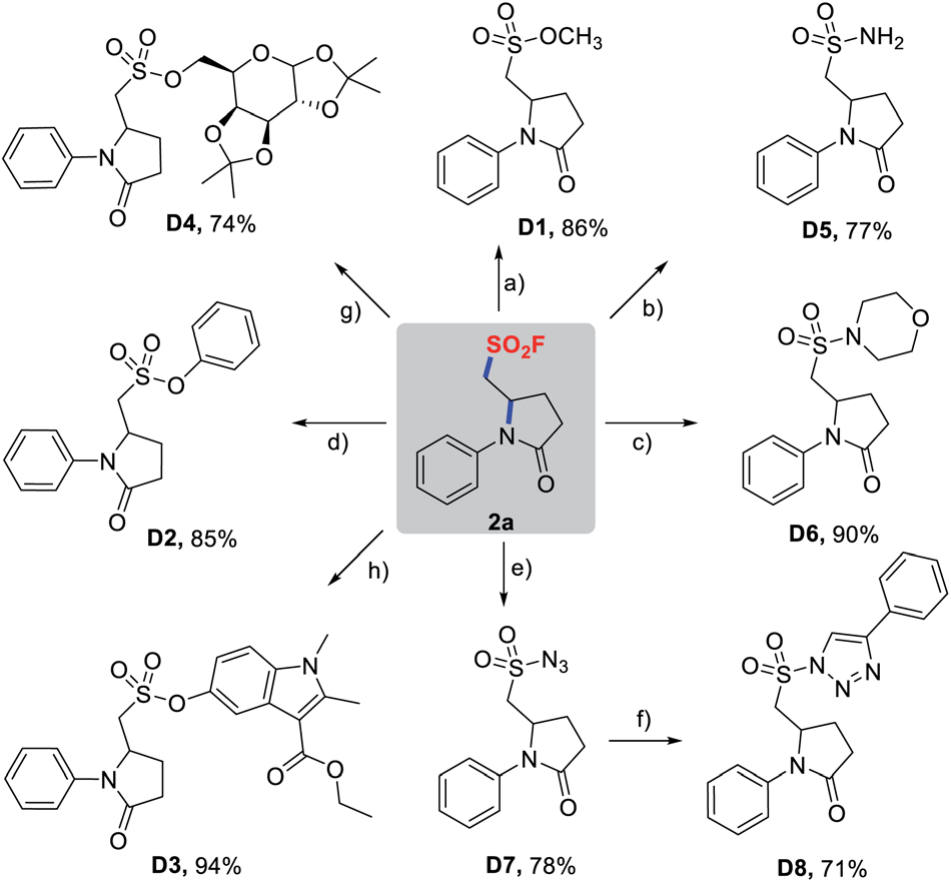
SuFEx reactions of **2a**. Reaction conditions: (a) MeONa, MeOH, rt, 15 min. (b) NH_3_·H_2_O, pyridine, MeCN, 60 °C, 4 h. (c) Morpholine, Et_3_N, MeCN, 80 °C, 24 h. (d) Phenol, Cs_2_CO_3_, MeCN, rt, 12 h. (e) TMSN_3_, DMAP, MeCN, 50 °C, 6 h. (f) Phenylacetylene, CuTC, toluene, rt, 24 h. (g) TMS-protected diacetonefructose, DBU, MeCN, 3 h. (h) TBS-protected mecarbinate, TBAF, MeCN, 2 h.

With success in the preparation of **2a** on a 1 mmol scale without noticeable erosion in yield (58%), we then investigated diversification of **2a** through a wide variety of SuFEx click reactions (Scheme 4). As demonstrated in Scheme 4, pyrrolidinone-based sulfonyl fluoride **2a** readily underwent SuFEx with methanol, phenols, TBS-protected mecarbinate and TMS-protected diacetonefructose, affording the corresponding sulfonate esters **D1–D4** in the presence of a base or silicon additives. Likewise, S(vi)–N bonds were smoothly formed to give sulfonamides **D5**, **D6**, and sulfonyl azide **D7**, and **D7** could be further transformed into sulfonyl triazole **D8***via* a copper-catalyzed azide–alkyne click reaction.

**Scheme 5:**
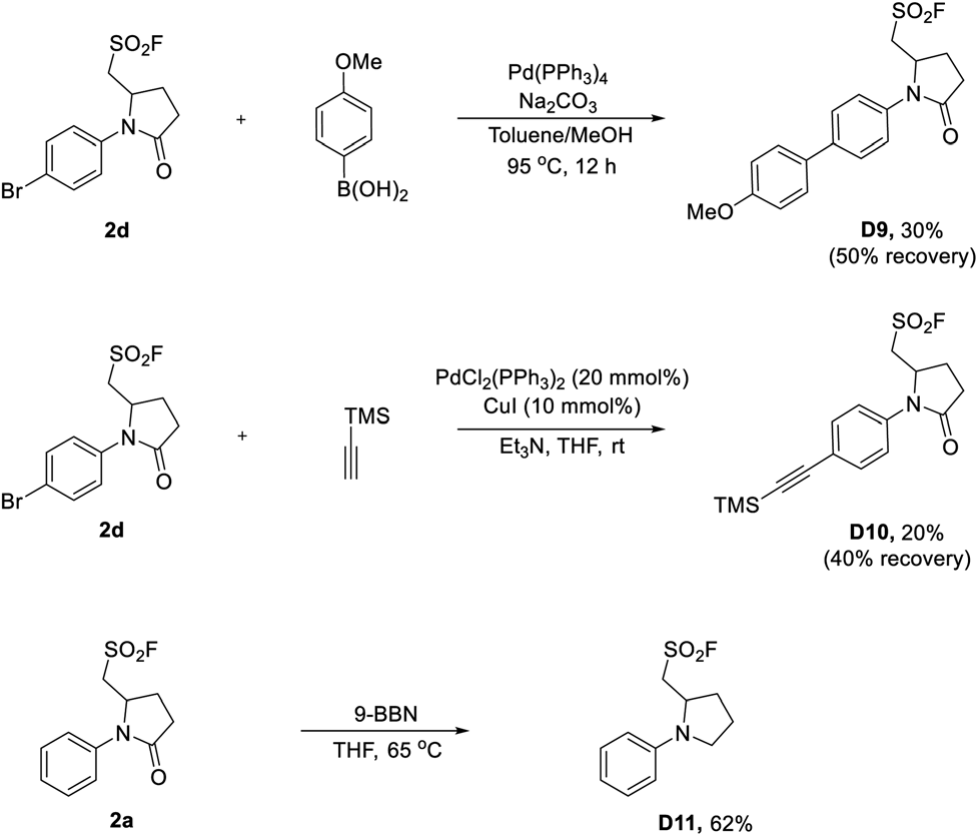
Cross-coupling and reduction reactions of SFs.

The synthetic utility of sulfonyl fluorides was also demonstrated by cross-coupling reactions (Scheme 5). As shown in Scheme 5, **2d** could be used as the coupling partner in Pd-catalyzed Suzuki and Sonogashira reactions, which proceeded chemoselectively at the *para*-bromophenyl moiety of **2d** affording **D9** and **D10** with 30% and 20% yields (not optimized), respectively. Additionally, the pyrrolidinone skeleton of **2a** could be smoothly reduced to pyrrolidine **D11** with 9-borabicyclo[3.3.1]nonane (9-BBN). Taken together, the above-mentioned transformations demonstrated the chemical stability and robustness of alkyl sulfonyl fluorides, and also broaden their applications in organic synthesis.

**Scheme 6:**
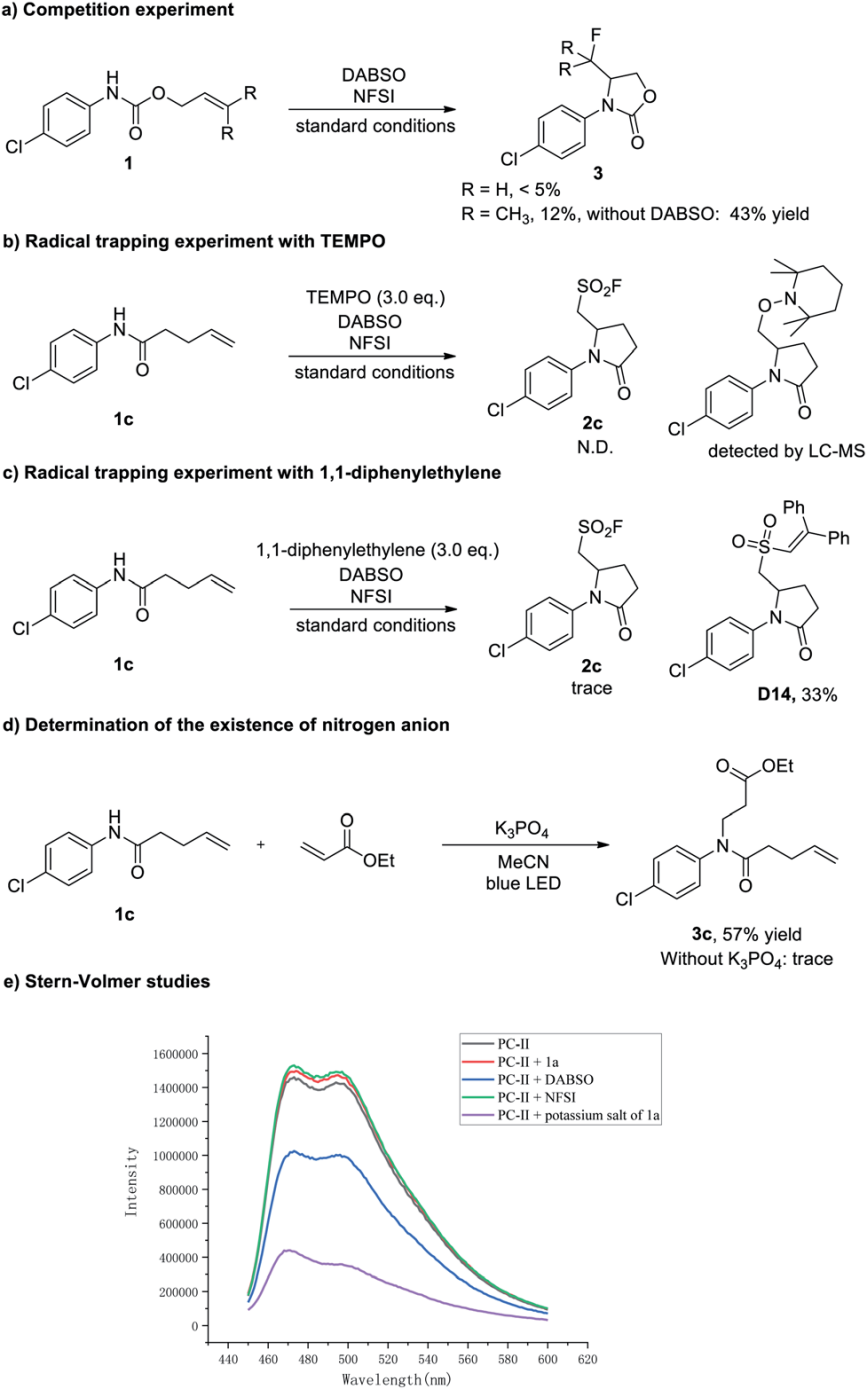
Mechanistic studies.

To gain mechanistic insight into this three-component aminofluorosulfonylation reaction, several control experiments were carried out (Scheme 6). First, the aminofluorination products could be detected in some cases (less than 5% yield). In contrast, when the reaction was performed in the absence of DABSO, the fluorinated product could be isolated in up to 43% yields (Scheme 6a). Then, the formation of **2c** was almost completely suppressed when a radical scavenger TEMPO (2,2,6,6-tetramethyl-1-piperidinyloxy) was added to the reaction, and the trapping product could be detected by LC-MS (Scheme 6b, see the ESI† for details). Next, a trace amount of **2c** was detected when a milder radical scavenger 1,1-diphenylethylene was introduced into this reaction, and the olefination products **D12** and **D13** were detected by LC-MS, which suggested that an amidyl radical might be generated (see the ESI† for details). Meanwhile, the sulfur dioxide insertion product **D14** was isolated in 33% yield (Scheme 6c), indicating the existence of an alkyl sulfonyl radical in this transformation. Furthermore, the observation of an aza-Michael product with ethyl acrylate suggested that the amidyl anion is formed under these conditions^28^ (Scheme 6d). Finally, Stern–Volmer studies showed that the potassium salt of **1a** could quench the excited Ir photocatalyst (Scheme 6e, see the ESI† for details).

**Scheme 7:**
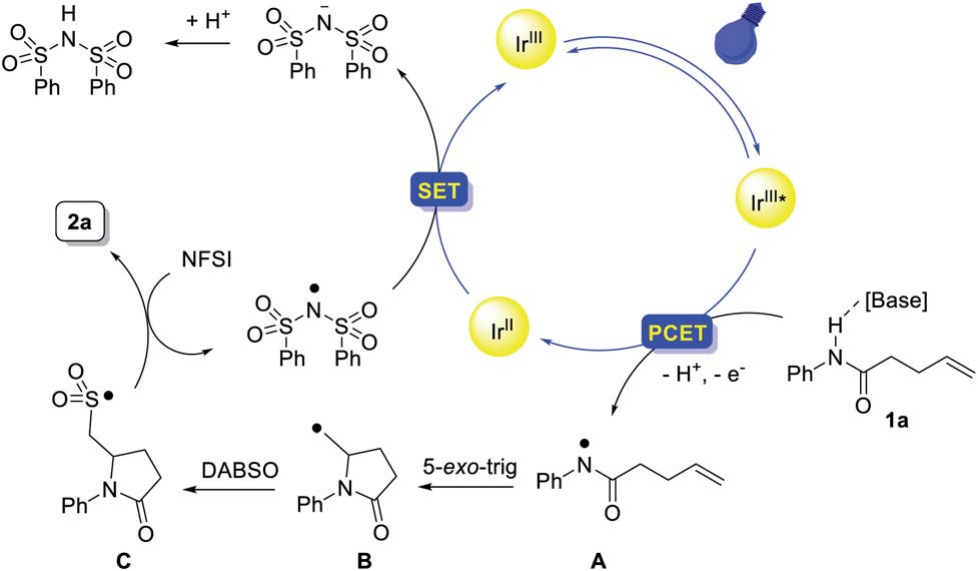
Proposed mechanism.

On the basis of these mechanistic experiments and related literature reports,^12–17,28^ we propose a mechanistic scenario initiated by the formation of an amidyl radical **A** through a stepwise or concerted proton-coupled electron transfer (PCET) process (Scheme 7). Subsequent intramolecular addition to the unactivated olefin results in the formation of a γ-lactam-bearing alkyl radical **B**. Then, trapping of the alkyl radical **B** with SO_2_ affords an alkylsulfonyl radical **C**. Subsequent fluorine atom transfer from NFSI provides the sulfonyl fluoride product. Meanwhile, the (PhSO_2_)_2_N radical generated from *N*-fluorobenzenesulfonimide (NFSI) (*E*_pc_ = −0.78 V *vs.* SCE in MeCN)^29^ could accept one electron from [Ir^II^] to regenerate the photocatalyst (*E*_1/2_(Ir^III^/Ir^II^) = −1.37 V *vs.* SCE).^30^

## Conclusions

In conclusion, the first three-component aminofluorosulfonylation of unactivated alkenes has been developed for the synthesis of sulfonyl fluorides by merging photocatalytic proton-coupled electron transfer (PCET) with radical relay processes. Diverse aliphatic sulfonyl fluorides featuring medicinally privileged heterocyclic scaffolds (pyrrolidinone, oxazolidinone and imidazolidinone) have been efficiently provided under mild conditions, employing easy-to-handle DABSO and NFSI as the sulfur dioxide surrogate and fluorine source, respectively. The SO_2_F-containing products obtained could be used for further diversification through SuFEx click reactions and transition metal-catalyzed cross-coupling reactions. Control experiments and Stern–Volmer studies have revealed that a PCET-based activation is key to the formation of amidyl radicals and subsequent alkyl and sulfonyl radicals. Further elaboration of this difunctionalization strategy for the synthesis of structurally diverse sulfonyl fluorides towards biological applications is ongoing in our laboratory.

## Data availability

The electronic supplementary information include experimental detail, NMR data and HRMS data.

## Author contributions

T. Zhong conducted most of the experiments and wrote the initial manuscript draft. J. T. Yi and Z.-D. Chen performed part of the experiments. Q.-C. Zhuang and Y.-Z. Li contributed to substrate preparation. J. Weng and T. Zhong conceived the project and finalized the manuscript draft. J. Weng and G. Lu directed the project. All authors contributed to discussions.

## Conflicts of interest

There are no conflicts to declare.

## Supplementary Material

SC-012-D1SC02503A-s001
