# Supplementary material for: Photoredox-catalyzed aminofluorosulfonylation of unactivated olefins
Source: Chem Sci. 2021 Jun 7;12(27):9359–65. doi: 10.1039/d1sc02503a (PMC8278970; doi:10.1039/d1sc02503a)

Electronic Supplementary Material (ESI) for Chemical Science.
This journal is © The Royal Society of Chemistry 2021

Photoredox-Catalyzed Aminofluorosulfonylation of Unactivated Olefins

Tao Zhong, Ji-Tao Yi, Zhi-Da Chen, Quan-Can Zhuang, Yong-Zhao Li, Gui Lu, and Jiang Weng*

Guangdong Provincial Key Laboratory of Chiral Molecule and Drug Discovery, School of Pharmaceutical Sciences, Sun Yat-sen University, Guangzhou, 510006, P.R. China.

Table of Contents

1. General Information ……………………………………………………………………….S3

2. Typical Procedure for the Synthesis of Substrates……………………………………….S3

3. Procedure for the Synthesis of Intermediates……………………………………………S5

4. Details for Optimization of Reaction Conditions…………………………………………S6

5. General Procedure for the Synthesis of Sulfonyl Fluorides ………………………….S12

6. Derivatization Reactions of Sulfonyl Fluorides…………………………………………S13

7. Mechanistic Experiments…………………………………………………………………….S16

8. Stern-Volmer studies…………………………………………………………………………S19

9. Large-scale Experiments…………………………………………………………………….S25

10. Characterization Data of Compounds 1d, 1e, 1l-1r, 1t-1ad, 1af-1ao, 2a-2ao, D1-D14, S5...S26

11. References……………………………………………………………………………………S48

12. NMR Spectra of Compounds 1d, 1e, 1l-1r, 1t-1ad, 1af-1ao, 2a-2ao, D1-D14, S5……….S49

1. General Information

All the commercial reagents were used as such without further purification. All solvents were used as commercial anhydrous grade without further purification. The flash column chromatography was carried out over silica gel (230-400 mesh). ^{1}H, ^{13}C and ^{19}F NMR spectra were recorded on a Bruker Avance-300 MHz spectrometer or Bruker Avance-400 MHz spectrometer or Bruker Avance-500 MHz spectrometer. Chemical shifts in ^{1}H NMR spectra were reported in parts per million (ppm, δ) downfield from the internal standard Me_4Si (TMS, δ = 0 ppm). Chemical shifts in ^{13}C NMR spectra were reported relative to the central line of the chloroform signal (δ = 77.0 ppm). Peaks were labeled as singlet (s), doublet (d), triplet (t), quartet (q), and multiplet (m) Low resolution mass spectrometry (LRMS) was performed on a Fisons Platform spectrometer (ESI). High resolution mass spectrometry (HRMS) was performed via electron ionisation (EI) or electrospray ionisation (ESI) sources. The *m/z* ratios are reported in Daltons; high resolution values are calculated to four decimal places from the molecular formula. Chemical yields refer to pure isolated substances.

2. Typical Procedure for the Synthesis of Substrates

Method A:[1] (Scheme S1). A flame-dried round-bottomed flask was degassed, flushed with nitrogen, and charged with dry DCM (6.25 mL, 0.4 M), EDC-HCl (1-(3-dimethylaminopropyl)-3-ethylcarbodiimide hydrochloride, 3.25 mmol, 1.3 eq.), and DMAP (3.5 mmol, 1.4 eq.). The reaction flask was cooled to 0 °C and then the carboxylic acid (2.5 mmol, 1.0 eq.) was added. After five minutes of stirring, the substituted aniline (3 mmol, 1.2 eq.) was added. The ice bath was then removed and the reaction mixture was stirred overnight. Upon completion of reaction (monitored by TLC), the mixture was washed with 1 M HCl and the aqueous layer was extracted with dichloromethane or ethyl acetate for three times. The combined organic layer was washed with brine and dried with Na_2SO_4. The crude product was purified by silica gel column chromatography to afford the desired product.

Scheme S1. Preparation of aryl amide (**Method A**)

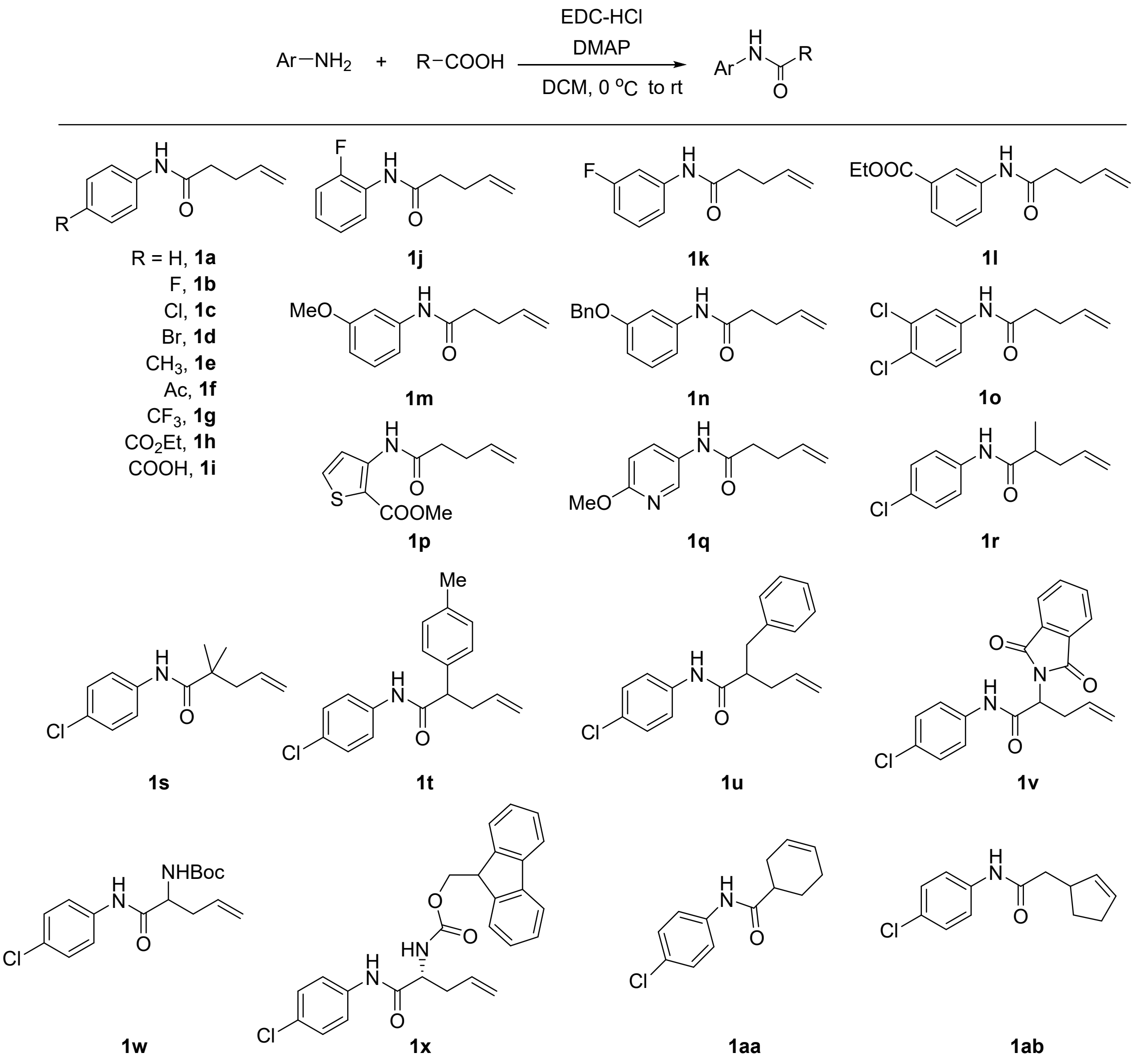

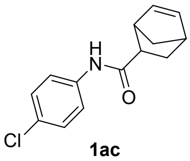

1ac

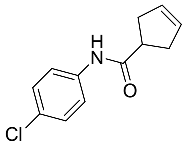

1ad

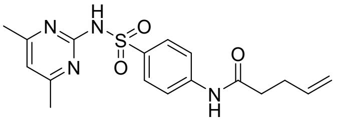

1al

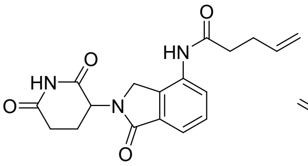

1am

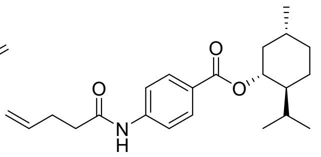

1an

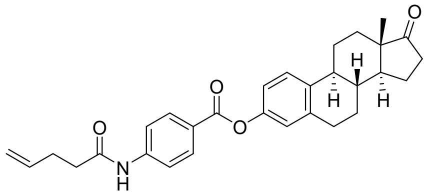

1ao

Method B:[1] (Scheme S2). A flame-dried round-bottomed flask was degassed, flushed with nitrogen, and charged with phenyl isocyanate (2.5 mmol, 1 eq.), dry DCM (5 mL), Et_3N (7.5 mmol, 1.1 eq.) and alcohol/amine (2.5 mmol, 1 eq.). The reaction mixture was stirred at room temperature until the alcohol/amine was fully consumed (monitored by TLC). The reaction mixture was washed with 1 M HCl, water and brine and then dried with Na_2SO_4. Then, the organic phase was concentrated under reduced pressure. The crude product was purified by silica gel column chromatography to afford the desired product.

Scheme S2. Preparation of phenyl Carbamate/urea (**Method B**)

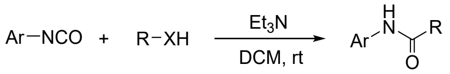

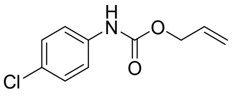

1ae

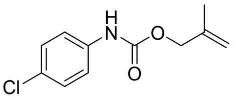

1af

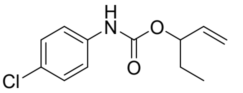

1ag

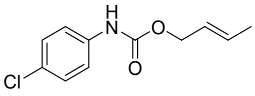

1ah

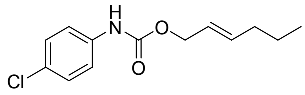

1ai

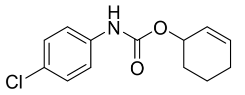

1aj

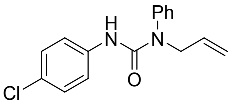

1ak

Method C:[2] (Scheme S3). A flame-dried round-bottomed flask was degassed, flushed with nitrogen, and charged with (3-carboxypropyl)triphenylphosphonium bromide (3 mmol, 1 eq.) and suspended in dry THF (1.0 M). The mixture was cooled to 0 ^oC followed by slow addition of NaHMDS (3 mL, 2.0 M in THF). After 30 minutes of stirring, the corresponding aldehyde was subsequently added dropwise into the reaction (3.6 mmol, 1.2 eq.). The reaction was left to slowly warm to room temperature overnight until starting material was consumed (monitored by TLC). The reaction mixture was quenched by a solution of saturated ammonium chloride solution and the pH was adjusted to 2 by addition of 2 M HCl. Then the aqueous layer was extracted with 50 mL ethyl acetate. After concentrating the organic phase, the residue was directly used in the next step without purification. The reaction flask was degassed, flushed with nitrogen, and charged with CDI (3 mmol, 1.0 eq.) and a solution of the corresponding carboxylic acid (1.0 eq.) in dry THF (1.0 M). After 1 h of stirring, the substituted aniline (3 mmol, 1.0 eq.) was added and the reaction mixture was subsequently stirred overnight. Upon completion of reaction (monitored by TLC), the reaction mixture was taken to dryness under reduced pressure. The crude product was purified by silica gel column chromatography to afford the desired product.

Scheme S3. Preparation of aryl amide (**Method C**)

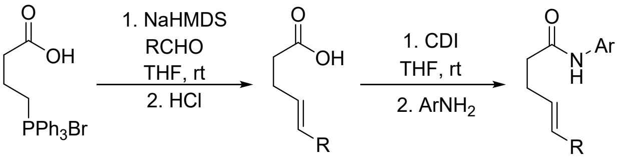

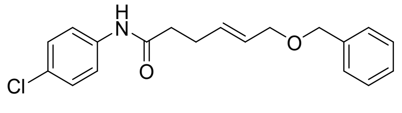

1y

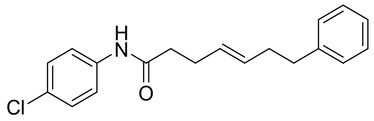

1z

3. Procedure for the Synthesis of Intermediates

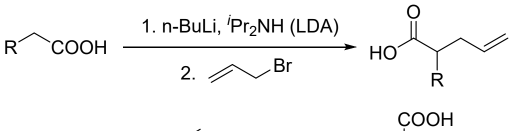

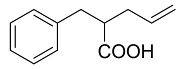

S1

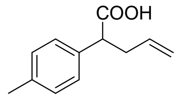

S2

S1 and **S2** was synthesized according to the method of literature. [3] n-BuLi (13 mL, 1.6 M in hexane, 21 mmol) was added dropwise to a cold (0 ^{o}C) solution of iPr$_2$NH (3.0 mL, 21 mmol) in THF (10 mL), and the mixture was stirred for 30 minutes. Then, a solution of carboxylic acid (10 mmol) in THF (10 mL) was added dropwise over 20 minutes and stirring was continued for another 30 minutes at the same temperature. Allyl bromide (0.9 mL, 10.5 mmol) was added, and the stirring was continued for another 6 h. The solvent was removed under reduced pressure. The resulting residue was diluted with water (50 mL) and extracted with ethyl acetate (50 mL). The pH value of the separated aqueous layer was adjusted to 2 by 6 M HCl and then extracted with ethyl acetate. The combined organic layers were washed with brine, dried with Na_2SO_4, and filtered. The solvent was evaporated, and the crude acid was taken forward to the next step without further purification.

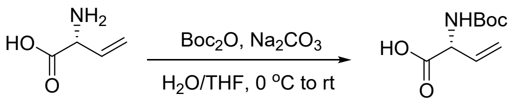

S3

S3 was synthesized according to the method of literature. [4] To a solution of L-allylglycine (576 mg, 5 mmol) in THF/H_2O (10:1, 11 mL) at 0 ^{o}C were added Boc_2O (1.20 g, 5.5 mmol) and Na_2CO_3 (1.06 g, 10 mmol). The reaction was stirred for 16 h at room temperature, after which the reaction mixture was acidified to pH 2 with 2 M HCl and extracted with ethyl acetate. The combined organic layers were washed with brine, dried Na_2SO_4, filtered, and the solvent was removed in vacuo to afford crude (S)-2-((tert-butoxycarbonyl)amino)pent- 4-enoic acid as a colorless oil.

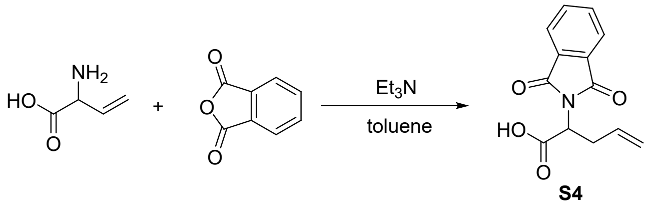

S4

S4 is synthesized according to the method of literature. [4] To a round-bottom flask equipped with a magnetic stir bar was added D/L-2-allylglycine (576 mg, 5 mmol). The flask was evacuated and back-filled with nitrogen three times, and Et_3N (1.39 mL, 10 mmol), toluene (33.3 mL, 0.15 M), and phthalic anhydride (1.481g, 10 mmol) were added. The flask was then fitted with a Dean–Stark

apparatus and refluxed at 140 °C for 16 h. The crude reaction mixture was then concentrated in vacuo, acidified with water and HCl, then extracted into ethyl acetate and washed with water. After concentrating in vacuo, the crude solid was dissolved in aqueous K_2CO_3, washed with ether. The aqueous layer was acidified with HCl and extracted with ethyl acetate. The organic layers were dried over Na_2SO_4 and concentrated in vacuo to afford crude racemic **S4**.

4. Details for Optimization of Reaction Conditions

Table S1. Initial evaluation of solvents and fluorine sources ($Na_2S_2O_5$ as the SO_2 surrogate)

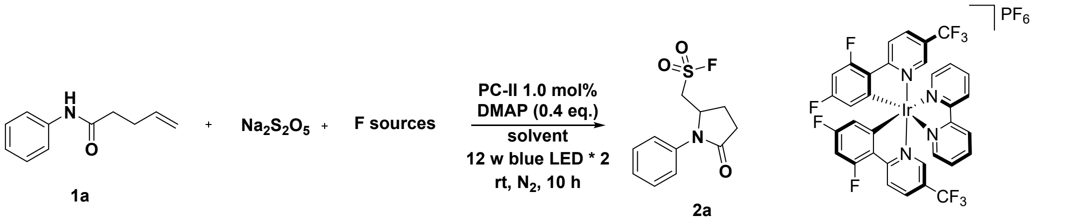

PC-II: $[Ir(dF(CF_3)ppy)_2(bpy)]PF_6$

Entry[a]	SO_2	F source	Solvent (1 mL)	Yield[b] (%)
1	$Na_2S_2O_5$	Selectfluor	$PhCF_3$	trace
**2**	**$Na_2S_2O_5$**	**Selectfluor**	**MeCN**	**16**
3	$Na_2S_2O_5$	Selectfluor	DCE	trace
4	$Na_2S_2O_5$	Selectfluor	DMSO	ND
5	$Na_2S_2O_5$	NFSI	$PhCF_3$	ND
6	$Na_2S_2O_5$	NFSI	MeCN	trace
7	$Na_2S_2O_5$	NFSI	DCE	trace
8	$Na_2S_2O_5$	NFSI	DMSO	ND

[a]: Reaction conditions: **1a** (0.1 mmol), **$Na_2S_2O_5$** (0.2 mmol, 2.0 eq.), **F sources** (0.2 mmol, 2.0 eq.), PC, base in 1.0 mL solvent under N_2 atmosphere; [b]: ^{19}F NMR yields calculated with $PhCF_3$ as internal standard;

Table S2. Initial evaluation of base

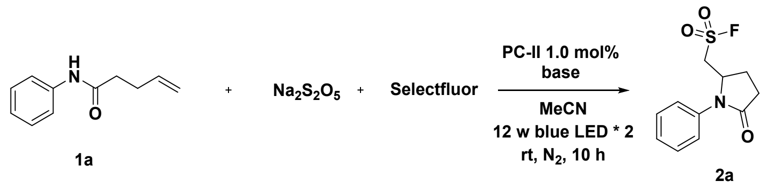

Entry[a]	Base (1.1 eq.)	Additive (1.5 eq.)	Solvent (1 mL)	Yield[b] (%)
1	DMAP (0.4 eq.)		MeCN	16
2	DMAP		MeCN	11
**3**	**K_2CO_3**		**MeCN (2 mL)**	**21**
4	Na_2HPO_4		MeCN (2 mL)	trace
5	DMAP (0.4 eq.)	TBAI	MeCN (2 mL)	ND

[a]: Reaction conditions: **1a** (0.1 mmol), **$Na_2S_2O_5$** (0.2 mmol, 2.0 eq.), **Selectfluor** (0.2 mmol, 2.0 eq.), PC, base in MeCN under N_2 atmosphere; [b]: ^{19}F NMR yields calculated with $PhCF_3$ as internal standard;

Table S3. Evaluation of base and flurine sources (DABSO as the SO_2 surrogate)

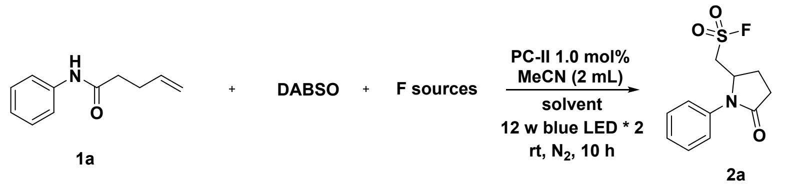

Entry[a]	SO_2	F source	Base (1.1 eq.)	Solvent (2 mL)	Yield[b] (%)
1	DABSO	Selectfluor	DMAP	MeCN	trace
2	DABSO	Selectfluor	K_2CO_3	MeCN	trace
3	DABSO	NFSI	DMAP	MeCN	trace
**4**	**DABSO**	**NFSI**	**K_2CO_3**	**MeCN**	**41**
5	variation from entry 4		without PC		N.D.
6	variation from entry 4		without light		N.D.

[a]: Reaction conditions: **1a** (0.1 mmol), **DABSO** (0.15 mmol, 1.5 eq.), **F sources** (0.2 mmol, 2.0 eq.), PC, base in 2.0 mL MeCN under N_2 atmosphere; [b]: ^{19}F NMR yields calculated with $PhCF_3$ as internal standard;

Table S4. Evaluation of varying amounts of the substrates

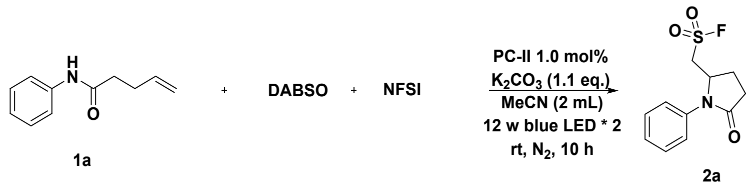

Entry[a]	DABSO	NFSI	Yield[b] (%)
1	1.0 eq.	2.0 eq.	28
2	2.0 eq.	2.0 eq.	30
3[c]	1.5 eq.	2.0 eq.	13
4	1.5 eq.	1.1 eq.	trace
5	1.5 eq.	1.5 eq.	42
**6**	**1.5 eq.**	**2.0 eq.**	**45**
7	1.5 eq.	2.5 eq.	44
8	1.5 eq.	3.0 eq.	45

[a]: Reaction conditions: **1a** (0.1 mmol), **DABCO** (X eq.), **NFSI** (Y eq.), PC, K_2CO_3 (1.1 eq.) in 2.0 mL MeCN under N_2 atmosphere; [b]: ^{19}F NMR yields calculated with $PhCF_3$ as internal standard; [c]: 0.5 mL H_2O was added.

Table S5. Further evaluation of the base

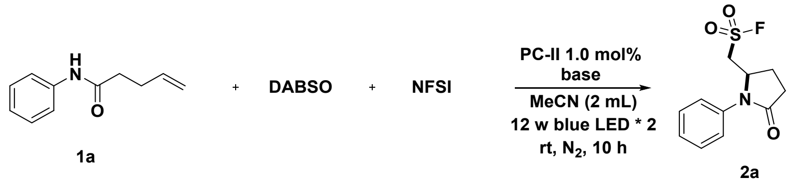

Entry[a]	Base (1.1 eq.)	Yield[b] (%)
1	K_2CO_3	45
2	Na_2CO_3	44
3	$K_3PO_4 \cdot 3H_2O$	42
4	Et_3N	<10
5	Cs_2CO_3	<20
6	KOH	33
7	DABCO	<20
8	Imidazole	33
9	KOAc	37
10	K_2HPO_4	22
11	$NaHCO_3$	22
**12**	**K_3PO_4**	**48**
13	$CaCO_3$	34

[a]: Reaction conditions: **1a** (0.1 mmol), **DABSO** (0.15 mmol, 1.5 eq.), **NFSI** (0.2 mmol, 2.0 eq.), PC, base in 2.0 mL MeCN under N_2 atmosphere; [b]: ^{19}F NMR yields calculated with $PhCF_3$ as internal standard;

Table S6. Evaluation of the reaction concentration

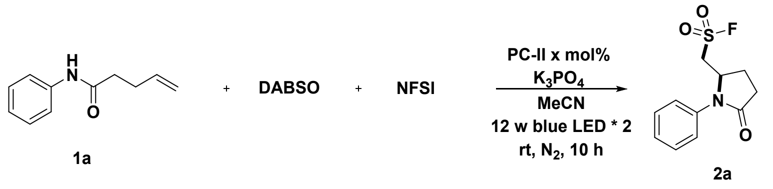

Entry[a]	Base (1.1 eq.)	PC	Solvent 2 mL	Yield[b] (%)
1	K_3PO_4	1 mol%	MeCN	48
2	K_3PO_4	1.5 mol%	MeCN	51
3	K_3PO_4	2.0 mol%	MeCN	48
4	K_3PO_4	1.5 mol%	MeCN (3 mL)	53
5	K_3PO_4	1.5 mol%	MeCN (4 mL)	60
6	K_3PO_4	1.5 mol%	MeCN (5 mL)	54
**7**	**K_3PO_4 (1.0 eq.)**	**1.5 mol%**	**MeCN (4 mL)**	**64**
8	K_3PO_4 (1.2 eq.)	1.5 mol%	MeCN (4 mL)	50
9	K_3PO_4 (1.3 eq.)	1.5 mol%	MeCN (4 mL)	47
10	K_3PO_4 (1.5 eq.)	1.5 mol%	MeCN (4 mL)	48
11	K_3PO_4 (2.0 eq.)	1.5 mol%	MeCN (4 mL)	49

[a]: Reaction conditions: **1a** (0.1 mmol), **DABSO** (0.15 mmol, 1.5 eq.), **NFSI** (0.2 mmol, 2.0 eq.), PC, base in MeCN under N_2 atmosphere; [b]: ^{19}F NMR yields calculated with $PhCF_3$ as internal standard;

Table S7. Selected optimization experiments

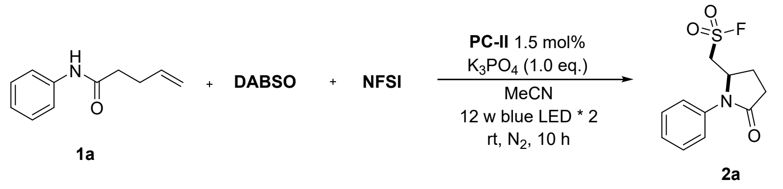

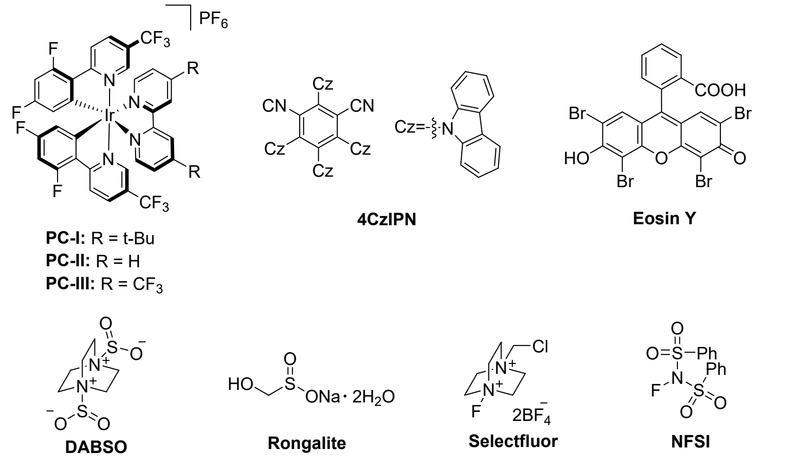

entry	variation from the standard conditions[a]	yield (%)[b]
1	none	64 (60)[c]
2	**PC-I** instead of **PC-II**	30
3	**PC-III** instead of **PC-II**	45
4	**4CzIPN** instead of **PC-II**	50
5	**Eosin Y** instead of **PC-II**	N.D.
6	**[Ru(bpy)$_3$]Cl$_2$** instead of **PC-II**	trace
7	Selectfluor instead of NFSI	trace
8	$Na_2S_2O_5$ instead of DABSO	trace
9	Rongalite instead of DABSO	N.D.
10	K_2CO_3 instead of K_3PO_4	55
11	$Bu_4N[OP(O)(OMe)_2]$ instead of K_3PO_4	trace
12	without **PC 2**	N.D.
13	without light	N.D.
14	without base	50

[a]: Reaction conditions: **1a** (0.1 mmol), **DABSO** (0.15 mmol, 1.5 eq.), **NFSI** (0.2 mmol, 2.0 eq.), PC, K_3PO_4 in 4.0 mL MeCN under N_2 atmosphere; [b]: ^{19}F NMR yields calculated with $PhCF_3$ as internal standard; [c]: Isolated yield.

5. General Procedure for the Synthesis of Sulfonyl Fluorides 2

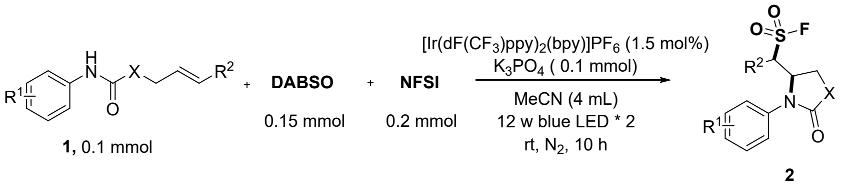

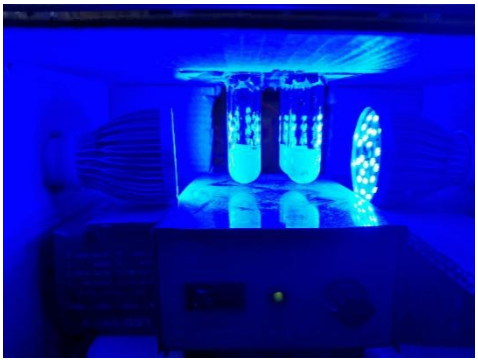

General Procedure: Under N_2 atmosphere, a 10 mL reaction tube was charged with amides **1** (0.1 mmol, 1.0 eq.), DABSO (36.1 mg, 0.15 mmol, 1.5 eq.), NFSI (63.1 mg, 0.2 mmol, 2.0 eq.), [Ir(dF(CF$_3$)ppy)$_2$(bpy)]PF$_6$ (1.5 mg, 1.5 mol%), K_3PO_4 (21.3 mg, 0.1 mmol, 1.0 eq.) and MeCN (4.0 mL), and the reaction mixture was irradiated with two 12 W blue lamps and the heat from light was blown away by fan. After stirring at room temperature for 10 h, the reaction mixture was filtered through a pad of celite, eluted with ethyl acetate, concentrated, and purified by flash column chromatography (eluent: petroleum ether/ethyl acetate) on silica gel to give the desired product **2.**

6. Derivatization Reactions of Sulfonyl Fluorides

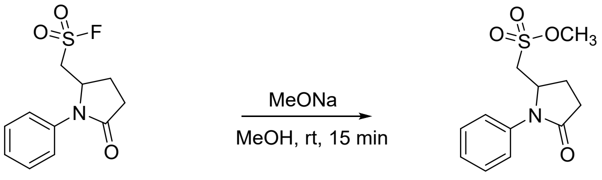

2a **D1,** 86%

To an oven-dried sealed tube equipped with a magnetic stir bar were added sulfonyl fluoride **2a** (25.7 mg, 0.1 mmol, 1.0 eq.), MeONa (27 mg, 0.5 mmol, 5 eq.) and methanol (0.5 mL) under N_2 atmosphere. The mixture was stirred at room temperature for 15 minutes. After removal of the solvent under reduced pressure with a rotary evaporator, the crude product was purified by column chromatography on silica gel (petroleum ether/ethyl acetate 3:1) to give a white solid **D1** (23.2 mg, 86% yield). [5]

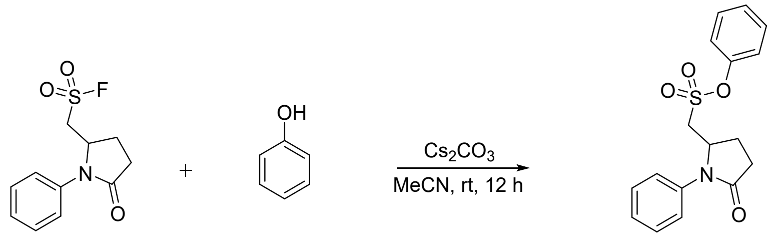

2a **D2**, 85%

To a 5 mL glass vial were added sulfonyl fluoride **2a** (25.7 mg, 0.10 mmol, 1.0 eq.), phenol (10.4 mg, 0.11 mmol, 1.1 eq.), Cs_2CO_3 (65.2 mg, 0.20 mmol, 2.0 eq.), followed by the addition of dry MeCN (0.5 mL). The reaction mixture was stirred at room temperature for 12 h to achieve full conversion. The crude reaction mixture was concentrated in vacuo and purified by flash chromatography (petroleum ether/ethyl acetate 5:1 to 3:1), giving the desired product as a white solid **D2** (28.3 mg, 85% yield). [6]

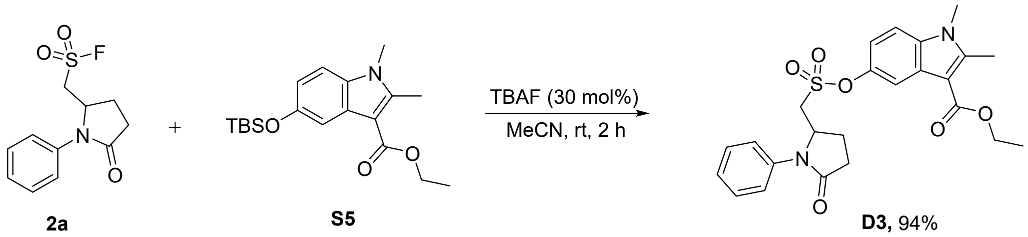

2a **S5** **D3,** 94%

To a solution of sulfonyl fluoride **2a** (25.7 mg, 0.1 mmol, 1.0 eq.), TBS-protected mecarbinate (34.8 mg, 0.11 mmol, 1.1 eq.) in MeCN (1 mL) was added tetrabutylammonium fluoride (TBAF) (30μL, 0.03 mmol, 1 mol in THF) at room temperature. The mixture was stirred at room temperature until the completion of the reaction (2 h) as indicated by TLC. After the solvent was evaporated under vacuum, the residue was purified by flash column chromatography over silica gel (petroleum ether/ethyl acetate 3:1 to 1:1) to afford the product as a white solid **D3** (44 mg, 94%). [7]

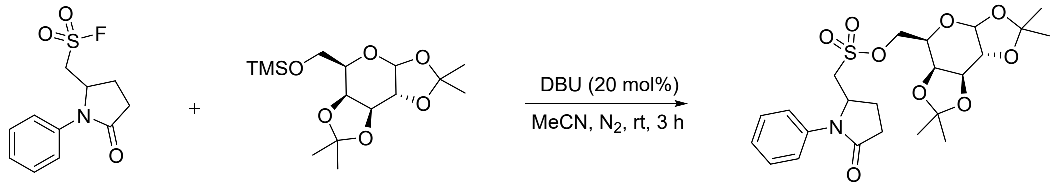

2a **D4,** 74%

In a 10 mL flask, sulfonyl fluoride **2a** (42.5 mg, 0.165 mmol, 1.1 eq.) and TMS-protected alcohol [8] (49.9 mg, 0.15 mmol, 1.0 eq.) were dissolved in anhydrous MeCN (0.5 mL). DBU (4.6 mg, 4.5 μL, 0.03 mmol, 0.2 eq.) was added under a positive nitrogen flow. The flask was sealed and stirred at room temperature for 2 h. The solvent was removed immediately, and the the crude reaction mixture was concentrate in vacuo and purified by flash chromatography (petroleum ether/ethyl acetate 3:1 to 2:1) to afford the pure triazole product as white solids **D4** (55 mg, 74% yield).

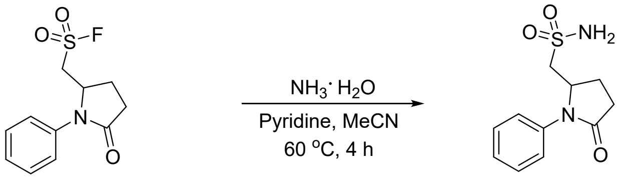

2a **D5,** 77%

To an oven-dried sealed tube equipped with a magnetic stir bar were added sulfonyl fluoride **2a** (51.5 mg, 0.2 mmol, 1.0 eq.), pyridine (31.7 mg, 0.4 mmol, 2.0 eq.), ammonium hydroxide (143 μL, 2.0 mmol, 10 eq.) and MeCN (2 mL) under N_2 atmosphere. The mixture

was stirred at 60 °C for 4 h. After removal of the solvent under reduced pressure with a rotary evaporator, the crude product was purified by column chromatography on silica gel (petroleum ether/ethyl acetate 1:1 to 0:1) to give a colorless oil **D5** (39.2 mg, 77% yield). [5]

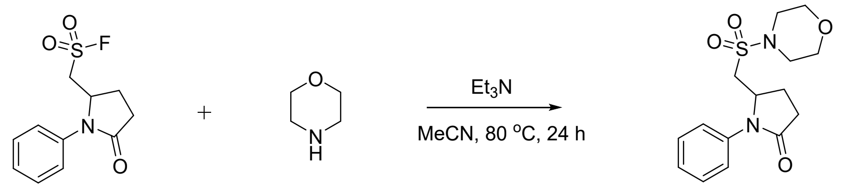

2a **D6,** 90%

Sulfonyl fluoride **2a** (25.7 mg, 0.10 mmol, 1.0 eq.) was added to a solution of morpholine (17.4 mg, 0.20 mmol, 2.0 eq.) and triethylamine (28 μL, 0.2 mmol, 2.0 eq.) in MeCN (0.1 mL). The reaction mixture was stirred at 80 °C for 24 h to achieve full conversion. The crude reaction mixture was concentrated in vacuo and purified by flash chromatography (petroleum ether/ethyl acetate 5:1 to 3:1) to give the product as a white solid **D6** (29.1 mg, 90% yield). [6]

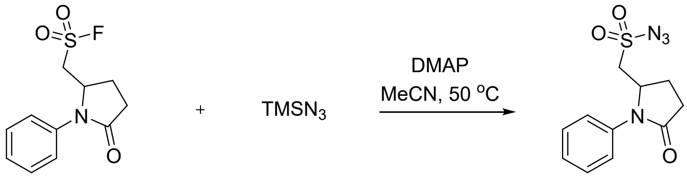

2a **D7,** 78%

To a solution of sulfonyl fluoride **2a** (51.5 mg, 0.2 mmol, 1.0 eq.) in MeCN (0.4 mL) at 50 °C was added DMAP (36.7 mg, 0.3 mmol, 1.5 eq.) followed by $TMSN_3$ (20 μL, 0.15 mmol). The solution was stirred at 50 °C for 15 minutes, then further two portions of $TMSN_3$ (20 μL, 0.15 mmol) were added at intervals of 15 minutes. The solution was stirred for 6 h to achieve full conversion. The crude reaction mixture was concentrated in vacuo and purified by flash chromatography (petroleum ether/ethyl acetate 5:1 to 3:1) to give the product as a white solid **D7** (43 mg, 78% yield). [6]

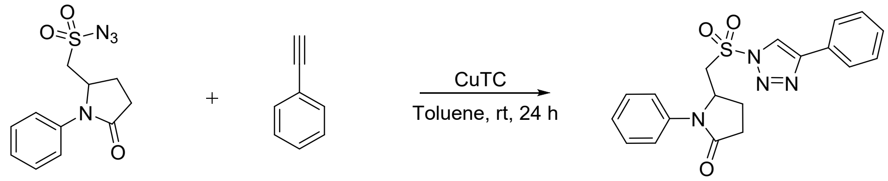

D5 **D8,** 71%

To a dry toluene (0.4 mL) suspension of CuTC (1.9 mg, 0.01 mmol), was added alkyne (11 μL, 0.10 mmol, 1.0 eq.) with vigorous stirring. After 10 minutes, a toluene (0.1 mL) solution of sulfonyl azide **D7** (30.9 mg, 0.11 mmol, 1.1 eq.) was added dropwise over 15 minutes. The reaction was stirred at room temperature until complete consumption of the alkyne by TLC (24 h). The crude reaction mixture was concentrate in vacuo and purified by flash chromatography (petroleum ether/ethyl acetate 5:1) to afford the pure triazole product as white solids **D8** (27 mg, 71% yield). [6]

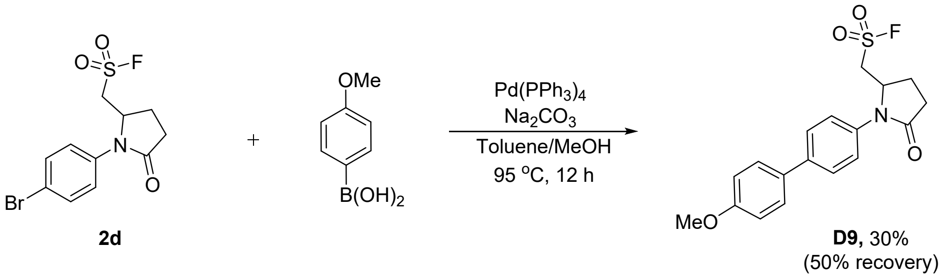

2d **D9,** 30% (50% recovery)

To a 10 mL Schlenk flask purged with N_2 gas was added sulfonyl fluoride **2d** (33.6 mg, 0.1 mmol, 1.0 eq.), $Pd(PPh_3)_4$ (5.8 mg, 0.005 mmol, 5 mol%), sodium carbonate (21.2 mg, 0.2 mmol, 2.0 eq.) and phenylboronic acid (22.8 mg, 0.15 mmol, 1.5 eq.). Then degassed anhydrous toluene (0.4 mL) and methanol (0.1 mL) was added by syringe. The reaction mixture was stirred at 95 °C for 12 h. After cooling to room temperature, the solvent was removed under vacuum, and the residue was purified by column chromatography on silica gel (petroleum ether/ethyl acetate 5:1) to afford the product as a white solid **D9** (10.9 mg, 30% yield) and the recovery **2d** (16.8 mg, 40%). [9]

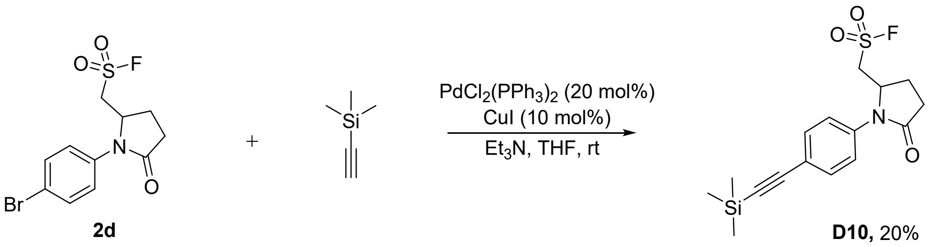

2d **D10,** 20% (40% recovery)

To a solution of sulfonyl fluoride **2d** (33.6 mg, 0.1 mmol, 1.0 eq.) in THF (0.5 mL) was added ethynyltrimethylsilane (51 μL, 0.36 mmol, 3.6 eq.), Et_3N (69.5 μL, 0.5 mmol, 5.0 eq.), and CuI (1.9 mg, 0.01 mmol, 10 mol%). The resulting mixture was stirred in glove box for 20 minutes, at which time $PdCl_2(PPh_3)_2$ (14.1 mg, 0.02 mmol, 20 mol%) was added. The reaction mixture was stirred overnight and then concentrated. Flash chromatography on silica gel (petroleum ether/ethyl acetate 5:1 to 3:1) afforded the product as a white solid **D10** (7.0 mg, 20% yield) and the recovery **2d** (13.4 mg, 40%). [10]

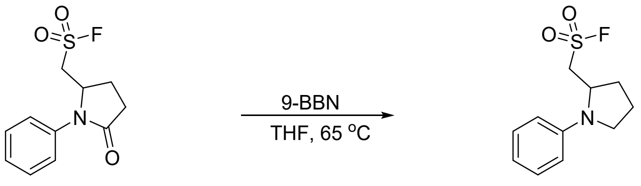

2a **D11,** 62%

In a 5 mL flask, sulfonyl fluoride **2a** (42.5 mg, 0.165 mmol, 1.1 eq.), 9-BBN (0.44 mL, 0.22 mmol, 0.5 M in THF) were added in a glove box under a nitrogen atmosphere. The reaction mixture was heated to 65 °C. The reaction was complete after refluxing for 1 h. After cooling to room temperature, the solvent was removed under vacuum, and the residue was purified by column chromatography on silica gel (petroleum ether/ethyl acetate 20:1) to afford the product as a white solid **D11** (15 mg, 62% yield). [11]

7. Mechanistic Experiments

(1) Radical trapping experiments with TEMPO

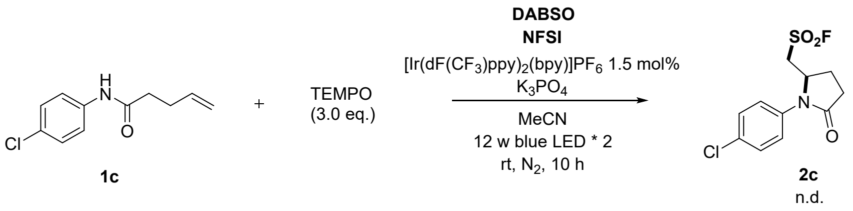

Under N_2 atmosphere, a 10 mL reaction tube was charged with amide **1c** (0.1 mmol, 1.0 eq.), DABSO (36.1 mg, 0.15 mmol, 1.5 eq.), NFSI (63.1 mg, 0.2 mmol, 2.0 eq.), $[Ir(dF(CF_3)ppy)_2(bpy)]PF_6$ (1.5 mg, 1.5 mol%), K_3PO_4 (21.3 mg, 0.1 mmol, 1.0 eq.), TEMPO (94 mg, 0.6 mmol, 3 eq.) and MeCN (4.0 mL), and the reaction was carried out under the illumination of two 12 W blue lamps and the heat from light was blown away by fan. After stirring at room temperature for 10 h, TLC and LC-MS analysis demonstrated the sulfonyl fluoride **2c** is not founded, and the amidyl radical or γ-lactam bearing alkyl radical combined with TEMPO were detected by LC-MS.

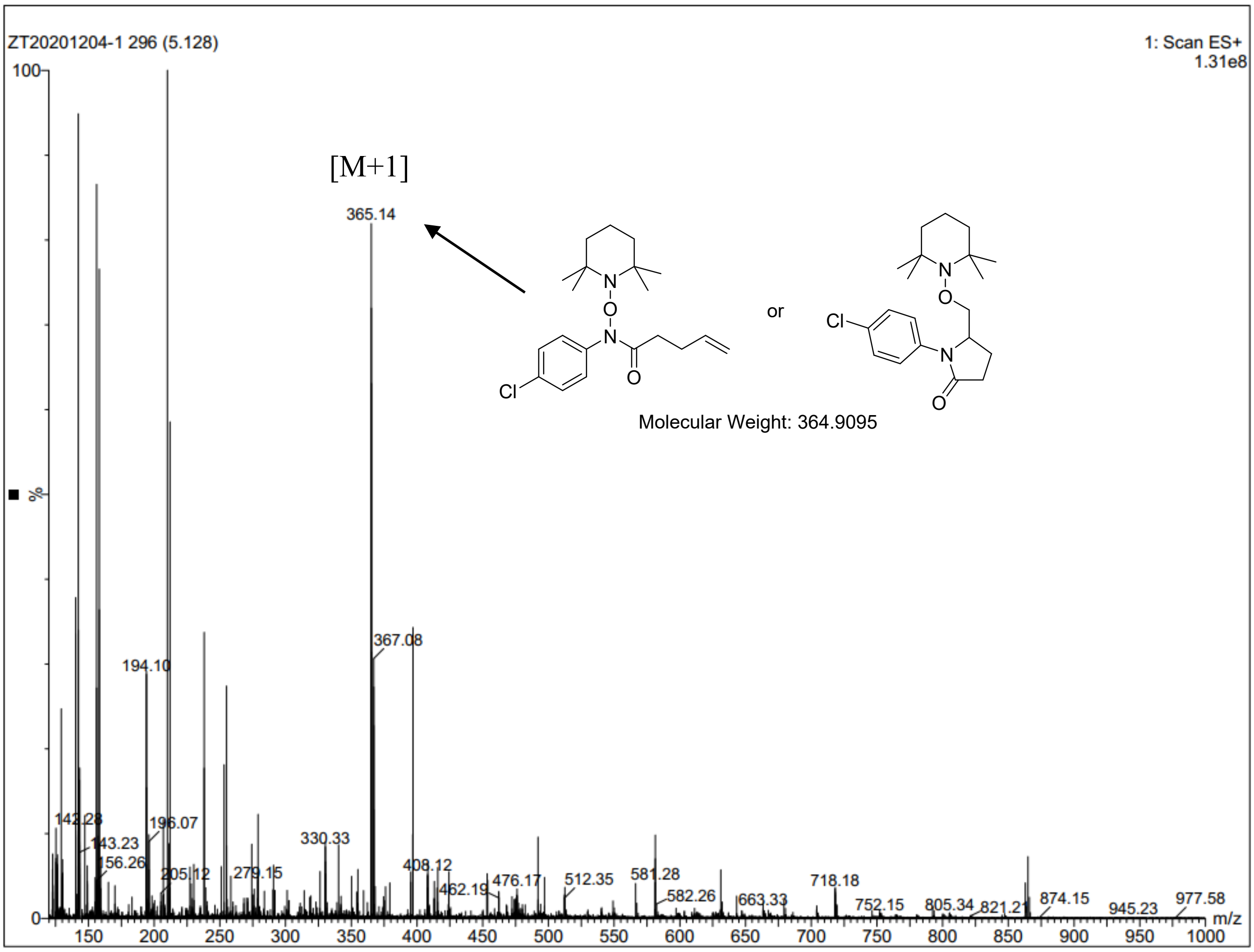

(2) Radical trapping experiments with 1,1-diphenylethylene

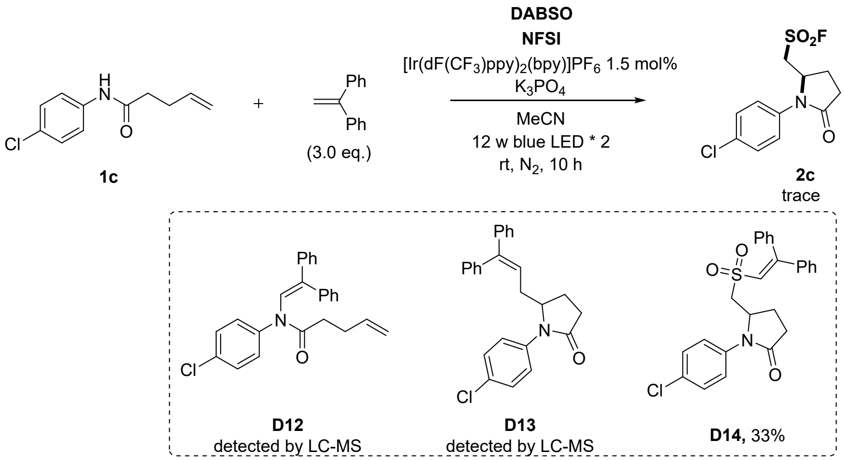

Under N_2 atmosphere, a 10 mL reaction tube was charged with amide **1c** (0.1 mmol, 1.0 eq.), DABSO (36.1 mg, 0.15 mmol, 1.5 eq.), NFSI (63.1 mg, 0.2 mmol, 2.0 eq.), [Ir(dF(CF_3)ppy)$_2$(bpy)]PF_6 (1.5 mg, 1.5 mol%), K_3PO_4 (21.3 mg, 0.1 mmol, 1.0 eq.), 1,1-diphenylethylene (108 mg, 0.6 mmol, 3 eq.) and MeCN (4.0 mL), and the reaction was carried out under the illumination of two 12 W blue lamps and the heat from light was blown away by fan. After stirring at room temperature for 10 h, TLC analysis demonstrated only a small amount of sulfonyl fluoride **2c** was formed, and the **D12** or **D13** were determined by LC-MS. **D14** was isolated in 33% yield.

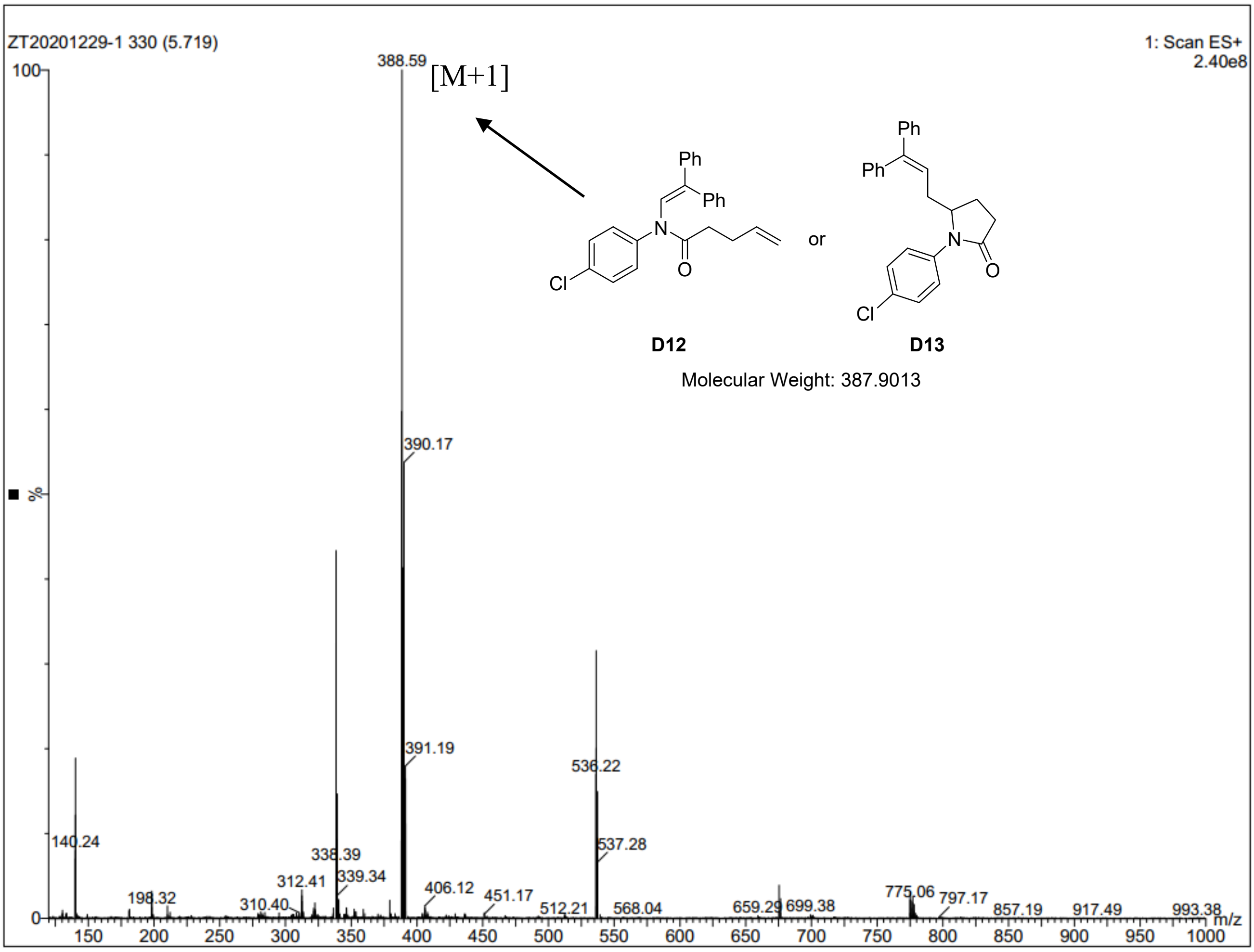

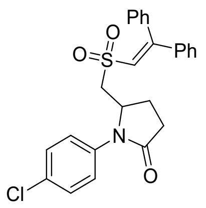

1-(4-chlorophenyl)-5-(((2,2-diphenylvinyl)sulfonyl)methyl)pyrrolidin-2-one (D14)

Compound **D14** was prepared according to **General Procedure** with additional 1,1-diphenylethylene (108 mg, 0.6 mmol, 3 eq.). The residue was purified by flash column chromatography on silica (petroleum ether/ethyl acetate 3:1) to give a white solid (14.9 mg, 33% yield).

^{1}H NMR (400 MHz, $CDCl_3$) δ 7.46 – 7.37 (m, 2H), 7.38 – 7.28 (m, 8H), 7.22 (dd, *J* = 11.0, 4.0 Hz, 4H), 6.78 (s, 1H), 4.69 (ddd, *J* = 13.0, 6.2, 3.8 Hz, 1H), 2.96 (dd, *J* = 13.4, 1.7 Hz, 1H), 2.77 (dd, *J* = 13.4, 10.3 Hz, 1H), 2.57 – 2.43 (m, 3H), 2.19 (dt, *J* = 10.6, 9.0 Hz, 1H). ^{13}C NMR (101 MHz, $CDCl_3$) δ 173.8, 157.0, 138.7, 135.2, 135.1, 131.4, 131.0, 129.8, 129.8, 129.6, 128.9, 128.4, 128.3, 126.9, 124.0, 56.0, 53.8, 30.6, 24.6. **HRMS (ESI):** *m/z* calculated for [M] ($C_{25}H_{22}NO_3SCl$) from $[M+H]^+$ is 452.1082, found 452.108

(3) Determination of the existence of nitrogen anion

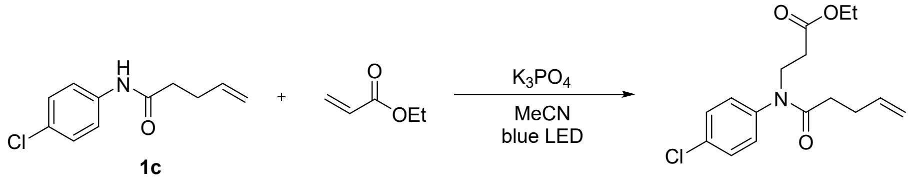

1c

3c, 57% yield
Without K_3PO_4: trace

Under N_2 atmosphere, a 10 mL reaction tube was charged with amide **1c** (0.1 mmol, 1.0 eq.), ethyl acrylate (0.15 mmol,1.5 eq.), with or without K_3PO_4 (21.3 mg, 0.1 mmol, 1.0 eq.), MeCN (4.0 mL), and the reaction was carried out under the illumination of two 12 W blue lamps and the heat from light was blown away by fan. After stirring at room temperature for 10 h, TLC analysis demonstrated that with the help of K_3PO_4, an aza-Michael product was formed, and only trace mount was formed without K_3PO_4. [12] The aza-Michael product and amide **1c** couldn't be separated for their similar polarity, but it can be demonstrated in ^{1}H-NMR analysis as the following figure.

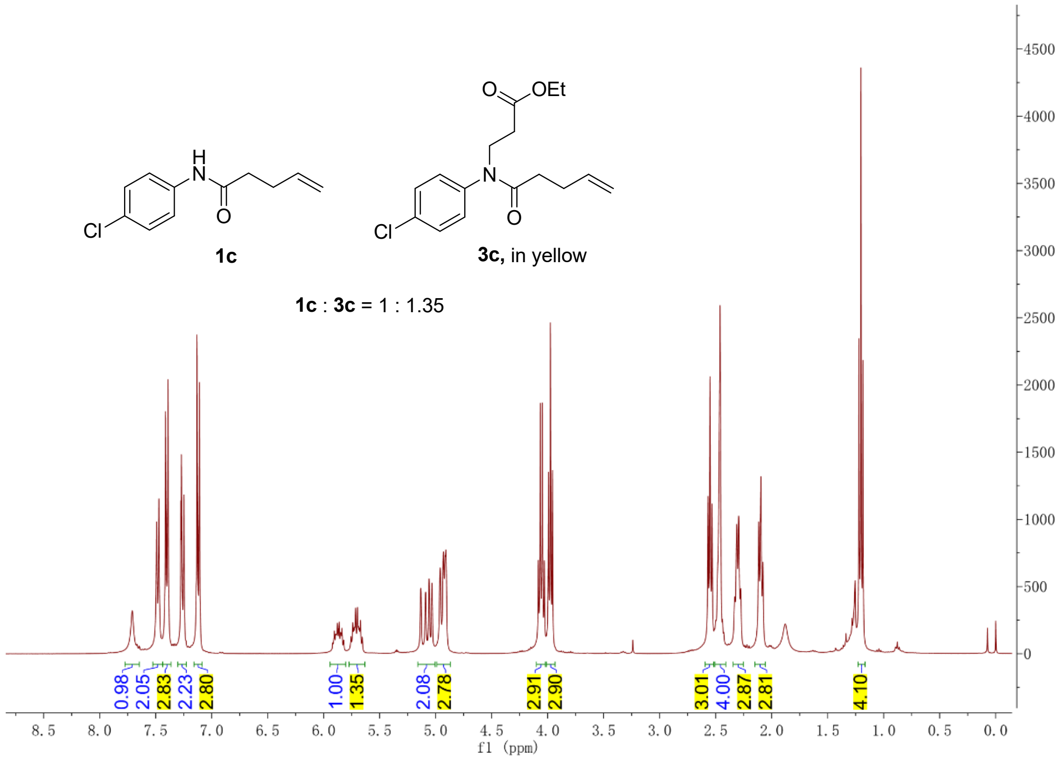

8. Stern-Volmer studies

Stern-Volmer studies were carried out with Fluoromax-4 (HORIBA Instruments Incorporated). Different solutions containing 0.2 mM **PC-II** and x mM substrates were irradiated at 370 nm and luminescence was measured at 473 nm.

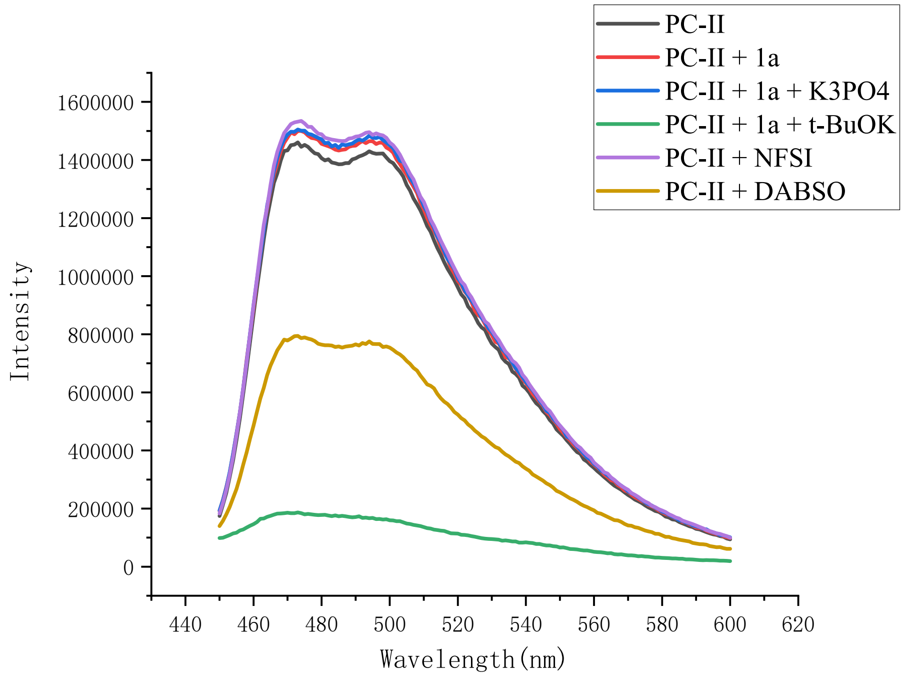

Figure S1 Fluorescence quenching of the excited **PC-II** with each sample in MeCN

Samples were excited at 370 nm. **PC-II** (0.2 mM) in MeCN (black line), **PC-II** (0.2 mM) with **1a** (2.0 mM) in MeCN (red line), **PC-II** (0.2 mM) with **1a** and K_3PO_4 (2.0 mM) in MeCN (blue line), **PC-II** (0.2 mM) with **1a** and *t*-BuOK (2.0 mM) in MeCN (green line), **PC-II** (0.2 mM) with **NFSI** (2.0 mM) in MeCN (purple line), **PC-II** (0.2 mM) with **DABSO** (2.0 mM) in MeCN (brown line).

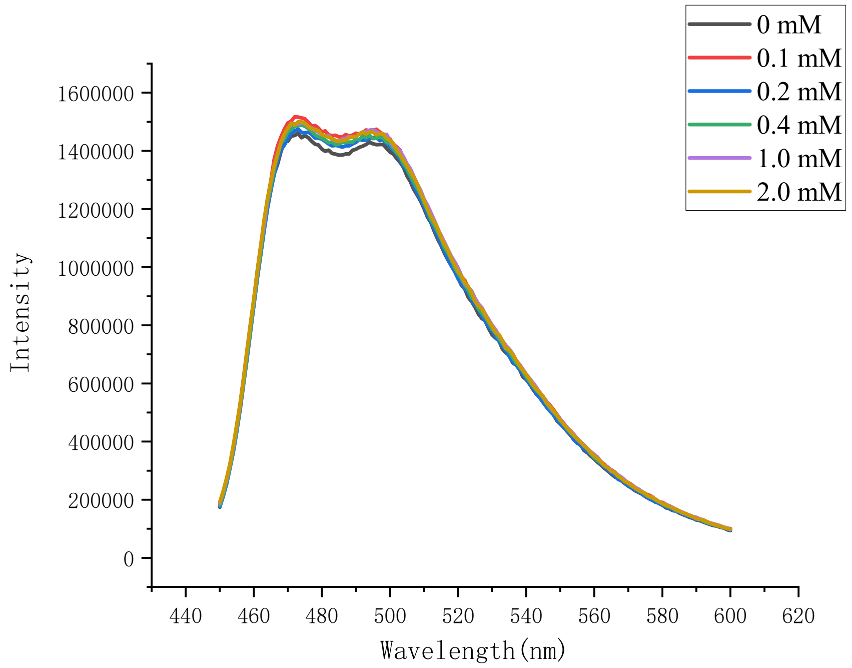

Figure S2 Fluorescence quenching of the excited **PC-II** with different concentrations of **1a**

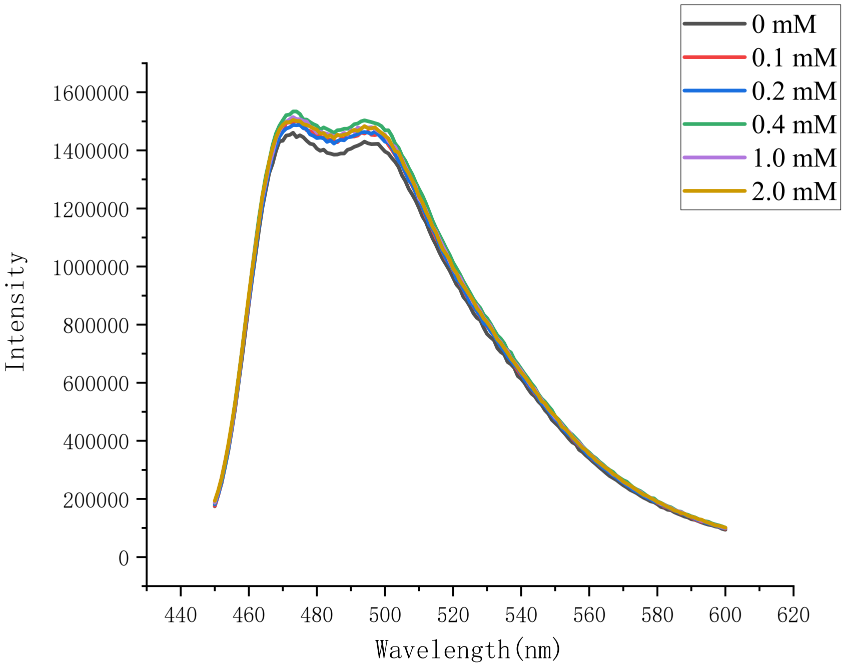

Figure S3 Fluorescence quenching of the excited **PC-II** with different concentrations of **1a** and 1.0 eq. K_3PO_4

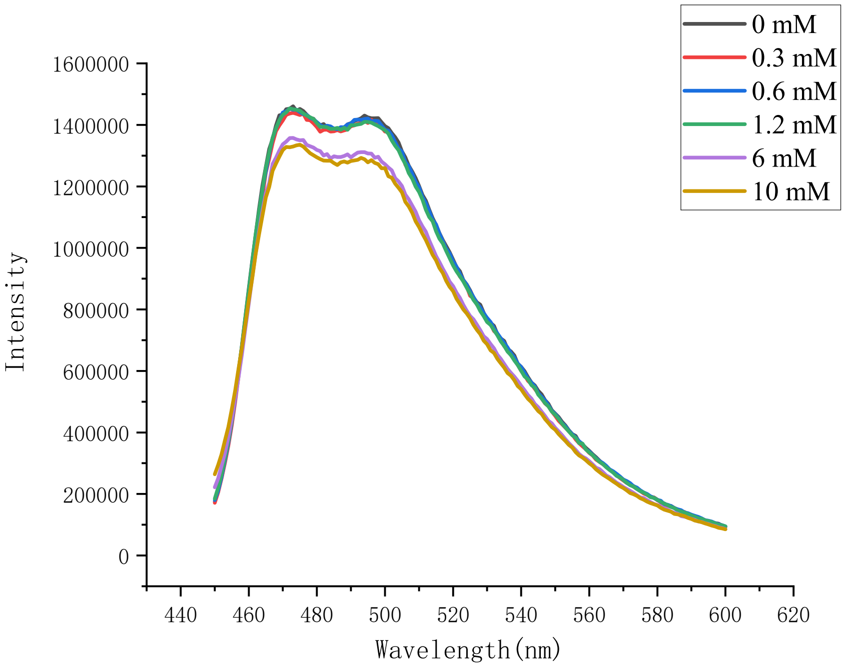

Figure S4 Fluorescence quenching of the excited **PC-II** with different concentrations of **1a** and 2.0 eq. K_3PO_4

Given that different concentrations of **1a** and 1.0 eq. K_3PO_4 can't quench the excited **PC-II** photocatalyst, we try to increase the concentration of **1a** and double the amount of K_3PO_4. As shown in **Figure S4**, when the concentration of **1a** and K_3PO_4 was increase to 6 mM and 10 mM, a slight quenching was obtained. Therefore, base on the previous works,[12] we replaced K_3PO_4 with *t*-BuOK to increase the basicity so that favorably form potassium salt of **1a**. As shown in **Figure S5**, obvious quenching was obtained. The result shows that the **PC-II** photocatalyst can be quenched by the potassium salt of **1a**.

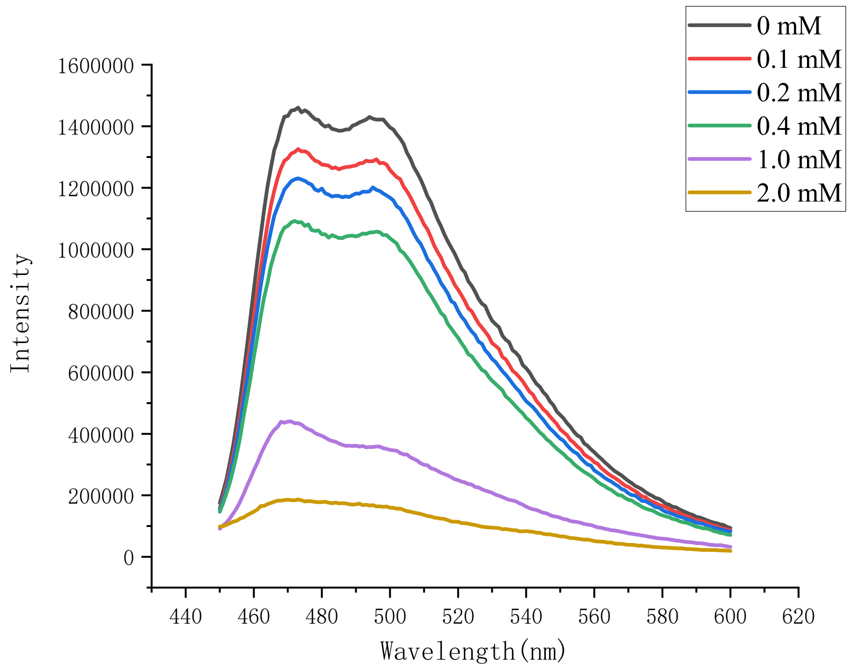

Figure S5 Fluorescence quenching of the excited **PC-II** with different concentrations of **1a** and 1.0 eq. *t*-BuOK

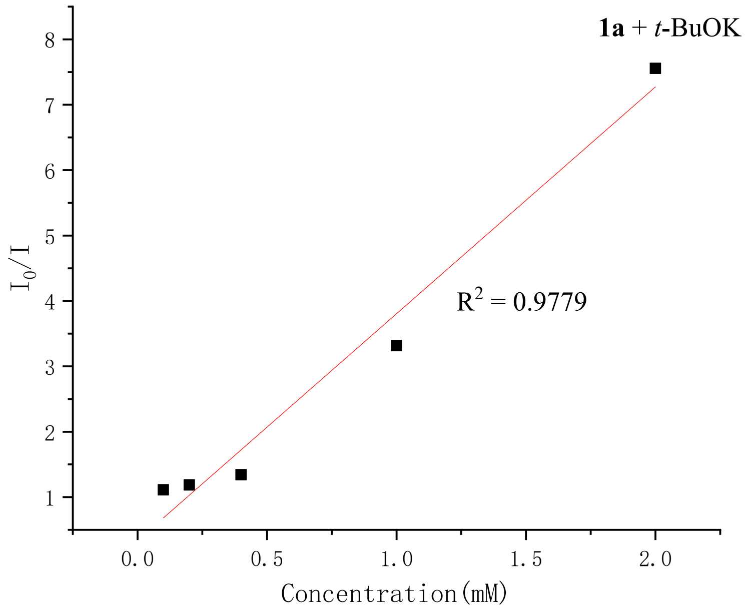

Figure S6 Stern-Volmer emission quenching studies of the excited **PC-II** by **1a** and 1.0 eq. *t*-BuOK

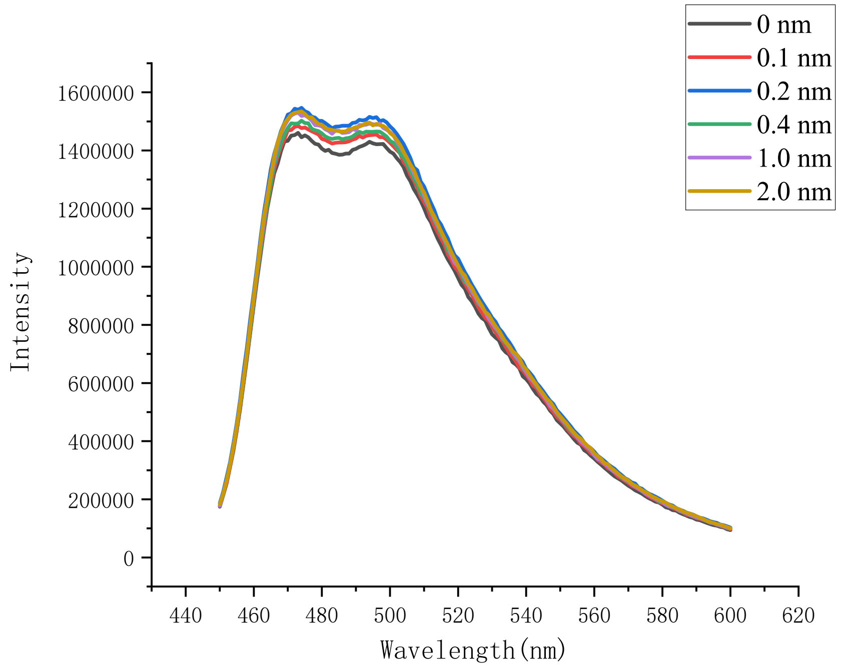

Figure S7 Fluorescence quenching of the excited **PC-II** with different concentrations of NFSI

However, as shown in **Figure S8**, the results of fluorescence quenching of the excited **PC-II** with different concentrations of DABSO suggest that the excited **PC-II** can also be quenched by DABSO. We think that after the release of sulfur dioxide, DABSO was converted to DABCO, so that the tertiary amine compounds can quench the excited **PC-II** photocatalyst. Therefore, as shown in **Figure S10**, we investigated the fluorescence quenching of excited **PC-II** with different concentrations of DABCO.

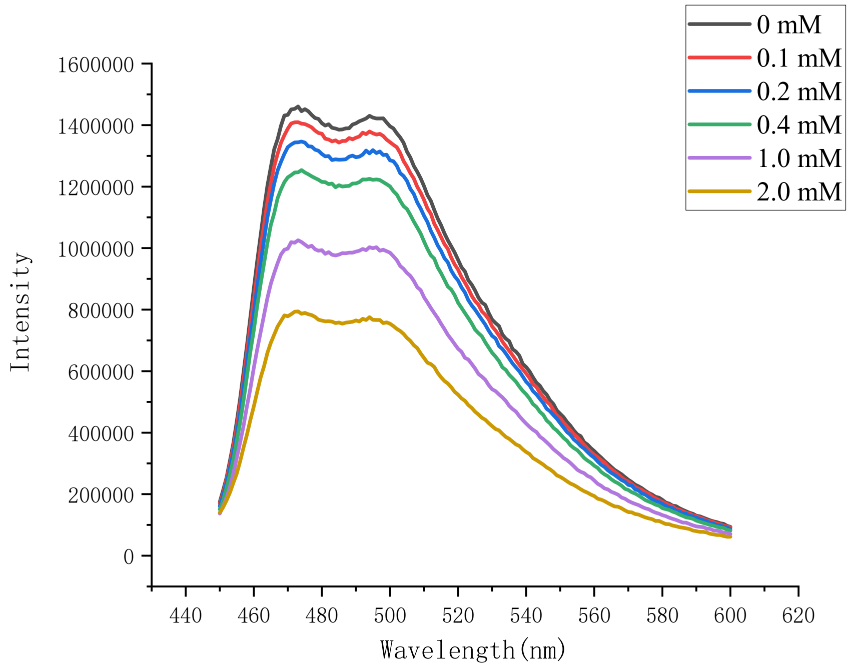

Figure S8 Fluorescence quenching of the excited **PC-II** with different concentrations of DABSO

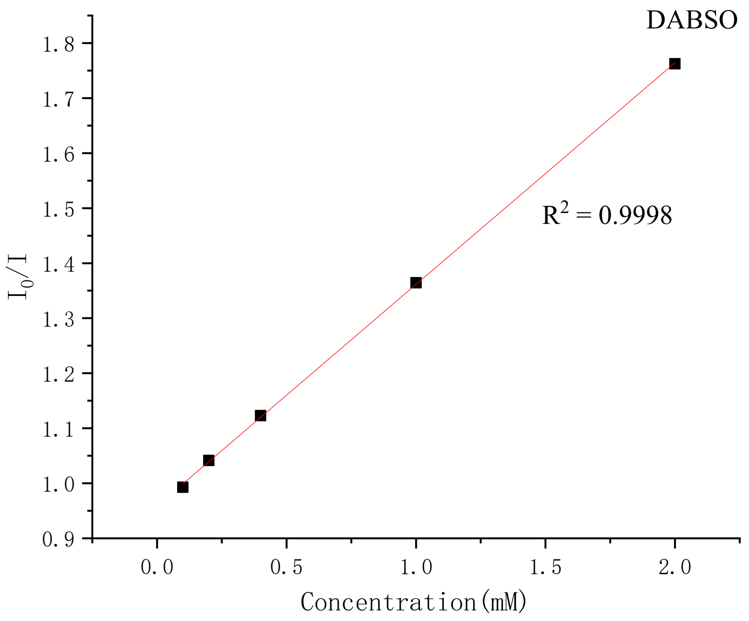

Figure S9 Stern-Volmer emission quenching studies of the excited **PC-II** with DABSO

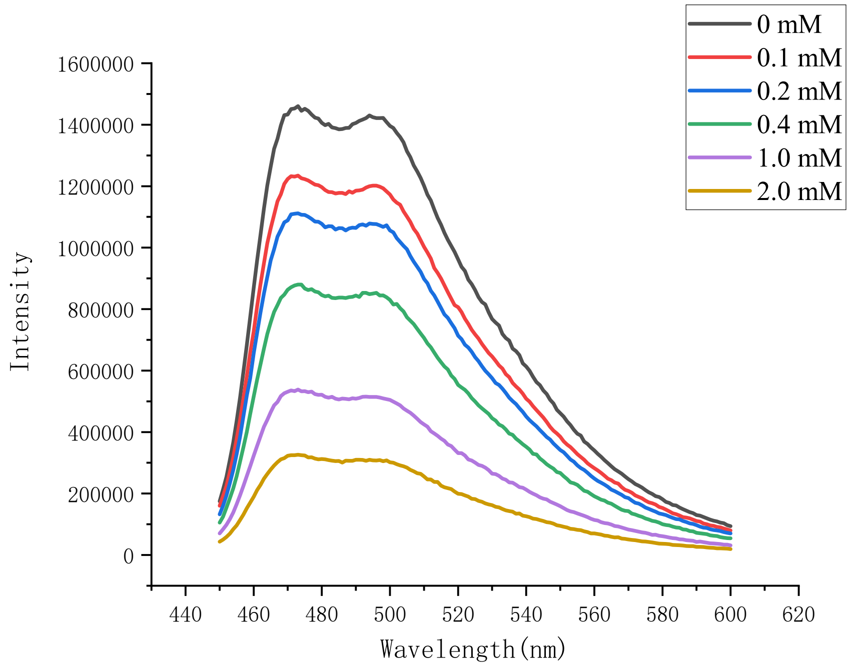

Figure S10 Fluorescence quenching of the excited **PC-II** with different concentrations of DABCO

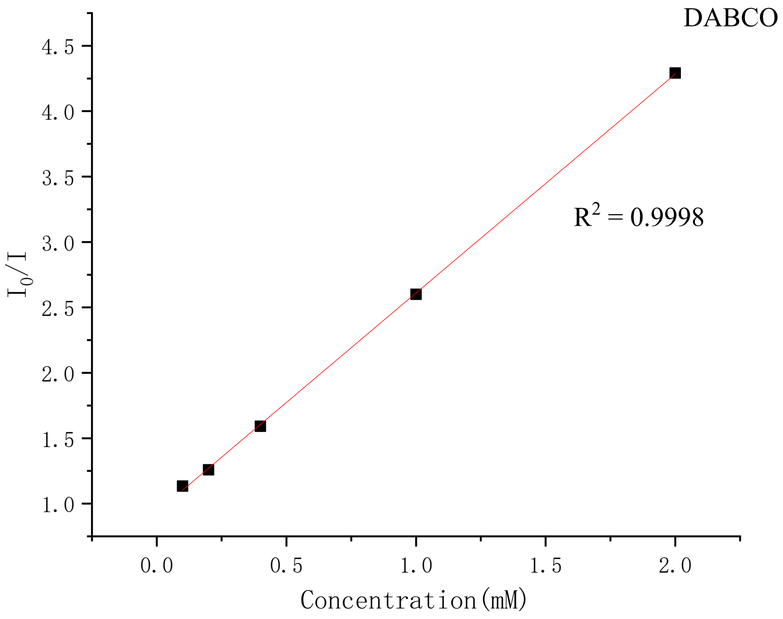

Figure S11 Stern-Volmer emission quenching studies of **PC-I**I by DABCO

9. Large-scale Experiment

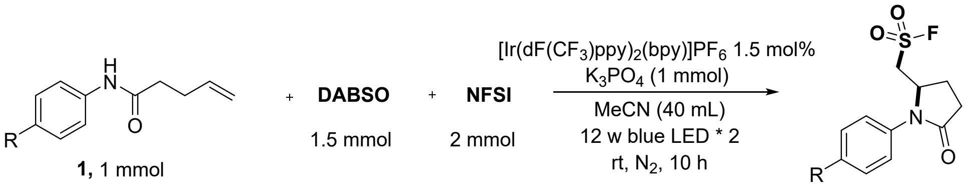

2a R = H 58%
2d R = Br 68%

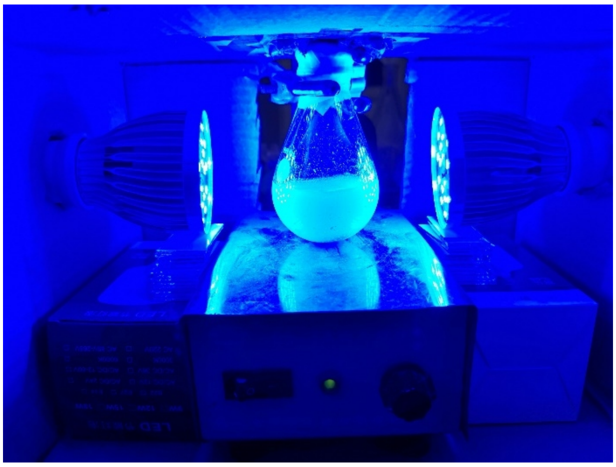

To a 100 mL round-bottom flask equipped with a magnetic stirring bar, amide **1** (1 mmol, 1.0 eq.), DABSO (361 mg, 1.5 mmol, 1.5 eq.), NFSI (631 mg, 2 mmol, 2.0 eq.), $[Ir(dF(CF_3)ppy)_2(bpy)]PF_6$ (15 mg, 1.5 mol%), K_3PO_4 (213 mg, 1 mmol, 1.0 eq.), and MeCN (4.0 mL), were added successively under N_2 atmosphere. The reaction was carried out under the illumination of two 12 W blue lamps and the heat from light was blown away by fan. After stirring at room temperature for 10 h, the reaction mixture was filtered through a pad of celite, eluted with ethyl acetate, concentrated, and purified by flash column chromatography (eluent: petroleum ether/ ethyl acetate 5:1 to 3:1) on silica gel to give the desired product **2**.

10. Characterization of Compounds 1d, 1e, 1l-1r, 1t-1ad, 1af-1ao, 2a-2ao, D1-D14, S5

Characterization of compounds **1a**, **1b**, **1c**, **1f**, **1g**, **1h**, **1i**, **1j**, **1k**, **1s** have been reported in the literature.[13] Characterization of compounds **1ae** have been reported in the literature.[14]

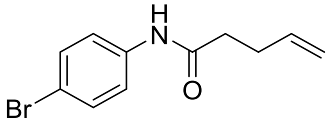

N-(4-bromophenyl)pent-4-enamide (1d)

Compound **1d** was prepared according to **Method A**.
^{1}H NMR (400 MHz, CDCl3) δ 7.55 (s, 1H), 7.39 (s, 4H), 5.85 (ddd, J = 10.3, 7.8, 4.1 Hz, 1H), 5.20 – 4.97 (m, 2H), 2.45 (d, J = 8.2 Hz, 4H).^{13}C NMR (101 MHz, CDCl3) δ 170.9, 137.0, 136.7, 132.0, 121.6, 116.9, 116.1, 36.8, 29.4. **HRMS (ESI):** m/z calculated for [M] ($C_{11}H_{12}NOBr$) from $[M-H]^+$ is 252.0029, found 252.0022.

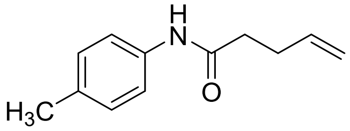

N-(p-tolyl)pent-4-enamide (1e)

Compound **1e** was prepared according to **Method A**.
^{1}H NMR (400 MHz, $CDCl_3$) δ 7.58 (s, 1H), 7.38 (d, *J* = 8.3 Hz, 2H), 7.09 (d, *J* = 8.2 Hz, 2H), 5.86 (dq, *J* = 10.5, 6.1 Hz, 1H), 5.07 (dd, *J* = 26.4, 13.5 Hz, 2H), 2.53 – 2.37 (m, 4H), 2.30 (s, 3H). ^{13}C NMR (101 MHz, $CDCl_3$) δ 170.7, 137.0, 135.4, 133.9, 129.5, 120.2, 115.8, 36.7, 29.6, 20.9. **HRMS (ESI):** *m/z* calculated for [M] ($C_{12}H_{15}NO$) from $[M+Na]^+$ is 212.1046, found 212.1031.

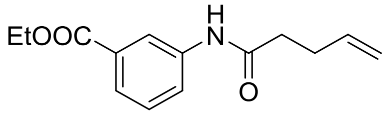

ethyl 3-(pent-4-enamido)benzoate (1l)

Compound **1l** was prepared according to the **Method A**.
1H NMR (400 MHz, $CDCl_3$) δ 8.01 (s, 1H), 7.93 (d, J = 8.1 Hz, 1H), 7.83 (s, 1H), 7.76 (d, J = 7.8 Hz, 1H), 7.36 (t, J = 7.9 Hz, 1H), 6.12 – 5.63 (m, 1H), 5.31 – 4.79 (m, 2H), 4.35 (q, J = 7.1 Hz, 2H), 2.47 (d, J = 2.7 Hz, 4H), 1.36 (t, J = 7.1 Hz, 3H). 13C NMR (101 MHz, $CDCl_3$) δ 171.1, 166.4, 138.2, 136.8, 131.2, 129.2, 125.3, 124.5, 120.7, 116.0, 61.3, 36.7, 29.4, 14.3. **HRMS (ESI):** m/z calculated for [M] ($C_{14}H_{17}NO_3$) from $[M+Na]^+$ is 270.1101, found 270.1090.

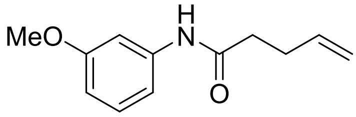

N-(3-methoxyphenyl)pent-4-enamide (1m)

Compound **1m** was prepared according to **Method A**.
^{1}H NMR (400 MHz, $CDCl_3$) δ 7.77 (s, 1H), 7.30 (s, 1H), 7.17 (t, J = 8.1 Hz, 1H), 6.98 (d, J = 7.9 Hz, 1H), 6.70 – 6.59 (m, 1H), 5.84 (ddd, J = 15.9, 8.6, 4.9 Hz, 1H), 5.05 (dd, J = 25.6, 13.7 Hz, 2H), 3.75 (s, 3H), 2.44 (s, 4H). ^{13}C NMR (101 MHz, $CDCl_3$) δ 171.0, 160.1, 139.2, 136.9, 129.6, 115.9, 112.1, 110.1, 105.7, 55.3, 36.8, 29.5. **HRMS (ESI):** m/z calculated for [M] ($C_{12}H_{15}NO_2$) from $[M+Na]^+$ is 228.0995, found 228.0980.

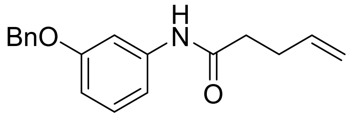

N-(3-(benzyloxy)phenyl)pent-4-enamide (1n)

Compound **1n** was prepared according to **Method A**.
^{1}H NMR (400 MHz, CDCl3) δ 7.37 (ddd, J = 23.2, 13.1, 7.0 Hz, 7H), 7.19 (t, J = 8.1 Hz, 1H), 6.98 (d, J = 7.7 Hz, 1H), 6.73 (d, J = 7.7 Hz, 1H), 5.87 (ddd, J = 16.5, 10.4, 6.0 Hz, 1H), 5.28 – 4.86 (m, 4H), 2.46 (dd, J = 10.5, 5.1 Hz, 4H).^{13}C NMR (101 MHz, CDCl3) δ 170.7, 159.4, 139.2, 136.9 (d, J = 3.3 Hz), 129.8, 128.6, 128.0, 127.6, 116.0, 112.3, 111.1, 106.5, 70.0, 36.9, 29.5. **HRMS (ESI):** m/z calculated for [M] ($C_{18}H_{19}NO_2$) from $[M+Na]^+$ is 304.1308, found 304.1295.

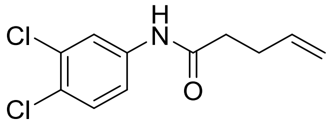

N-(3,4-dichlorophenyl)pent-4-enamide (1o)

Compound **1o** was prepared according to **Method A**.
^{1}H NMR (400 MHz, $CDCl_3$) δ 7.74 (d, J = 1.9 Hz, 1H), 7.53 (s, 1H), 7.38 – 7.27 (m, 2H), 5.97 – 5.77 (m, 1H), 5.25 – 4.99 (m, 2H), 2.46 (d, J = 2.7 Hz, 4H). ^{13}C NMR (101 MHz, $CDCl_3$) δ 170.9, 137.3, 136.6, 132.8, 130.5, 127.5, 121.7, 119.2, 116.3, 36.7, 29.3. **HRMS (ESI):** m/z calculated for [M] ($C_{11}H_{11}Cl_2NO$) from $[M+Na]^+$ is 266.0110, found 266.0093.

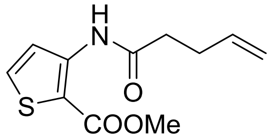

methyl 3-(pent-4-enamido)thiophene-2-carboxylate (1p)

Compound **1p** was prepared according to **Method A**.
^{1}H NMR (400 MHz, $CDCl_3$) δ 10.17 (s, 1H), 8.12 (d, J = 5.4 Hz, 1H), 7.45 (d, J = 5.4 Hz, 1H), 6.17 – 5.70 (m, 1H), 5.06 (dd, J = 30.3, 13.7 Hz, 2H), 3.88 (s, 3H), 2.99 – 2.29 (m, 4H). ^{13}C NMR (101 MHz, $CDCl_3$) δ 170.0, 164.9, 144.9, 136.5, 131.8, 122.4, 115.9, 109.9, 52.0, 36.8, 29.2. **HRMS (ESI):** m/z calculated for [M] ($C_{11}H_{13}NO_3S$) from $[M+Na]^+$ is 262.0508, found 262.0505.

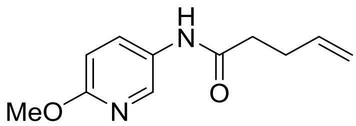

N-(6-methoxypyridin-3-yl)pent-4-enamide (1q)

Compound **1q** was prepared according to **Method A**.
^{1}H NMR (400 MHz, $CDCl_3$) δ 8.12 (d, J = 2.6 Hz, 1H), 7.83 (dd, J = 8.9, 2.7 Hz, 2H), 6.67 (d, J = 8.9 Hz, 1H), 6.18 – 5.57 (m, 1H), 5.04 (ddd, J = 13.7, 11.6, 1.5 Hz, 2H), 3.87 (s, 3H), 2.43 (d, J = 2.5 Hz, 4H). ^{13}C NMR (101 MHz, $CDCl_3$) δ 171.2, 161.0, 138.8, 136.7, 132.6, 128.6, 115.9, 110.4, 53.5, 36.2, 29.4. **HRMS (ESI):** *m/z* calculated for [M] ($C_{11}H_{14}N_2O_2$) from $[M+H]^+$ is 207.1128, found 207.1112.

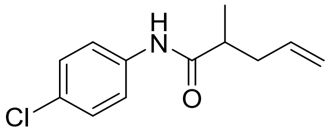

N-(4-chlorophenyl)-2-methylpent-4-enamide (1r)

Compound **1r** was prepared according to **Method A**.
^{1}H NMR (400 MHz, $CDCl_3$) δ 7.65 – 7.36 (m, 3H), 7.25 (d, J = 9.0 Hz, 2H), 5.78 (ddd, J = 13.8, 10.1, 6.9 Hz, 1H), 5.25 – 4.93 (m, 2H), 2.44 (ddd, J = 14.0, 13.2, 6.9 Hz, 2H), 2.23 (dd, J = 13.5, 6.3 Hz, 1H), 1.23 (d, J = 6.7 Hz, 3H). ^{13}C NMR (101 MHz, $CDCl_3$) δ 174.4, 136.5, 135.5, 129.3, 129.0, 121.4, 117.4, 42.2, 38.4, 17.5. **HRMS (ESI):** m/z calculated for [M] ($C_{12}H_{14}ClNO$) from $[M+H]^+$ is 224.0837, found 224.0830.

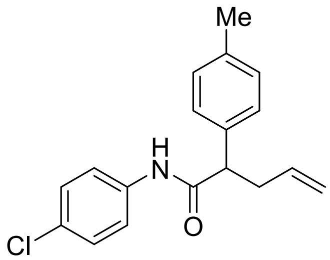

N-(4-chlorophenyl)-2-(p-tolyl)pent-4-enamide (1t)

Compound **1t** was prepared according to **Method A**, starting from **S2**.
^{1}H NMR (400 MHz, $CDCl_3$) δ 7.37 (d, J = 8.8 Hz, 2H), 7.21 (ddd, J = 17.6, 10.7, 5.1 Hz, 7H), 5.74 (ddt, J = 17.1, 10.1, 6.9 Hz, 1H), 5.04 (ddd, J = 13.6, 11.2, 1.2 Hz, 2H), 3.54 (t, J = 7.6 Hz, 1H), 2.97 (dt, J = 14.2, 7.0 Hz, 1H), 2.57 (dt, J = 14.6, 7.4 Hz, 1H), 2.35 (s, 3H). ^{13}C NMR (101 MHz, $CDCl_3$) δ 171.3, 137.5, 136.3, 135.7, 135.6, 129.8, 129.2, 128.9, 127.9, 121.0, 117.0, 53.6, 37.3, 21.1. **HRMS (ESI):** *m/z* calculated for [M] ($C_{18}H_{18}ClNO$) from $[M+Na]^+$ is 322.0969, found 322.0985.

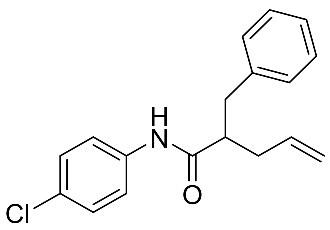

2-benzyl-N-(4-chlorophenyl)pent-4-enamide (1u)

Compound **1u** was prepared according to **Method A**, starting from **S1**.
^{1}H NMR (400 MHz, CDCl$_3$) δ 7.32 – 7.14 (m, 9H), 6.91 (s, 1H), 5.90 – 5.76 (m, 1H), 5.22 – 5.04 (m, 2H), 2.97 (dd, J = 13.5, 9.1 Hz, 1H), 2.86 (dd, J = 13.5, 5.0 Hz, 1H), 2.61 – 2.45 (m, 2H), 2.40 – 2.27 (m, 1H). ^{13}C NMR (101 MHz, CDCl$_3$) δ 172.8, 139.5, 136.0, 135.4, 129.4, 129.0, 128.9, 128.7, 126.7, 121.5, 117.6, 50.9, 38.9, 36.8. **HRMS (ESI):** *m/z* calculated for [M] ($C_{18}H_{18}ClNO$) from $[M+Na]^+$ is 322.0969, found 322.0985.

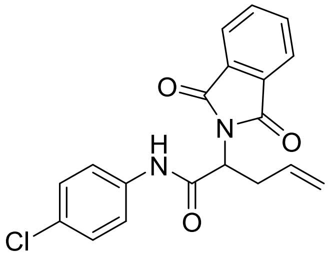

N-(4-chlorophenyl)-2-(1,3-dioxoisoindolin-2-yl)pent-4-enamide (1v)

Compound **1v** was prepared according to **Method A**.
^{1}H NMR (400 MHz, CDCl$_3$) δ 8.49 (s, 1H), 7.89 – 7.81 (m, 2H), 7.79 – 7.71 (m, 2H), 7.44 (d, J = 8.7 Hz, 2H), 7.23 (d, J = 8.8 Hz, 2H), 6.03 – 5.59 (m, 1H), 5.31 – 4.76 (m, 3H), 3.20 – 2.91 (m, 2H). ^{13}C NMR (101 MHz, CDCl$_3$) δ 168.3, 166.7, 136.0, 134.5, 133.0, 131.4, 129.6, 128.9, 123.7, 121.3, 119.6, 55.3, 33.7. **HRMS (ESI):** *m/z* calculated for [M] ($C_{19}H_{15}ClN_2O_3$) from $[M+H]^+$ is 355.0844, found 355.0853.

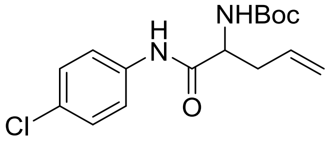

tert-butyl (1-((4-chlorophenyl)amino)-1-oxopent-4-en-2-yl)carbamate (1w)

Compound **1w** was prepared according to **Method A**.
^{1}H NMR (400 MHz, CDCl$_3$) δ 8.91 (s, 1H), 7.37 (d, J = 8.1 Hz, 2H), 7.15 (d, J = 7.8 Hz, 2H), 5.80 (td, J = 16.9, 7.2 Hz, 1H), 5.35 (s, 1H), 5.16 (t, J = 12.9 Hz, 2H), 4.37 (s, 1H), 2.85 – 2.39 (m, 2H), 1.43 (s, 9H). ^{13}C NMR (101 MHz, CDCl$_3$) δ 170.3, 156.4, 136.4, 132.9, 129.2, 128.9, 121.1, 119.3, 80.9, 54.7, 36.5, 28.4. **HRMS (ESI):** m/z calculated for [M] ($C_{16}H_{21}ClN_2O_3$) from $[M+Na]^+$ is 347.1133, found 347.1145.

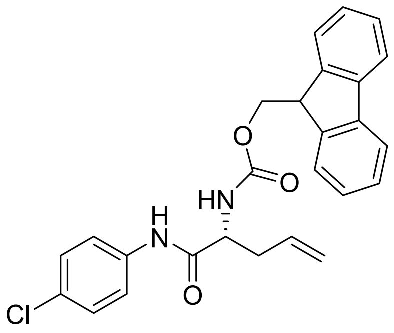

(9H-fluoren-9-yl)methyl ®-(1-((4-chlorophenyl)amino)-1-oxopent-4-en-2-yl)carbamate (1x)

Compound **1x** was prepared according to **Method A**.
^{1}H NMR (400 MHz, DMSO-*d$_6$*) δ 10.21 (s, 1H), 7.88 (d, J = 7.4 Hz, 2H), 7.73 (d, J = 7.5 Hz, 3H), 7.64 (d, J = 8.6 Hz, 2H), 7.47 – 7.28 (m, 6H), 5.90 – 5.72 (m, 1H), 5.10 (dd, J = 30.6, 13.6 Hz, 2H), 4.38 – 4.13 (m, 4H), 2.48 – 2.33 (m, 2H). ^{13}C NMR (101 MHz, DMSO-*d$_6$*) δ 170.5, 156.0, 143.8, 143.7, 140.7, 137.7, 134.0, 128.6, 127.6, 127.0, 125.3, 120.8, 120.1, 117.7, 65.7, 55.0, 46.6, 36.0. **HRMS (ESI):** m/z calculated for [M] ($C_{26}H_{23}ClN_2O_3$) from $[M+Na]^+$ is 469.1289, found 469.1320.

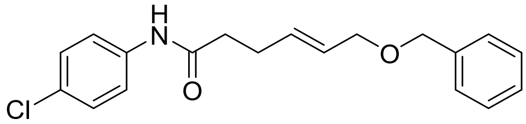

(E)-6-(benzyloxy)-N-(4-chlorophenyl)hex-4-enamide (1y)

Compound **1y** was prepared according to **Method C**.
^{1}H NMR (400 MHz, $CDCl_3$) δ 7.69 (d, J = 25.3 Hz, 1H), 7.43 (dd, J = 14.1, 8.8 Hz, 2H), 7.37 – 7.19 (m, 7H), 5.87 – 5.51 (m, 2H), 4.50 (d, J = 6.9 Hz, 2H), 4.04 (dd, J = 50.0, 6.0 Hz, 2H), 2.53 – 2.35 (m, 4H). ^{13}C NMR (101 MHz, $CDCl_3$) δ 170.7, 138.0, 136.5, 131.9, 129.2, 128.9, 128.5, 128.0, 127.9, 127.7, 121.3, 72.6, 65.7, 37.1, 23.5. **HRMS (ESI):** m/z calculated for [M] ($C_{19}H_{20}ClNO_2$) from $[M+Na]^+$ is 352.1075, found 352.1093.

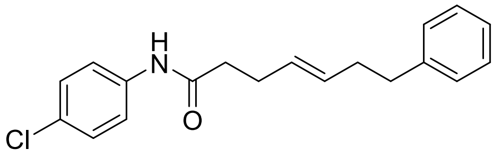

N-(4-chlorophenyl)cyclohex-3-ene-1-carboxamide (1z)

Compound **1z** was prepared according to **Method C**.
^{1}H NMR (400 MHz, $CDCl_3$) δ 7.42 (t, J = 12.9 Hz, 2H), 7.33 – 7.13 (m, 8H), 5.68 – 5.30 (m, 2H), 2.82 – 2.59 (m, 2H), 2.50 – 2.08 (m, 6H). ^{13}C NMR (101 MHz, $CDCl_3$) δ 170.9, 142.0, 136.5, 130.7, 129.0, 128.8, 128.5, 128.4, 128.3, 125.9, 121.1, 37.5, 35.8, 29.2, 23.3. **HRMS (ESI):** m/z calculated for [M] ($C_{19}H_{20}ClNO$) from $[M+Na]^+$ is 336.1126, found 336.1127.

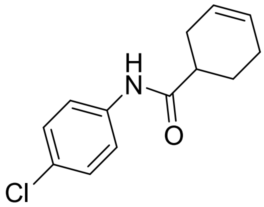

N-(4-chlorophenyl)cyclohex-3-ene-1-carboxamide (1aa)

Compound **1aa** was prepared according to **Method A**.
^{1}H NMR (400 MHz, DMSO-d_6) δ 10.02 (s, 1H), 7.64 (d, J = 8.8 Hz, 2H), 7.49 – 7.19 (m, 2H), 6.30 – 5.11 (m, 2H), 2.61 – 2.51 (m, 1H), 2.30 – 1.98 (m, 4H), 1.88 (d, J = 12.1 Hz, 1H), 1.56 (dt, J = 12.3, 10.1 Hz, 1H). ^{13}C NMR (101 MHz, DMSO-d_6) δ 165.9, 131.9, 131.6, 124.5, 124.2, 116.3, 111.3, 32.0, 28.3, 24.6, 23.8. **HRMS (ESI):** m/z calculated for [M] ($C_{13}H_{14}ClNO$) from $[M+Na]^+$ is 258.0656, found 258.0637.

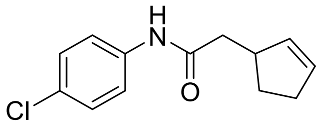

N-(4-chlorophenyl)-2-(cyclopent-2-en-1-yl)acetamide (1ab)

Compound **1ab** was prepared according to **Method A**.
^{1}H NMR (400 MHz, DMSO-d_6) δ 10.01 (s, 1H), 7.62 (d, J = 8.8 Hz, 2H), 7.33 (d, J = 8.8 Hz, 2H), 5.73 (ddd, J = 22.7, 5.6, 2.2 Hz, 2H), 3.18 – 2.83 (m, 1H), 2.44 – 2.18 (m, 4H), 2.11 – 1.94 (m, 1H), 1.46 (ddt, J = 12.6, 9.0, 6.2 Hz, 1H). ^{13}C NMR (101 MHz, DMSO-d_6) δ 170.5, 138.1, 134.3, 130.7, 128.5, 126.5, 120.5, 42.4, 42.1, 31.4, 29.0. **HRMS (ESI):** m/z calculated for [M] ($C1_3H_{14}ClNO$) from $[M+Na]^+$ is 258.0656, found 258.0638.

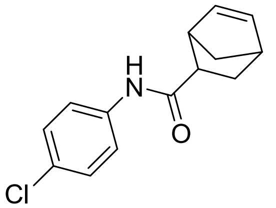

2-methoxybenzene-1-sulfonyl fluoride (1ac)

Compound **1ac** was prepared according to **Method B**.

^{1}H NMR (400 MHz, DMSO-d_6) δ 9.86 (s, 1H), 7.59 (d, J = 8.8 Hz, 2H), 7.30 (d, J = 8.8 Hz, 2H), 6.15 (dd, J = 5.5, 3.0 Hz, 1H), 5.83 (dd, J = 5.5, 2.7 Hz, 1H), 3.27 (s, 1H), 3.09 – 2.97 (m, 1H), 2.86 (s, 1H), 1.80 (ddd, J = 12.6, 9.3, 3.7 Hz, 1H), 1.48 – 1.26 (m, 3H). ^{13}C NMR (101 MHz, DMSO-d_6) δ 172.0, 138.4, 137.2, 131.6, 128.4, 126.1, 120.5, 49.5, 46.1, 44.2, 42.2, 28.2. **HRMS (ESI):** m/z calculated for [M] ($C_{14}H_{14}ClNO$) from $[M+Na]^+$ is 270.0656, found 270.0641.

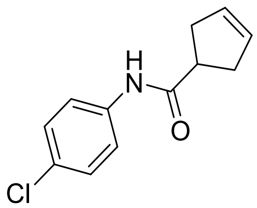

N-(4-chlorophenyl)cyclopent-3-ene-1-carboxamide (1ad)

Compound **1ad** was prepared according to **Method A**.
^{1}H NMR (400 MHz, DMSO-d_6) δ 10.05 (s, 1H), 7.64 (d, J = 8.9 Hz, 2H), 7.34 (d, J = 8.9 Hz, 2H), 5.66 (s, 2H), 3.26 – 3.09 (m, 1H), 2.67 – 2.51 (m, 4H). ^{13}C NMR (101 MHz, DMSO-d_6) δ 174.0, 138.3, 129.0, 128.5, 126.5, 120.6, 42.9, 36.6. **HRMS (ESI):** m/z calculated for [M] ($C_{12}H_{12}ClNO$) from $[M+Na]^+$ is 244.0500, found 244.0492.

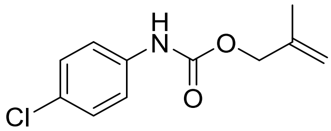

2-methylallyl (4-chlorophenyl)carbamate (1af)

Compound **1af** was prepared according to **Method B**.
^{1}H NMR (400 MHz, $CDCl_3$) δ 7.34 (d, J = 8.6 Hz, 2H), 7.26 (d, J = 8.9 Hz, 2H), 6.77 (s, 1H), 4.98 (d, J = 24.5 Hz, 2H), 4.58 (s, 2H), 1.78 (s, 3H). ^{13}C NMR (101 MHz, $CDCl_3$) δ 153.3, 140.1, 136.5, 129.1, 128.6, 120.0, 113.1, 68.7, 19.5. **HRMS (ESI):** m/z calculated for [M] ($C_{11}H_{12}ClNO_2$) from $[M+Na]^+$ is 248.0449, found 248.0435.

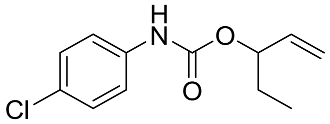

pent-1-en-3-yl (4-chlorophenyl)carbamate (1ag)

Compound **1ag** was prepared according to **Method B**.
^{1}H NMR (400 MHz, $CDCl_3$) δ 7.34 (d, J = 8.7 Hz, 2H), 7.25 (d, J = 8.9 Hz, 2H), 6.71 (s, 1H), 5.81 (ddd, J = 17.1, 10.5, 6.5 Hz, 1H), 5.26 (ddt, J = 31.9, 10.5, 1.2 Hz, 2H), 5.16 (q, J = 6.5 Hz, 1H), 1.78 – 1.63 (m, 2H), 0.95 (t, J = 7.4 Hz, 3H). ^{13}C NMR (101 MHz, $CDCl_3$) δ 153.0, 136.7, 136.4, 129.1, 128.4, 119.9, 117.1, 27.4, 9.5. **HRMS (ESI):** m/z calculated for [M] ($C_{12}H_{14}ClNO_2$) from [M+Na]+ is 262.0605, found 262.0588.

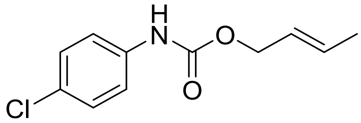

(E)-but-2-en-1-yl (4-chlorophenyl)carbamate (1ah)

Compound **1ah** was prepared according to **Method B**.
^{1}H NMR (400 MHz, $CDCl_3$) δ 7.34 (d, J = 8.7 Hz, 2H), 7.27 (d, J = 8.8 Hz, 2H), 6.68 (s, 1H), 5.85 (dq, J = 13.0, 6.5 Hz, 1H), 5.72 – 5.56 (m, 1H), 4.60 (d, J = 6.5 Hz, 2H), 1.82 – 1.70 (m, 3H). ^{13}C NMR (101 MHz, $CDCl_3$) δ 153.4, 136.6, 131.9, 129.1, 128.5, 125.2, 120.0, 66.2, 17.9. **HRMS (ESI):** m/z calculated for [M] ($C_{11}H_{12}ClNO_2$) from $[M+Na]^+$ is 248.0449, found 248.0443.

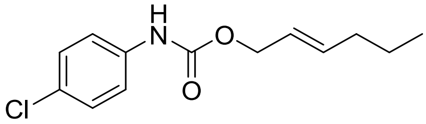

(E)-hex-2-en-1-yl (4-chlorophenyl)carbamate (1ai)

Compound **1ai** was prepared according to **Method B**.
^{1}H NMR (400 MHz, $CDCl_3$) δ 7.34 (d, J = 8.8 Hz, 2H), 7.29 – 7.25 (m, 2H), 6.68 (s, 2H), 5.92 – 5.75 (m, 1H), 5.62 (dddd, J = 13.1, 7.9, 4.6, 3.2 Hz, 1H), 4.61 (dd, J = 6.5, 0.7 Hz, 2H), 2.06 (q, J = 7.1 Hz, 2H), 1.55 – 1.28 (m, 2H), 0.92 (t, J = 7.4 Hz, 3H). ^{13}C NMR (101 MHz, $CDCl_3$) δ 153.4, 136.9, 136.6, 129.1, 124.0, 119.9, 66.3, 34.4, 22.1, 13.7. **HRMS (ESI):** m/z calculated for [M] ($C_{13}H_{16}ClNO_2$) from $[M+Na]^+$ is 276.0762, found 276.0759.

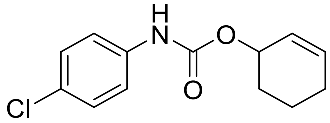

cyclohex-2-en-1-yl (4-chlorophenyl)carbamate (1aj)

Compound **1aj** was prepared according to **Method B**.
^{1}H NMR (400 MHz, $CDCl_3$) δ 7.33 (d, J = 8.7 Hz, 2H), 7.25 (d, J = 8.9 Hz, 2H), 6.65 (s, 1H), 6.10 – 5.92 (m, 1H), 5.85 – 5.69 (m, 1H), 5.27 (dd, J = 3.4, 1.5 Hz, 1H), 2.17 – 1.64 (m, 6H). ^{13}C NMR (101 MHz, $CDCl_3$) δ 153.2, 136.7, 133.1, 129.1, 128.3, 125.7, 119.9, 69.2, 28.6, 25.0, 18.8. **HRMS (ESI):** m/z calculated for [M] ($C_{13}H_{14}ClNO_2$) from $[M+Na]^+$ is 274.0605, found 274.0601.

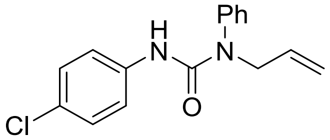

1-allyl-3-(4-chlorophenyl)-1-phenylurea (1ak)

Compound **1ak** was prepared according to **Method B**.
^{1}H NMR (400 MHz, $CDCl_3$) δ 7.52 – 7.44 (m, 2H), 7.42 – 7.36 (m, 1H), 7.33 – 7.28 (m, 2H), 7.26 – 7.15 (m, 2H), 6.18 (s, 1H), 6.04 – 5.84 (m, 1H), 5.22 – 5.03 (m, 2H), 4.33 (dt, J = 6.2, 1.3 Hz, 2H). ^{13}C NMR (101 MHz, $CDCl_3$) δ 153.8, 141.2, 137.5, 133.9, 130.4, 128.8, 128.6, 128.4, 127.9, 120.5, 117.7, 52.4. **HRMS (ESI):** m/z calculated for [M] ($C_{16}H_{15}ClN_2O$) from $[M+H]^+$ is 287.0946, found 287.0942.

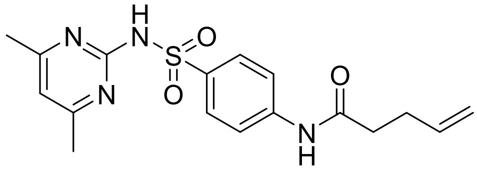

N-(4-(N-(4,6-dimethylpyrimidin-2-yl)sulfamoyl)phenyl)pent-4-enamide (1al)

Compound **1al** was prepared according to **Method A**.
^{1}H NMR (400 MHz, DMSO-*d*$_6$) δ 11.59 (s, 1H), 10.26 (s, 1H), 7.93 (d, J = 8.0 Hz, 2H), 7.74 (d, J = 8.0 Hz, 2H), 6.73 (s, 1H), 5.83 (dd, J = 16.3, 9.6 Hz, 1H), 5.01 (dd, J = 42.7, 13.5 Hz, 2H), 2.43 (d, J = 6.8 Hz, 2H), 2.33 (d, J = 5.9 Hz, 2H), 2.24 (s, 6H). ^{13}C NMR (101 MHz, DMSO-*d*$_6$) δ 171.1, 167.3, 156.2, 142.8, 137.4, 134.2, 129.3, 117.9, 115.3, 113.6, 35.5, 28.8, 22.9. **HRMS (ESI):** m/z calculated for [M] ($C_{17}H_{20}N4O_3S$) from $[M+H]^+$ is 361.1329, found 361.1348.

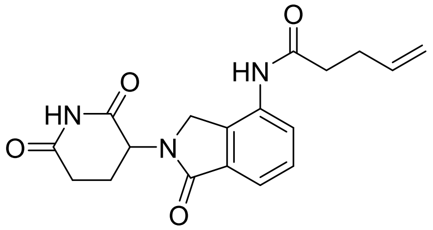

N-(2-(2,6-dioxopiperidin-3-yl)-1-oxoisoindolin-4-yl)pent-4-enamide (1am)

Compound **1am** was prepared according to **Method A**.
^{1}H NMR (400 MHz, $CDCl_3$) δ 7.77 (s, 1H), 7.30 (s, 1H), 7.17 (t, J = 8.1 Hz, 1H), 6.98 (d, J = 7.9 Hz, 1H), 6.72 – 6.57 (m, 1H), 5.84 (ddd, J = 15.9, 8.6, 4.9 Hz, 1H), 5.05 (dd, J = 25.6, 13.7 Hz, 2H), 3.75 (s, 3H), 2.44 (s, 4H). ^{13}C NMR (101 MHz, $CDCl_3$) δ 172.9, 171.1, 170.6, 167.8, 137.5, 133.8, 133.7, 132.6, 128.6, 125.2, 119.0, 115.3, 51.5, 46.5, 34.9, 31.2, 29.1, 22.6. **HRMS (ESI):** m/z calculated for [M] ($C_{18}H_{19}N_3O_4$) from $[M+H]^+$ is 342.1448, found 342.1458.

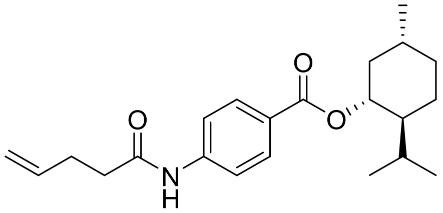

(1R,2S,5R)-2-isopropyl-5-methylcyclohexyl 4-(pent-4-enamido)benzoate (1an)

Compound **1an** was prepared according to **Method A**.

^{1}H NMR (400 MHz, $CDCl_3$) δ 8.65 (s, 1H), 7.96 (d, J = 8.7 Hz, 2H), 7.65 (d, J = 8.6 Hz, 2H), 5.80 (tt, J = 16.4, 6.2 Hz, 1H), 5.01 (ddd, J = 13.7, 11.5, 1.3 Hz, 2H), 4.88 (td, J = 10.8, 4.3 Hz, 1H), 2.46 (ddd, J = 12.5, 6.9, 3.6 Hz, 4H), 2.07 (d, J = 11.9 Hz, 1H), 2.00 – 1.83 (m, 1H), 1.80 – 1.63 (m, 2H), 1.51 (ddd, J = 19.8, 14.5, 5.7 Hz, 2H), 1.08 (dd, J = 23.3, 12.0 Hz, 2H), 0.89 (d, J = 6.8 Hz, 7H), 0.75 (d, J = 6.9 Hz, 3H). ^{13}C NMR (101 MHz, $CDCl_3$) δ 171.6, 166.0, 142.4, 136.6, 130.7, 125.9, 119.0, 115.8, 74.9, 47.2, 41.0, 36.7, 34.2, 31.4, 29.3, 26.5, 23.7, 22.0, 20.7, 16.6. **HRMS (ESI):** m/z calculated for [M] ($C_{22}H_{31}NO_3$) from $[M+Na]^+$ is 380.2196, found 380.2192.

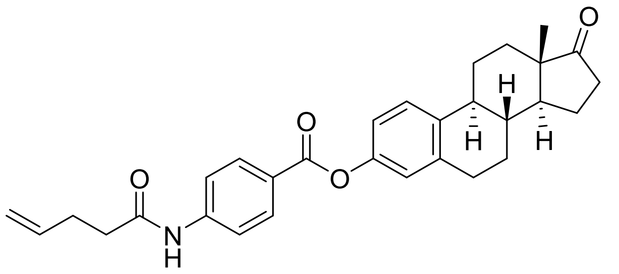

(8R,9S,13S,14S)-13-methyl-17-oxo-7,8,9,11,12,13,14,15,16,17-decahydro-6H-cyclopenta[a]phenanthren-3-yl 4-(pent-4-enamido) benzoate (1ao)

Compound **1ao** was prepared according to **Method A**.
^{1}H NMR (400 MHz, $CDCl_3$) δ 8.12 (d, J = 8.7 Hz, 2H), 7.90 (s, 1H), 7.65 (d, J = 8.7 Hz, 2H), 7.31 (d, J = 8.5 Hz, 1H), 7.07 – 6.85 (m, 2H), 6.00 – 5.75 (m, 1H), 5.22 – 4.99 (m, 2H), 3.02 – 2.83 (m, 2H), 2.62 – 1.93 (m, 12H), 1.74 – 1.39 (m, 5H), 0.92 (s, 3H). ^{13}C NMR (101 MHz, $CDCl_3$) δ 171.1, 165.2, 148.9, 142.9, 138.2, 137.5, 136.7, 131.5, 126.5, 124.7, 121.8, 118.9, 118.9, 116.1, 50.5, 48.1, 44.2, 38.0, 36.9, 36.0, 31.6, 29.5, 29.3, 26.4, 25.8, 21.6, 13.9. **HRMS (ESI):** m/z calculated for [M] ($C_{30}H_{33}NO_4$) from $[M+H]^+$ is 472.2482, found 472.2514.

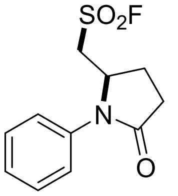

(5-oxo-1-phenylpyrrolidin-2-yl)methanesulfonyl fluoride (2a)

Compound **2a** was prepared according to **General Procedure**. The residue was purified by flash column chromatography on silica (petroleum ether/ethyl acetate 5:1) to give a white solid (15.4 mg, 60% yield).
^{1}H NMR (400 MHz, CDCl3) δ 7.44 (dt, J = 9.2, 1.9 Hz, 2H), 7.40 – 7.34 (m, 2H), 7.32 – 7.26 (m, 1H), 4.88 – 4.62 (m, 1H), 3.67 (ddd, J = 14.6, 4.5, 2.3 Hz, 1H), 3.40 (ddd, J = 14.6, 10.0, 3.0 Hz, 1H), 2.83 – 2.52 (m, 3H), 2.36 – 2.17 (m, 1H). ^{13}C NMR (101 MHz, $CDCl_3$) δ 173.5, 135.8, 129.8, 127.1, 123.8, 54.9, 53.1 (d, *J* = 14.8 Hz), 30.3, 23.8. ^{19}F NMR (376 MHz, $CDCl_3$) δ 60.51. **HRMS (ESI):** *m/z* calculated for [M] ($C_{11}H_{12}FNO_3S$) from $[M+H]^+$ is 258.0595, found 258.0603

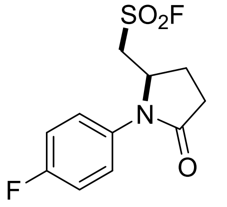

(1-(4-fluorophenyl)-5-oxopyrrolidin-2-yl)methanesulfonyl fluoride (2b)

Compound **2b** was prepared according to **General Procedure**. The residue was purified by flash column chromatography on silica (petroleum ether/ethyl acetate 5:1) to give a white solid (15.4 mg, 56% yield).
^{1}H NMR (400 MHz, $CDCl_3$) δ 7.40 – 7.28 (m, 2H), 7.20 – 7.08 (m, 2H), 4.79 – 4.60 (m, 1H), 3.64 (ddd, *J* = 14.6, 4.3, 2.4 Hz, 1H), 3.41 (ddd, *J* = 14.6, 9.8, 2.8 Hz, 1H), 2.76 – 2.54 (m, 3H), 2.34 – 2.19 (m, 1H). ^{13}C NMR (101 MHz, $CDCl_3$) δ 173.5, 161.1 (d, *J* = 247.9 Hz), 131.8 (d, *J* = 3.0 Hz), 125.9 (d, *J* = 8.5 Hz), 116.8 (d, *J* = 22.8 Hz), 55.2, 53.1 (d, *J* = 14.9 Hz), 30.1, 23.9. ^{19}F NMR (376 MHz, $CDCl_3$) δ 60.78, -113.72. **HRMS (ESI):** *m/z* calculated for [M] ($C_{11}H_{11}F_2NO_3S$) from $[M+H]^+$ is 276.0500, found 276.0495

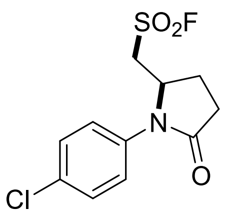

(1-(4-chlorophenyl)-5-oxopyrrolidin-2-yl)methanesulfonyl fluoride (2c)

Compound **2c** was prepared according to the **General Procedure**. The residue was purified by flash column chromatography on silica (petroleum ether/ethyl acetate 5:1) to give a white solid (22.7 mg, 78% yield).
^{1}H NMR (400 MHz, $CDCl_3$) δ 7.45 – 7.39 (m, 2H), 7.39 – 7.33 (m, 2H), 4.95 – 4.65 (m, 1H), 3.66 (ddd, *J* = 14.6, 4.3, 2.3 Hz, 1H), 3.41 (ddd, *J* = 14.6, 9.8, 2.7 Hz, 1H), 2.81 – 2.58 (m, 3H), 2.39 – 2.20 (m, 1H). ^{13}C NMR (101 MHz, $CDCl_3$) δ 173.4, 134.4, 132.5, 130.0, 124.7, 54.7, 53.0 (d, *J* = 14.9 Hz), 30.2, 23.8. ^{19}F NMR (376 MHz, $CDCl_3$) δ 60.75. **HRMS (ESI):** *m/z* calculated for [M] ($C_{11}H_{11}NO_3FSCl$) from $[M+H]^+$ is 292.0205, found 292.0201

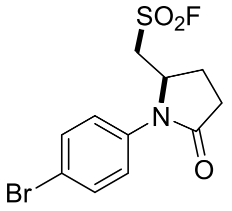

(1-(4-bromophenyl)-5-oxopyrrolidin-2-yl)methanesulfonyl fluoride (2d)

Compound **2d** was prepared according to **General Procedure**. The residue was purified by flash column chromatography on silica (petroleum ether/ethyl acetate 5:1) to give a white solid (24.1 mg, 72% yield).
^{1}H NMR (400 MHz, $CDCl_3$) δ 7.63 – 7.47 (m, 2H), 7.34 – 7.17 (m, 2H), 4.81 – 4.71 (m, 1H), 3.65 (ddd, *J* = 14.6, 4.3, 2.3 Hz, 1H), 3.42 (ddd, *J* = 14.6, 9.8, 2.7 Hz, 1H), 2.83 – 2.51 (m, 3H), 2.34 – 2.19 (m, 1H). ^{13}C NMR (126 MHz, $CDCl_3$) δ 173.3, 134.9, 132.9, 124.9, 120.3, 54.6, 53.0 (d, *J* = 15.0 Hz), 30.2, 23.7. ^{19}F NMR (376 MHz, $CDCl_3$) δ 60.72. **HRMS (ESI):** *m/z* calculated for [M] ($C_{11}H_{11}NO_3FSBr$) from $[M+H]^+$ is 335.9700, found 335.9706

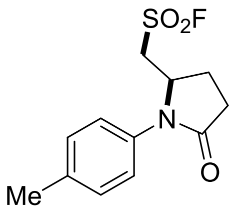

(5-oxo-1-(p-tolyl)pyrrolidin-2-yl)methanesulfonyl fluoride (2e)

Compound **2e** was prepared according to **General Procedure**. The residue was purified by flash column chromatography on silica (petroleum ether/ethyl acetate 5:1) to give a white solid (11.4 mg, 42% yield).
^{1}H NMR (400 MHz, $CDCl_3$) δ 7.28 – 7.15 (m, 4H), 4.82 – 4.62 (m, 1H), 3.66 (ddd, *J* = 14.6, 4.5, 2.4 Hz, 1H), 3.38 (ddd, *J* = 14.6, 10.0, 3.1 Hz, 1H), 2.74 – 2.56 (m, 3H), 2.35 (s, 3H), 2.28 – 2.17 (m, 1H). ^{13}C NMR (101 MHz, $CDCl_3$) δ 173.5, 137.2, 133.1, 130.4, 124.0, 55.1, 53.2 (d, *J* = 14.6 Hz), 30.2, 23.9, 21.1. ^{19}F NMR (376 MHz, $CDCl_3$) δ 60.47. **HRMS (ESI):** *m/z* calculated for [M] ($C_{12}H_{14}NO_3FS$) from $[M+H]^+$ is 272.0751, found 272.0739

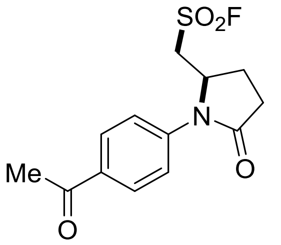

(1-(4-acetylphenyl)-5-oxopyrrolidin-2-yl)methanesulfonyl fluoride (2f)

Compound **2f** was prepared according to **General Procedure**. The residue was purified by flash column chromatography on silica (petroleum ether/ethyl acetate 3:1 to 1:1) to give a white solid (15.0 mg, 50% yield).
^{1}H NMR (400 MHz, $CDCl_3$) δ 8.03 (d, *J* = 8.6 Hz, 2H), 7.58 (d, *J* = 8.6 Hz, 2H), 4.90 (ddd, *J* = 9.8, 6.1, 4.0 Hz, 1H), 3.69 (ddd, *J* = 14.7, 4.0, 2.1 Hz, 1H), 3.47 (ddd, *J* = 14.6, 9.8, 2.6 Hz, 1H), 2.84 – 2.62 (m, 3H), 2.60 (s, 3H), 2.38 – 2.25 (m, 1H). ^{13}C NMR (126 MHz, $CDCl_3$) δ 196.8, 173.4, 140.2, 134.7, 130.0, 122.0, 54.2, 52.8 (d, *J* = 15.1 Hz), 30.4, 26.7, 23.6. ^{19}F NMR (376 MHz, $CDCl_3$) δ 60.61. **HRMS (ESI):** *m/z* calculated for [M] ($C_{13}H_{14}NO_4FS$) from $[M+H]^+$ is 322.0520, found 322.0517

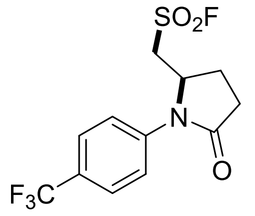

(5-oxo-1-(4-(trifluoromethyl)phenyl)pyrrolidin-2-yl)methanesulfonyl fluoride (2g)

Compound **2g** was prepared according to **General Procedure**. The residue was purified by flash column chromatography on silica (petroleum ether/ethyl acetate 5:1) to give a white solid (8.1 mg, 25% yield).
^{1}H NMR (500 MHz, $CDCl_3$) δ 7.71 (d, *J* = 8.5 Hz, 2H), 7.60 (d, *J* = 8.4 Hz, 2H), 4.88 (s, 1H), 3.68 (d, *J* = 14.7 Hz, 1H), 3.56 – 3.36 (m, 1H), 2.91 – 2.56 (m, 3H), 2.32 (ddd, *J* = 12.7, 7.2, 3.6 Hz, 1H). ^{13}C NMR (101 MHz, $CDCl_3$) δ 173.5, 139.1, 128.4 (d, *J* = 33.1 Hz), 127.0 (dd, *J* = 7.2, 3.6 Hz), 125.1, 122.6, 54.3, 52.8 (d, *J* = 15.1 Hz), 30.3, 23.7. ^{19}F NMR (376 MHz, $CDCl_3$) δ 60.70 (s), -62.56 (s). **HRMS (ESI):** *m/z* calculated for [M] ($C_{12}H_{11}NO_3F_4S$) from $[M+H]^+$ is 326.0469, found 326.0468

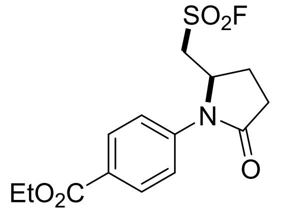

ethyl 4-(2-((fluorosulfonyl)methyl)-5-oxopyrrolidin-1-yl)benzoate (2h)

Compound **2h** was prepared according to **General Procedure**. The residue was purified by flash column chromatography on silica (petroleum ether/ethyl acetate 5:1 to 3:1) to give a white solid (18.4 mg, 56% yield).
^{1}H NMR (400 MHz, $CDCl_3$) δ 8.10 (d, *J* = 8.5 Hz, 2H), 7.54 (d, *J* = 8.6 Hz, 2H), 4.87 (d, *J* = 2.9 Hz, 1H), 4.37 (q, *J* = 7.1 Hz, 2H), 3.77 – 3.59 (m, 1H), 3.57 – 3.36 (m, 1H), 2.85 – 2.45 (m, 3H), 2.42 – 2.05 (m, 1H), 1.38 (t, *J* = 7.1 Hz, 3H). ^{13}C NMR (101 MHz, $CDCl_3$) δ 173.4, 165.7, 140.0, 131.1, 128.2, 121.9, 61.2, 54.3, 52.7 (d, *J* = 15.0 Hz), 30.4, 23.6, 14.4. ^{19}F NMR (376 MHz, $CDCl_3$) δ 60.52. **HRMS (ESI):** *m/z* calculated for [M] ($C_{14}H_{16}NO_5FS$) from $[M+H]^+$ is 352.0625, found 352.0609

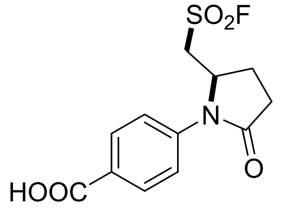

4-(2-((fluorosulfonyl)methyl)-5-oxopyrrolidin-1-yl)benzoic acid (2i)

Compound **2i** was prepared according to **General Procedure**. The residue was purified by flash column chromatography on silica (petroleum ether/ethyl acetate 3:1 to 1:1) to give a white solid (12.6 mg, 42% yield).
^{1}H NMR (400 MHz, DMSO-d_6) δ 12.90 (s, 1H), 7.98 (d, *J* = 8.7 Hz, 2H), 7.66 (d, *J* = 8.7 Hz, 2H), 5.03 (t, *J* = 7.9 Hz, 1H), 4.38 (ddd, *J* = 14.1, 8.6, 5.2 Hz, 1H), 4.12 (ddd, *J* = 15.1, 5.0, 2.5 Hz, 1H), 2.77 (dt, *J* = 15.3, 7.9 Hz, 1H), 2.59 – 2.39 (m, 2H), 2.25 – 2.07 (m, 1H). ^{13}C NMR (101 MHz, DMSO-d_6) δ 173.8, 166.8, 140.7, 130.2, 127.2, 122.0, 53.8, 51.6 (d, *J* = 11.7 Hz), 30.1, 22.7. ^{19}F NMR (376 MHz, DMSO-d_6) δ 60.51. **HRMS (ESI):** *m/z* calculated for [M] ($C_{12}H_{12}NO_5FS$) from $[M+H]^+$ is 300.0347, found 300.0345

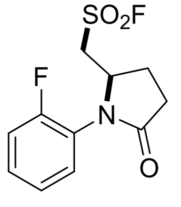

(1-(2-fluorophenyl)-5-oxopyrrolidin-2-yl)methanesulfonyl fluoride (2j)

Compound **2j** was prepared according to **General Procedure**. The residue was purified by flash column chromatography on silica (petroleum ether/ethyl acetate 5:1) to give a white solid (17.6 mg, 64% yield).
^{1}H NMR (400 MHz, $CDCl_3$) δ 7.46 – 7.36 (m, 1H), 7.36 – 7.19 (m, 3H), 4.67 (ddd, *J* = 9.6, 5.1, 2.4 Hz, 1H), 3.61 – 3.36 (m, 2H), 2.82 – 2.53 (m, 3H), 2.39 – 2.25 (m, 1H). ^{13}C NMR (101 MHz, $CDCl_3$) δ 174.0, 157.9 (d, *J* = 249.4 Hz), 130.4 (d, *J* = 6.7 Hz), 129.6, 125.3,

123.2, 117.2 (d, *J* = 20.0 Hz), 55.5, 53.5 (d, *J* = 14.8 Hz), 29.4, 24.7. ^{19}F NMR (376 MHz, $CDCl_3$) δ 60.94, -119.82. **HRMS (ESI):** *m/z* calculated for [M] ($C_{11}H_{11}NO_3F_2S$) from $[M+H]^+$ is 276.0500, found 276.0490

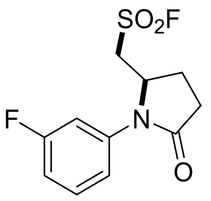

(1-(3-fluorophenyl)-5-oxopyrrolidin-2-yl)methanesulfonyl fluoride (2k)

Compound **2k** was prepared according to **General Procedure**. The residue was purified by flash column chromatography on silica (petroleum ether/ethyl acetate 5:1) to give a white solid (17.0 mg, 62% yield).
^{1}H NMR (400 MHz, $CDCl_3$) δ 7.40 (td, *J* = 8.2, 6.5 Hz, 1H), 7.31 (dt, *J* = 10.4, 2.2 Hz, 1H), 7.12 (dd, *J* = 8.1, 1.4 Hz, 1H), 6.98 (td, *J* = 8.2, 1.8 Hz, 1H), 4.78 (ddd, *J* = 9.8, 4.9, 2.5 Hz, 1H), 3.69 (ddd, *J* = 14.7, 4.3, 2.2 Hz, 1H), 3.44 (ddd, *J* = 14.6, 9.8, 2.9 Hz, 1H), 2.83 – 2.54 (m, 3H), 2.35 – 2.14 (m, 1H). ^{13}C NMR (101 MHz, $CDCl_3$) δ 173.4, 163.2 (d, *J* = 247.6 Hz), 137.5 (d, *J* = 10.1 Hz), 131.0 (d, *J* = 9.2 Hz), 118.0 (d, *J* = 3.1 Hz), 113.7 (d, *J* = 21.2 Hz), 110.9 (d, *J* = 25.0 Hz), 54.6, 52.8 (d, *J* = 14.9 Hz), 30.3, 23.6. ^{19}F NMR (376 MHz, $CDCl_3$) δ 60.57, -109.75. **HRMS (ESI):** *m/z* calculated for [M] ($C_{11}H_{11}NO_3F_2S$) from $[M+H]^+$ is 276.0500, found 276.0500

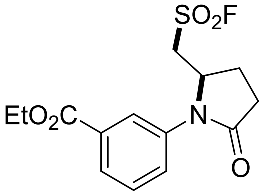

ethyl 3-(2-((fluorosulfonyl)methyl)-5-oxopyrrolidin-1-yl)benzoate (2l)

Compound **2l** was prepared according to **General Procedure**. The residue was purified by flash column chromatography on silica (petroleum ether/ethyl acetate 5:1 to 3:1) to give a white solid (20.4 mg, 62% yield).
^{1}H NMR (400 MHz, $CDCl_3$) δ 8.02 – 7.80 (m, 2H), 7.68 (ddd, *J* = 8.0, 2.1, 1.0 Hz, 1H), 7.52 (t, *J* = 7.9 Hz, 1H), 4.84 (ddd, *J* = 9.4, 5.8, 2.4 Hz, 1H), 4.49 – 4.27 (m, 2H), 3.66 (ddd, *J* = 14.7, 4.0, 2.4 Hz, 1H), 3.45 (ddd, *J* = 14.6, 9.7, 3.3 Hz, 1H), 2.87 – 2.52 (m, 3H), 2.39 – 2.20 (m, 1H), 1.39 (t, *J* = 7.1 Hz, 3H). ^{13}C NMR (101 MHz, $CDCl_3$) δ 173.5, 165.7, 136.2, 132.2, 129.9, 128.3, 128.0, 124.2, 61.5, 54.8, 53.0 (d, *J* = 14.9 Hz), 30.2, 23.8, 14.3. ^{19}F NMR (376 MHz, $CDCl_3$) δ 60.73. **HRMS (ESI):** *m/z* calculated for [M] ($C_{14}H_{16}NO_5FS$) from $[M+H]^+$ is 352.0625, found 352.0608

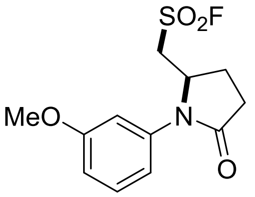

(1-(3-methoxyphenyl)-5-oxopyrrolidin-2-yl)methanesulfonyl fluoride (2m)

Compound **2m** was prepared according to **General Procedure**. The residue was purified by flash column chromatography on silica (petroleum ether/ethyl acetate 5:1 to 3:1) to give a colourless oil (10.1 mg, 35% yield).
^{1}H NMR (400 MHz, $CDCl_3$) δ 7.34 (t, *J* = 8.2 Hz, 1H), 7.01 (t, *J* = 2.2 Hz, 1H), 6.91 (dd, *J* = 7.9, 1.8 Hz, 1H), 6.82 (dd, *J* = 8.3, 2.4 Hz, 1H), 4.86 – 4.61 (m, 1H), 3.81 (s, 3H), 3.71 (ddd, *J* = 14.6, 4.5, 2.2 Hz, 1H), 3.50 – 3.32 (m, 1H), 2.79 – 2.56 (m, 3H), 2.33 – 2.17 (m, 1H). ^{13}C NMR (101 MHz, $CDCl_3$) δ 173.4, 160.7, 137.0, 130.5, 115.4, 112.7, 109.8, 55.5, 55.0, 53.1 (d, *J* = 14.7 Hz), 30.4, 23.8. ^{19}F NMR (376 MHz, $CDCl_3$) δ 60.44. **HRMS (ESI):** *m/z* calculated for [M] ($C_{12}H_{14}NO_4FS$) from $[M+H]^+$ is 310.0520, found 310.0506

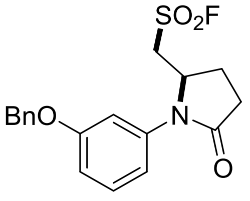

(1-(3-(benzyloxy)phenyl)-5-oxopyrrolidin-2-yl)methanesulfonyl fluoride (2n)

Compound **2n** was prepared according to **General Procedure**. The residue was purified by flash column chromatography on silica (petroleum ether/ethyl acetate 5:1 to 3:1) to give a colourless oil (12.3 mg, 34% yield).

^{1}H NMR (400 MHz, $CDCl_3$) δ 7.47 – 7.28 (m, 6H), 7.10 (t, *J* = 2.2 Hz, 1H), 6.91 (td, *J* = 8.1, 2.2 Hz, 2H), 5.12 – 5.03 (m, 2H), 4.82 – 4.58 (m, 1H), 3.69 (ddd, *J* = 14.6, 4.5, 2.3 Hz, 1H), 3.37 (ddd, *J* = 14.5, 10.0, 2.8 Hz, 1H), 2.84 – 2.53 (m, 3H), 2.35 – 2.18 (m, 1H). ^{13}C NMR (101 MHz, $CDCl_3$) δ 173.4, 159.8, 137.0, 136.5, 130.6, 128.7, 128.3, 127.6, 115.7, 113.6, 110.7, 70.3, 55.0, 53.1 (d, *J* = 14.7 Hz), 30.4, 23.8. ^{19}F NMR (376 MHz, $CDCl_3$) δ 60.61. **HRMS (ESI):** *m/z* calculated for [M] ($C_{18}H_{18}NO_4FS$) from $[M+H]^+$ is 386.0833, found 386.0816

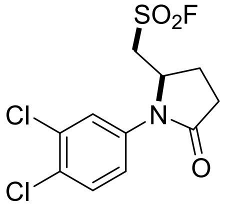

(1-(3,4-dichlorophenyl)-5-oxopyrrolidin-2-yl)methanesulfonyl fluoride (2o)

Compound **2o** was prepared according to **General Procedure**. The residue was purified by flash column chromatography on silica (petroleum ether/ethyl acetate 5:1 to 3:1) to give a white solid (20.2 mg, 62% yield).
^{1}H NMR (400 MHz, $CDCl_3$) δ 7.64 (d, *J* = 2.4 Hz, 1H), 7.51 (d, *J* = 8.7 Hz, 1H), 7.24 (dd, *J* = 8.8, 2.5 Hz, 1H), 4.86 – 4.64 (m, 1H), 3.73 – 3.57 (m, 1H), 3.45 (ddd, *J* = 14.4, 9.6, 2.4 Hz, 1H), 2.88 – 2.51 (m, 3H), 2.46 – 2.15 (m, 1H). ^{13}C NMR (126 MHz, $CDCl_3$) δ 173.3, 135.4, 133.8, 131.3, 130.7, 125.2, 122.0, 54.5, 52.8 (d, *J* = 15.1 Hz), 30.1, 23.6. ^{19}F NMR (376 MHz, $CDCl_3$) δ 60.89. **HRMS (ESI):** *m/z* calculated for [M] ($C_{11}H_{10}NO_3FSCl_2$) from $[M+H]^+$ is 325.9815, found 325.9822

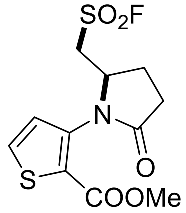

methyl 3-(2-((fluorosulfonyl)methyl)-5-oxopyrrolidin-1-yl)thiophene-2-carboxylate (2p)

Compound **2p** was prepared according to **General Procedure**. The residue was purified by flash column chromatography on silica (petroleum ether/ethyl acetate 3:1 to 1:1) to give a foamed solid (19.3 mg, 60% yield).
^{1}H NMR (400 MHz, DMSO-d_6) δ 7.94 (d, *J* = 5.3 Hz, 1H), 7.19 (d, *J* = 5.3 Hz, 1H), 4.67 (ddd, *J* = 11.2, 7.2, 3.4 Hz, 1H), 4.36 (ddd, *J* = 14.7, 8.8, 5.8 Hz, 1H), 4.03 (ddd, *J* = 14.9, 5.4, 3.3 Hz, 1H), 3.78 (s, 3H), 2.70 – 2.56 (m, 1H), 2.53 – 2.31 (m, 2H), 2.17 (ddd, *J* = 15.6, 8.3, 3.6 Hz, 1H). ^{13}C NMR (126 MHz, DMSO-d_6) δ 173.6, 160.7, 139.1, 131.6, 128.4, 125.6, 55.6, 52.5 (d, *J* = 11.9 Hz), 52.1, 28.9, 24.1. ^{19}F NMR (376 MHz, DMSO-d_6) δ 59.85. **HRMS (ESI):** *m/z* calculated for [M] ($C_{11}H_{12}NO_5FS_2$) from $[M+H]^+$ is 344.0033, found 344.0023

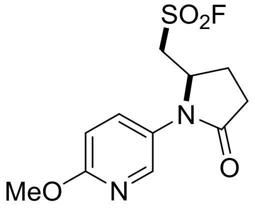

(1-(6-methoxypyridin-3-yl)-5-oxopyrrolidin-2-yl)methanesulfonyl fluoride (2q)

Compound **2q** was prepared according to **General Procedure**. The residue was purified by flash column chromatography on silica (petroleum ether/ethyl acetate 3:1 to 1:1) to give a white solid (11.5 mg, 40% yield).
^{1}H NMR (400 MHz, DMSO-d_6) δ 8.18 (d, *J* = 2.6 Hz, 1H), 7.77 (dd, *J* = 8.8, 2.7 Hz, 1H), 6.89 (d, *J* = 8.8 Hz, 1H), 4.79 (td, *J* = 7.9, 3.8 Hz, 1H), 4.41 – 4.24 (m, 1H), 4.13 (ddd, *J* = 15.0, 5.1, 3.1 Hz, 1H), 3.86 (s, 3H), 2.73 – 2.58 (m, 1H), 2.43 (ddd, *J* = 13.6, 11.1, 6.6 Hz, 2H), 2.25 – 2.02 (m, 1H). ^{13}C NMR (101 MHz, DMSO-d_6) δ 173.8, 161.5, 143.3, 136.2, 127.3, 110.5, 54.7, 53.3, 52.1 (d, *J* = 11.7 Hz), 29.4, 23.2. ^{19}F NMR (376 MHz, DMSO-d_6) δ 60.66. **HRMS (ESI):** *m/z* calculated for [M] ($C_{11}H_{13}N_2O_4FS$) from $[M+H]^+$ is 289.0653, found 289.0644

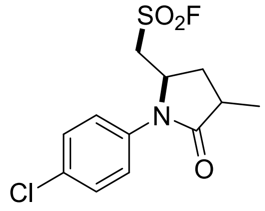

(1-(4-chlorophenyl)-4-methyl-5-oxopyrrolidin-2-yl)methanesulfonyl fluoride (2r)

Compound **2r** was prepared according to **General Procedure**. The residue was purified by flash column chromatography on silica (petroleum ether/ethyl acetate 5:1) to give a white solid (21.4 mg, 70% yield, *dr* = 2.5:1).
^{1}H NMR (500 MHz, $CDCl_3$) δ 7.42 (dd, *J* = 21.0, 8.9 Hz, 4H), 4.73 (dd, *J* = 9.8, 8.1 Hz, 1H), 3.60 (dd, *J* = 12.0, 2.7 Hz, 1H), 3.54 – 3.32 (m, 1H), 2.89 – 2.70 (m, 1H), 2.57 (dd, *J* = 12.8, 9.2 Hz, 1H), 2.27 – 2.09 (m, 1H), 1.32 (d, *J* = 7.0 Hz, 3H). ^{13}C NMR (101 MHz, $CDCl_3$) δ 175.7, 134.8, 131.9, 129.9, 123.8, 52.8, 52.1 (d, *J* = 14.6 Hz), 35.6, 31.9, 15.9. ^{19}F NMR (376 MHz, $CDCl_3$) δ 60.21. **HRMS (ESI):** *m/z* calculated for [M] ($C_{12}H_{13}NO_3FSCl$) from $[M+H]^+$ is 306.0361, found 306.0356

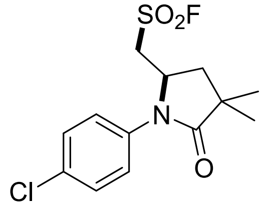

(1-(4-chlorophenyl)-4,4-dimethyl-5-oxopyrrolidin-2-yl)methanesulfonyl fluoride (2s)

Compound **2s** was prepared according to **General Procedure**. The residue was purified by flash column chromatography on silica (petroleum ether/ethyl acetate 5:1) to give a white solid (23.7 mg, 74% yield).
^{1}H NMR (400 MHz, $CDCl_3$) δ 7.49 – 7.31 (m, 2H), 7.29 (dd, *J* = 8.5, 1.5 Hz, 2H), 4.64 (dt, *J* = 12.1, 7.4 Hz, 1H), 3.90 – 3.67 (m, 1H), 3.41 – 3.12 (m, 1H), 2.56 (dd, *J* = 13.3, 7.2 Hz, 1H), 2.02 (dd, *J* = 13.3, 7.1 Hz, 1H), 1.34 (s, 3H), 1.25 (s, 3H). ^{13}C NMR (101 MHz, $CDCl_3$) δ 178.5, 134.6, 132.6, 129.9, 125.2, 54.2 (d, *J* = 15.3 Hz), 51.2, 40.9, 40.2, 25.8, 25.2. ^{19}F NMR (376 MHz, $CDCl_3$) δ 60.73. **HRMS (ESI):** *m/z* calculated for [M] ($C_{13}H_{15}NO_3FSCl$) from $[M+H]^+$ is 320.0518, found 320.0509

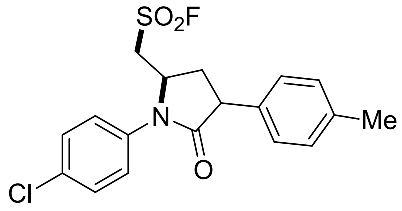

(1-(4-chlorophenyl)-5-oxo-4-(p-tolyl)pyrrolidin-2-yl)methanesulfonyl fluoride (2t)

Compound **2t** was prepared according to **General Procedure**. The residue was purified by flash column chromatography on silica (petroleum ether/ethyl acetate 5:1) to give a white solid (27.5 mg, 72% yield, *dr* = 2.8:1).
^{1}H NMR (400 MHz, $CDCl_3$) δ 7.48 (d, *J* = 9.0 Hz, 2H), 7.42 (d, *J* = 9.0 Hz, 2H), 7.16 (q, *J* = 8.2 Hz, 4H), 4.84 (td, *J* = 7.5, 2.6 Hz, 1H), 3.95 (t, *J* = 9.2 Hz, 1H), 3.71 – 3.61 (m, 1H), 3.59 – 3.48 (m, 1H), 2.83 – 2.57 (m, 2H), 2.34 (s, 3H). ^{13}C NMR (101 MHz, $CDCl_3$) δ 173.6, 137.6, 134.7, 134.4, 132.2, 129.9, 129.8, 127.9, 124.0, 52.9, 52.2 (d, *J* = 15.0 Hz), 46.7, 32.8, 21.1. ^{19}F NMR (376 MHz, $CDCl_3$) δ 60.39. **HRMS (ESI):** *m/z* calculated for [M] ($C_{18}H_{17}NO_3FSCl$) from $[M+H]^+$ is 382.0674, found 382.0659

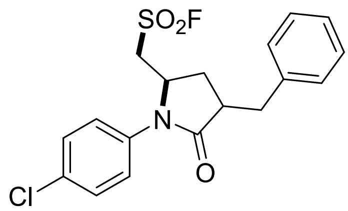

(4-benzyl-1-(4-chlorophenyl)-5-oxopyrrolidin-2-yl)methanesulfonyl fluoride (2u)

Compound **2u** was prepared according to **General Procedure**. The residue was purified by flash column chromatography on silica (petroleum ether/ethyl acetate 5:1) to give a white solid (24.4 mg, 64% yield, *dr* = 2:1).
^{1}H NMR (400 MHz, $CDCl_3$) (*dr* = 1:2) δ 7.49 – 7.12 (m, 27H), 4.64 – 4.42 (m, 1H), 4.42 – 4.20 (m, 2H), 3.54 (ddd, *J* = 14.6, 3.8, 2.3 Hz, 2H), 3.42 – 3.01 (m, 10H), 2.94 (dd, *J* = 13.6, 7.8 Hz, 2H), 2.84 – 2.67 (m, 1H), 2.41 – 2.31 (m, 4H), 2.18 – 2.06 (m, 1H), 2.07 – 1.89 (m, 1H). ^{13}C NMR (126 MHz, $CDCl_3$) δ 174.5, 137.8, 134.5, 132.2, 129.9, 129.2, 128.9, 127.1, 124.2, 53.0, 52.6 (d, *J* = 14.8 Hz),

42.5, 36.7, 29.4. ^{19}F NMR (376 MHz, $CDCl_3$) δ 60.28, 59.94. **HRMS (ESI):** *m/z* calculated for [M] ($C_{18}H_{17}NO_3FSCl$) from $[M+Na]^+$ is 404.0494, found 404.0476

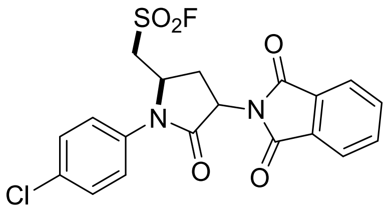

(1-(4-chlorophenyl)-4-(1,3-dioxoisoindolin-2-yl)-5-oxopyrrolidin-2-yl)methanesulfonyl fluoride (2v)

Compound **2v** was prepared according to **General Procedure**. The residue was purified by flash column chromatography on silica (petroleum ether/ethyl acetate 5:1) to give a white solid (33.2 mg, 76% yield, *dr* = 2:1).
^{1}H NMR (400 MHz, $CDCl_3$) δ 7.86 (dd, *J* = 5.4, 3.0 Hz, 2H), 7.80 – 7.70 (m, 2H), 7.53 – 7.32 (m, 4H), 5.26 (ddd, *J* = 10.8, 7.1, 3.0 Hz, 1H), 5.04 – 4.93 (m, 1H), 3.81 – 3.45 (m, 2H), 2.94 (dt, *J* = 14.3, 8.4 Hz, 1H), 2.86 – 2.66 (m, 1H). ^{13}C NMR (101 MHz, $CDCl_3$) δ 168.9, 167.4, 134.6, 134.4, 133.2, 131.7, 130.1, 124.9, 123.8, 53.0, 52.7 (d, *J* = 15.2 Hz), 48.7, 28.8. ^{19}F NMR (376 MHz, $CDCl_3$) δ 60.67. **HRMS (ESI):** *m/z* calculated for [M] ($C_{19}H_{14}N_2O_5FSCl$) from $[M+Na]^+$ is 459.0188, found 459.0183

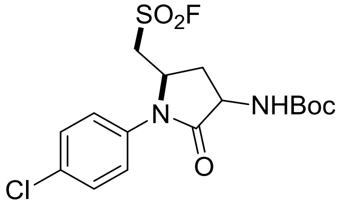

tert-butyl (1-(4-chlorophenyl)-5-((fluorosulfonyl)methyl)-2-oxopyrrolidin-3-yl)carbamate (2w)

Compound **2w** was prepared according to **General Procedure**. The residue was purified by flash column chromatography on silica (petroleum ether/ethyl acetate 5:1) to give a white solid (20.3 mg, 50% yield, *dr* = 1.5:1).
^{1}H NMR (400 MHz, $CDCl_3$) δ 7.58 – 7.34 (m, 4H), 5.18 (d, *J* = 5.9 Hz, 1H), 4.79 (dd, *J* = 11.8, 7.4 Hz, 1H), 4.45 (d, *J* = 5.8 Hz, 1H), 3.69 – 3.36 (m, 2H), 2.89 (dd, *J* = 13.6, 8.8 Hz, 1H), 2.61 – 2.26 (m, 1H), 1.45 (s, 9H). ^{13}C NMR (126 MHz, $CDCl_3$) δ 171.4, 155.7, 134.3, 132.6, 130.1, 123.9, 80.8, 52.2, 52.0 (d, *J* = 15.1 Hz), 51.3, 31.6, 28.4. ^{19}F NMR (376 MHz, $CDCl_3$) δ 60.19. **HRMS (ESI):** *m/z* calculated for [M] ($C_{16}H_{20}N_2O_5FSCl$) from $[M+Na]^+$ is 429.0658, found 429.0641

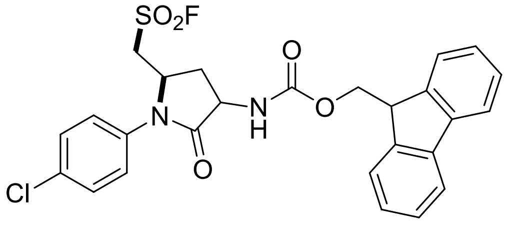

(9H-fluoren-9-yl)methyl (1-(4-chlorophenyl)-5-((fluorosulfonyl)methyl)-2-oxopyrrolidin-3-yl)carbamate (2x)

Compound **2x** was prepared according to **General Procedure**. The residue was purified by flash column chromatography on silica (petroleum ether/ethyl acetate 5:1) to give a white solid (23.8 mg, 45% yield, *dr* = 2:1).
^{1}H NMR (500 MHz, DMSO-*d*$_6$, dr = 2:1) δ 7.90 (d, *J* = 7.5 Hz, 7H), 7.81 (d, *J* = 8.6 Hz, 2H), 7.72 (t, *J* = 8.2 Hz, 6H), 7.57 (d, *J* = 8.8 Hz, 4H), 7.51 (t, *J* = 10.0 Hz, 6H), 7.42 (t, *J* = 7.1 Hz, 6H), 7.35 (dt, *J* = 12.2, 7.9 Hz, 8H), 4.98 (t, *J* = 7.1 Hz, 2H), 4.76 (d, *J* = 5.2 Hz, 1H), 4.68 (d, *J* = 10.3 Hz, 2H), 4.59 – 4.47 (m, 2H), 4.37 (ddd, *J* = 14.3, 9.6, 5.8 Hz, 7H), 4.25 (t, *J* = 6.5 Hz, 4H), 4.12 (d, *J* = 14.8 Hz, 4H), 2.76 (dt, *J* = 12.4, 9.0 Hz, 1H), 2.40 (dd, *J* = 20.6, 11.5 Hz, 2H), 2.22 – 1.91 (m, 2H). ^{13}C NMR (126 MHz, DMSO-*d*$_6$) δ 171.5, 155.9, 143.8, 140.7, 135.6, 135.1, 130.8, 129.9, 129.0, 129.0, 127.6, 127.0, 126.6, 125.1, 124.6, 120.1, 65.6, 52.9 (d, *J* = 12.0 Hz), 51.3, 51.2, 51.0, 50.6, 50.5, 46.6, 30.9, 29.5. ^{19}F NMR (376 MHz, DMSO-*d*$_6$) δ 61.33, 59.85. **HRMS (ESI):** *m/z* calculated for [M] ($C_{26}H_{22}N_2O_5FSCl$) from $[M+Na]^+$ is 551.0814, found 551.0805

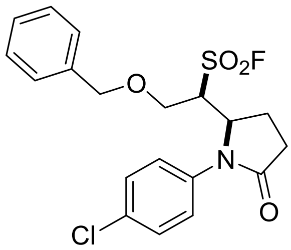

2-(benzyloxy)-1-(1-(4-chlorophenyl)-5-oxopyrrolidin-2-yl)ethanesulfonyl fluoride (2y)

Compound **2y** was prepared according to **General Procedure**. The residue was purified by flash column chromatography on silica (petroleum ether/ethyl acetate 5:1) to give a white solid (23.0 mg, 56% yield, *dr* = 1:1).
^{1}H NMR (400 MHz, $CDCl_3$) δ 7.42 – 7.33 (m, 5H), 7.31 – 7.20 (m, 4H), 5.01 (t, *J* = 5.1 Hz, 1H), 4.50 (s, 2H), 3.93 – 3.75 (m, 3H), 2.69 – 2.51 (m, 2H), 2.50 – 2.38 (m, 2H). ^{13}C NMR (101 MHz, $CDCl_3$) δ 173.9, 136.1, 134.3, 132.5, 129.9, 128.8, 128.5, 128.1, 125.1, 74.3, 63.2, 62.2 (d, *J* = 10.1 Hz), 57.0, 30.7, 19.6. ^{19}F NMR (376 MHz, $CDCl_3$) δ 58.65. **HRMS (ESI):** *m/z* calculated for [M] ($C_{19}H_{19}NO_4FSCl$) from $[M+H]^+$ is 412.0780, found 412.0769

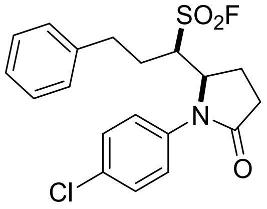

1-(1-(4-chlorophenyl)-5-oxopyrrolidin-2-yl)-3-phenylpropane-1-sulfonyl fluoride (2z)

Compound **2z** was prepared according to **General Procedure**. The residue was purified by flash column chromatography on silica (petroleum ether/ethyl acetate 5:1) to give a white solid (23.0 mg, 58% yield, *dr* = 1.6:1).
^{1}H NMR (400 MHz, $CDCl_3$) δ 7.38 – 7.21 (m, 5H), 7.17 – 6.97 (m, 4H), 4.52 (ddd, *J* = 12.9, 7.8, 3.1 Hz, 1H), 3.54 (td, *J* = 6.3, 3.0 Hz, 1H), 2.88 – 2.73 (m, 3H), 2.63 – 2.46 (m, 2H), 2.46 – 2.34 (m, 2H), 2.14 – 1.93 (m, 1H). ^{13}C NMR (101 MHz, $CDCl_3$) δ 173.7, 138.2, 134.5, 132.2, 129.6, 129.2, 128.4, 127.3, 125.5, 60.4 (d, *J* = 8.9 Hz), 59.4, 32.5, 30.2, 28.4, 18.5. ^{19}F NMR (376 MHz, $CDCl_3$) δ 60.05. **HRMS (ESI):** *m/z* calculated for [M] ($C_{19}H_{19}NO_3FSCl$) from $[M+H]^+$ is 396.0831, found 396.0817

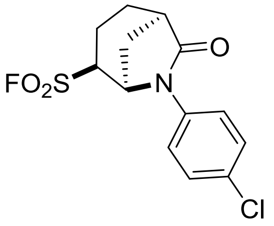

6-(4-chlorophenyl)-7-oxo-6-azabicyclo[3.2.1]octane-4-sulfonyl fluoride (2aa)

Compound **2aa** was prepared according to **General Procedure**. The residue was purified by flash column chromatography on silica (petroleum ether/ethyl acetate 5:1) to give a white solid (22.2 mg, 70% yield, *dr* = 1:1).
^{1}H NMR (400 MHz, $CDCl_3$) δ 7.59 (d, *J* = 8.8 Hz, 2H), 7.38 (d, *J* = 8.8 Hz, 2H), 4.82 (d, *J* = 2.0 Hz, 1H), 3.85 (d, *J* = 2.2 Hz, 1H), 2.78 (s, 1H), 2.48 – 2.28 (m, 3H), 2.17 – 1.92 (m, 3H). ^{13}C NMR (101 MHz, $CDCl_3$) δ 175.4, 135.3, 131.0, 129.8, 121.4, 56.6, 56.2 (d, *J* = 11.0 Hz), 40.7, 30.4, 21.8, 19.5. ^{19}F NMR (376 MHz, $CDCl_3$) δ 54.83. **HRMS (ESI):** *m/z* calculated for [M] ($C_{13}H_{13}NO_3FSCl$) from $[M+H]^+$ is 318.0361, found 318.0369

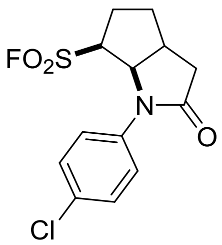

1-(4-chlorophenyl)-2-oxooctahydrocyclopenta[b]pyrrole-6-sulfonyl fluoride (2ab)

Compound **2ab** was prepared according to **General Procedure**. The residue was purified by flash column chromatography on silica (petroleum ether/ethyl acetate 10:1) to give a white solid (17.5 mg, 55% yield, *dr* > 20:1).
^{1}H NMR (400 MHz, $CDCl_3$) δ 7.41 (s, 4H), 5.12 (d, *J* = 7.4 Hz, 1H), 3.73 (dd, *J* = 4.9, 2.7 Hz, 1H), 3.17 (d, *J* = 3.5 Hz, 1H), 2.92 (dd, *J* = 17.9, 9.8 Hz, 1H), 2.53 – 2.23 (m, 4H), 1.92 – 1.65 (m, 1H). ^{13}C NMR (101 MHz, $CDCl_3$) δ 172.8, 134.5, 132.4, 129.7, 125.0, 66.2, 65.5 (d, *J* = 12.7 Hz), 37.8, 35.2, 32.2, 27.1. ^{19}F NMR (376 MHz, $CDCl_3$) δ 50.00. **HRMS (ESI):** *m/z* calculated for [M] ($C_{13}H_{13}NO_3FSCl$) from $[M+H]^+$ is 318.0361, found 318.0346

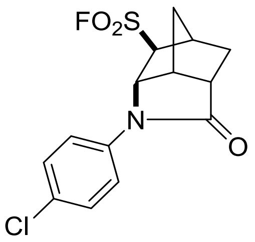

1-(4-chlorophenyl)-2-oxooctahydro-3,5-methanocyclopenta[b]pyrrole-6-sulfonyl fluoride (2ac)

Compound **2ac** was prepared according to **General Procedure**. The residue was purified by flash column chromatography on silica (petroleum ether/ethyl acetate 5:1) to give a white solid (18.5 mg, 56% yield, *dr* > 20:1).
^{1}H NMR (400 MHz, $CDCl_3$) δ 7.63 (d, *J* = 8.9 Hz, 2H), 7.35 (d, *J* = 8.9 Hz, 2H), 4.72 (d, *J* = 4.5 Hz, 1H), 3.46 (s, 1H), 3.32 (s, 1H), 3.14 (s, 1H), 2.73 (dd, *J* = 11.0, 4.4 Hz, 1H), 2.36 (d, *J* = 11.9 Hz, 1H), 2.30 – 2.14 (m, 1H), 1.79 (dd, *J* = 28.8, 12.8 Hz, 2H). ^{13}C NMR (126 MHz, $CDCl_3$) δ 176.8, 136.3, 130.6, 129.4, 121.2, 69.0 (d, *J* = 11.6 Hz), 63.5, 44.3, 42.6, 40.8, 34.8, 34.6. ^{19}F NMR (376 MHz, $CDCl_3$) δ 54.98. **HRMS (ESI):** *m/z* calculated for [M] ($C_{14}H_{13}NO_3FSCl$) from $[M+H]^+$ is 330.0361, found 330.0351

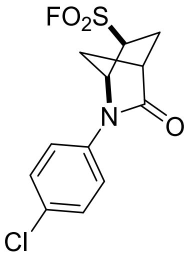

2-(4-chlorophenyl)-3-oxo-2-azabicyclo[2.2.1]heptane-6-sulfonyl fluoride (2ad)

Compound **2ad** was prepared according to **General Procedure**. The residue was purified by flash column chromatography on silica (petroleum ether/ethyl acetate 5:1) to give a white solid (8.5 mg, 28% yield, *dr* = 6:1).
^{1}H NMR (400 MHz, $CDCl_3$) δ 7.46 – 7.40 (m, 2H), 7.39 – 7.32 (m, 2H), 4.85 (s, 1H), 3.85 (dd, *J* = 7.5, 6.2 Hz, 1H), 3.16 (s, 1H), 2.56 – 2.48 (m, 1H), 2.47 – 2.36 (m, 1H), 2.20 (dt, *J* = 10.8, 6.1 Hz, 2H). ^{13}C NMR (101 MHz, $CDCl_3$) δ 174.2, 135.1, 130.5, 129.7, 119.9, 62.0, 60.7 (d, *J* = 13.7 Hz), 46.0, 36.9, 28.9. ^{19}F NMR (376 MHz, $CDCl_3$) δ 53.41. **HRMS (ESI):** *m/z* calculated for [M] ($C_{12}H_{11}NO_3FSCl$) from $[M+H]^+$ is 304.0205, found 304.0218

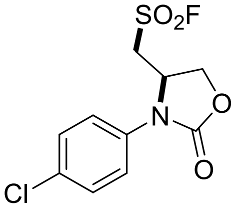

(3-(4-chlorophenyl)-2-oxooxazolidin-4-yl)methanesulfonyl fluoride (2ae)

Compound **2ae** was prepared according to **General Procedure**. The residue was purified by flash column chromatography on silica (petroleum ether/ethyl acetate 10:1) to give a white solid (14.1 mg, 48% yield).
^{1}H NMR (400 MHz, $CDCl_3$) δ 7.51 – 7.35 (m, 4H), 4.95 (dddd, *J* = 10.1, 7.9, 4.0, 2.2 Hz, 1H), 4.74 (dd, *J* = 9.5, 8.2 Hz, 1H), 4.54 (dd, *J* = 9.7, 4.0 Hz, 1H), 3.75 (dt, *J* = 14.8, 2.5 Hz, 1H), 3.62 (ddd, *J* = 14.9, 10.0, 4.9 Hz, 1H). ^{13}C NMR (101 MHz, $CDCl_3$) δ 154.1, 133.5, 132.2, 130.2, 122.7, 66.0, 52.1, 51.5 (d, *J* = 16.2 Hz). ^{19}F NMR (376 MHz, $CDCl_3$) δ 59.98. **HRMS (ESI):** *m/z* calculated for [M] ($C_{10}H_9NO_4FSCl$) from $[M+H]^+$ is 293.9998, found 293.9989

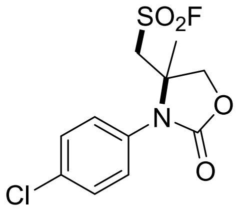

(3-(4-chlorophenyl)-4-methyl-2-oxooxazolidin-4-yl)methanesulfonyl fluoride (2af)

Compound **2af** was prepared according to **General Procedure**. The residue was purified by flash column chromatography on silica (petroleum ether/ethyl acetate 5:1) to give a white solid (19.1 mg, 62% yield).
^{1}H NMR (400 MHz, $CDCl_3$) δ 7.54 – 7.36 (m, 2H), 7.22 – 7.10 (m, 2H), 4.81 (d, *J* = 9.7 Hz, 1H), 4.31 (d, *J* = 9.7 Hz, 1H), 3.81 (dd, *J* = 14.6, 5.7 Hz, 1H), 3.51 (d, *J* = 14.6 Hz, 1H), 1.65 (s, 3H). ^{13}C NMR (126 MHz, $CDCl_3$) δ 155.8, 135.7, 131.7, 130.8, 130.4, 72.0, 60.6, 56.6 (d, *J* = 14.5 Hz), 24.1 (d, *J* = 2.1 Hz). ^{19}F NMR (376 MHz, $CDCl_3$) δ 65.40. **HRMS (ESI):** *m/z* calculated for [M] ($C_{11}H_{11}NO_4FSCl$) from $[M+Na]^+$ is 329.9974, found 329.9958

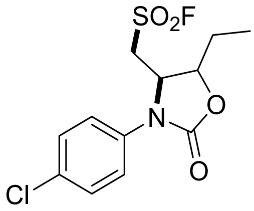

(3-(4-chlorophenyl)-5-ethyl-2-oxooxazolidin-4-yl)methanesulfonyl fluoride (2ag)

Compound **2ag** was prepared according to **General Procedure**. The residue was purified by flash column chromatography on silica (petroleum ether/ethyl acetate 5:1) to give a white solid (22.5 mg, 70% yield, *dr* = 1.6:1).
^{1}H NMR (400 MHz, $CDCl_3$) δ 7.48 – 7.39 (m, 4H), 4.64 – 4.58 (m, 1H), 4.54 (dt, *J* = 9.4, 2.6 Hz, 1H), 3.76 – 3.58 (m, 2H), 1.95 – 1.85 (m, 2H), 1.11 (t, *J* = 7.4 Hz, 3H). ^{13}C NMR (101 MHz, $CDCl_3$) δ 153.5, 133.7, 131.8, 130.1, 122.3, 79.1, 56.4, 51.5 (d, *J* = 15.5 Hz), 28.1, 8.7. ^{19}F NMR (376 MHz, $CDCl_3$) δ 60.46. **HRMS (ESI):** *m/z* calculated for [M] ($C_{12}H_{13}NO_4$ FSCl) from $[M+Na]^+$ is 344.0130, found 344.0115

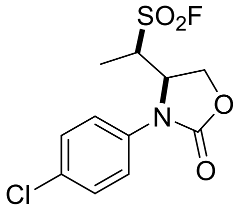

1-(3-(4-chlorophenyl)-2-oxooxazolidin-4-yl)ethanesulfonyl fluoride (2ah)

Compound **2ah** was prepared according to **General Procedure**. The residue was purified by flash column chromatography on silica (petroleum ether/ethyl acetate 5:1) to give a white solid (22.1 mg, 72% yield, *dr* = 1:1).
^{1}H NMR (400 MHz, $CDCl_3$) δ 7.43 (s, 4H), 5.30 – 5.02 (m, 1H), 4.75 – 4.43 (m, 2H), 3.98 – 3.53 (m, 1H), 1.54 (d, *J* = 7.1 Hz, 3H). ^{13}C NMR (126 MHz, $CDCl_3$) δ 154.5, 133.3, 132.2, 130.2, 122.9, 61.8, 56.0 (d, *J* = 12.4 Hz), 54.6, 7.7. ^{19}F NMR (376 MHz, $CDCl_3$) δ 50.46. **HRMS (ESI):** *m/z* calculated for [M] ($C_{11}H_{11}NO_4FSCl$) from $[M+Na]^+$ is 329.9974, found 329.9964

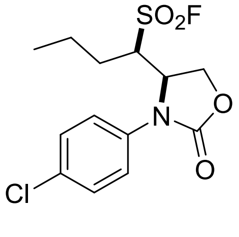

1-(3-(4-chlorophenyl)-2-oxooxazolidin-4-yl)butane-1-sulfonyl fluoride (2ai)

Compound **2ai** was prepared according to **General Procedure**. The residue was purified by flash column chromatography on silica (petroleum ether/ ethyl acetate 5:1) to give a white solid (21.5 mg, 64% yield, *dr* = 1.5:1).
^{1}H NMR (400 MHz, $CDCl_3$) δ 7.52 – 7.27 (m, 4H), 4.93 (dt, *J* = 8.2, 5.0 Hz, 1H), 4.71 – 4.53 (m, 2H), 3.60 (td, *J* = 6.6, 2.8 Hz, 1H), 2.04 (td, *J* = 14.8, 7.0 Hz, 1H), 1.85 – 1.61 (m, 1H), 1.61 – 1.48 (m, 2H), 0.97 (t, *J* = 7.3 Hz, 3H). ^{13}C NMR (101 MHz, $CDCl_3$) δ 154.8, 133.9, 132.3, 129.9, 124.2, 63.0, 61.4 (d, *J* = 10.4 Hz), 56.1, 28.6, 20.2, 13.6. ^{19}F NMR (376 MHz, $CDCl_3$) δ 59.03. **HRMS (ESI):** *m/z* calculated for [M] ($C_{13}H_{15}NO_4FSCl$) from $[M+Na]^+$ is 358.0287, found 358.0271

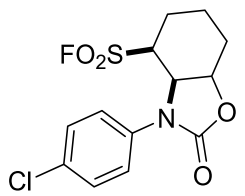

3-(4-chlorophenyl)-2-oxooctahydrobenzo[d]oxazole-4-sulfonyl fluoride (2aj)

Compound **2aj** was prepared according to **General Procedure**. The residue was purified by flash column chromatography on silica (petroleum ether/ethyl acetate 5:1) to give a white solid (18.7 mg, 56% yield, *dr* = 6:1).
^{1}H NMR (400 MHz, $CDCl_3$) δ 7.45 – 7.38 (m, 2H), 7.38 – 7.31 (m, 2H), δ 4.95 (dd, *J* = 11.1, 5.4 Hz, 1H), 4.90 (dd, *J* = 6.4, 3.1 Hz, 1H). 3.77 (d, *J* = 3.5 Hz, 1H), 2.21 – 1.99 (m, 4H), 1.91 – 1.70 (m, 2H). ^{13}C NMR (101 MHz, $CDCl_3$) δ 154.9, 134.0, 132.3, 129.8, 124.1, 72.4, 59.5 (d, *J* = 12.3 Hz), 54.2, 25.6, 21.3, 16.0. ^{19}F NMR (376 MHz, $CDCl_3$) δ 53.08. **HRMS (ESI):** *m/z* calculated for [M] ($C_{13}H_{13}NO_4FSCl$) from $[M+Na]^+$ is 356.0130, found 356.0117

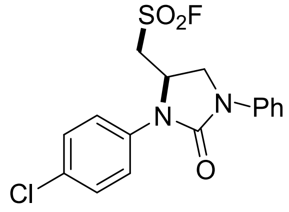

(3-(4-chlorophenyl)-2-oxo-1-phenylimidazolidin-4-yl)methanesulfonyl fluoride (2ak)

Compound **2ak** was prepared according to **General Procedure**. The residue was purified by flash column chromatography on silica (petroleum ether/ethyl acetate 5:1 to 2:1) to give a white solid (7.7 mg, 21% yield).
^{1}H NMR (400 MHz, DMSO-d_6) δ 7.66 – 7.52 (m, 4H), 7.53 – 7.44 (m, 2H), 7.39 (dd, *J* = 10.8, 5.3 Hz, 2H), 7.10 (t, *J* = 7.3 Hz, 1H), 5.21 (d, *J* = 8.2 Hz, 1H), 4.60 – 4.43 (m, 1H), 4.37 – 4.20 (m, 2H), 4.03 (dd, *J* = 9.7, 3.6 Hz, 1H). ^{13}C NMR (101 MHz, DMSO-d_6) δ 153.6, 139.4, 136.2, 128.8, 128.8, 128.5, 123.4, 123.0, 118.1, 51.2 (d, *J* = 11.8 Hz), 47.9, 46.2. ^{19}F NMR (376 MHz, DMSO-d_6) δ 61.37. **HRMS (ESI):** *m/z* calculated for [M] ($C_{16}H_{14}N_2O_3FSCl$) from $[M+Na]^+$ is 391.0290, found 391.0300

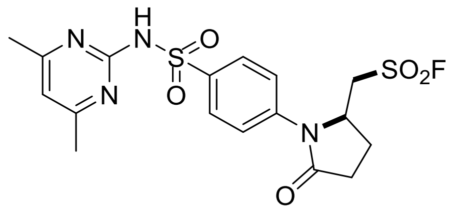

(1-(4-(N-(4,6-dimethylpyrimidin-2-yl)sulfamoyl)phenyl)-5-oxopyrrolidin-2-yl)methanesulfonyl fluoride (2al)

Compound **2al** was prepared according to **General Procedure**. The residue was purified by flash column chromatography on silica (petroleum ether/ethyl acetate 2:1 to 1:1) to give a faint yellow solid (15.5 mg, 35% yield).
^{1}H NMR (500 MHz, DMSO-d_6) δ 11.77 (s, 1H), 8.01 (d, *J* = 8.6 Hz, 2H), 7.70 (d, *J* = 8.6 Hz, 2H), 6.76 (s, 1H), 5.02 (t, *J* = 7.6 Hz, 1H), 4.44 – 4.28 (m, 1H), 4.13 (d, *J* = 14.9 Hz, 1H), 2.84 – 2.63 (m, 1H), 2.43 (dd, *J* = 21.7, 10.3 Hz, 2H), 2.25 (s, 6H), 2.16 (t, *J* = 10.4 Hz, 1H). ^{13}C NMR (126 MHz, DMSO-d_6) δ 173.8, 156.0, 140.2, 131.1, 128.9, 128.3, 126.3, 121.6, 53.8, 51.6 (d, *J* = 11.8 Hz), 30.0, 22.8, 22.7. ^{19}F NMR (376 MHz, DMSO-d_6) δ 60.54. **HRMS (ESI):** *m/z* calculated for [M] ($C_{17}H_{19}N_4O_5FS_2$) from $[M+H]^+$ is 443.0854, found 443.0870

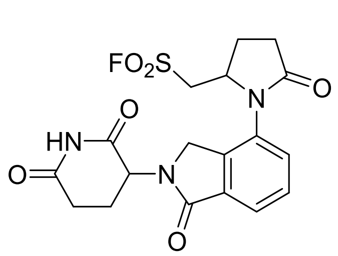

(1-(2-(2,6-dioxopiperidin-3-yl)-1-oxoisoindolin-4-yl)-5-oxopyrrolidin-2-yl)methanesulfonyl fluoride (2am)

Compound **2am** was prepared according to **General Procedure**. The residue was purified by flash column chromatography on silica (petroleum ether/ethyl acetate 1:1) to give a white solid (20.3 mg, 48% yield).
^{1}H NMR (400 MHz, DMSO-d_6, dr = 1:1) δ 11.01 (s, 1H), 7.72 (dd, *J* = 7.1, 1.6 Hz, 1H), 7.70 – 7.58 (m, 2H), 5.22 – 5.06 (m, 1H), 4.91 – 4.77 (m, 1H), 4.46 – 4.20 (m, 3H), 4.16 – 3.99 (m, 1H), 2.99 – 2.77 (m, 1H), 2.74 – 2.42 (m, 5H), 2.41 – 2.27 (m, 1H), 2.25 – 2.12

(m, 1H), 1.99 (ddd, J = 14.5, 7.3, 1.9 Hz, 1H). ^{13}C NMR (101 MHz, DMSO-d_6) δ 173.0, 172.8, 170.8, 167.4, 139.1, 133.1, 129.2, 128.7, 126.1, 122.2, 54.9, 52.5 (d, J = 11.8 Hz), 51.3, 46.5, 31.1, 29.3, 23.9, 22.6. ^{19}F NMR (376 MHz, DMSO-d_6) δ 60.99, 60.31. **HRMS (ESI):** *m/z* calculated for [M] ($C_{18}H_{18}N_3O_6FS$) from $[M+H]^+$ is 424.0973, found 424.0966

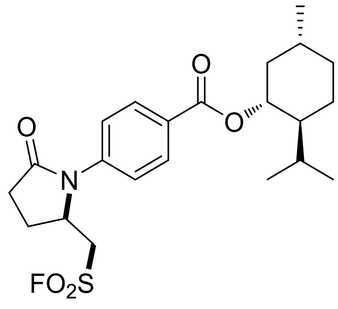

2-isopropyl-5-methylcyclohexyl 4-(2-((fluorosulfonyl)methyl)-5-oxopyrrolidin-1-yl)benzoate (2an)

Compound **2an** was prepared according to **General Procedure**. The residue was purified by flash column chromatography on silica (petroleum ether/ethyl acetate 5:1 to 3:1) to give a white solid (25.5 mg, 58% yield, *dr* = 1:1).
^{1}H NMR (400 MHz, $CDCl_3$, dr = 1:1) δ 8.11 (d, J = 8.4 Hz, 2H), 7.61 – 7.46 (m, 2H), 5.07 – 4.77 (m, 2H), 3.69 (ddd, J = 14.6, 5.8, 3.9 Hz, 1H), 3.44 (ddd, J = 14.6, 9.9, 2.6 Hz, 1H), 2.86 – 2.55 (m, 3H), 2.40 – 2.23 (m, 1H), 2.15 – 2.05 (m, 1H), 1.93 (tdd, J = 13.7, 6.9, 2.7 Hz, 1H), 1.73 (d, J = 11.5 Hz, 2H), 1.60 – 1.50 (m, 2H), 1.17 – 1.04 (m, 2H), 0.98 – 0.86 (m, 7H), 0.79 (d, J = 6.9 Hz, 3H). ^{13}C NMR (101 MHz, $CDCl_3$) δ 173.4, 165.2, 139.8, 131.2, 128.7, 122.1, 75.2, 54.3, 52.8 (d, J = 13.9 Hz), 47.3, 41.0, 34.4, 31.5, 30.4, 26.6, 23.7, 22.1, 20.8, 16.6. ^{19}F NMR (376 MHz, $CDCl_3$) δ 60.63, 60.54. **HRMS (ESI):** *m/z* calculated for [M] ($C_{22}H_{30}NO_5FS$) from $[M+Na]^+$ is 462.1721, found 462.1716

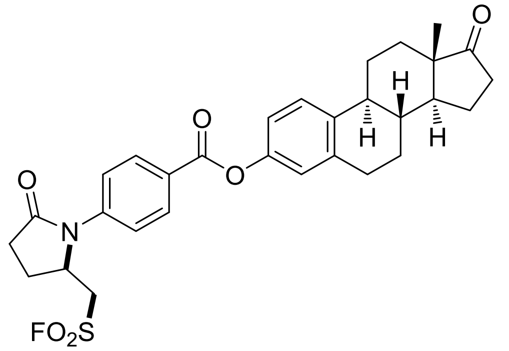

13-methyl-17-oxo-7,8,9,11,12,13,14,15,16,17-decahydro-6H-cyclopenta[a]phenanthren-3-yl 4-(2-((fluorosulfonyl)methyl)-5-oxopyrrolidin-1-yl)benzoate (2ao)

Compound **2ao** was prepared according to **General Procedure**. The residue was purified by flash column chromatography on silica (petroleum ether/ ethyl acetate 3:1 to 1:1) to give a white solid (25.5 mg, 46% yield).
^{1}H NMR (400 MHz, $CDCl_3$) δ 8.27 (d, J = 8.9 Hz, 2H), 7.64 (d, J = 8.8 Hz, 2H), 7.34 (d, J = 8.5 Hz, 1H), 7.05 – 6.79 (m, 2H), 5.01 – 4.82 (m, 1H), 3.72 (ddd, J = 14.6, 3.9, 1.9 Hz, 1H), 3.48 (ddd, J = 14.6, 9.8, 2.5 Hz, 1H), 3.01 – 2.90 (m, 2H), 2.84 – 2.61 (m, 3H), 2.56 – 2.39 (m, 2H), 2.32 (ddd, J = 11.6, 8.9, 3.7 Hz, 2H), 2.23 – 1.87 (m, 5H), 1.68 – 1.58 (m, 1H), 1.57 – 1.40 (m, 4H), 0.92 (s, 3H). ^{13}C NMR (126 MHz, $CDCl_3$) δ 220.9, 173.4, 164.6, 148.8, 140.7, 138.3, 137.7, 131.8, 127.2, 126.6, 121.8, 121.7, 118.9, 54.2, 52.8 (d, J = 15.0 Hz), 50.5, 48.0, 44.3, 38.1, 35.9, 31.6, 30.4, 29.5, 26.4, 25.9, 23.6, 21.7, 13.9. ^{19}F NMR (376 MHz, $CDCl_3$) δ 60.60. **HRMS (ESI):** *m/z* calculated for [M] ($C_{30}H_{32}NO_6FS$) from $[M+H]^+$ is 554.2007, found 554.2014

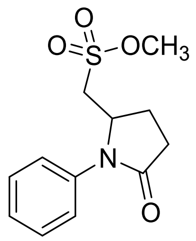

methyl (5-oxo-1-phenylpyrrolidin-2-yl)methanesulfonate (D1)

^{1}H NMR (400 MHz, $CDCl_3$) δ 7.53 – 7.31 (m, 4H), 7.32 – 7.19 (m, 1H), 4.89 – 4.57 (m, 1H), 3.85 (s, 3H), 3.40 (dd, J = 14.3, 2.0 Hz, 1H), 3.11 (dd, J = 14.2, 10.2 Hz, 1H), 2.76 – 2.50 (m, 3H), 2.35 – 2.13 (m, 1H). ^{13}C NMR (101 MHz, $CDCl_3$) δ 173.7, 136.3, 129.6, 126.6, 123.5, 55.6, 55.1, 51.8, 30.6, 24.1. **HRMS (ESI):** *m/z* calculated for [M] ($C_{12}H_{15}NO_4S$) from $[M+H]^+$ is 270.0795, found 270.0769

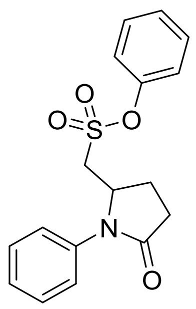

phenyl (5-oxo-1-phenylpyrrolidin-2-yl)methanesulfonate (D2)

^{1}H NMR (400 MHz, $CDCl_3$) δ 7.47 – 7.23 (m, 8H), 7.14 (d, *J* = 8.0 Hz, 2H), 4.97 – 4.58 (m, 1H), 3.59 (dd, *J* = 14.2, 2.1 Hz, 1H), 3.28 (dd, *J* = 14.2, 10.3 Hz, 1H), 2.84 – 2.53 (m, 3H), 2.50 – 2.20 (m, 1H). ^{13}C NMR (101 MHz, $CDCl_3$) δ 173.7, 148.7, 136.3, 130.2, 129.7, 127.6, 126.8, 123.9, 121.9, 55.4, 52.6, 30.5, 24.1. **HRMS (ESI):** *m/z* calculated for [M] ($C_{17}H_{17}NO_4S$) from $[M+H]^+$ is 332.0951, found 332.0949

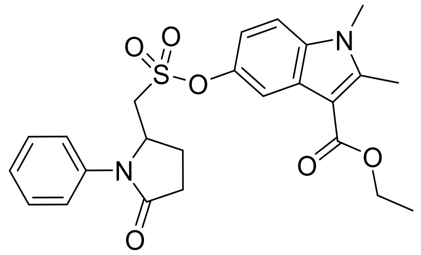

ethyl 1,2-dimethyl-5-((((5-oxo-1-phenylpyrrolidin-2-yl)methyl)sulfonyl)oxy)-1H-indole-3-carboxylate (D3)

^{1}H NMR (400 MHz, $CDCl_3$) δ 7.94 (d, *J* = 2.4 Hz, 1H), 7.47 – 7.32 (m, 4H), 7.25 – 7.17 (m, 2H), 7.02 (dd, *J* = 8.8, 2.4 Hz, 1H), 4.96 – 4.71 (m, 1H), 4.36 (q, *J* = 7.1 Hz, 2H), 3.64 (s, 3H), 3.64 – 3.58 (m, 1H), 3.29 (dd, *J* = 14.1, 10.3 Hz, 1H), 2.73 (s, 3H), 2.70 – 2.54 (m, 3H), 2.46 – 2.30 (m, 1H), 1.40 (t, *J* = 7.1 Hz, 3H). ^{13}C NMR (101 MHz, $CDCl_3$) δ 173.7, 165.4, 147.0, 143.9, 136.3, 135.0, 129.5, 127.2, 126.5, 123.6, 115.9, 114.3, 110.0, 104.5, 59.7, 55.3, 52.3, 30.5, 29.9, 24.0, 14.6, 12.0. **HRMS (ESI):** *m/z* calculated for [M] ($C_{24}H_{26}N_2O_6S$) from $[M+Na]^+$ is 493.1404, found 493.1403

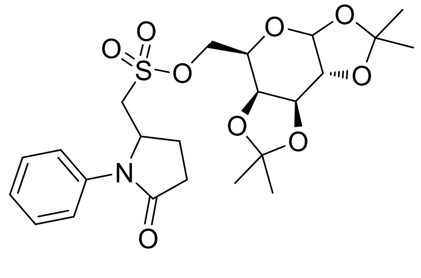

((5R,5aS,8aS,8bR)-2,2,7,7-tetramethyltetrahydro-3aH-bis([1,3]dioxolo)[4,5-b:4‘,5‘-d]pyran-5-yl)methyl (5-oxo-1-phenylpyrrolidin-2-yl)methanesulfonate (D4)

^{1}H NMR (400 MHz, $CDCl_3$) δ 7.48 – 7.34 (m, 4H), 7.28 – 7.15 (m, 1H), 5.43 (dd, *J* = 51.0, 5.0 Hz, 1H), 4.82 – 4.68 (m, 1H), 4.60 (ddd, *J* = 7.8, 3.7, 2.7 Hz, 1H), 4.43 – 4.26 (m, 3H), 4.18 (ddd, *J* = 7.8, 3.2, 2.0 Hz, 1H), 4.06 (ddd, *J* = 8.4, 4.5, 1.7 Hz, 1H), 3.50 (ddd, *J* = 36.2, 14.3, 2.3 Hz, 1H), 3.22 (ddd, *J* = 34.0, 14.3, 10.4 Hz, 1H), 2.76 – 2.62 (m, 1H), 2.63 – 2.47 (m, 2H), 2.40 – 2.25 (m, 1H), 1.55 – 1.37 (m, 6H), 1.35 – 1.24 (m, 6H). ^{13}C NMR (126 MHz, $CDCl_3$) δ 173.9, 136.4, 129.5, 126.4, 123.6, 110.0, 109.1, 96.2, 70.7, 70.2, 69.7, 66.3, 55.3, 52.9, 30.7, 25.9, 24.9, 24.4, 23.9. **HRMS (ESI):** *m/z* calculated for [M] ($C_{23}H_{31}NO_9S$) from $[M+H]^+$ is 520.1612, found 520.1598

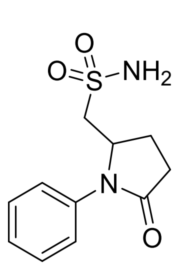

(5-oxo-1-phenylpyrrolidin-2-yl)methanesulfonamide (D5)

^{1}H NMR (400 MHz, DMSO-d_6) δ 7.51 (dd, *J* = 8.5, 0.9 Hz, 2H), 7.42 (t, *J* = 7.9 Hz, 2H), 7.22 (t, *J* = 7.3 Hz, 1H), 7.09 (s, 2H), 4.83 – 4.47 (m, 1H), 3.31 (dd, *J* = 13.9, 10.4 Hz, 1H), 3.07 (dd, *J* = 13.9, 1.5 Hz, 1H), 2.76 – 2.59 (m, 1H), 2.47 – 2.32 (m, 2H), 2.28 – 2.12 (m, 1H). ^{13}C NMR (101 MHz, DMSO-d_6) δ 173.5, 137.2, 129.0, 125.3, 122.9, 56.0, 54.9, 30.2, 23.6. **HRMS (ESI):** *m/z* calculated for [M] ($C_{11}H_{14}N_2O_3S$) from $[M+Na]^+$ is 277.0617, found 277.0601

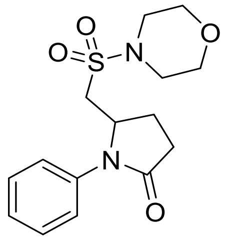

5-((morpholinosulfonyl)methyl)-1-phenylpyrrolidin-2-one (D6)

^{1}H NMR (400 MHz, $CDCl_3$) δ 7.52 – 7.35 (m, 4H), 7.29 – 7.16 (m, 1H), 4.88 – 4.55 (m, 1H), 3.82 – 3.44 (m, 4H), 3.23 – 3.05 (m, 5H), 2.85 (dd, *J* = 13.4, 10.3 Hz, 1H), 2.74 – 2.51 (m, 3H), 2.38 – 2.24 (m, 1H). ^{13}C NMR (101 MHz, $CDCl_3$) δ 173.8, 136.6, 129.5, 126.3, 123.1, 66.4, 54.9, 50.1, 45.6, 30.7, 24.4. **HRMS (ESI):** *m/z* calculated for [M] ($C_{15}H_{20}N_2O_4S$) from $[M+H]^+$ is 325.1217, found 325.1216

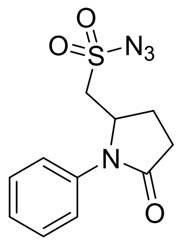

(5-oxo-1-phenylpyrrolidin-2-yl)methanesulfonyl azide (D7)

^{1}H NMR (400 MHz, $CDCl_3$) δ 7.50 – 7.35 (m, 4H), 7.33 – 7.22 (m, 1H), 4.88 – 4.62 (m, 1H), 3.60 (d, *J* = 14.2 Hz, 1H), 3.44 – 3.26 (m, 1H), 2.80 – 2.51 (m, 3H), 2.43 – 2.19 (m, 1H). ^{13}C NMR (101 MHz, $CDCl_3$) δ 173.6, 136.1, 129.8, 126.9, 123.6, 57.6, 54.9, 30.5, 24.1. **HRMS (ESI):** *m/z* calculated for [M] ($C_{11}H_{12}N_4O_3S$) from $[M+H]^+$ is 281.0703, found 281.0698

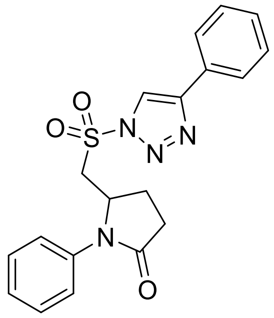

1-phenyl-5-(((4-phenyl-1H-1,2,3-triazol-1-yl)sulfonyl)methyl)pyrrolidin-2-one (D8)

^{1}H NMR (400 MHz, $CDCl_3$) δ 8.25 (s, 1H), 7.84 (dd, *J* = 8.1, 1.2 Hz, 2H), 7.53 – 7.39 (m, 3H), 7.34 (t, *J* = 7.8 Hz, 2H), 7.29 – 7.23 (m, 2H), 7.20 (t, *J* = 7.3 Hz, 1H), 4.82 (ddd, *J* = 7.4, 6.0, 3.5 Hz, 1H), 3.84 (d, *J* = 2.0 Hz, 1H), 3.68 (dd, *J* = 14.6, 9.9 Hz, 1H), 2.77 – 2.45 (m, 3H), 2.15 (tt, *J* = 12.8, 4.7 Hz, 1H). ^{13}C NMR (101 MHz, $CDCl_3$) δ 173.3, 147.9, 135.7, 129.7, 129.6, 129.2, 128.3, 126.9, 126.2, 123.4, 119.3, 57.6, 54.5, 30.3, 24.0. **HRMS (ESI):** *m/z* calculated for [M] ($C_{19}H_{18}N_4O_3S$) from $[M+H]^+$ is 383.1172, found 383.1184

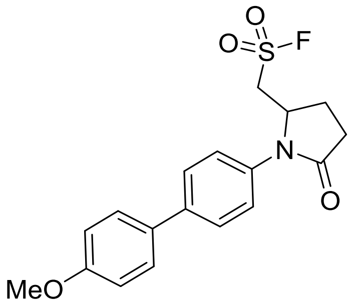

(1-(4'-methoxy-[1,1'-biphenyl]-4-yl)-5-oxopyrrolidin-2-yl)methanesulfonyl fluoride (D9)

^{1}H NMR (400 MHz, $CDCl_3$) δ 7.62 (d, *J* = 8.6 Hz, 2H), 7.51 (d, *J* = 8.7 Hz, 2H), 7.43 (d, *J* = 8.6 Hz, 2H), 6.99 (d, *J* = 8.7 Hz, 2H), 4.86 – 4.72 (m, 1H), 3.86 (s, 3H), 3.75 (ddd, *J* = 14.6, 4.4, 2.2 Hz, 1H), 3.43 (ddd, *J* = 14.5, 10.0, 2.7 Hz, 1H), 2.81 – 2.56 (m, 3H), 2.40 – 2.16 (m, 1H). ^{13}C NMR (101 MHz, $CDCl_3$) δ 173.4, 159.5, 139.7, 134.3, 132.5, 128.2, 128.0, 124.0, 114.4, 55.5, 54.9, 53.3 (d, *J* = 14.6 Hz), 30.3, 23.9. ^{19}F NMR (376 MHz, $CDCl_3$) δ 60.62. **HRMS (ESI):** *m/z* calculated for [M] ($C_{18}H_{18}NO_4FS$) from $[M+H]^+$ is 364.1013, found 364.0999

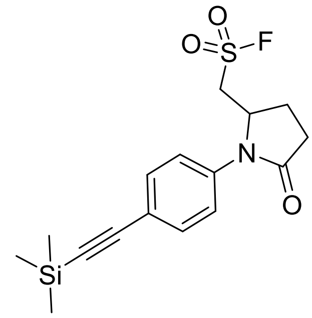

(5-oxo-1-(4-((trimethylsilyl)ethynyl)phenyl)pyrrolidin-2-yl)methanesulfonyl fluoride (D10)

^{1}H NMR (400 MHz, $CDCl_3$) δ 7.53 (d, *J* = 8.7 Hz, 2H), 7.39 (d, *J* = 8.6 Hz, 2H), 4.87 – 4.72 (m, 1H), 3.71 – 3.60 (m, 1H), 3.44 – 3.32 (m, 1H), 2.80 – 2.56 (m, 3H), 2.33 – 2.18 (m, 1H), 0.25 (s, 9H). ^{13}C NMR (126 MHz, $CDCl_3$) δ 173.2, 135.9, 133.4, 122.6, 121.6, 104.0, 95.5, 54.5, 52.9 (d, *J* = 15.0 Hz), 30.4, 23.7, 0.04. ^{19}F NMR (376 MHz, $CDCl_3$) δ 60.54. **HRMS (ESI):** *m/z* calculated for [M] ($C_{16}H_{20}NO_3FsiS$) from $[M+H]^+$ is 354.0990, found 354.0985

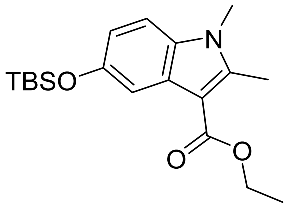

ethyl 5-((tert-butyldimethylsilyl)oxy)-1,2-dimethyl-1H-indole-3-carboxylate (S5)

^{1}H NMR (500 MHz, $CDCl_3$) δ 7.60 (d, *J* = 2.0 Hz, 1H), 7.11 (d, *J* = 8.7 Hz, 1H), 6.78 (dd, *J* = 8.7, 2.1 Hz, 1H), 4.38 (q, *J* = 7.1 Hz, 2H), 3.63 (s, 3H), 2.73 (s, 3H), 1.45 (t, *J* = 7.1 Hz, 3H), 1.02 (s, 9H), 0.23 (s, 6H). ^{13}C NMR (126 MHz, $CDCl_3$) δ 166.3, 151.1, 145.7, 132.1, 127.4, 115.8, 111.5, 109.4, 103.5, 59.3, 29.7, 25.9, 18.4, 14.7, 12.0, -4.2. **HRMS (ESI):** *m/z* calculated for [M] ($C_{19}H_{29}NO_3Si$) from $[M+H]^+$ is 348.1989, found 348.1985

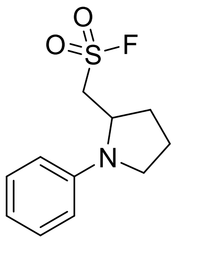

(1-phenylpyrrolidin-2-yl)methanesulfonyl fluoride (D11)

^{1}H NMR (400 MHz, $CDCl_3$) δ 7.28 (dd, *J* = 12.0, 10.9 Hz, 2H), 6.78 (t, *J* = 7.3 Hz, 1H), 6.61 (d, *J* = 8.0 Hz, 2H), 4.46 – 4.16 (m, 1H), 3.70 (dd, *J* = 14.6, 2.8 Hz, 1H), 3.47 (td, *J* = 8.6, 2.1 Hz, 1H), 3.33 – 3.11 (m, 2H), 2.32 – 1.96 (m, 4H). ^{13}C NMR (101 MHz, $CDCl_3$) δ 145.3, 129.9, 117.5, 112.2, 53.8, 52.3 (d, *J* = 10.1 Hz), 47.9, 30.7, 22.9. ^{19}F NMR (376 MHz, $CDCl_3$) δ 58.62. **HRMS (ESI):** *m/z* calculated for [M] ($C_{25}H_{22}NO_3SCl$) from $[M+H]^+$ is 452.1082, found 452.1088

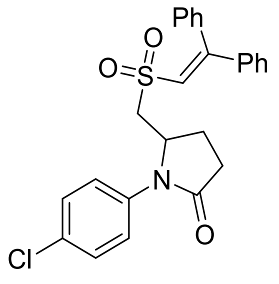

1-(4-chlorophenyl)-5-(((2,2-diphenylvinyl)sulfonyl)methyl)pyrrolidin-2-one (D14)

^{1}H NMR (400 MHz, $CDCl_3$) δ 7.46 – 7.37 (m, 2H), 7.38 – 7.28 (m, 8H), 7.22 (dd, *J* = 11.0, 4.0 Hz, 4H), 6.78 (s, 1H), 4.69 (ddd, *J* = 13.0, 6.2, 3.8 Hz, 1H), 2.96 (dd, *J* = 13.4, 1.7 Hz, 1H), 2.77 (dd, *J* = 13.4, 10.3 Hz, 1H), 2.57 – 2.43 (m, 3H), 2.19 (dt, *J* = 10.6, 9.0 Hz, 1H). ^{13}C NMR (101 MHz, $CDCl_3$) δ 173.8, 157.0, 138.7, 135.2, 135.1, 131.4, 131.0, 129.8, 129.8, 129.6, 128.9, 128.4, 128.3, 126.9, 124.0, 56.0, 53.8, 30.6, 24.6. **HRMS (ESI):** *m/z* calculated for [M] ($C_{25}H_{22}NO_3SCl$) from $[M+H]^+$ is 452.1082, found 452.1088

11. References

[1] D. C. Miller, G. J. Choi, H. S. Orbe, R. R. Knowles, *J. Am. Chem. Soc.* **2015**, *137*, 13492;

[2] J.-B. Peng, F.-P. Wu, D. Li, H.-Q. Geng,X. Qi, J. Ying, X.-F. Wu, *ACS Catal.* **2019**, 9, 2977;

[3] S. Zheng, S.-Q. Zhang, B. Saeednia, J. Zhou, J. M. Anna, X. Hong, G. A. Molander, *Chem. Sci.* **2020**, *11*, 4131;

[4] M. L. O'Duill, R. Matsuura, Y. Wang, J.L. Turnbull, J. A. Gurak, Jr., D.-W. Gao, G. Lu, P. Liu, K. M. Engle, *J. Am. Chem. Soc.* ***2017***, 139, 15576;

[5] Y. Liu, H. Wu, Y. Guo, J. C. Xiao, Q. Y. Chen, C. Liu, *Angew. Chem. Int. Ed.* **2017**, 56, 15432; *Angew. Chem.* **2017**, 129, 15634;

[6] R. Xu, T. Xu, M. Yang, T. Cao, S. Liao, *Nat. Commun.* **2019**, 10, 3752;

[7] J. Chen, B.-q. Huang, Z.-q. Wang, X.-j. Zhang, M. Yan, *Org. Lett.* **2019**, *21*, 9742.

[8] C. Liu, C. Yang, S. Hwang, S. L. Ferraro, J. P. Flynn, J. Niu, Angew. Chem. Int. Ed. **2020**, 59, 18435. *Angew. Chem.* **2020**, 132, 18593;

[9] X. Chen, J. Ren, H. Xie, W. Sun, M. Sun and B. Wu, *Org. Chem. Front.*, **2018**, 5, 184;

[10] O. Fadeyi, M. D. Parikh, M. Z. Chen, R. E. Kyne, Jr., A. P. Taylor, I. O'Doherty, S. E. Kaiser, S. Barbas, S. Niessen, M. Shi, S. L. Weinrich, J. C. Kath, L. H. Jones, R. P. Robinson, *Chembiochem* **2016**, *17*, 1925;

[11] C. J. Collins, M. Lanz, B. Singaram, *Tetrahedron Lett.* **1999**, 40, 3673;

[12] (a) D. F. Chen, J. C. K. Chu, T. Rovis, *J. Am. Chem. Soc.* **2017**, *139*, 14897; (b) J. C. Chu, T. Rovis, *Nature* **2016**, *539*, 272;

[13] Z. Li, L. Song, C. Li, *J. Am. Chem. Soc.* **2013**, *135*, 4640;

[14] F. Foschi, C. Loro, R. Sala, J. Oble, L. L. Presti, E. M. Beccalli, G. Poli, G. Broggini, *Org. Lett.* **2020**, 22, 1402;

12. NMR Spectra of Compounds 1d, 1e, 1l-1r, 1t-1ad, 1af-1ao, 2a-2ao, D1-D14, S5

^{1}H-NMR (400 MHz, $CDCl_3$)

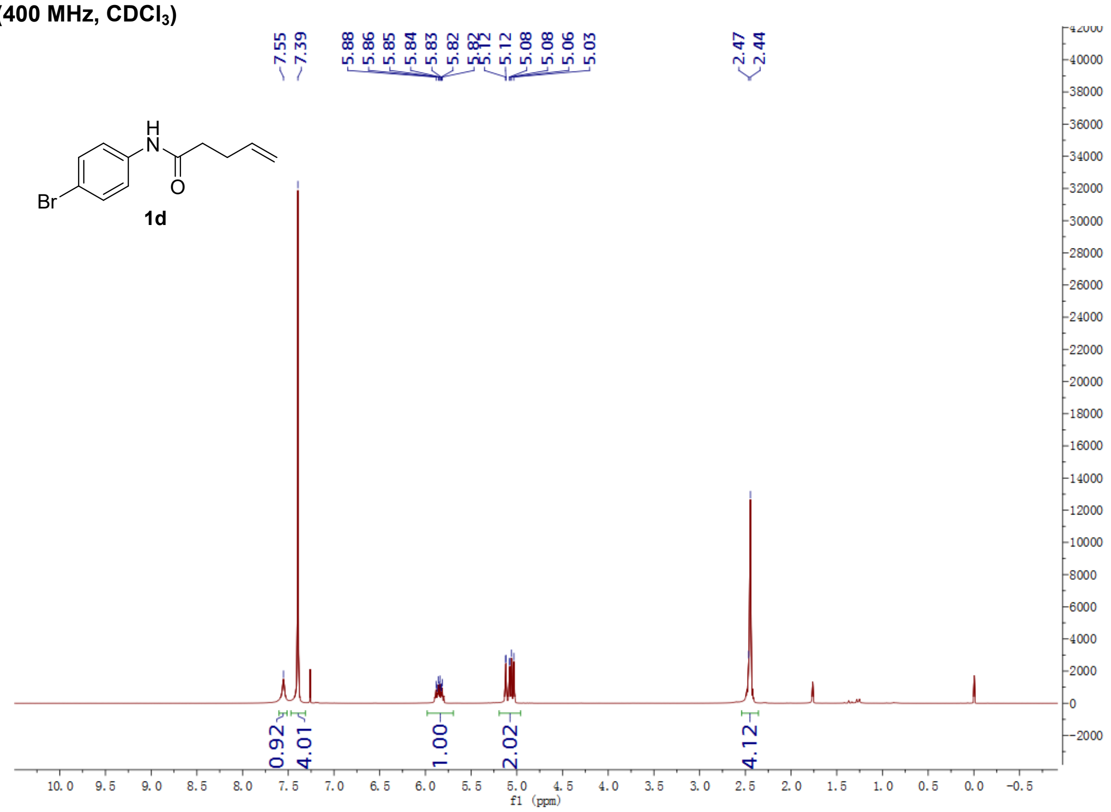

^{13}C-NMR (101 MHz, CDCl3)

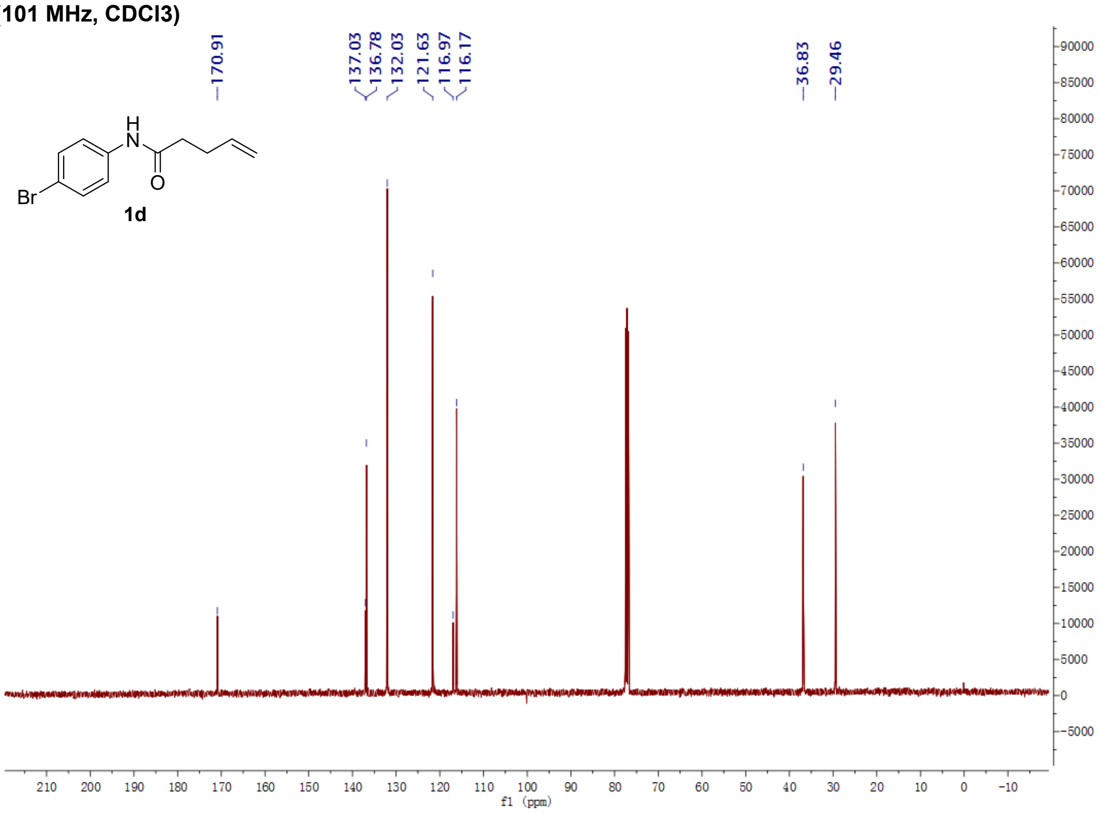

^{1}H-NMR (400 MHz, $CDCl_3$)

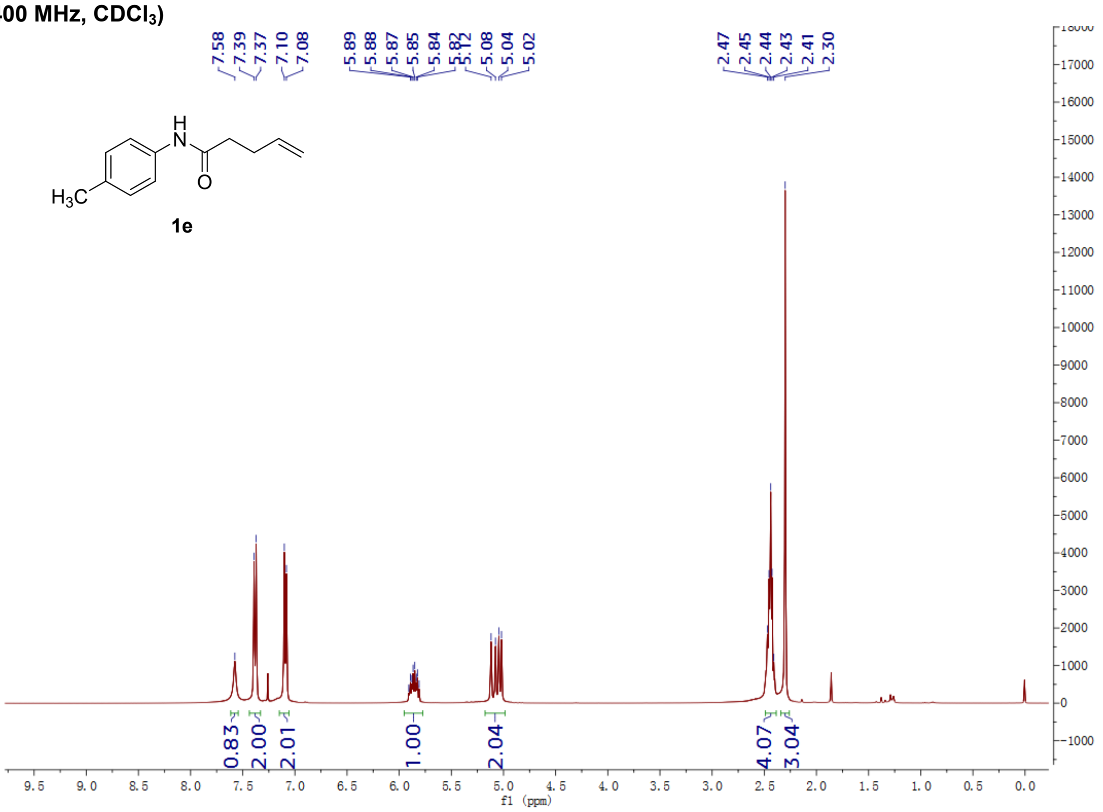

^{13}C-NMR (101 MHz, $CDCl_3$)

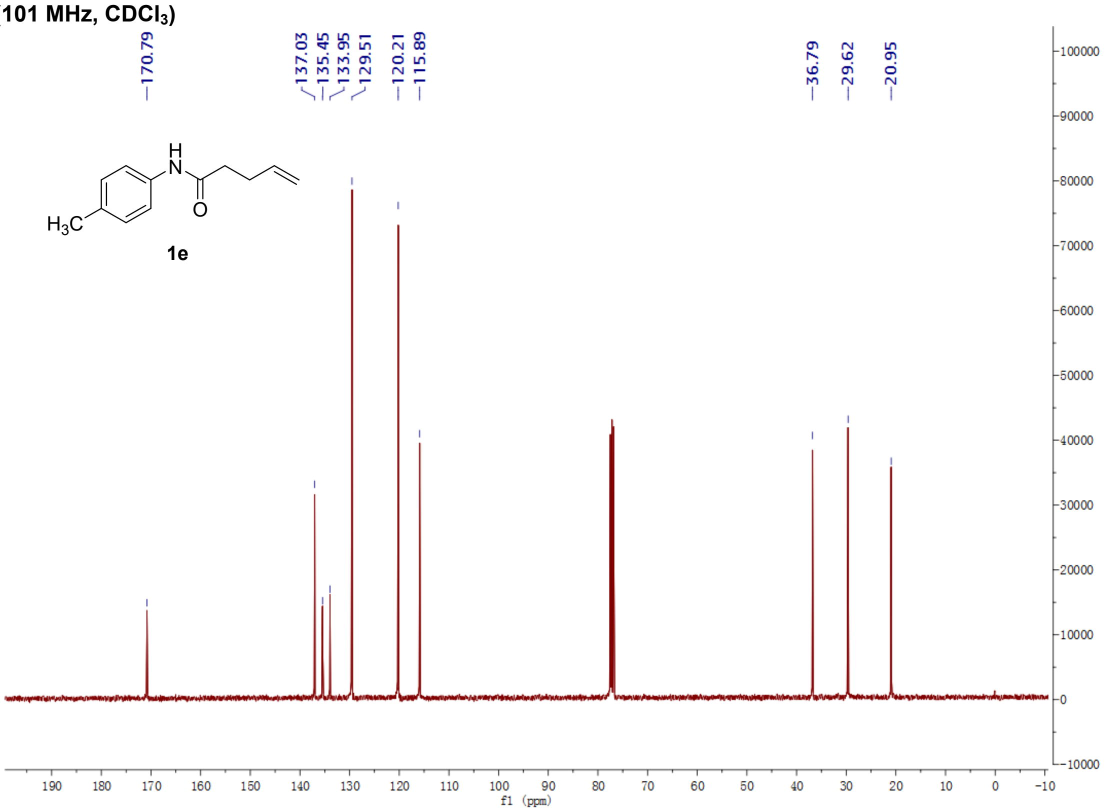

^{1}H-NMR (400 MHz, $CDCl_3$)

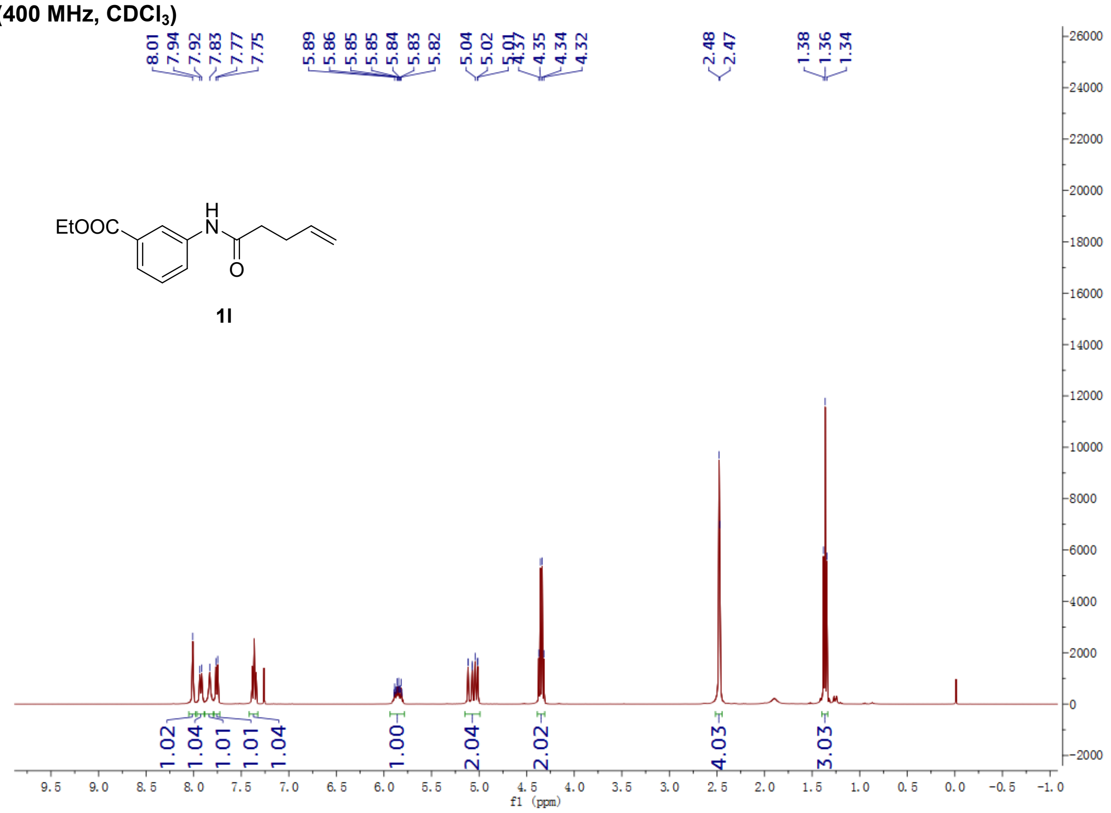

^{13}C-NMR (101 MHz, $CDCl_3$)

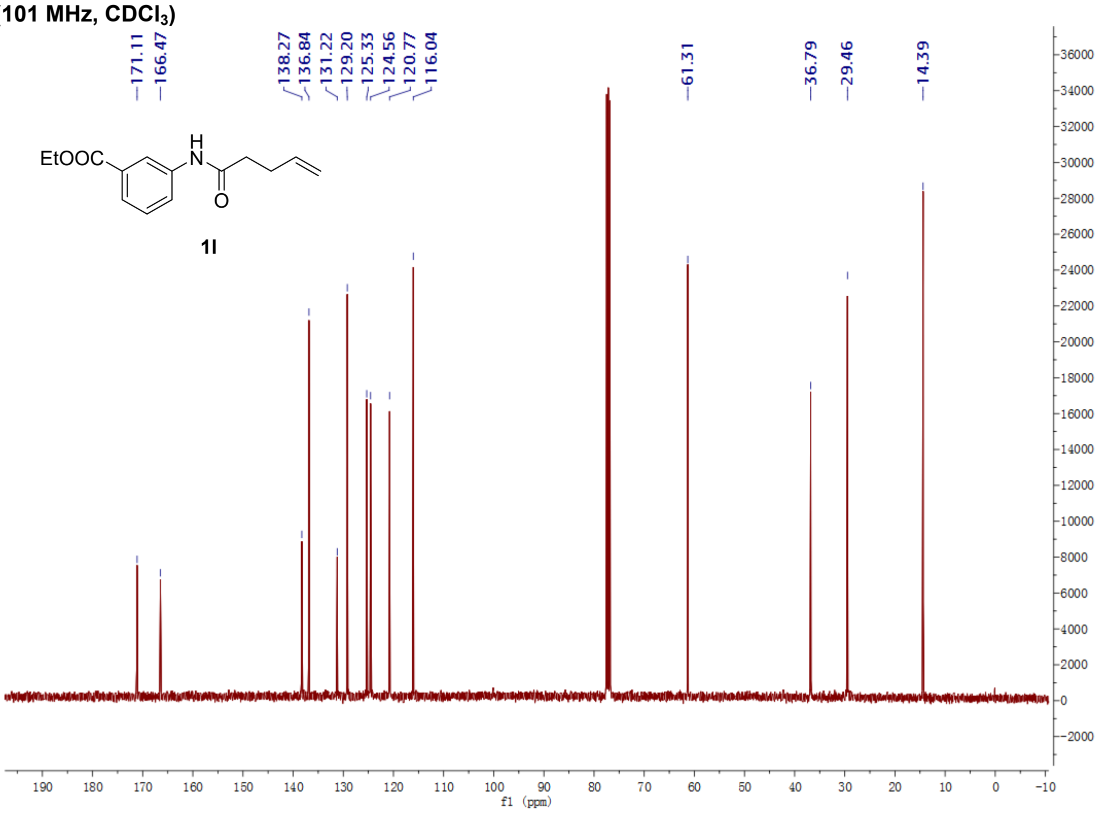

^{1}H-NMR (400 MHz, $CDCl_3$)

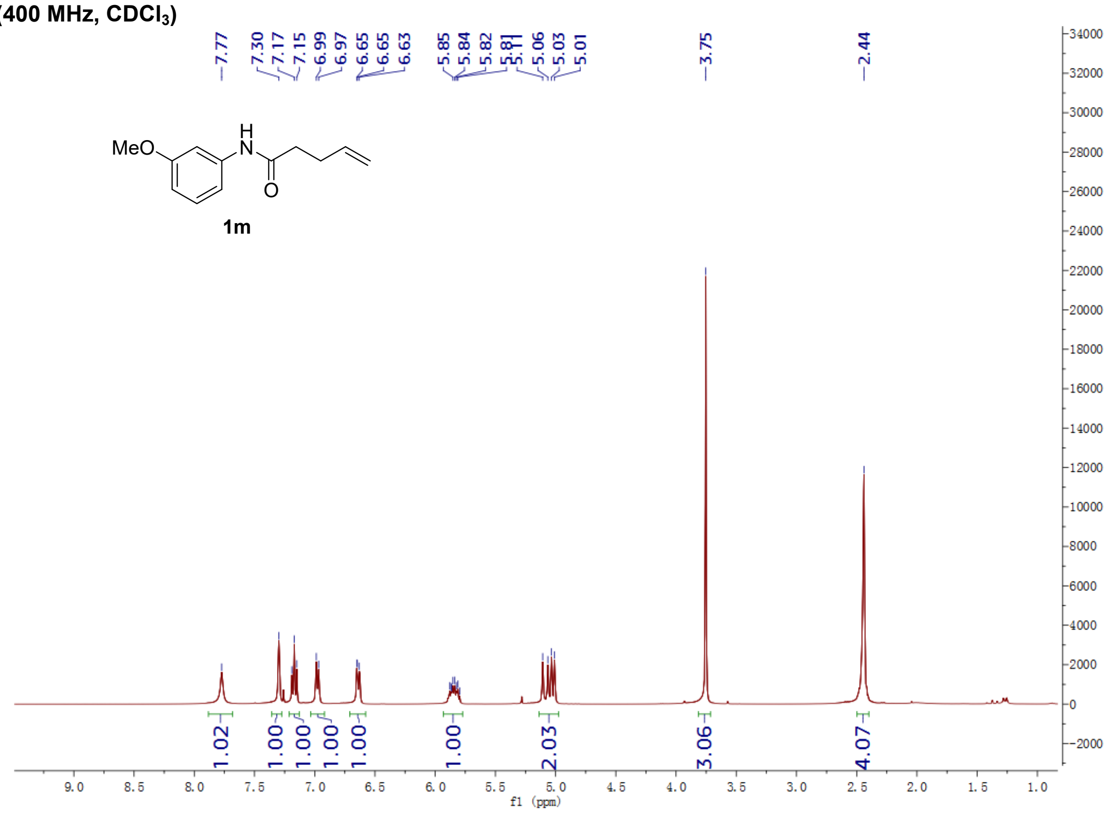

^{13}C-NMR (101 MHz, $CDCl_3$)

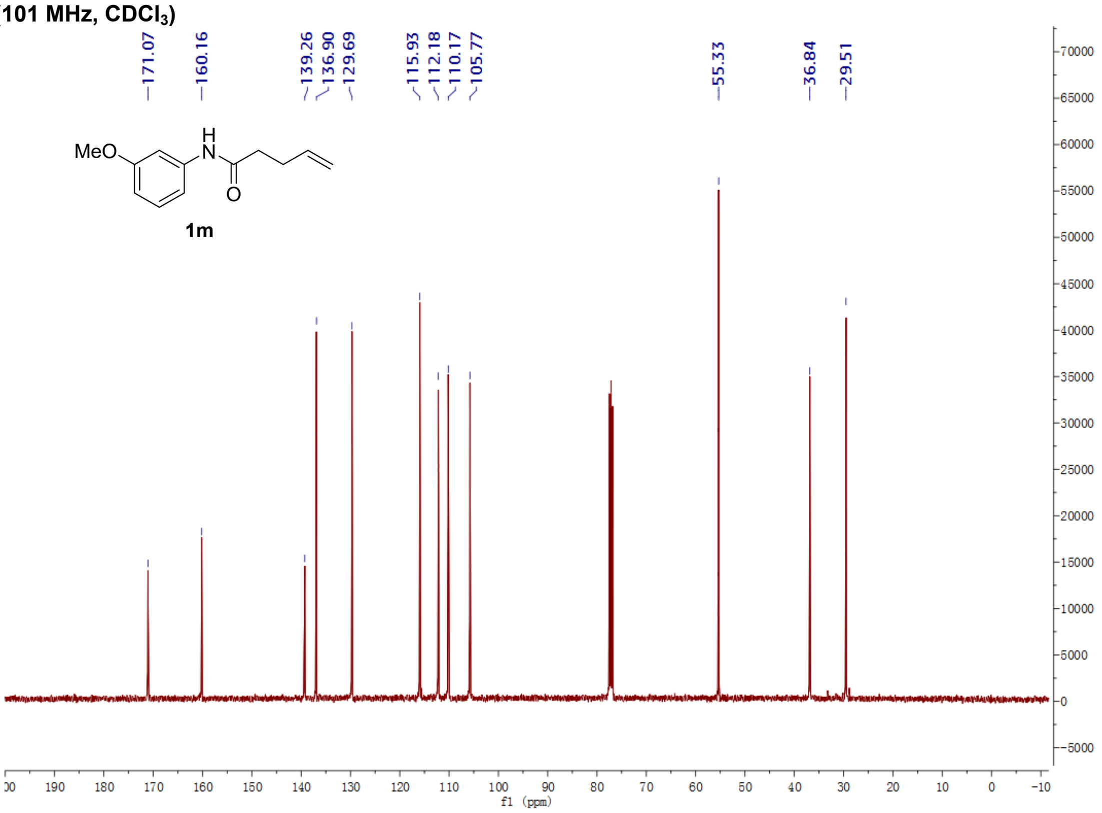

^{1}H-NMR (400 MHz, $CDCl_3$)

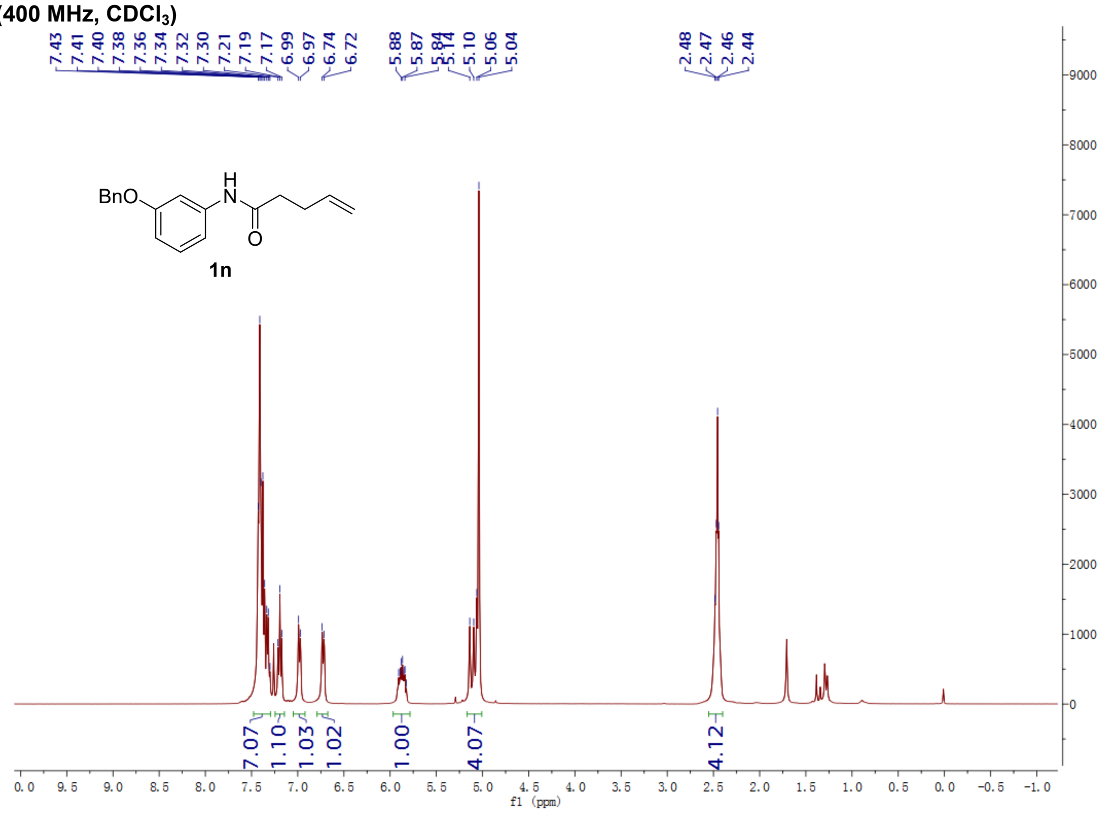

^{13}C-NMR (101 MHz, $CDCl_3$)

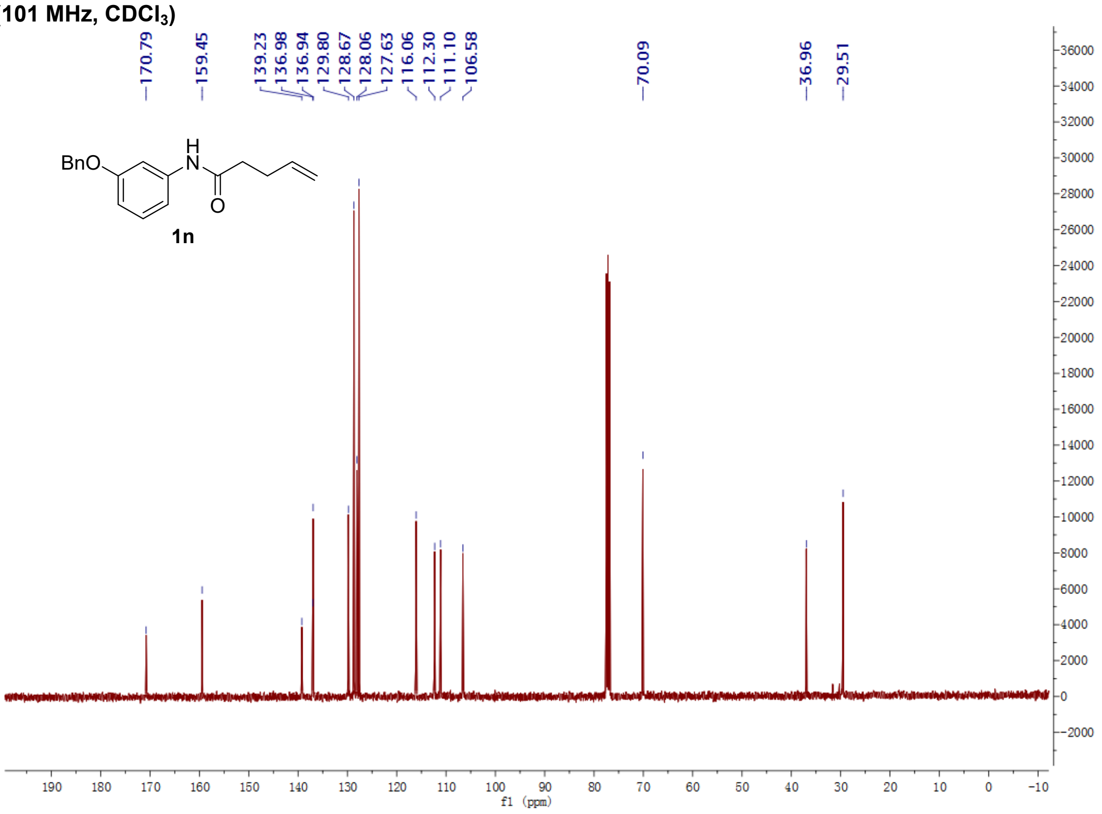

^{1}H-NMR (400 MHz, $CDCl_3$)

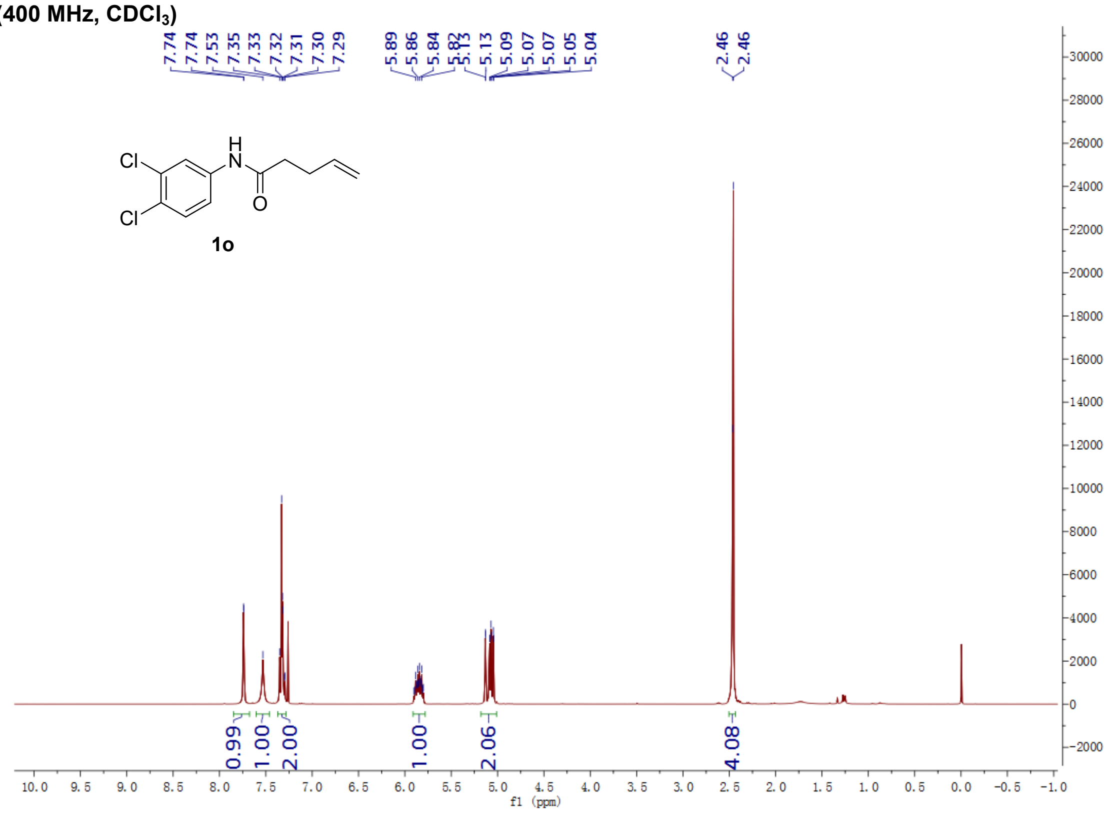

^{13}C-NMR (101 MHz, $CDCl_3$)

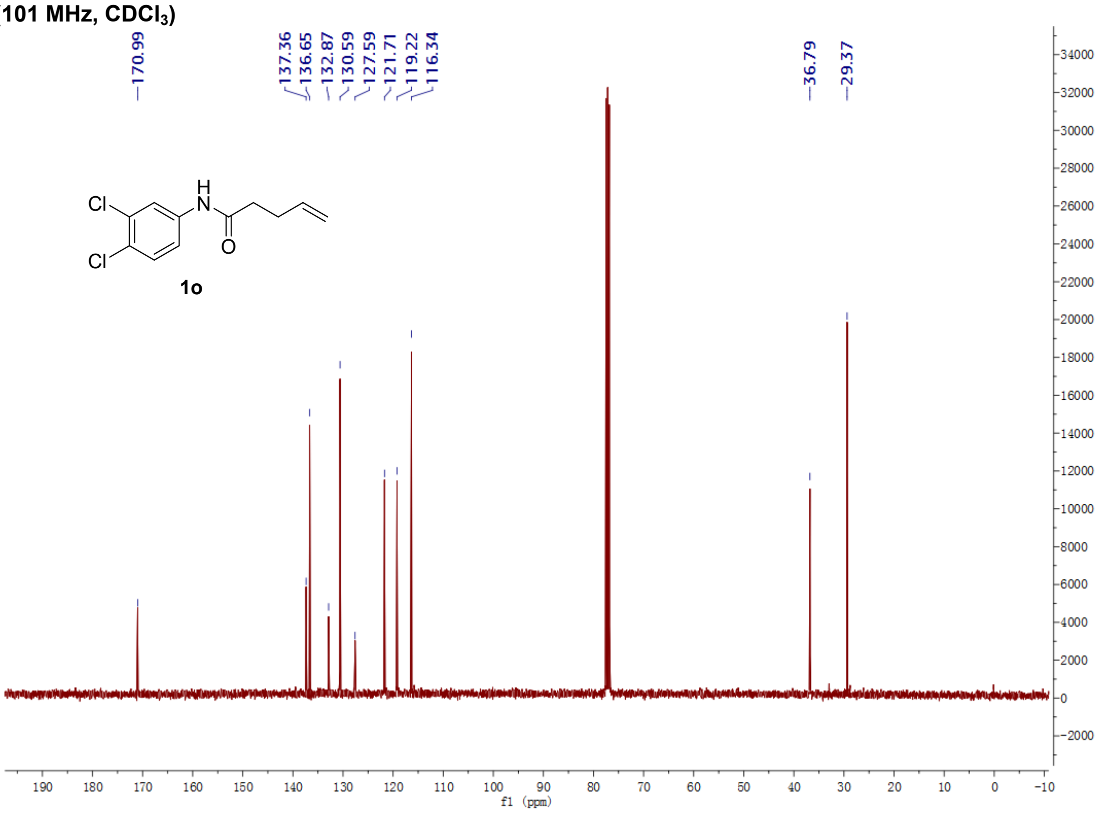

^{1}H-NMR (400 MHz, $CDCl_3$)

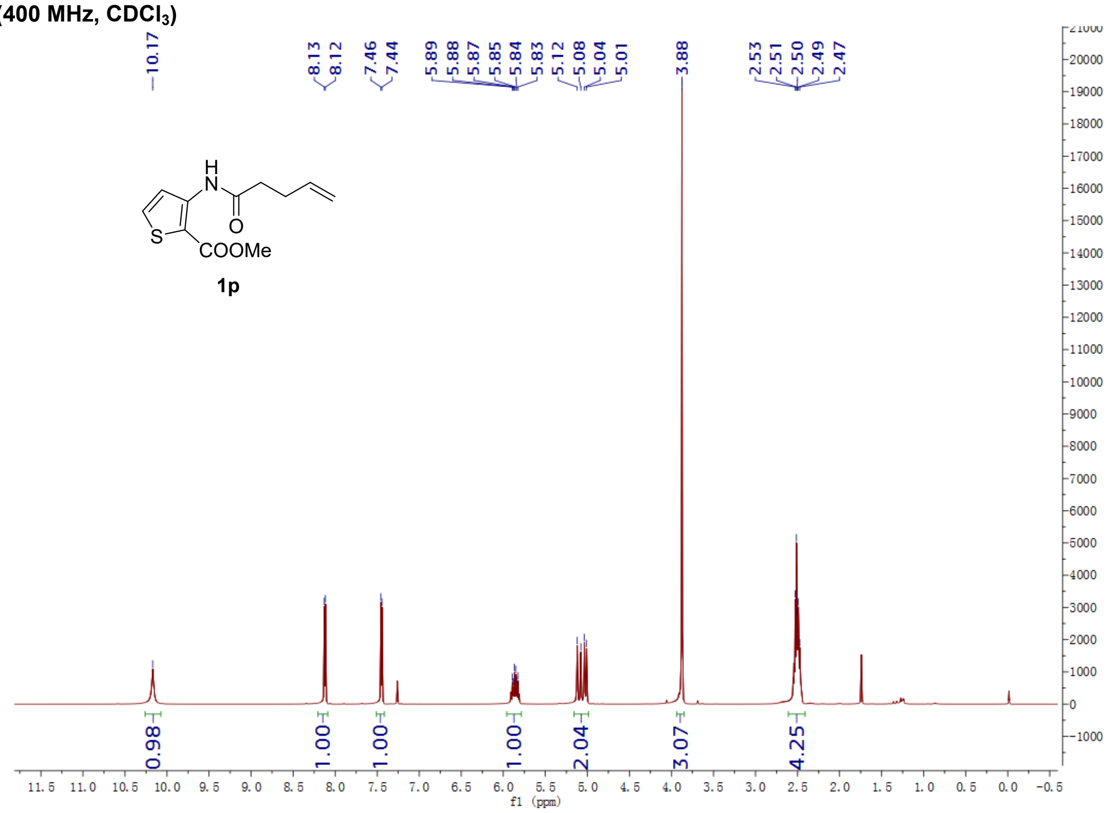

^{13}C-NMR (101 MHz, $CDCl_3$)

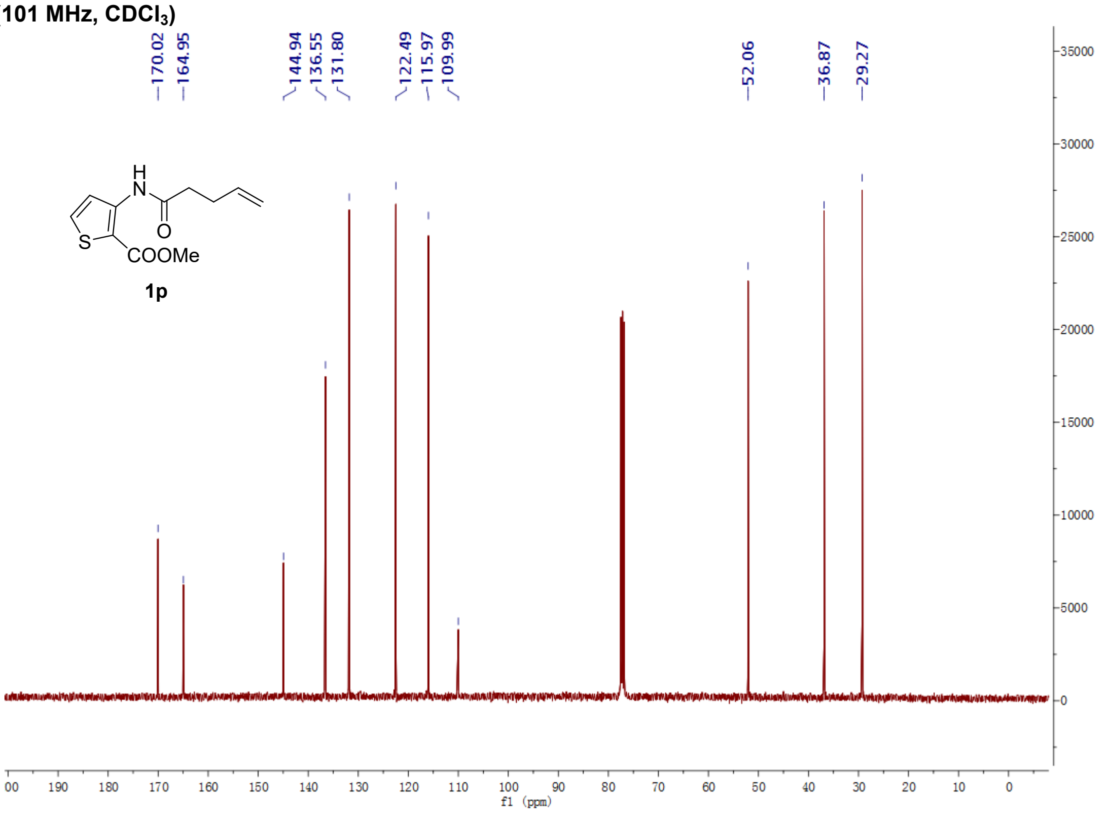

^{1}H-NMR (400 MHz, $CDCl_3$)

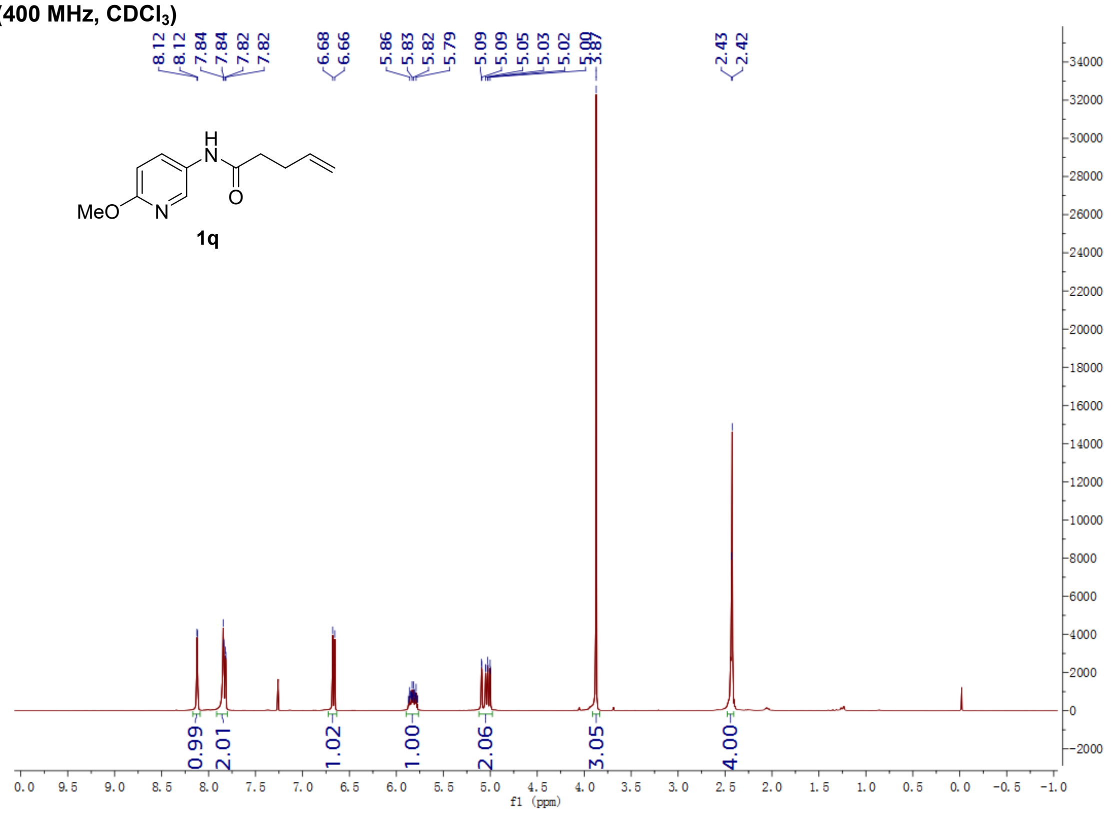

^{13}C-NMR (101 MHz, $CDCl_3$)

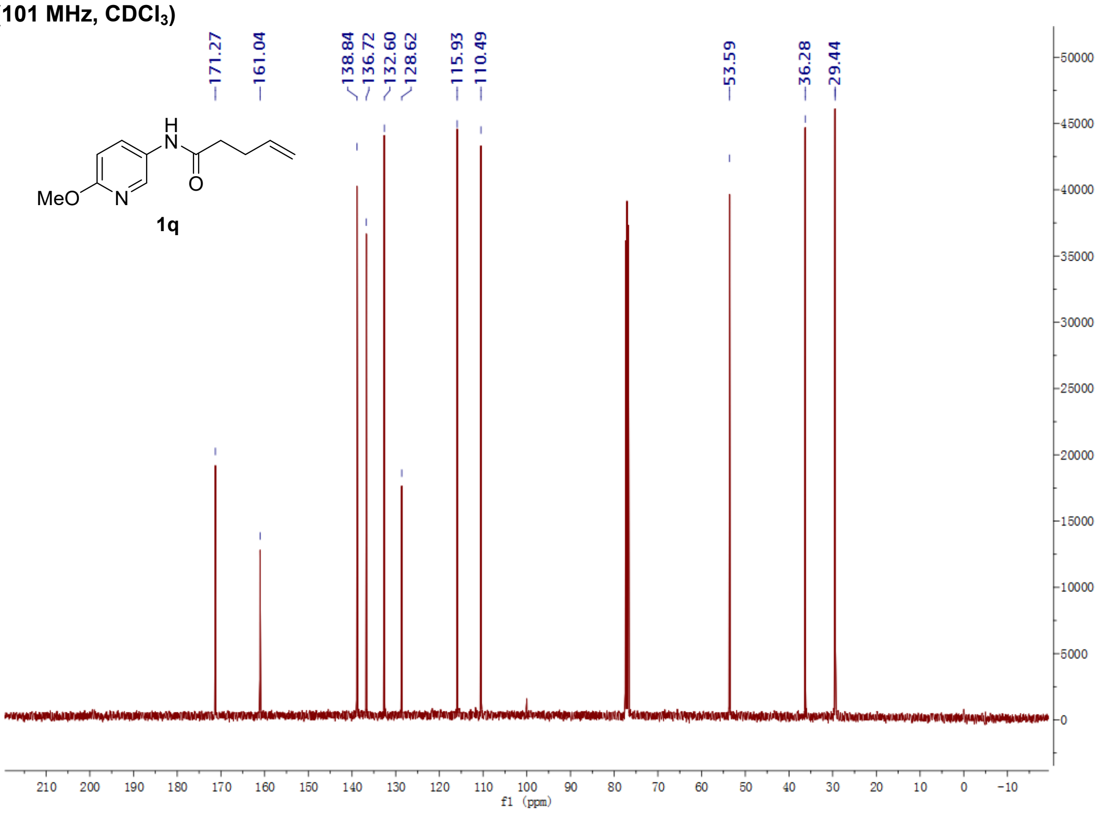

^{1}H-NMR (400 MHz, $CDCl_3$)

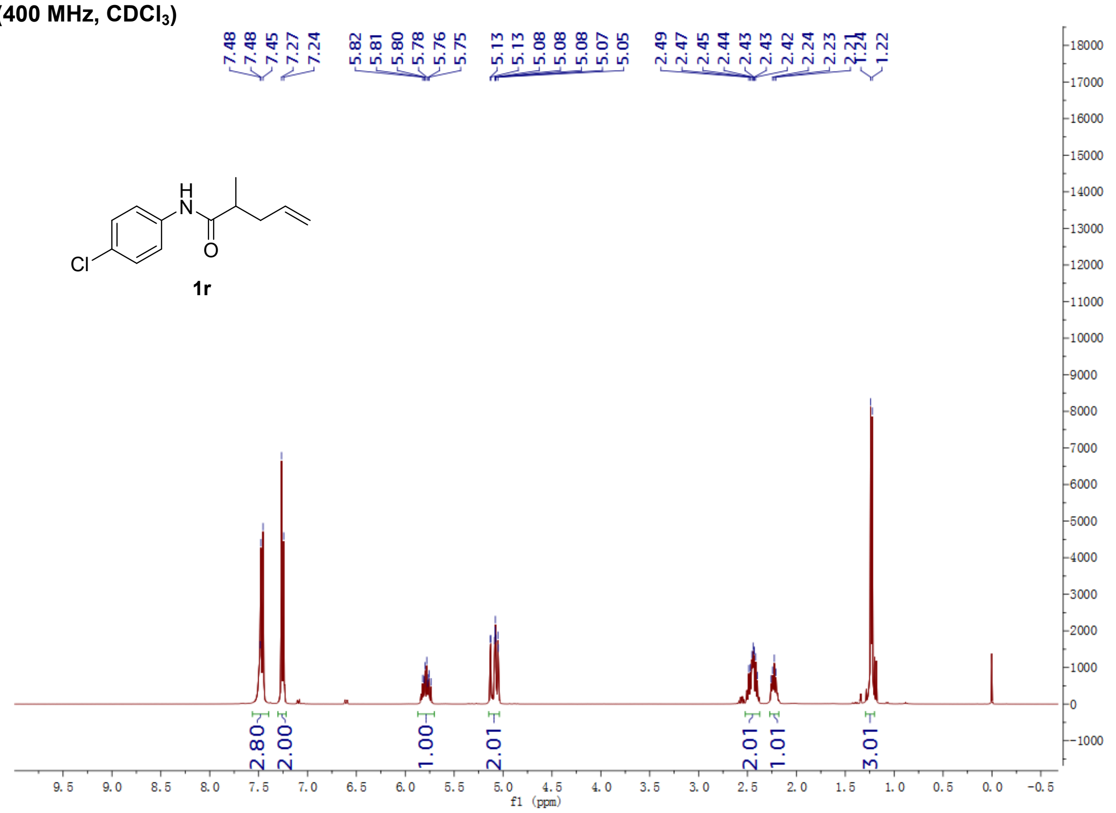

^{13}C-NMR (101 MHz, $CDCl_3$)

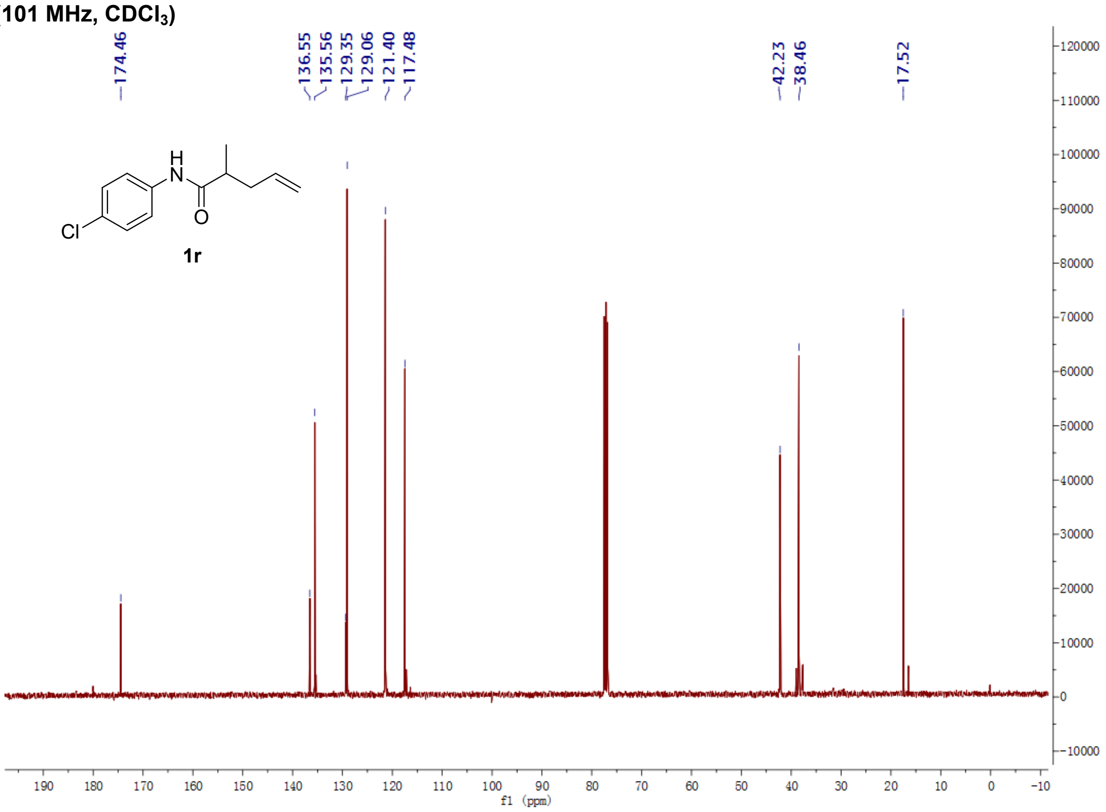

^{1}H-NMR (400 MHz, $CDCl_3$)

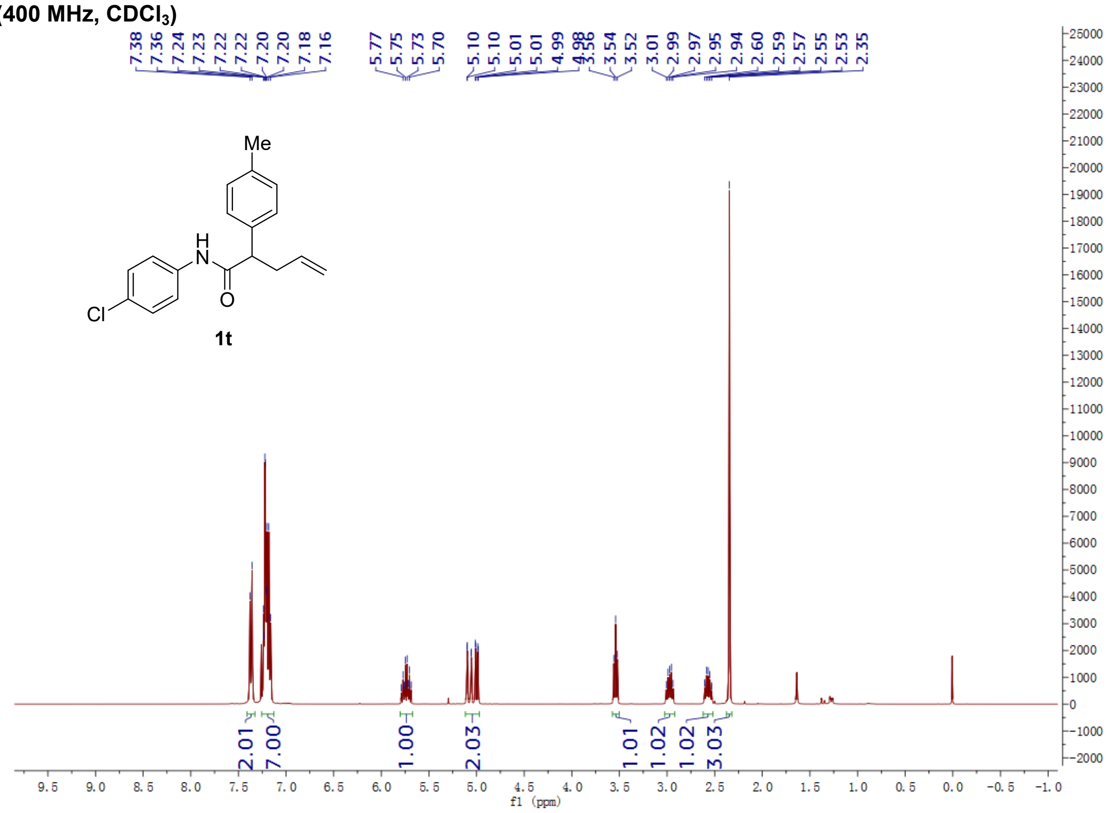

^{13}C-NMR (101 MHz, $CDCl_3$)

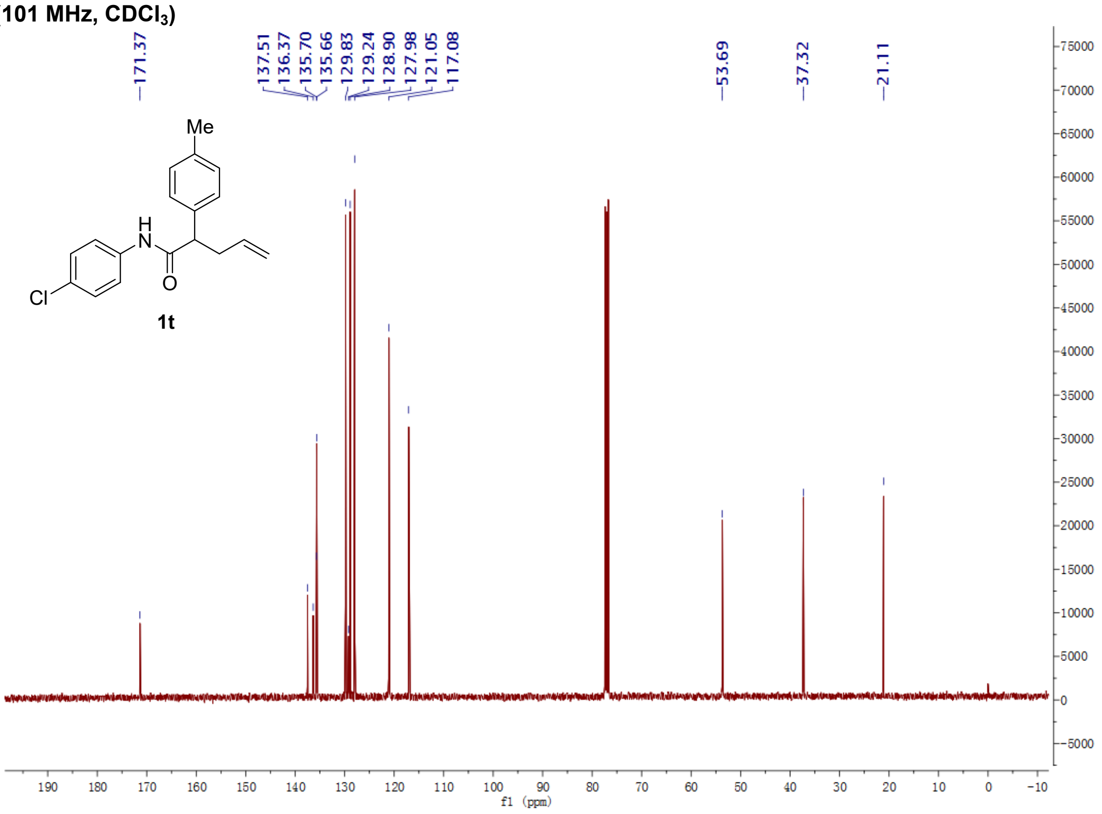

^{1}H-NMR (400 MHz, $CDCl_3$)

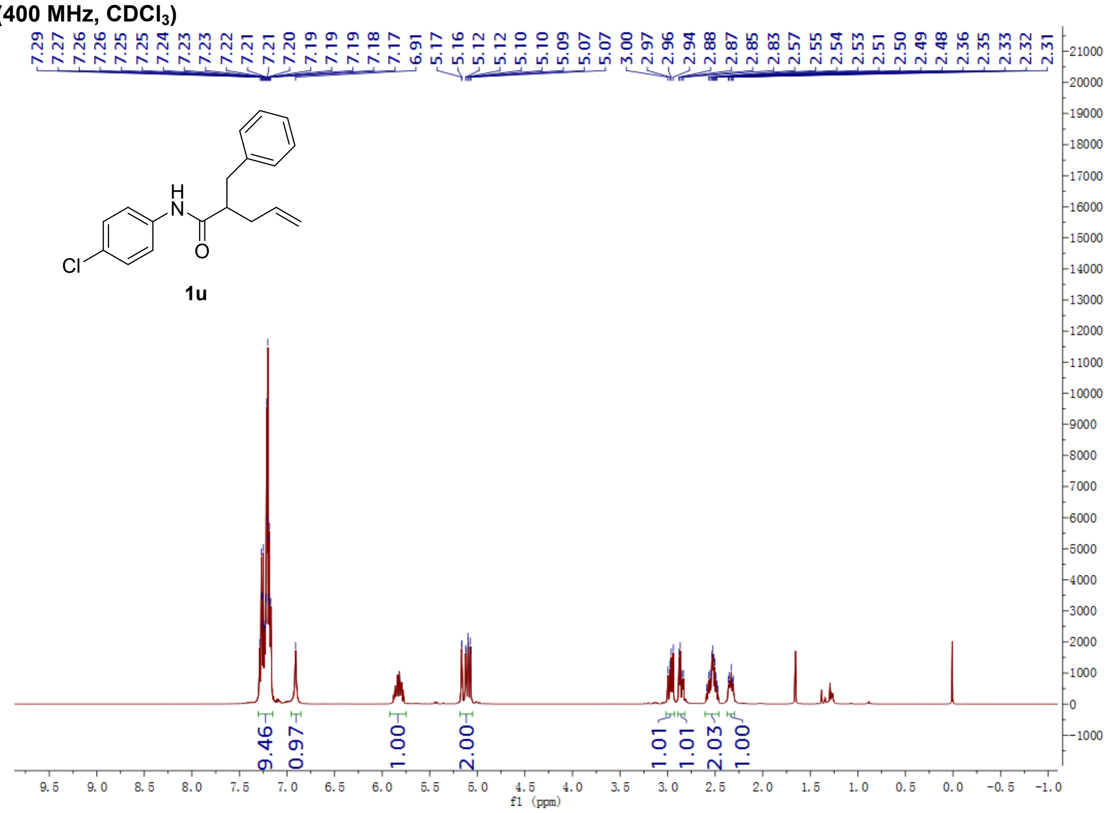

^{13}C-NMR (101 MHz, $CDCl_3$)

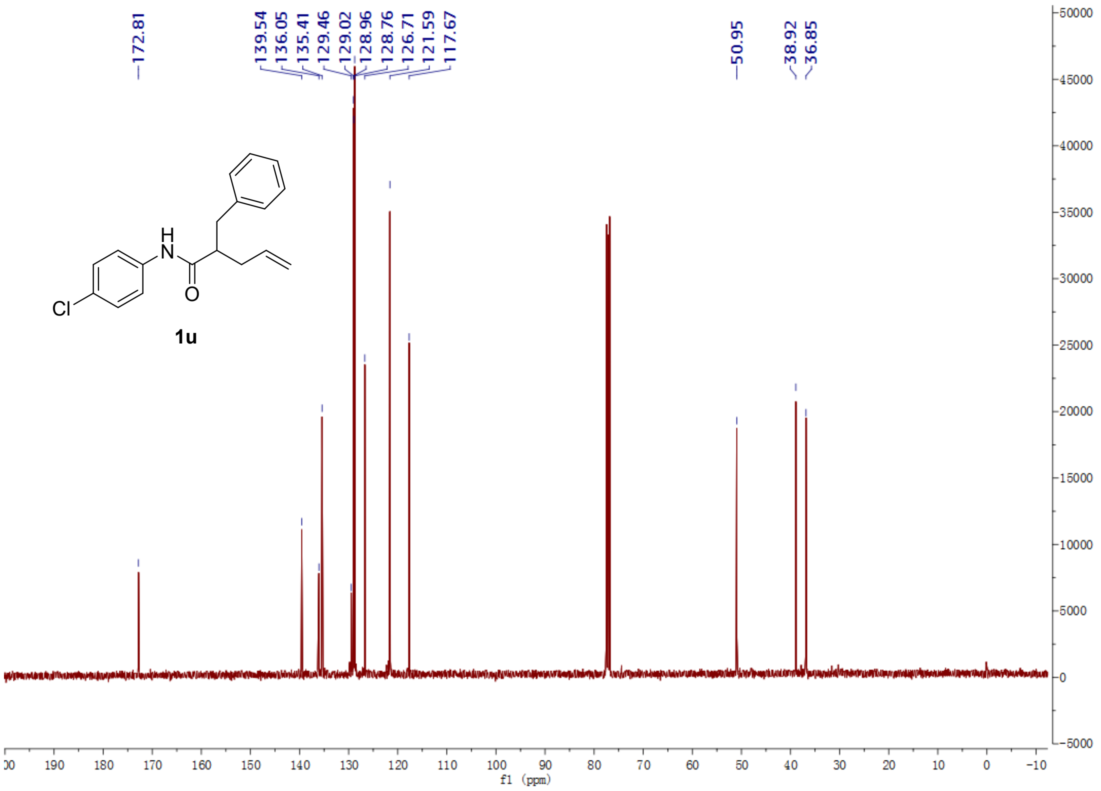

^{1}H-NMR (400 MHz, $CDCl_3$)

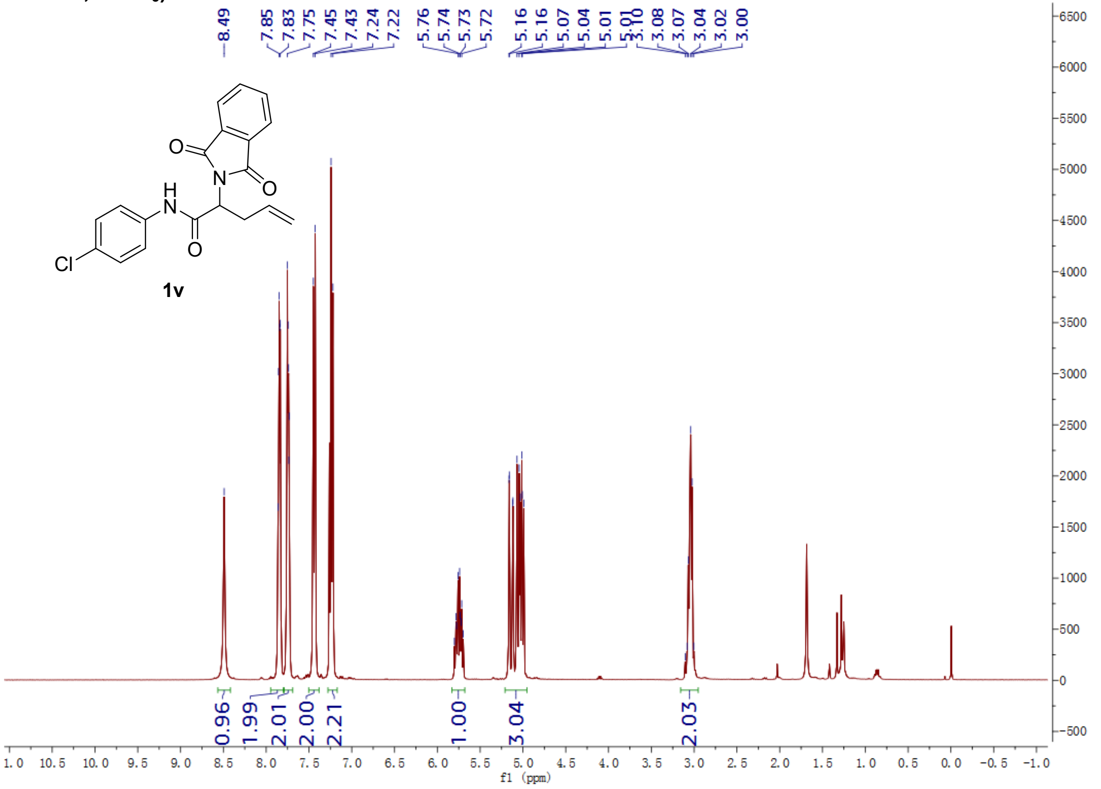

^{13}C-NMR (101 MHz, $CDCl_3$)

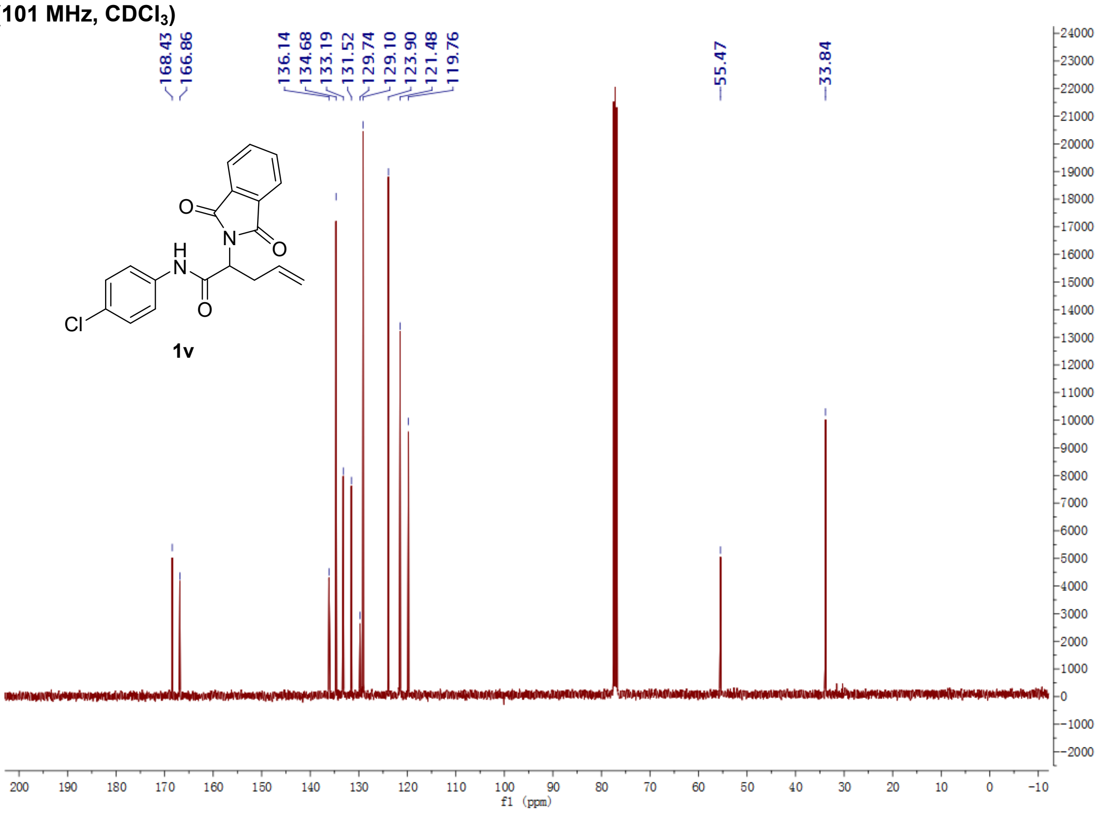

^{1}H-NMR (400 MHz, $CDCl_3$)

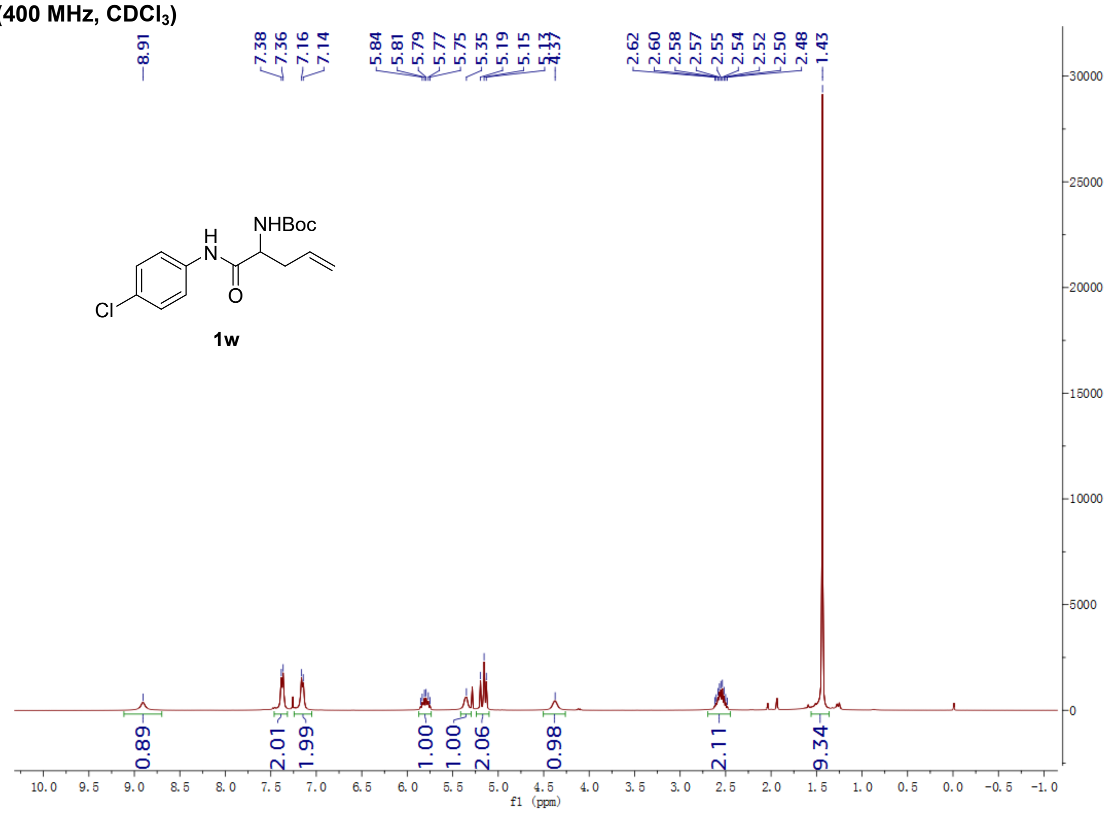

^{13}C-NMR (101 MHz, $CDCl_3$)

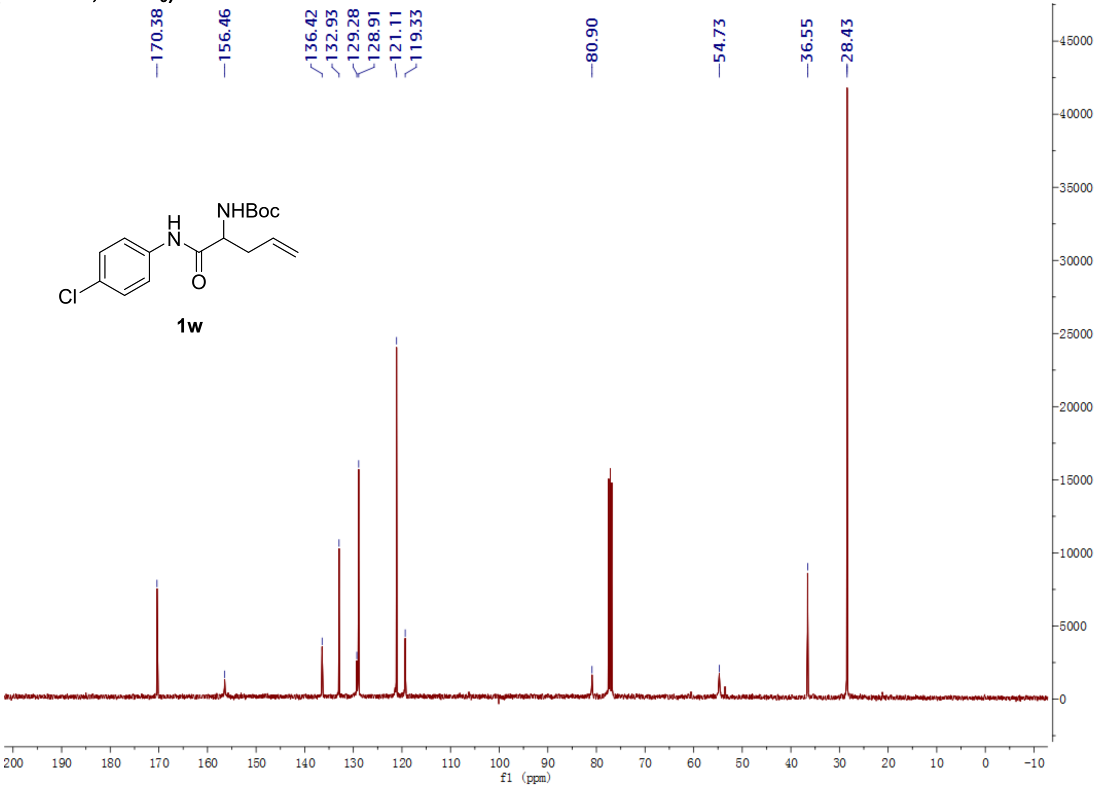

^{1}H-NMR (400 MHz, DMSO-d_6)

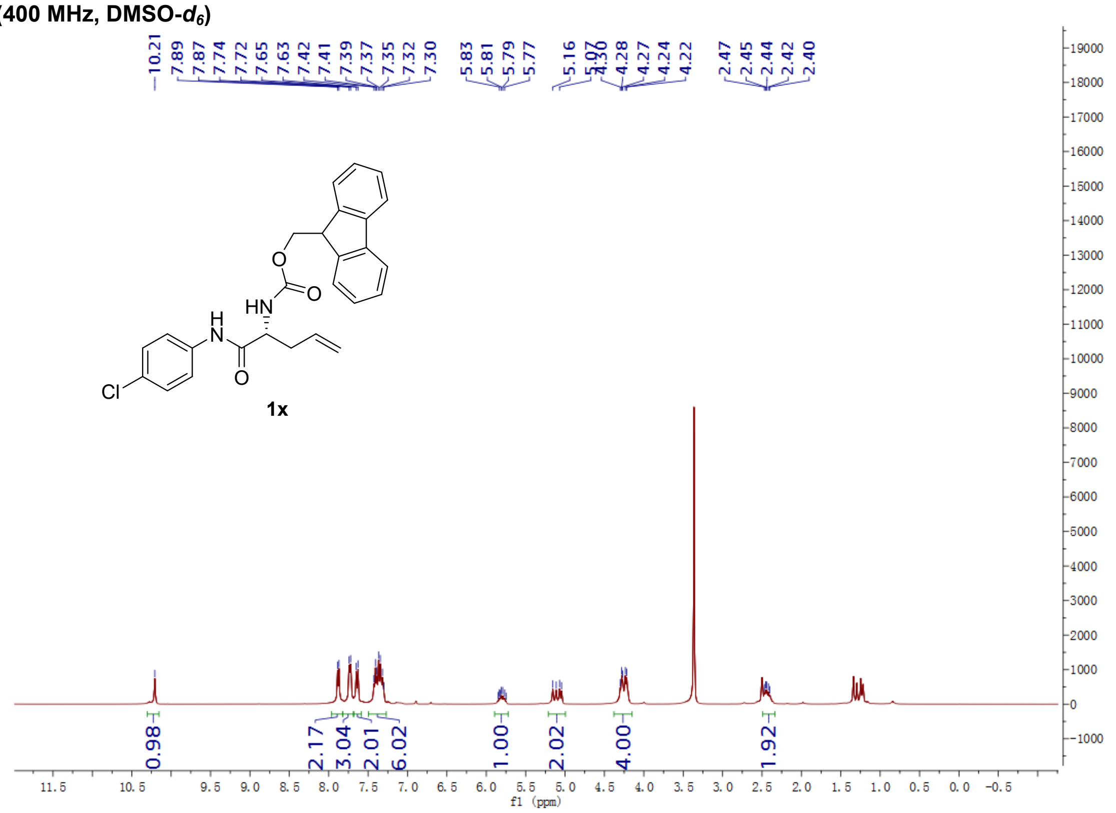

^{13}C-NMR (101 MHz, DMSO-d_6)

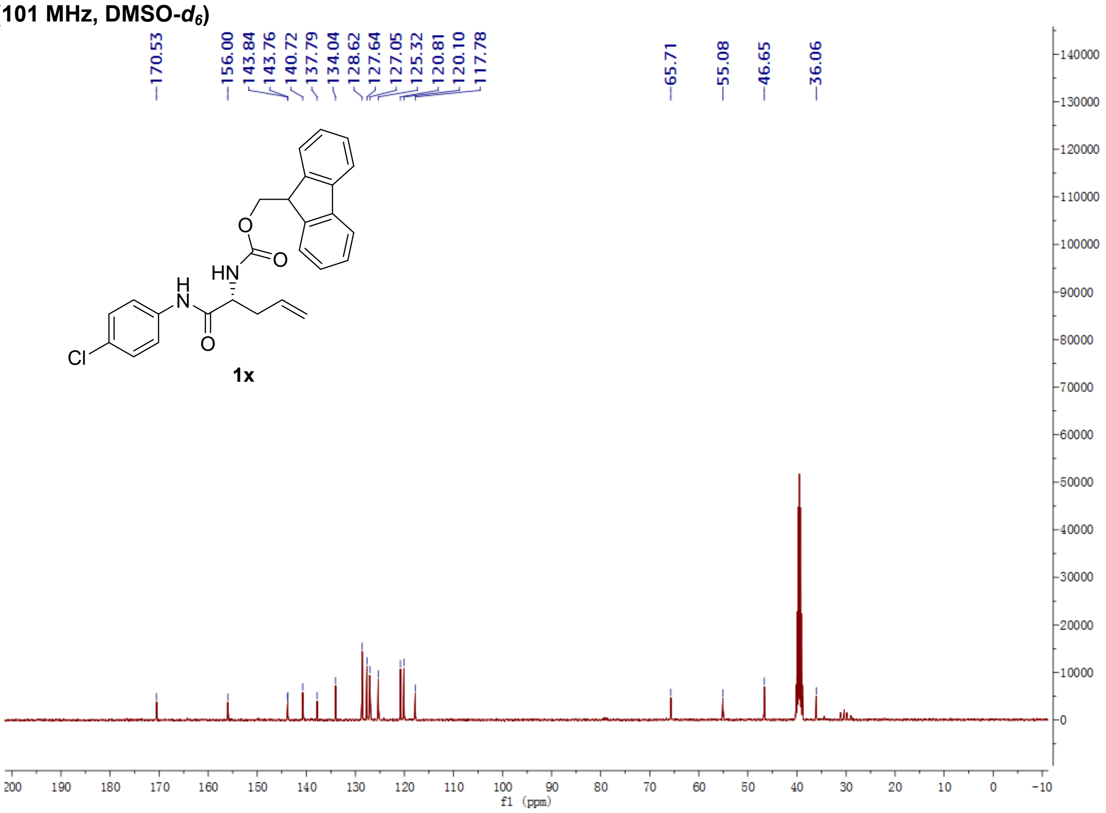

^{1}H-NMR (400 MHz, $CDCl_3$)

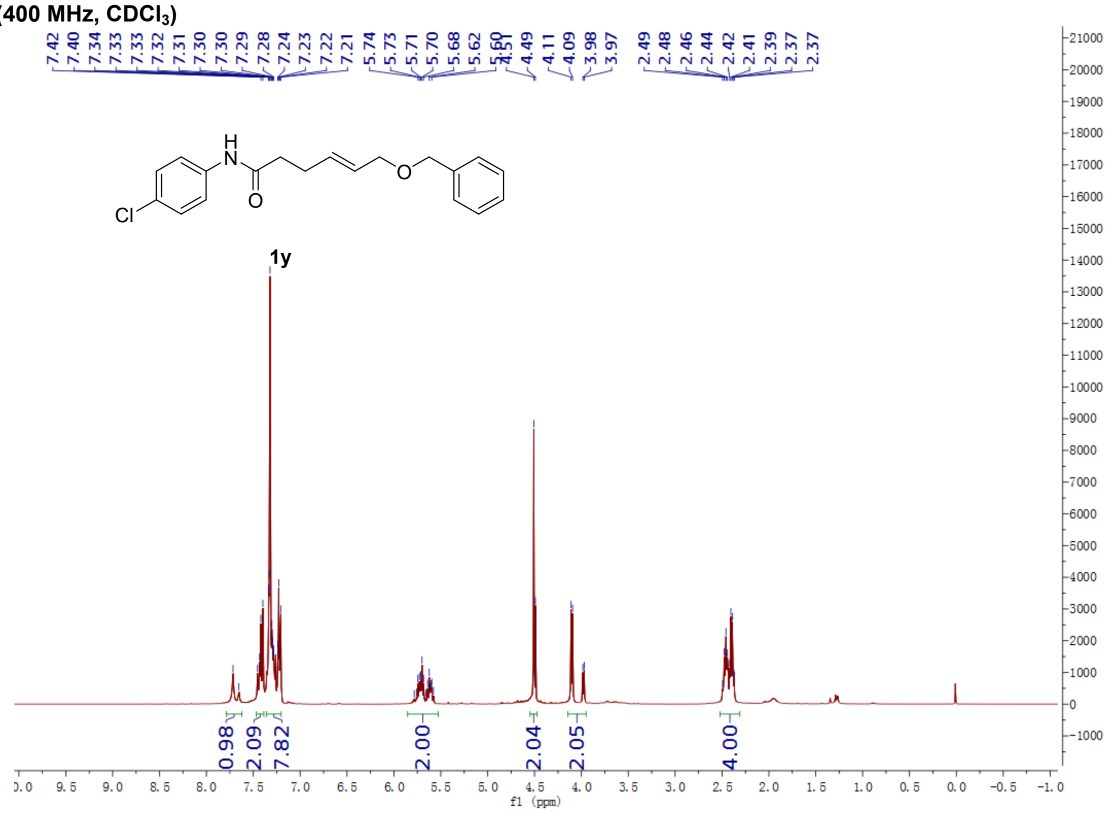

^{13}C-NMR (101 MHz, $CDCl_3$)

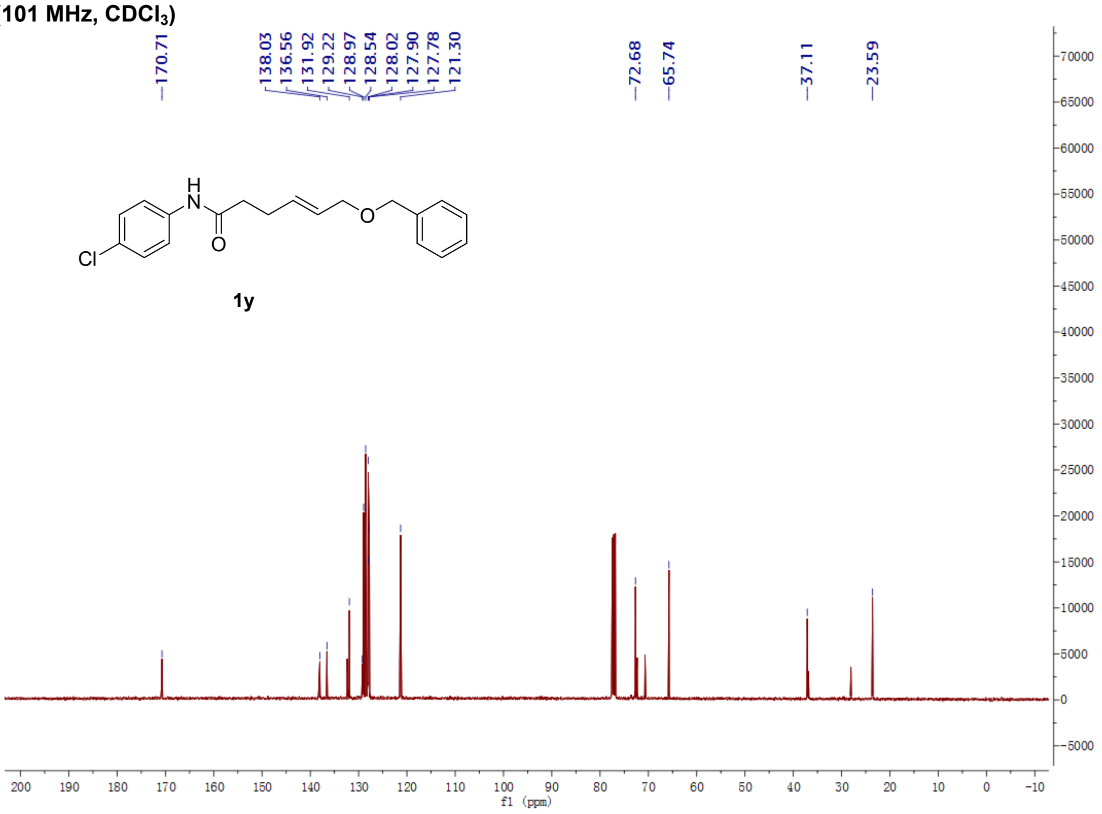

^{1}H-NMR (400 MHz, $CDCl_3$)

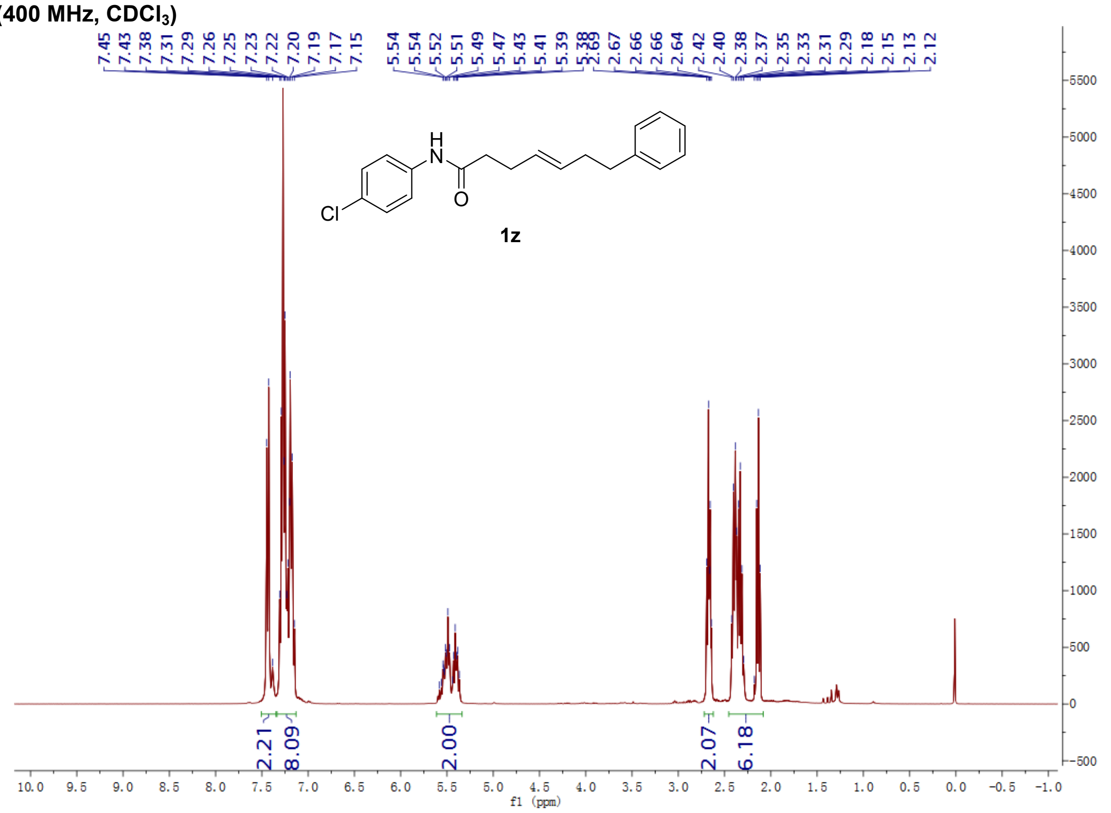

^{13}C-NMR (101 MHz, $CDCl_3$)

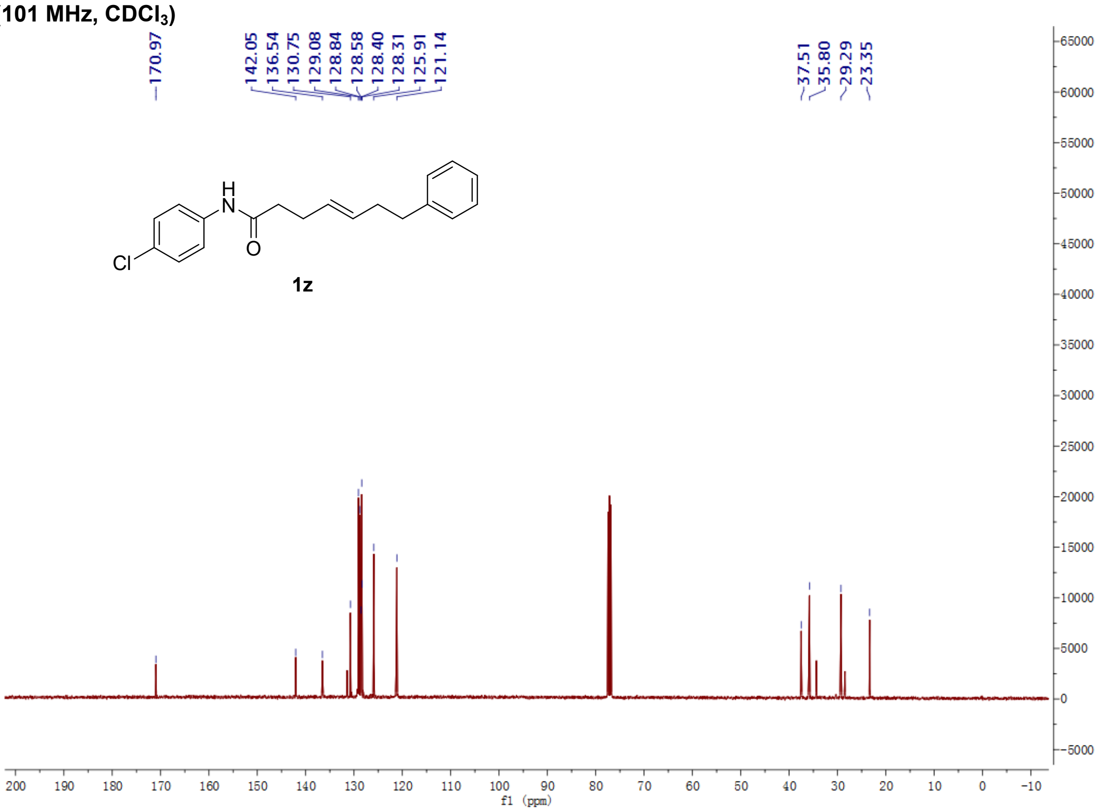

^{1}H-NMR (400 MHz, DMSO-d_6)

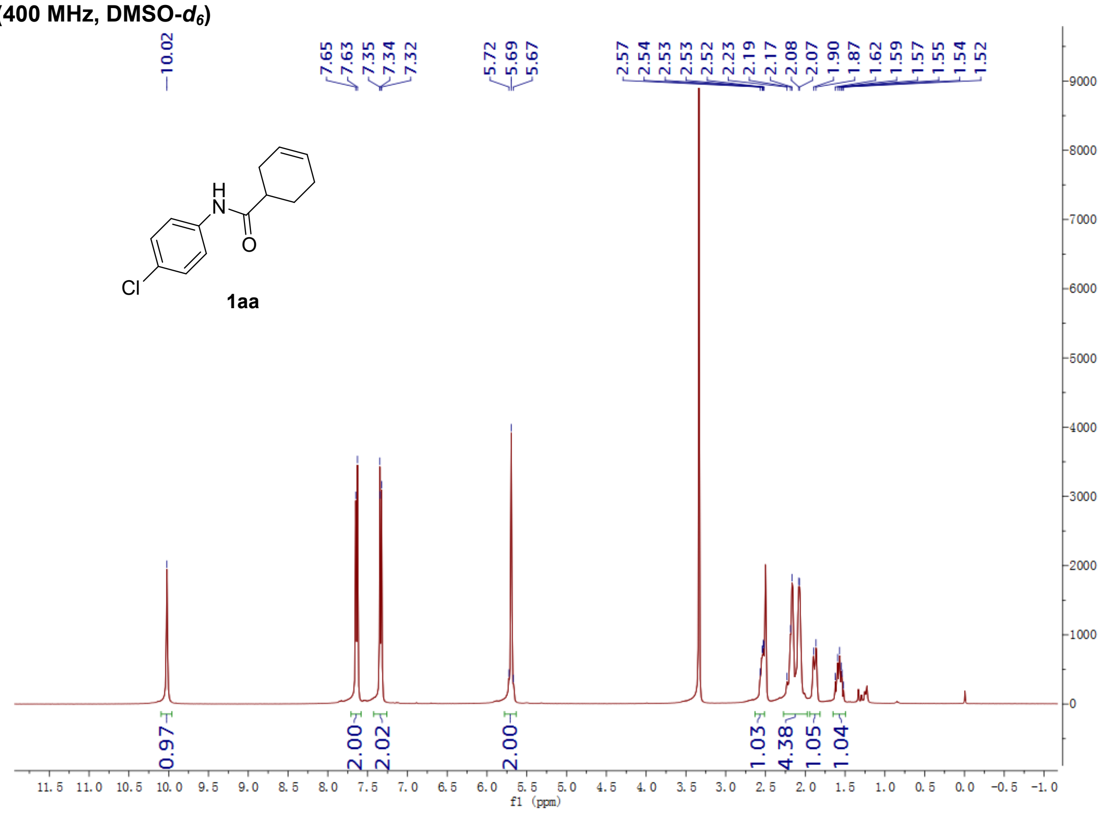

^{13}C-NMR (101 MHz, DMSO-d_6)

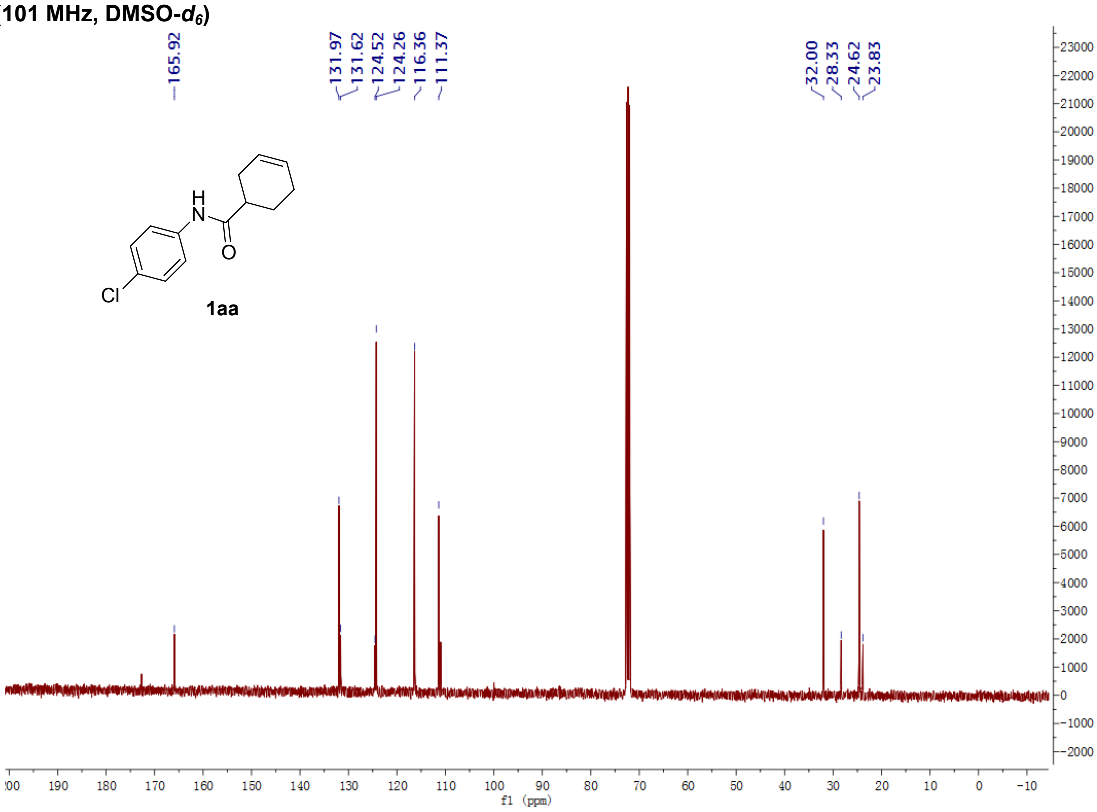

^{1}H-NMR (400 MHz, DMSO-d_6)

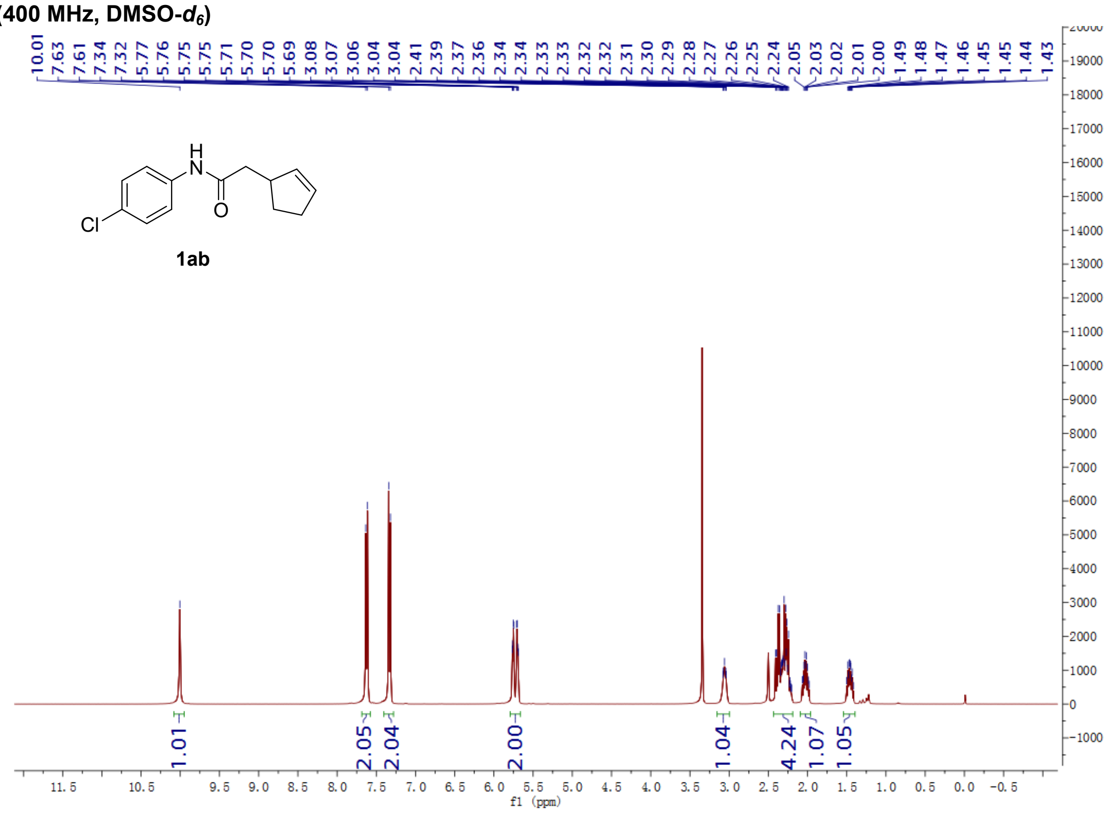

^{13}C-NMR (101 MHz, DMSO-d_6)

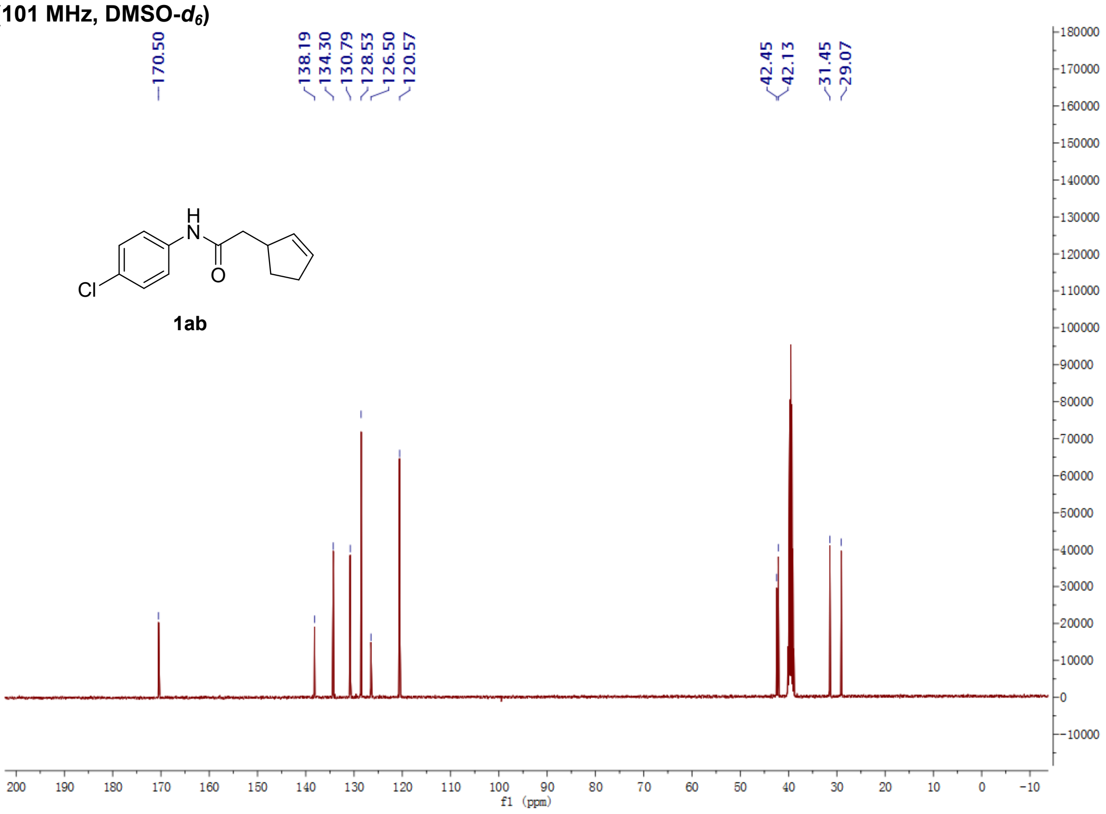

^{1}H-NMR (400 MHz, DMSO-d_6)

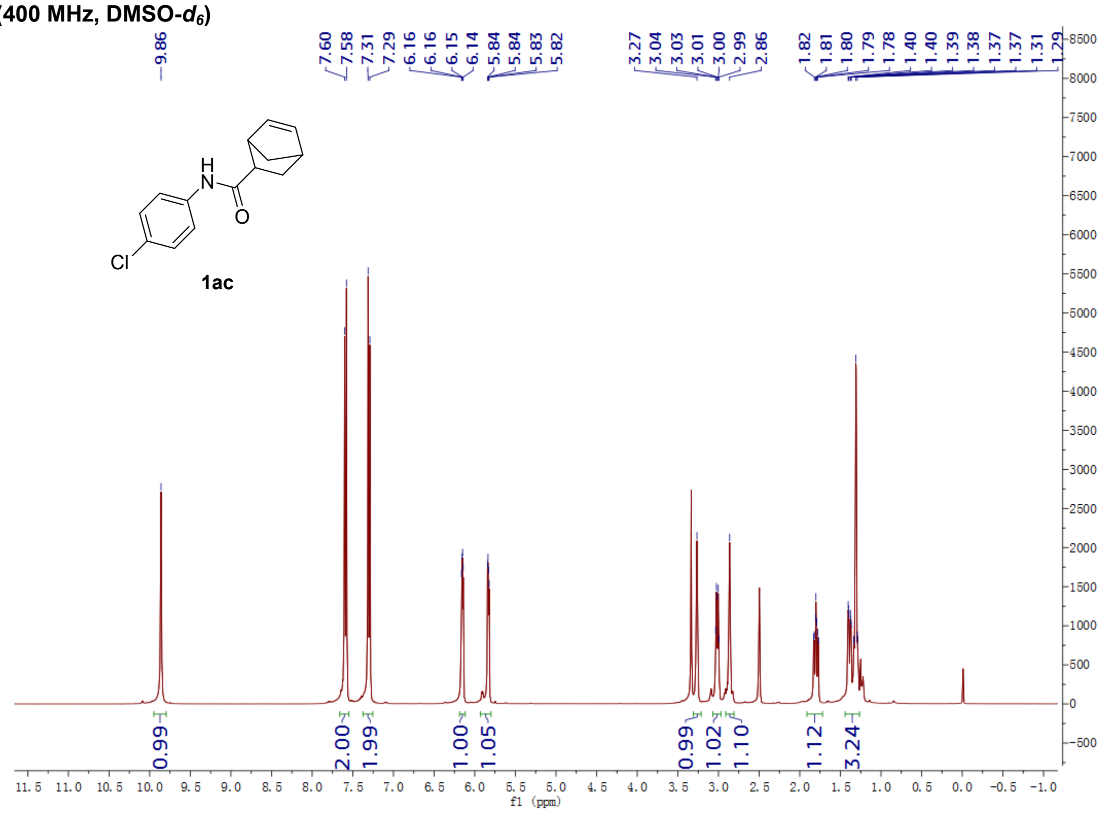

^{13}C-NMR (101 MHz, DMSO-d_6)

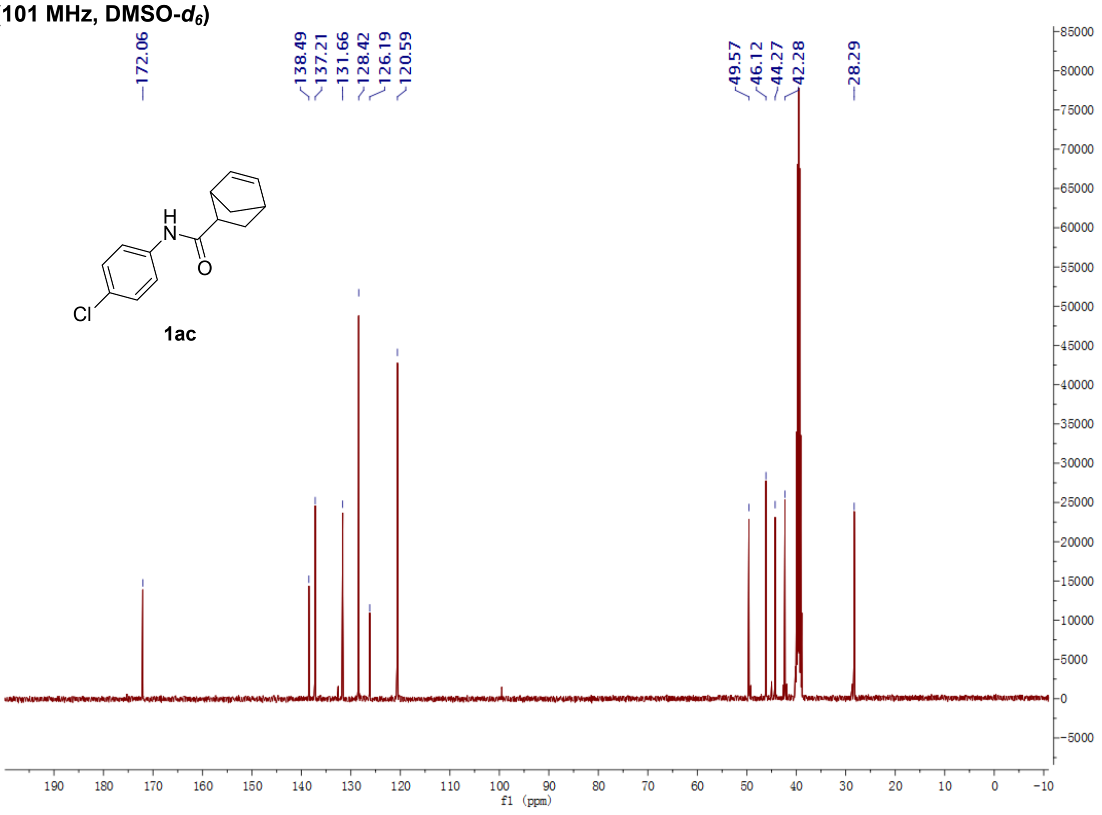

^{1}H-NMR (400 MHz, DMSO-d_6)

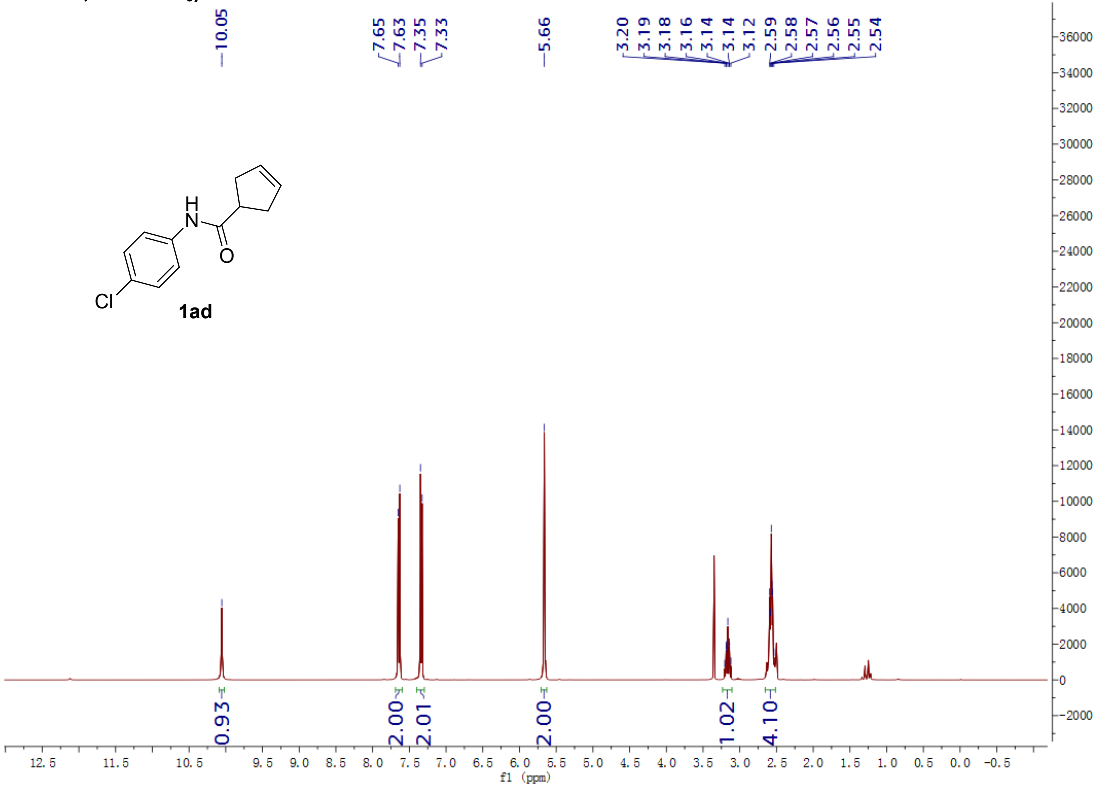

^{13}C-NMR (101 MHz, DMSO-d_6)

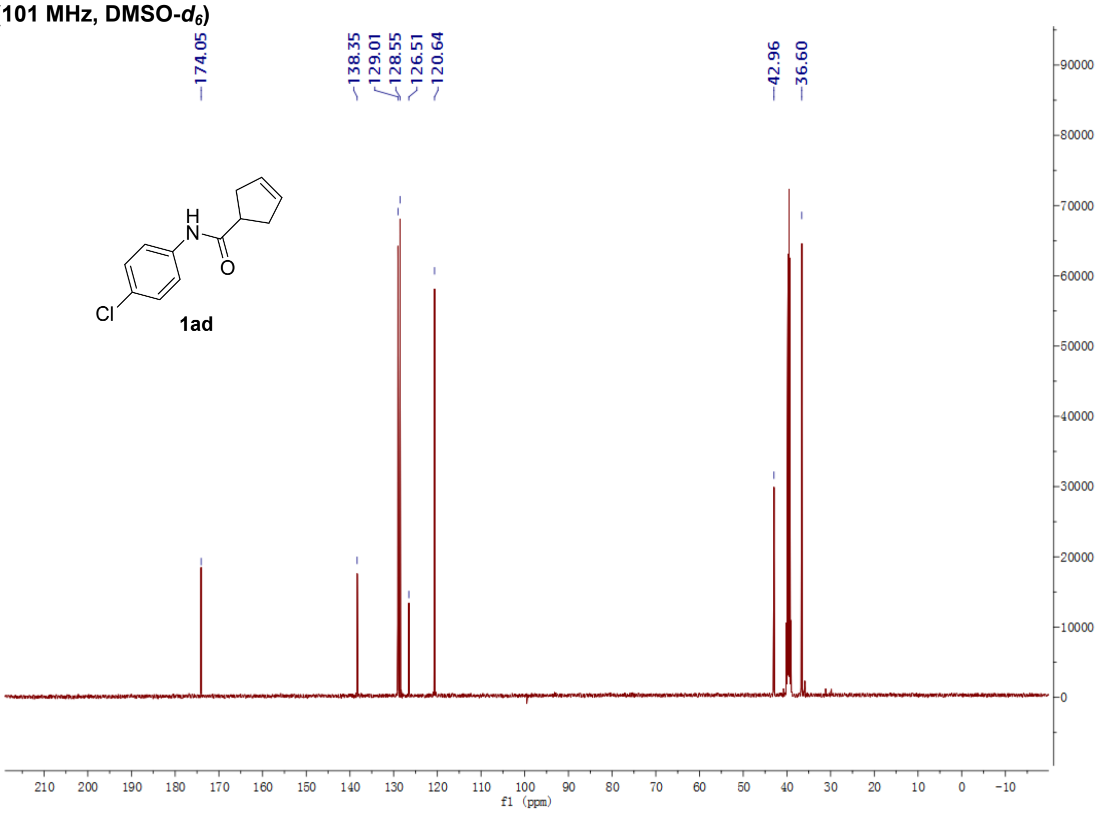

^{1}H-NMR (400 MHz, $CDCl_3$)

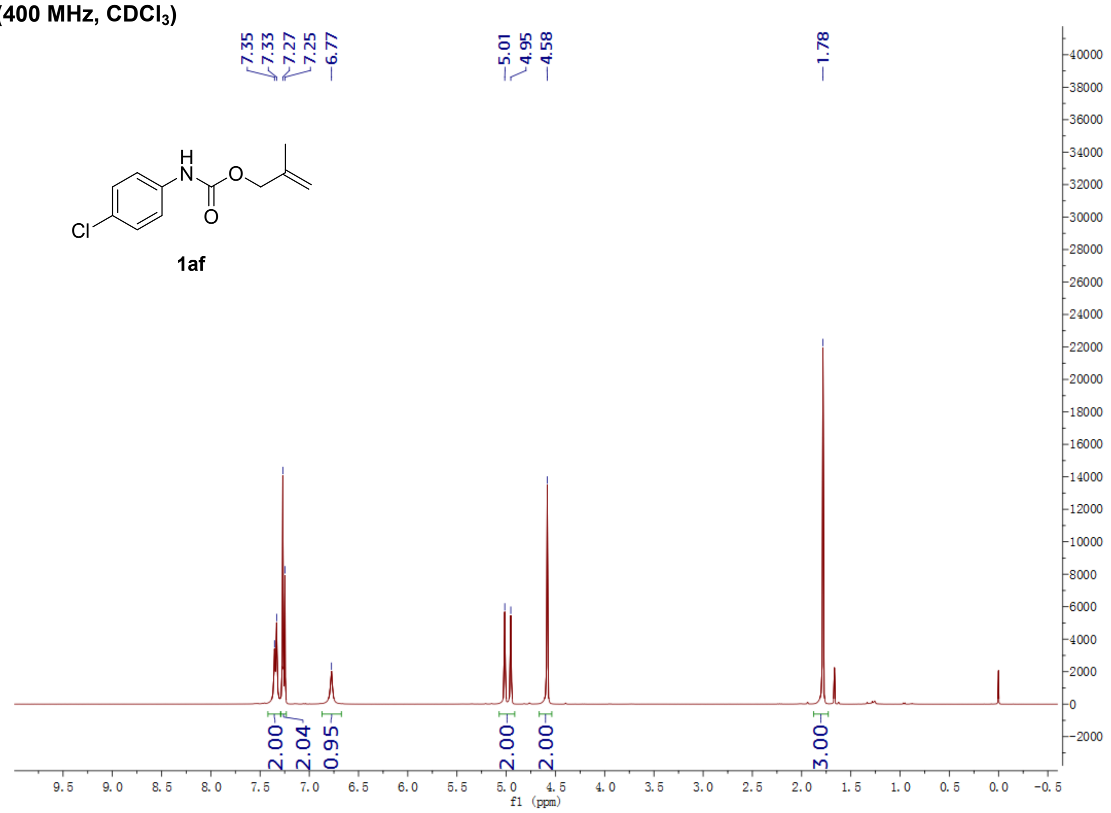

^{13}C-NMR (101 MHz, $CDCl_3$)

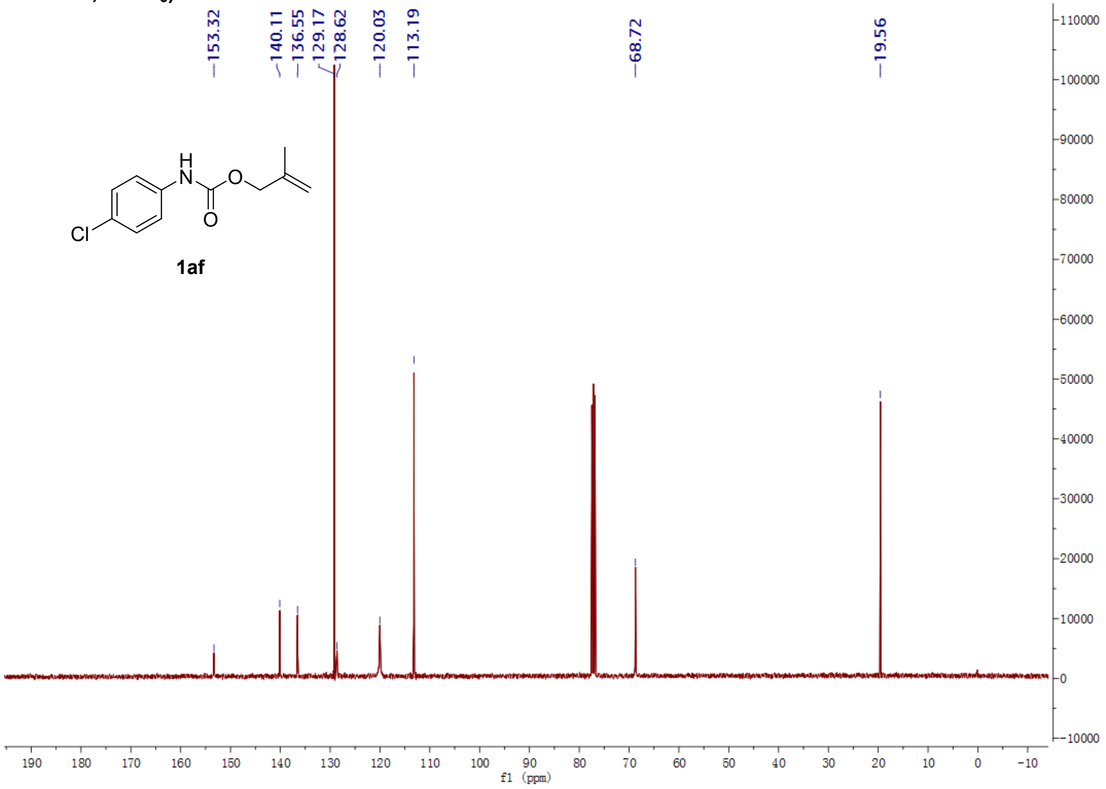

^{1}H-NMR (400 MHz, $CDCl_3$)

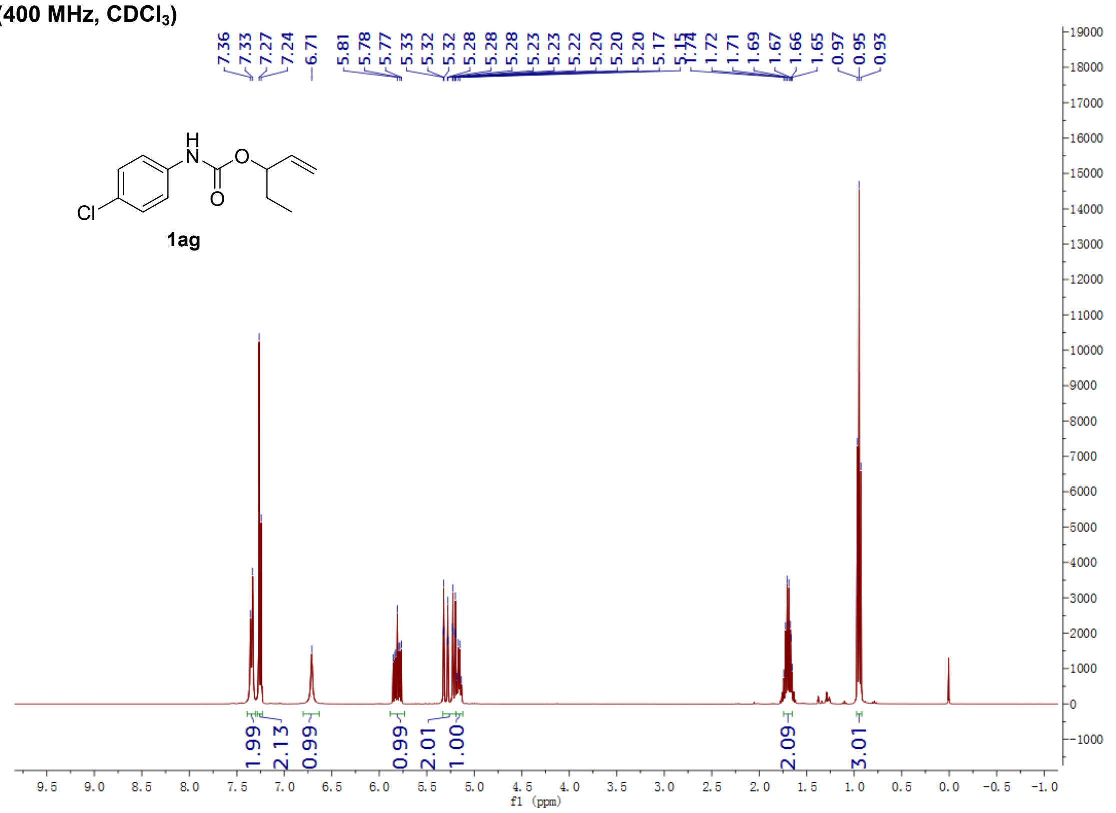

^{13}C-NMR (101 MHz, $CDCl_3$)

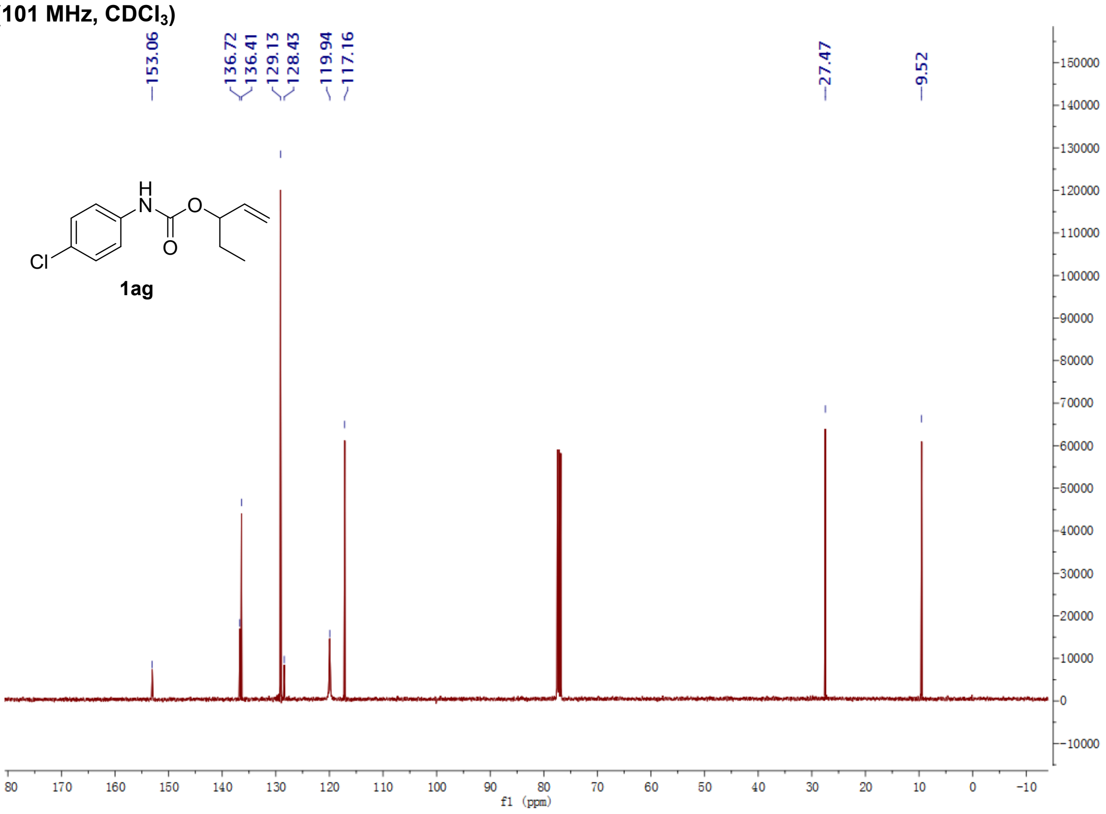

^{1}H-NMR (400 MHz, $CDCl_3$)

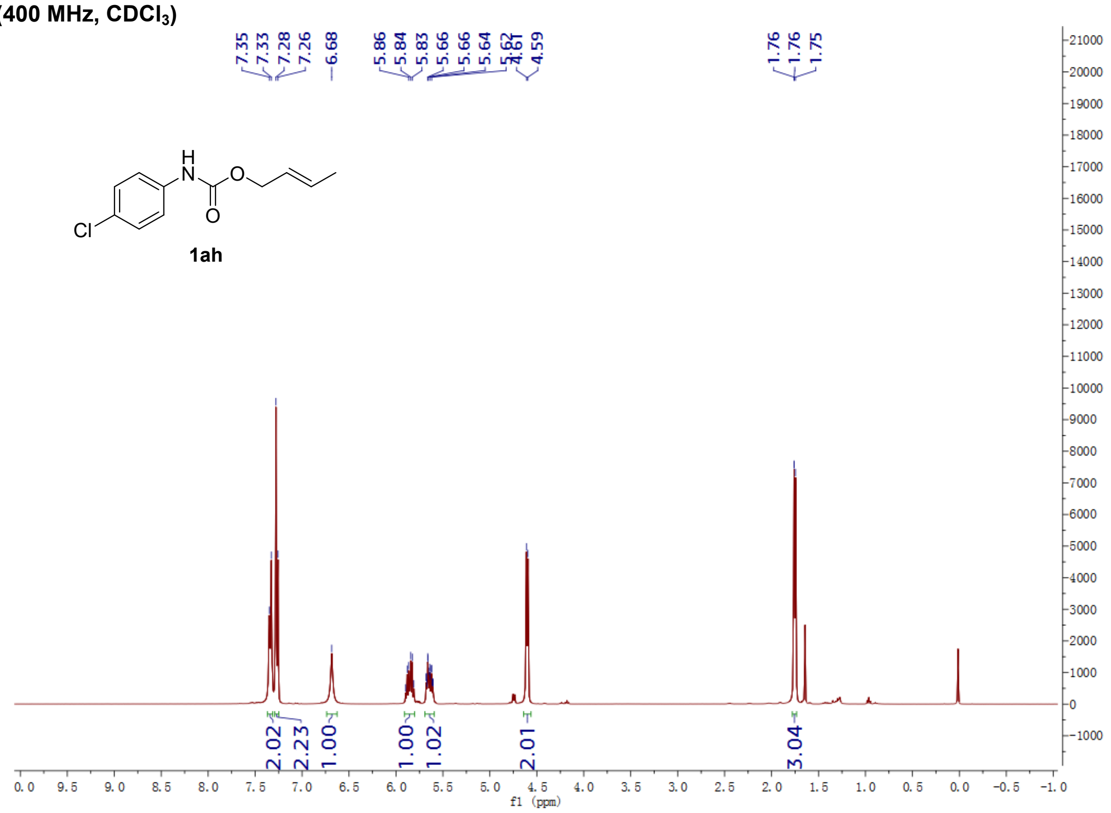

^{13}C-NMR (101 MHz, $CDCl_3$)

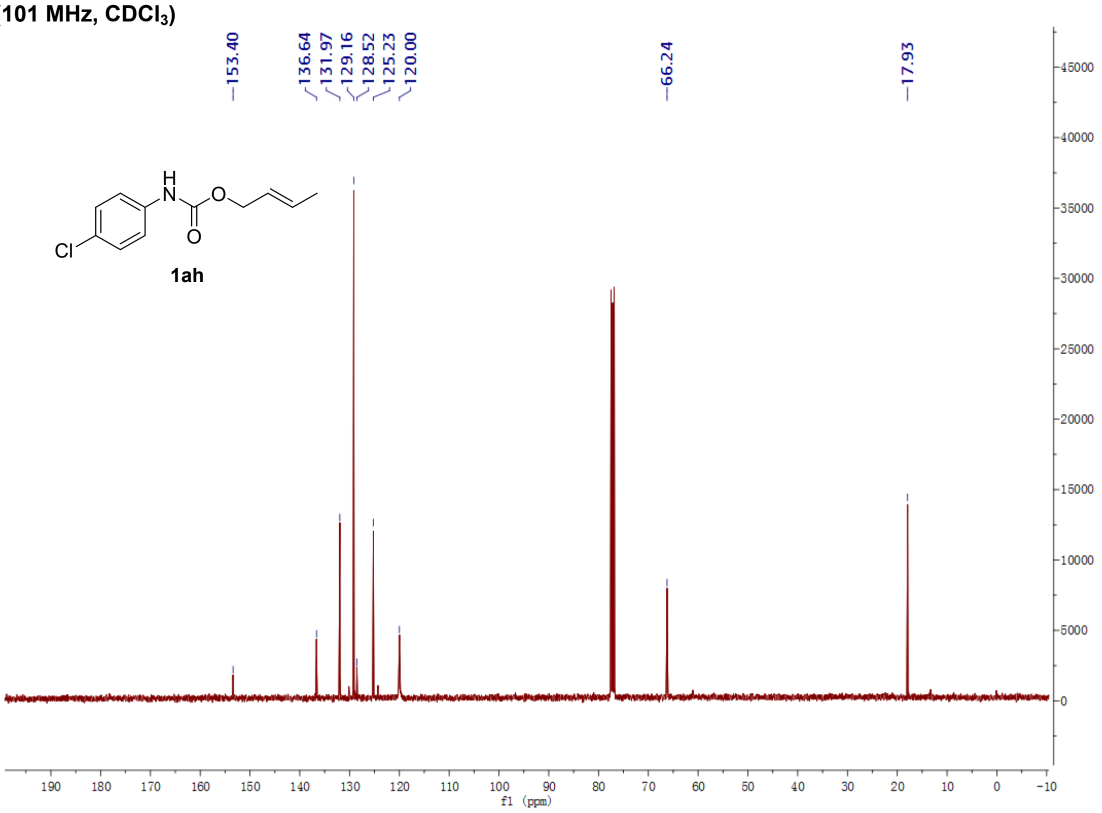

^{1}H-NMR (400 MHz, $CDCl_3$)

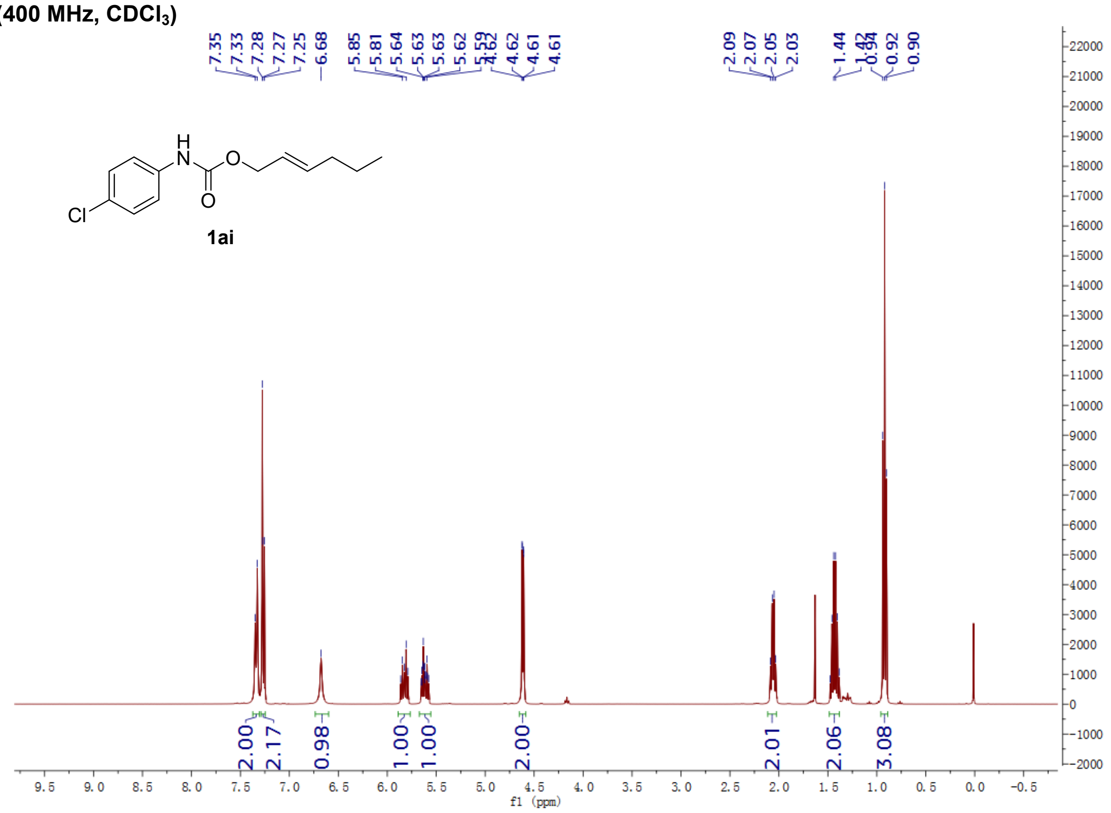

1ai

^{13}C-NMR (101 MHz, $CDCl_3$)

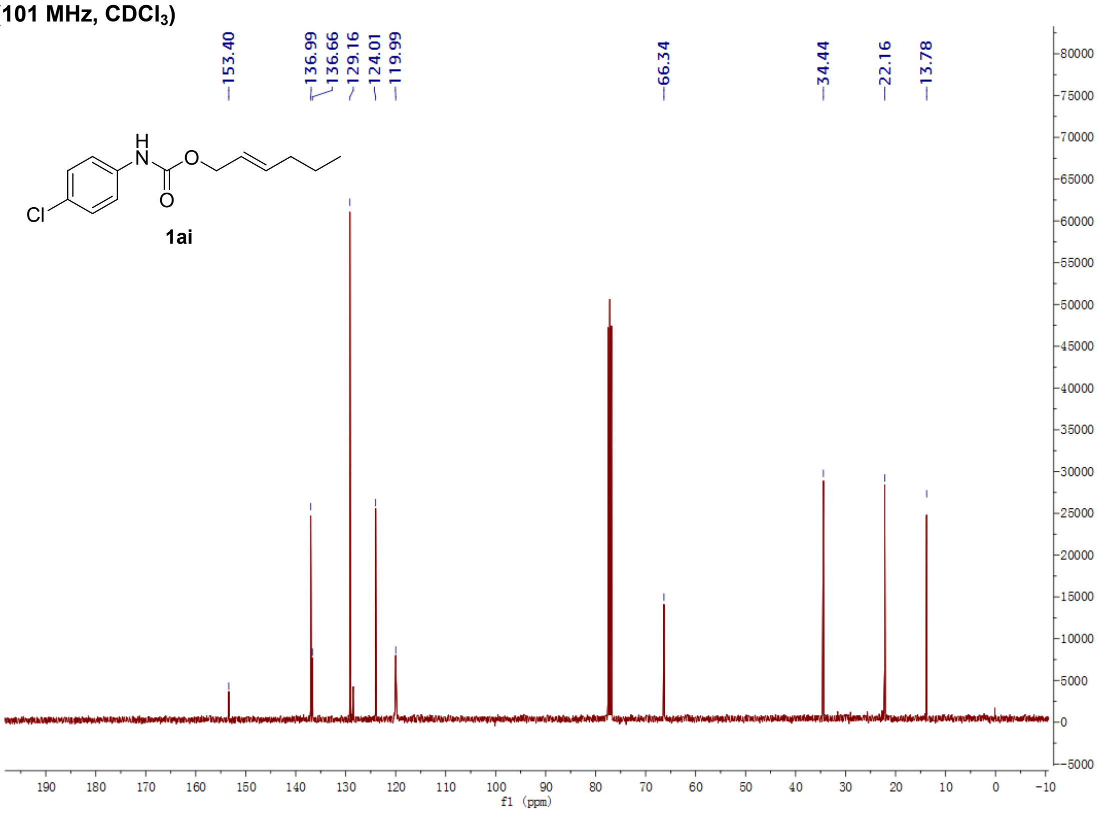

1ai

^{1}H-NMR (400 MHz, $CDCl_3$)

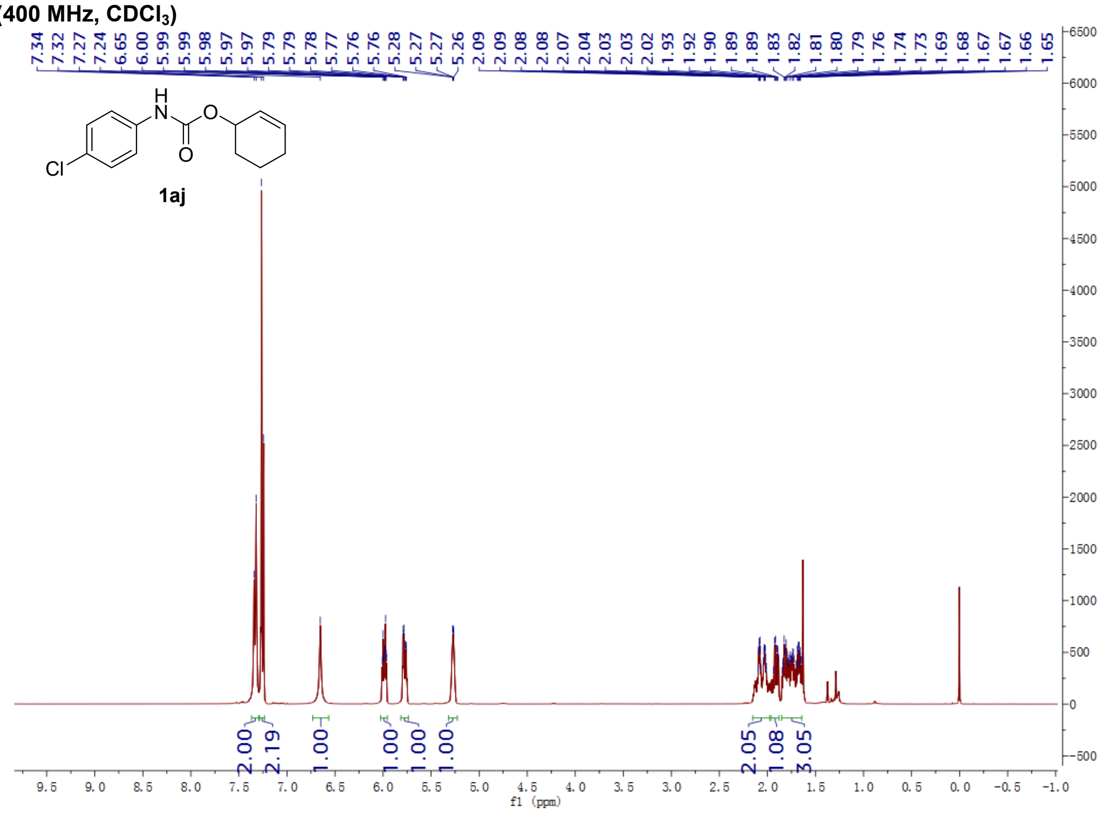

1aj

^{13}C-NMR (101 MHz, $CDCl_3$)

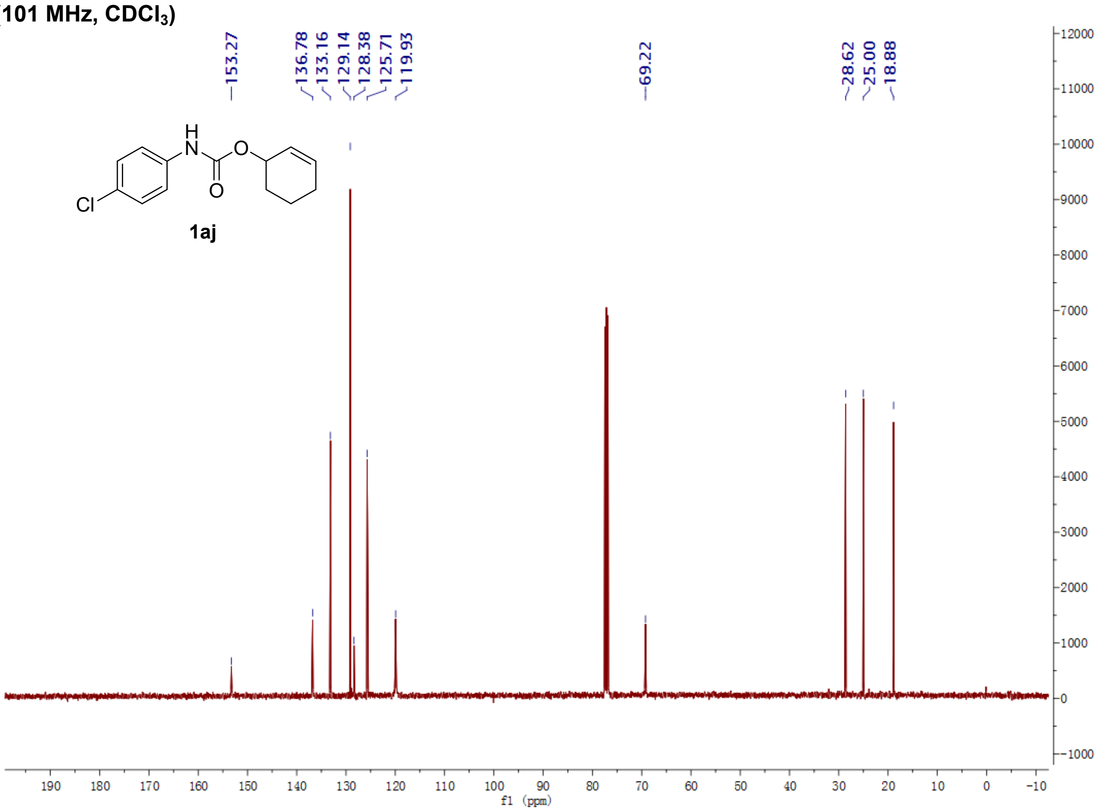

1aj

^{1}H-NMR (400 MHz, $CDCl_3$)

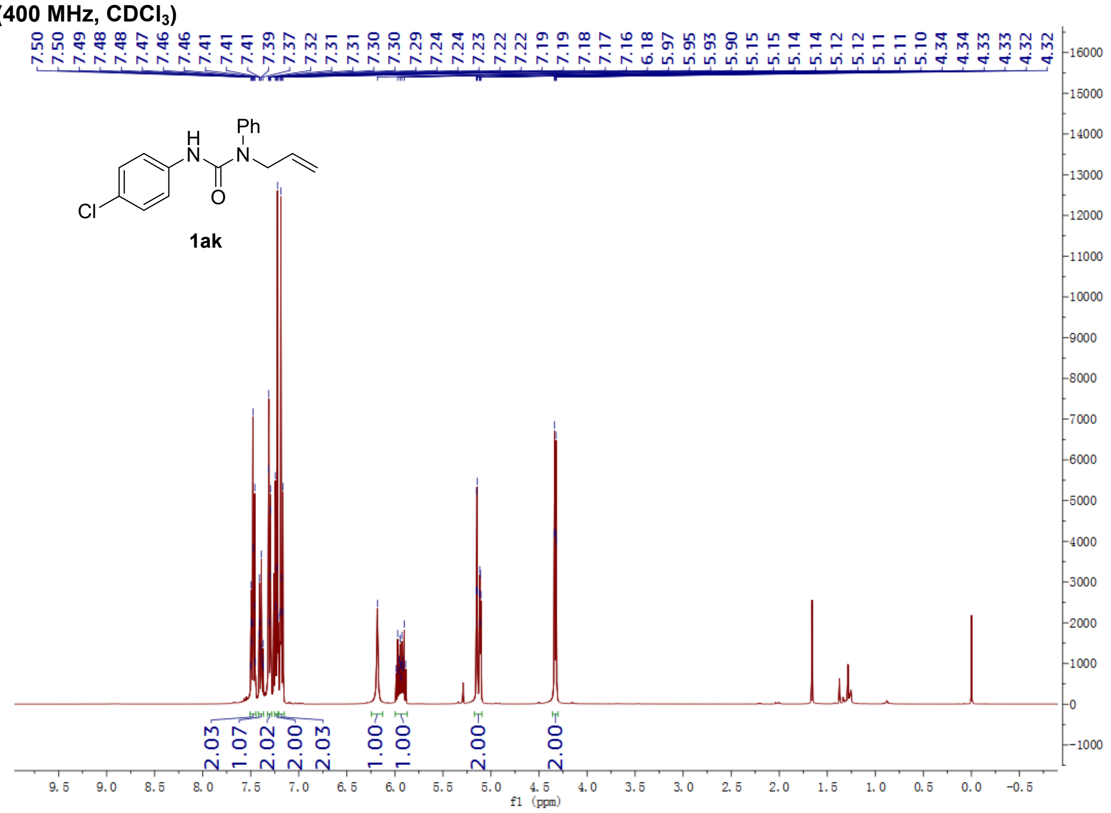

^{13}C-NMR (101 MHz, $CDCl_3$)

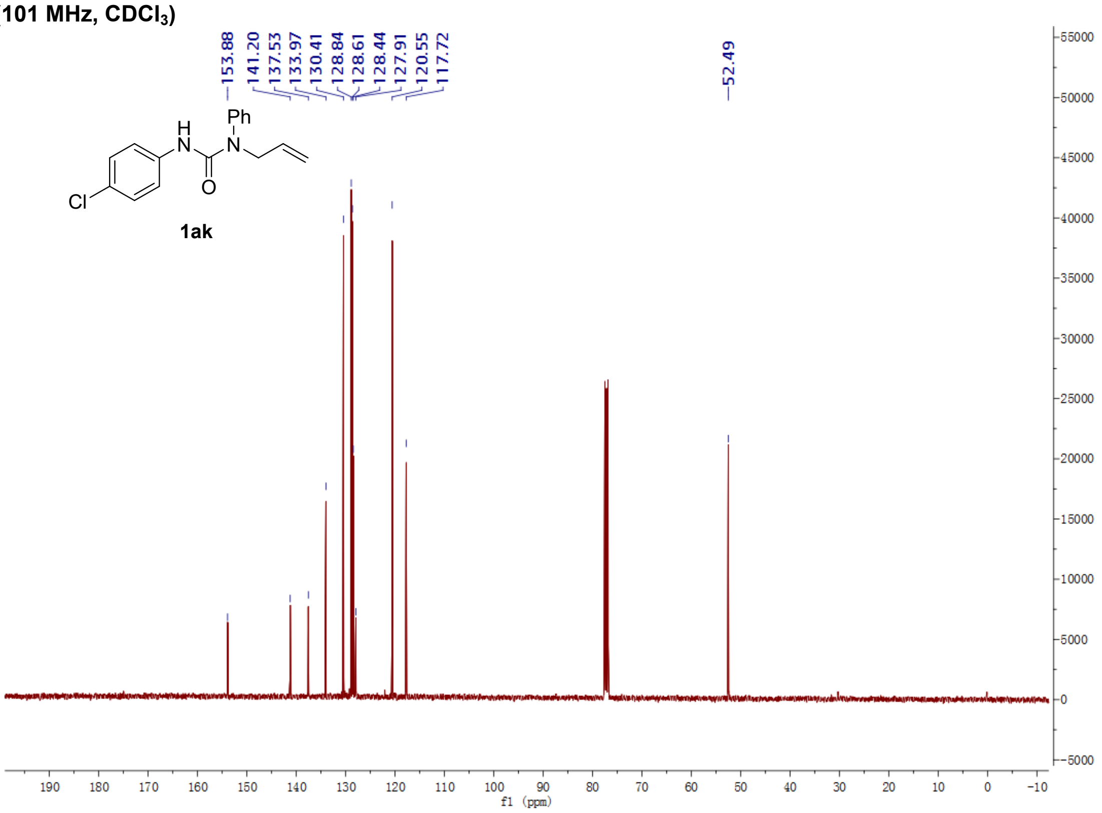

^{1}H-NMR (400 MHz, DMSO-d_6)

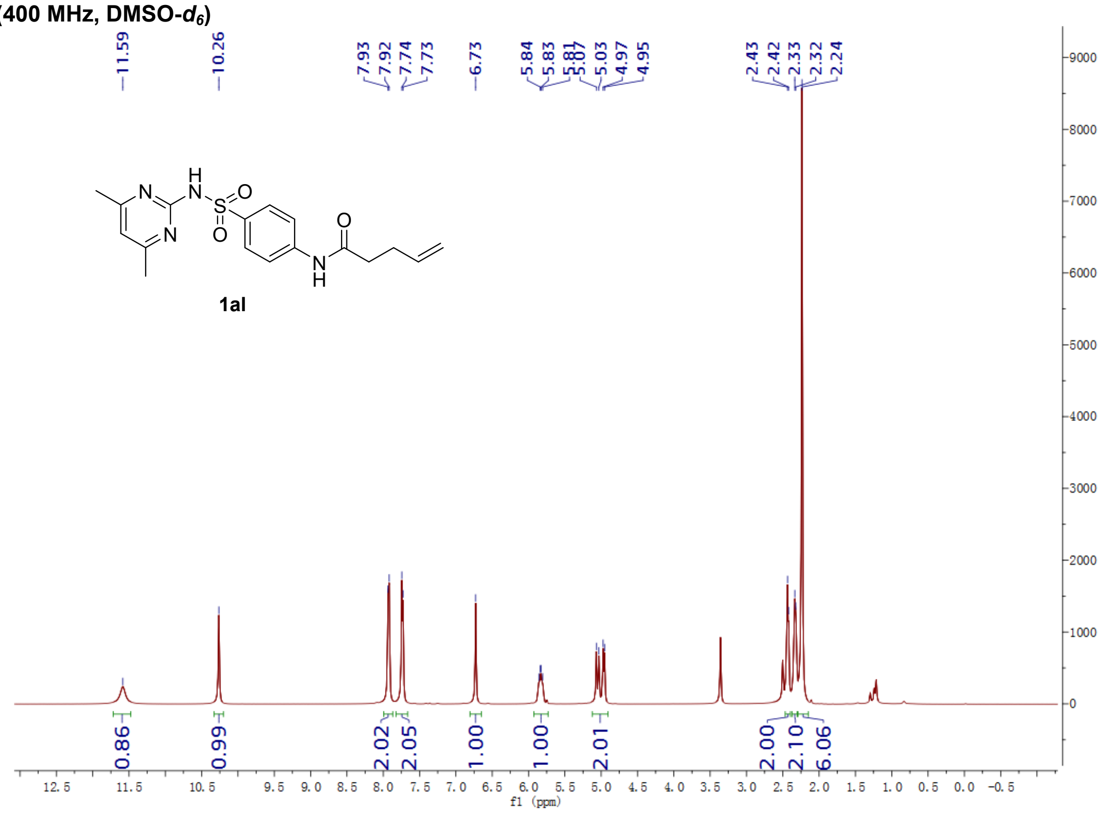

^{13}C-NMR (101 MHz, DMSO-d_6)

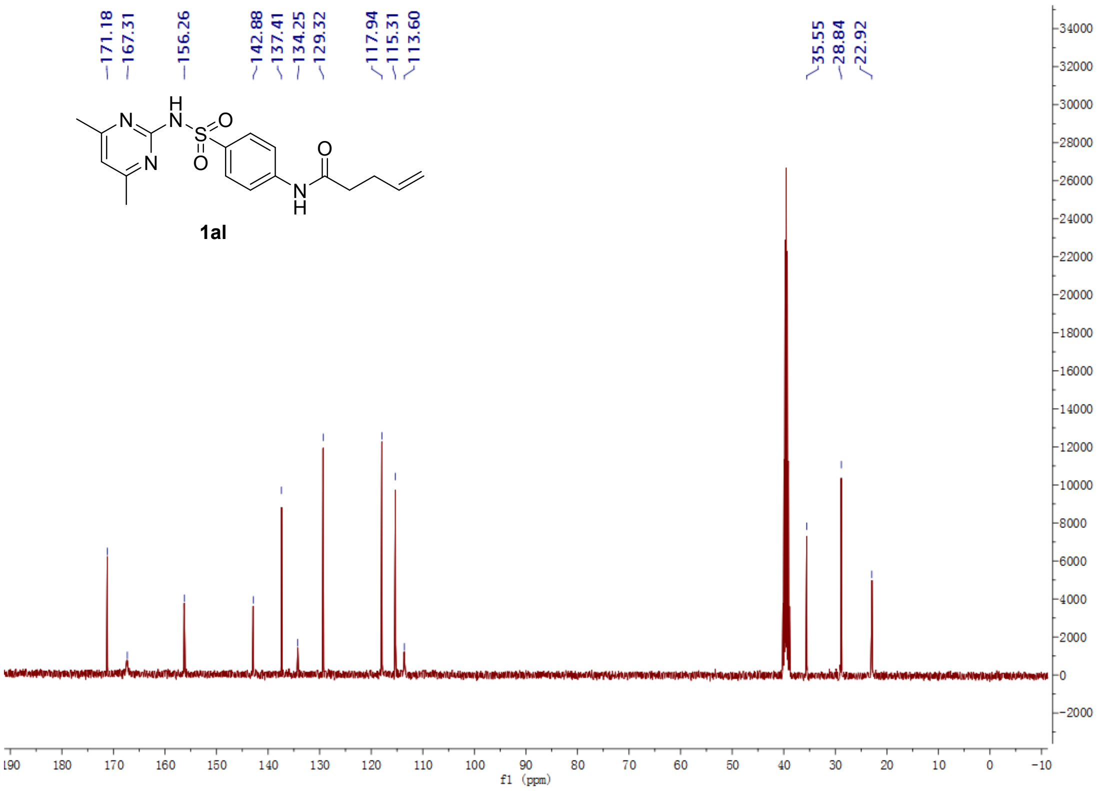

^{1}H-NMR (400 MHz, $CDCl_3$)

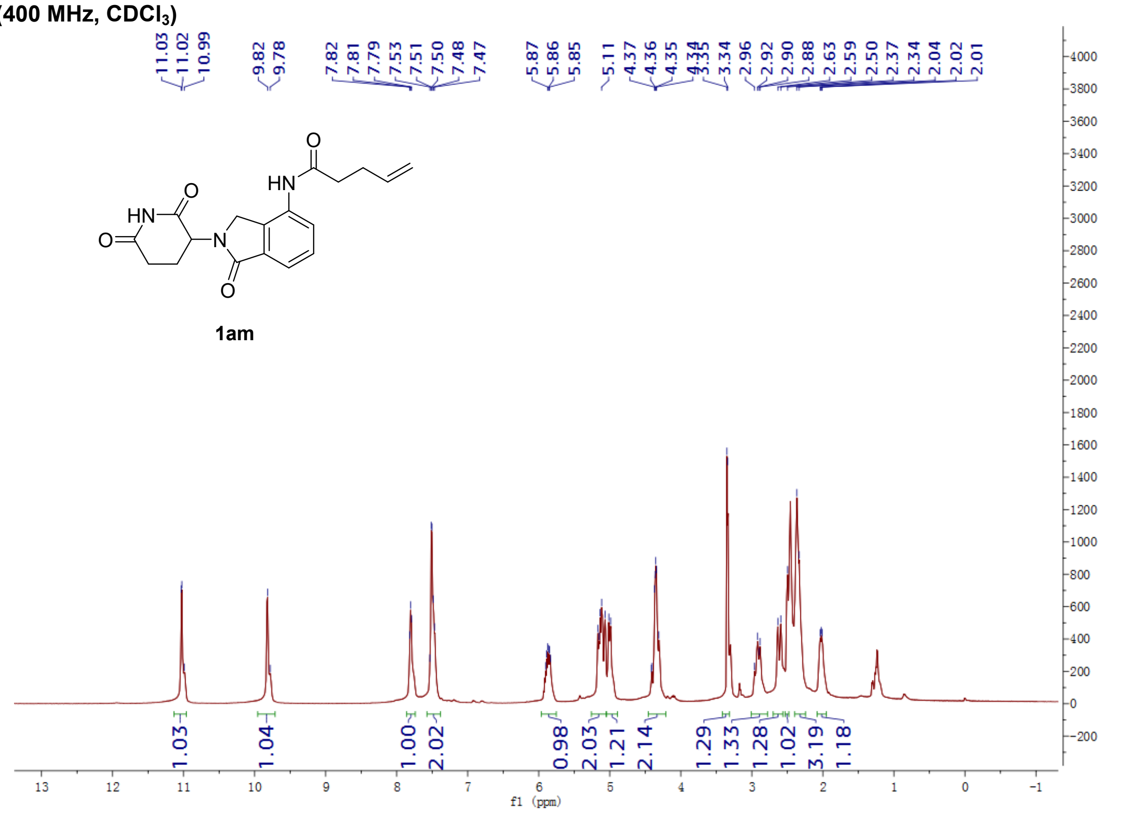

^{13}C-NMR (101 MHz, $CDCl_3$)

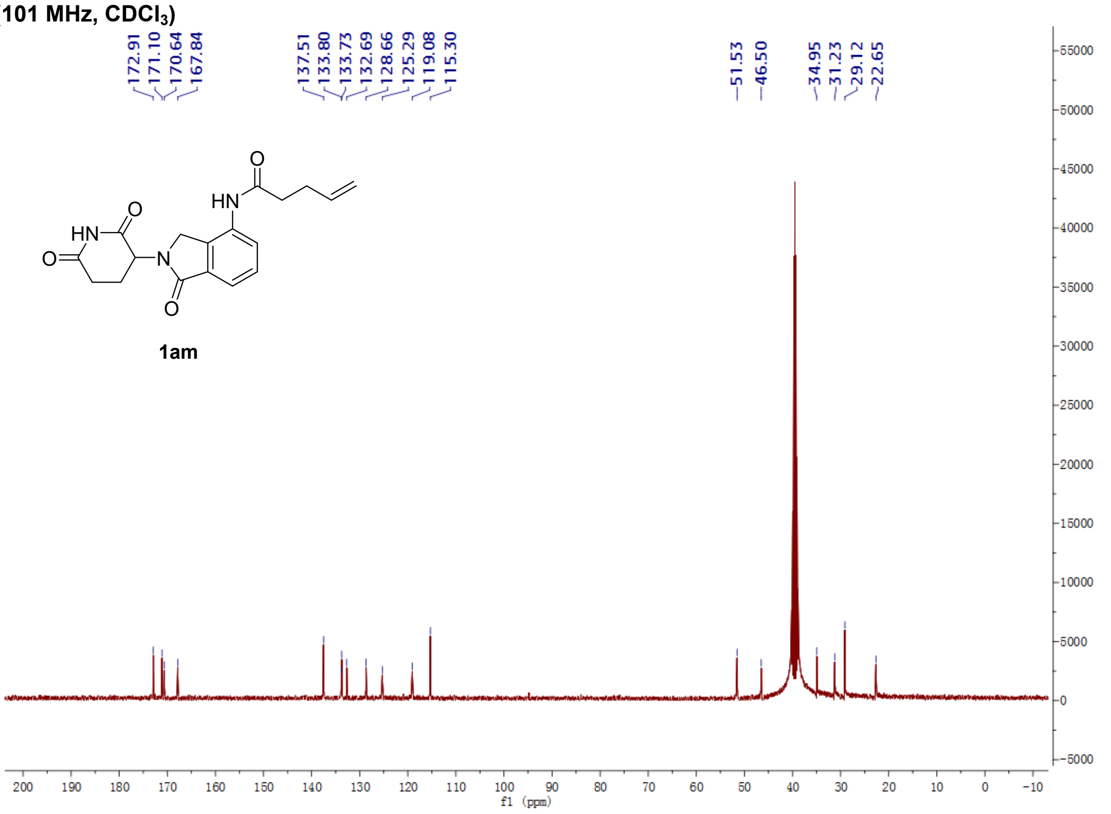

^{1}H-NMR (400 MHz, $CDCl_3$)

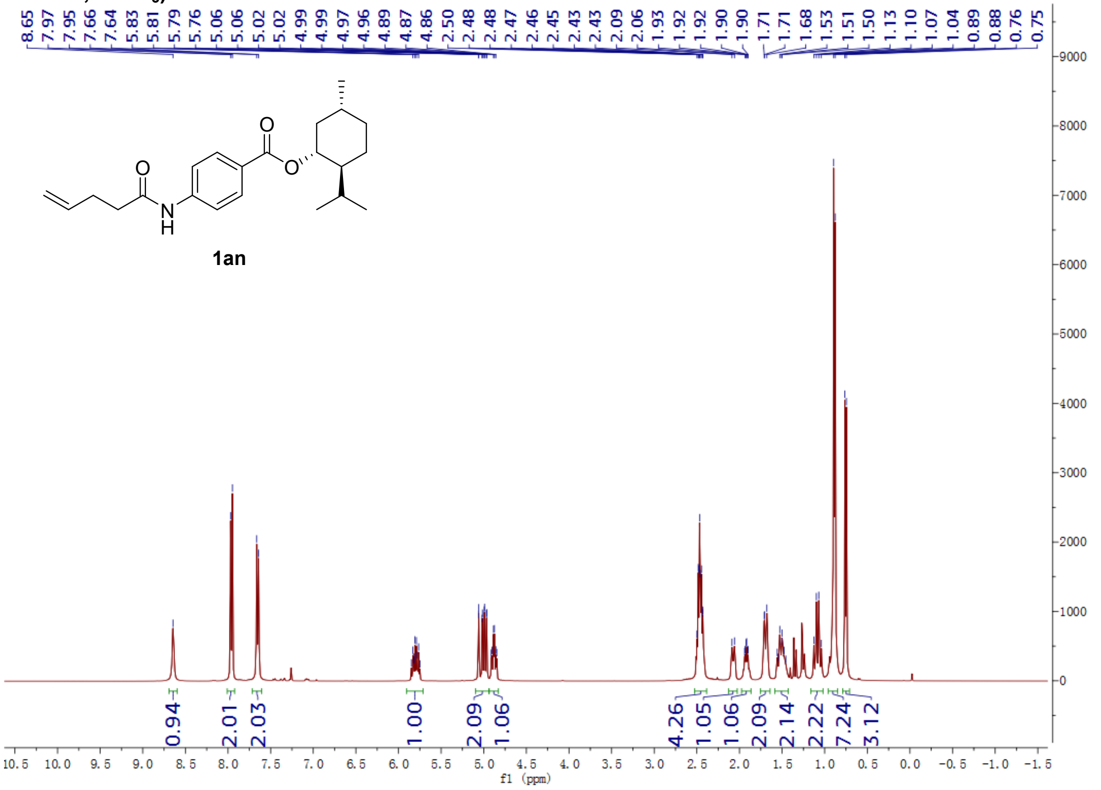

^{13}C-NMR (101 MHz, $CDCl_3$)

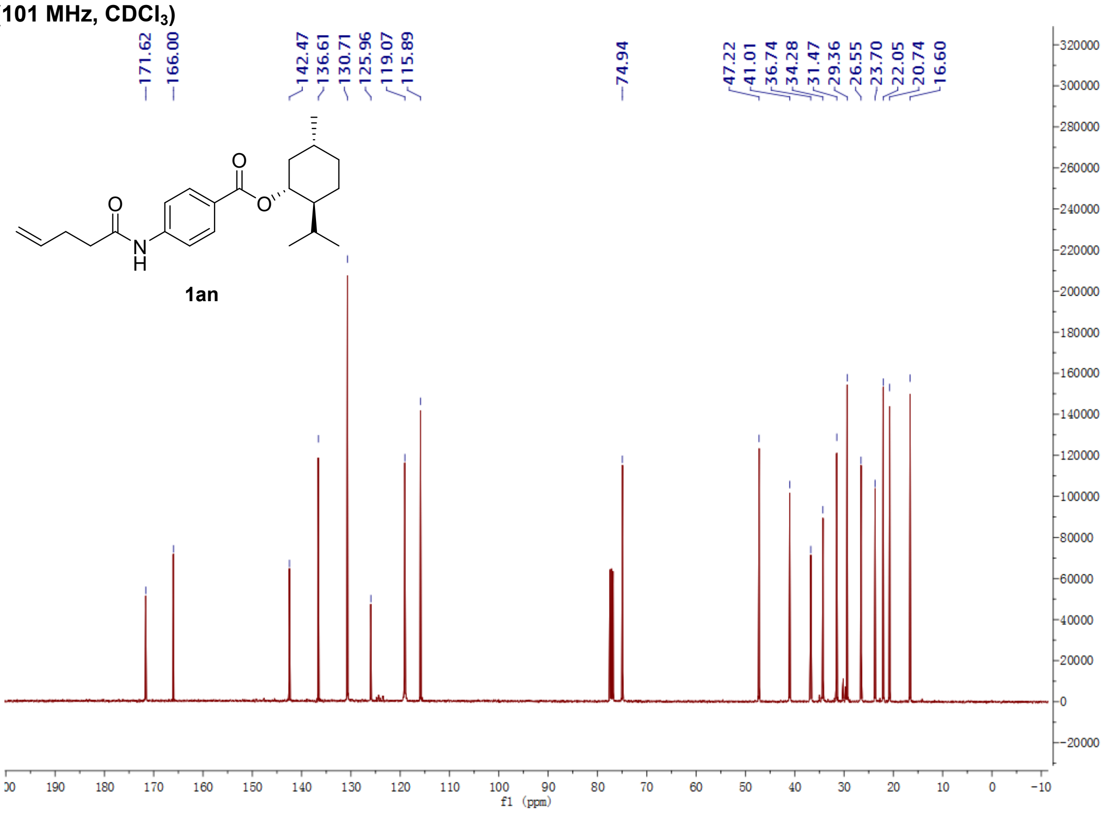

^{1}H-NMR (400 MHz, CDCl$_3$)

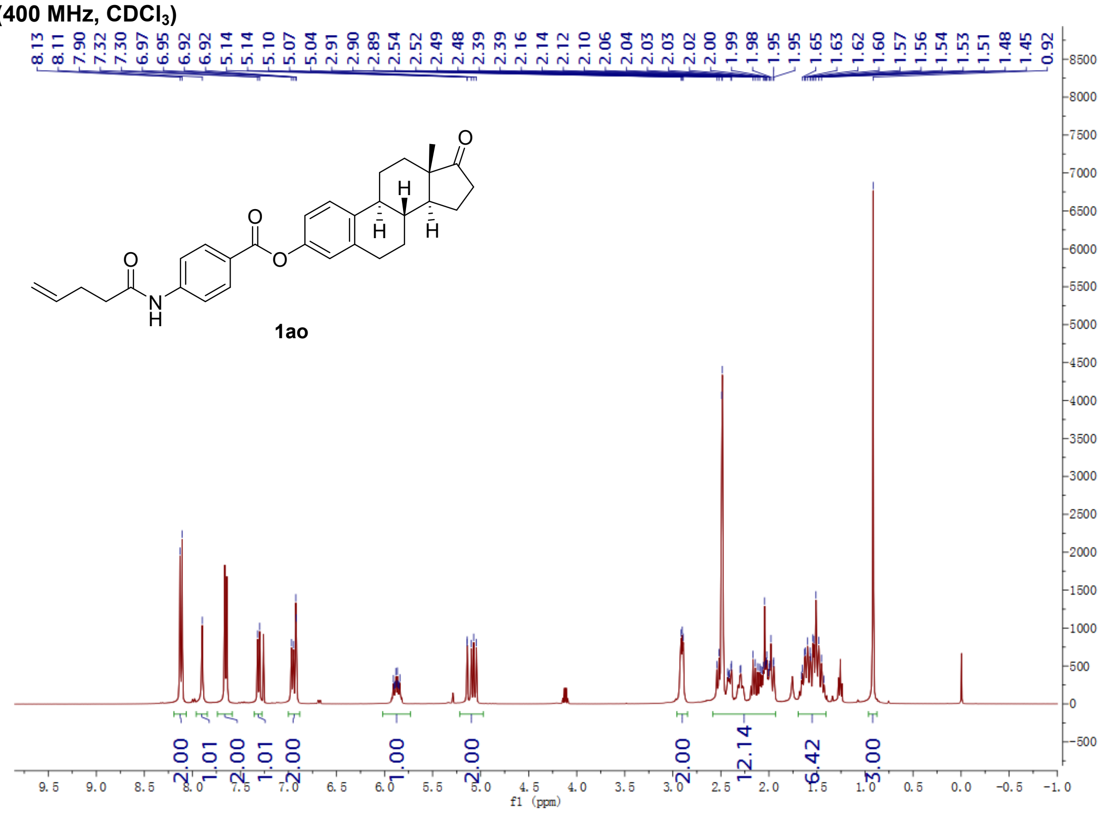

^{13}C-NMR (101 MHz, CDCl$_3$)

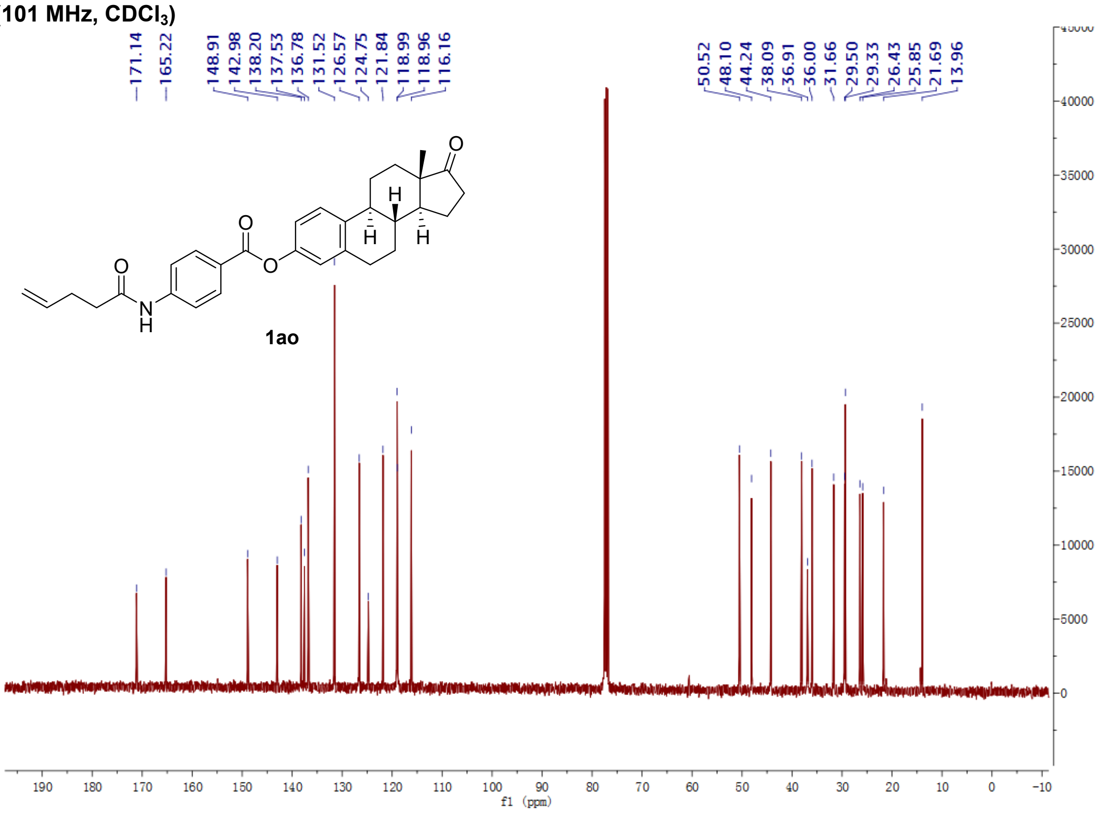

^{1}H-NMR (400 MHz, $CDCl_3$)

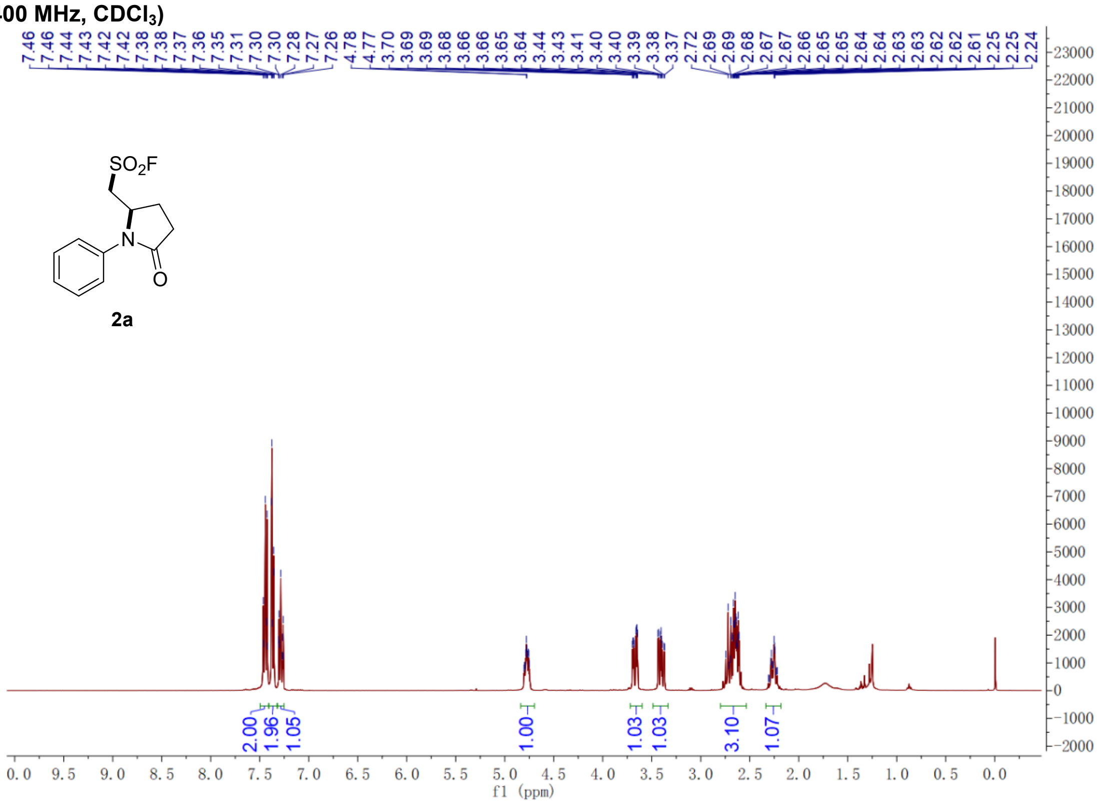

^{13}C-NMR (101 MHz, $CDCl_3$)

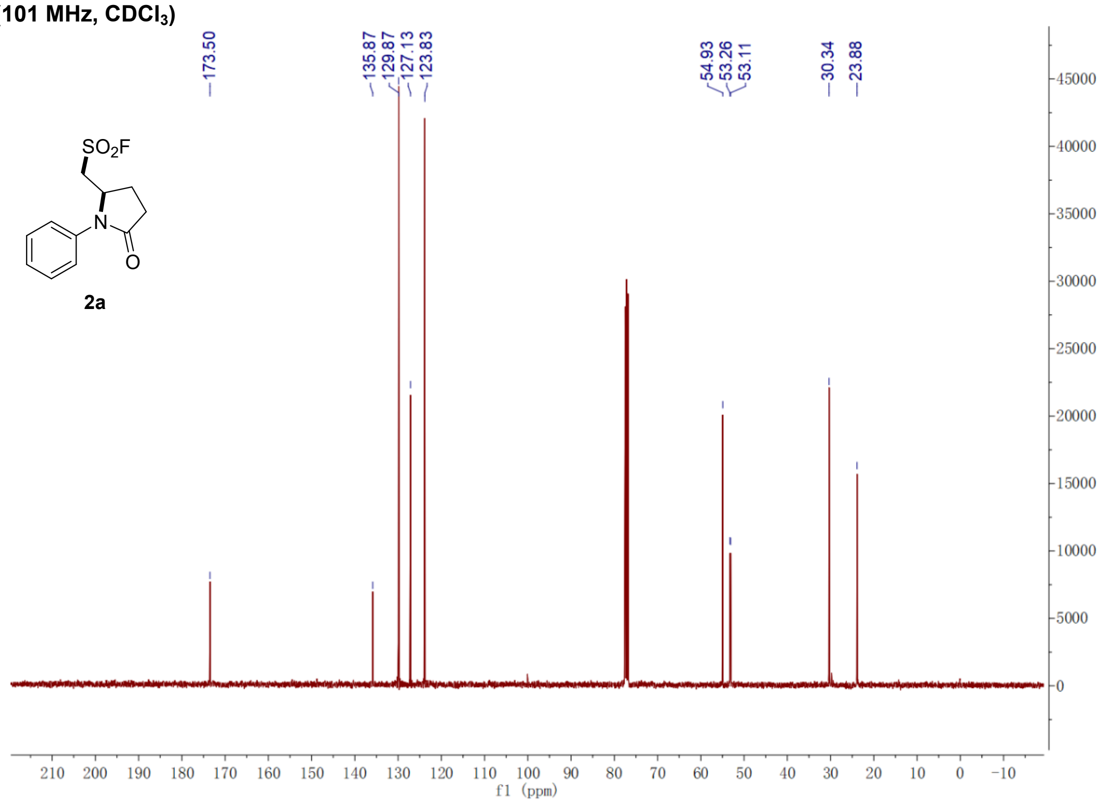

^{19}F-NMR (376 MHz, $CDCl_3$)

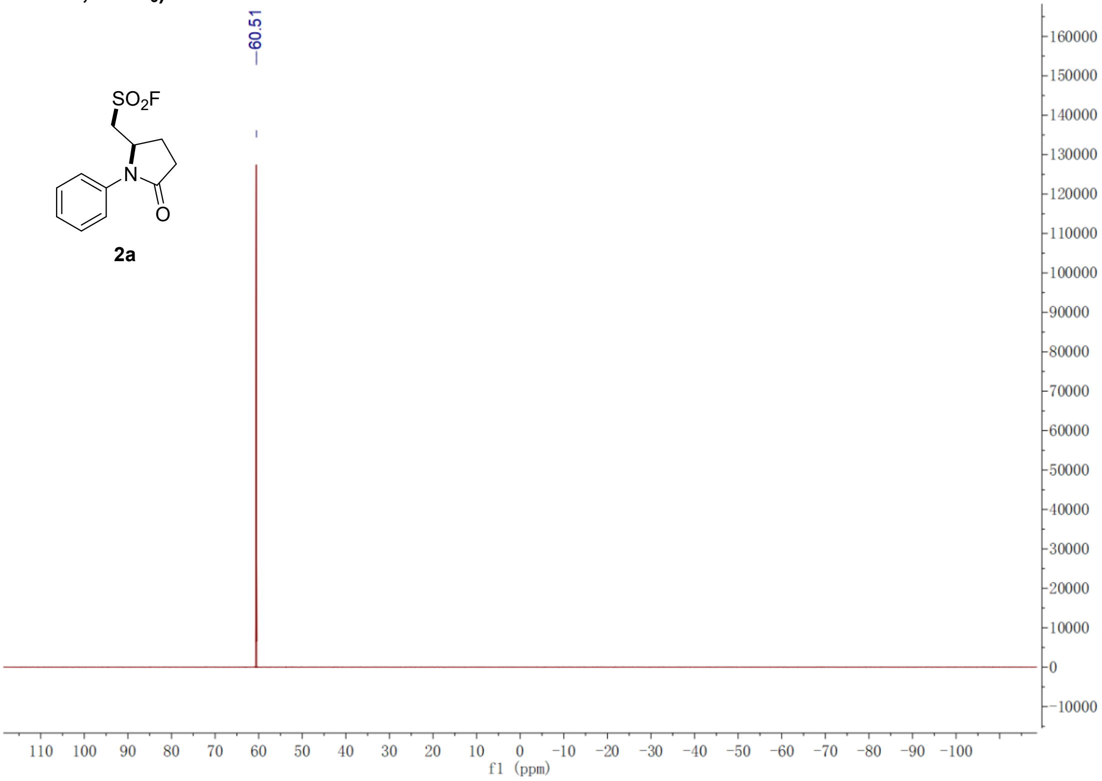

^{1}H-NMR (400 MHz, $CDCl_3$)

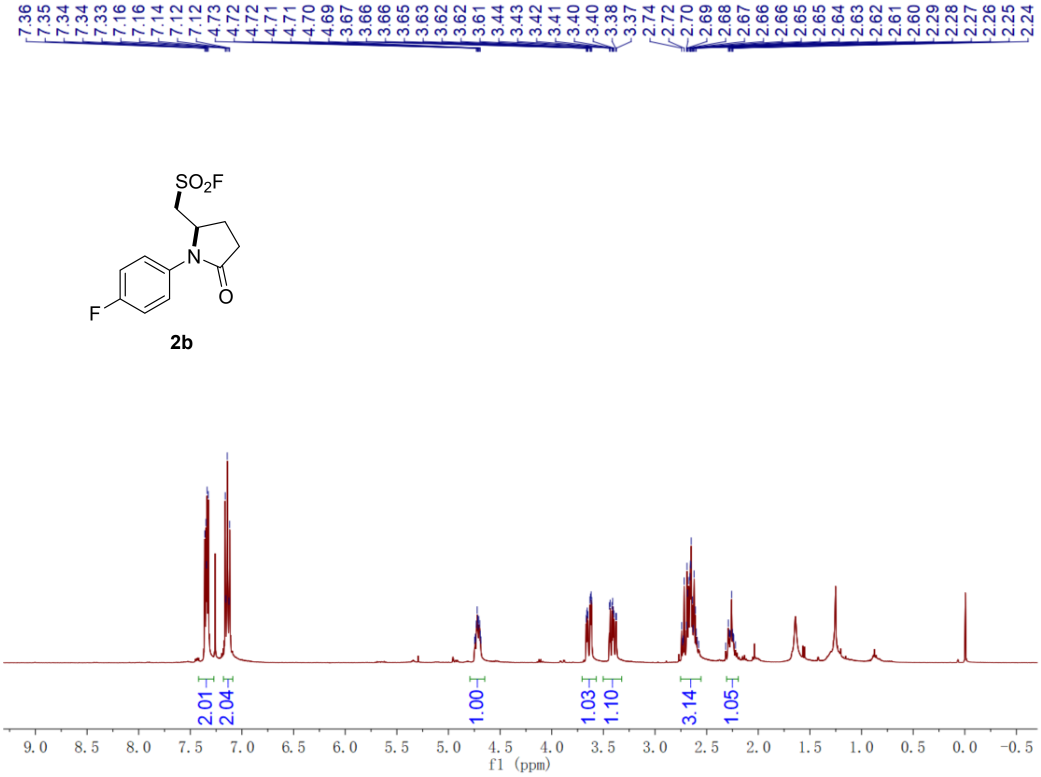

^{13}C-NMR (101 MHz, CDCl$_3$)

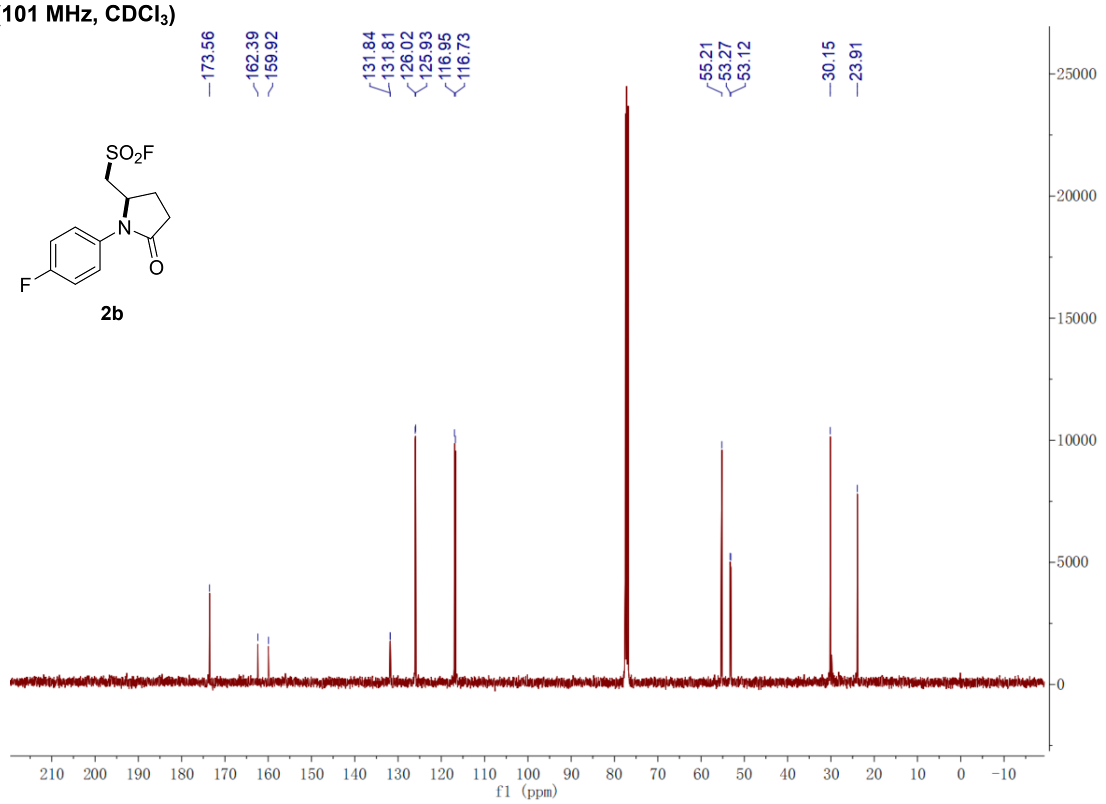

^{19}F NMR (376 MHz, CDCl$_3$)

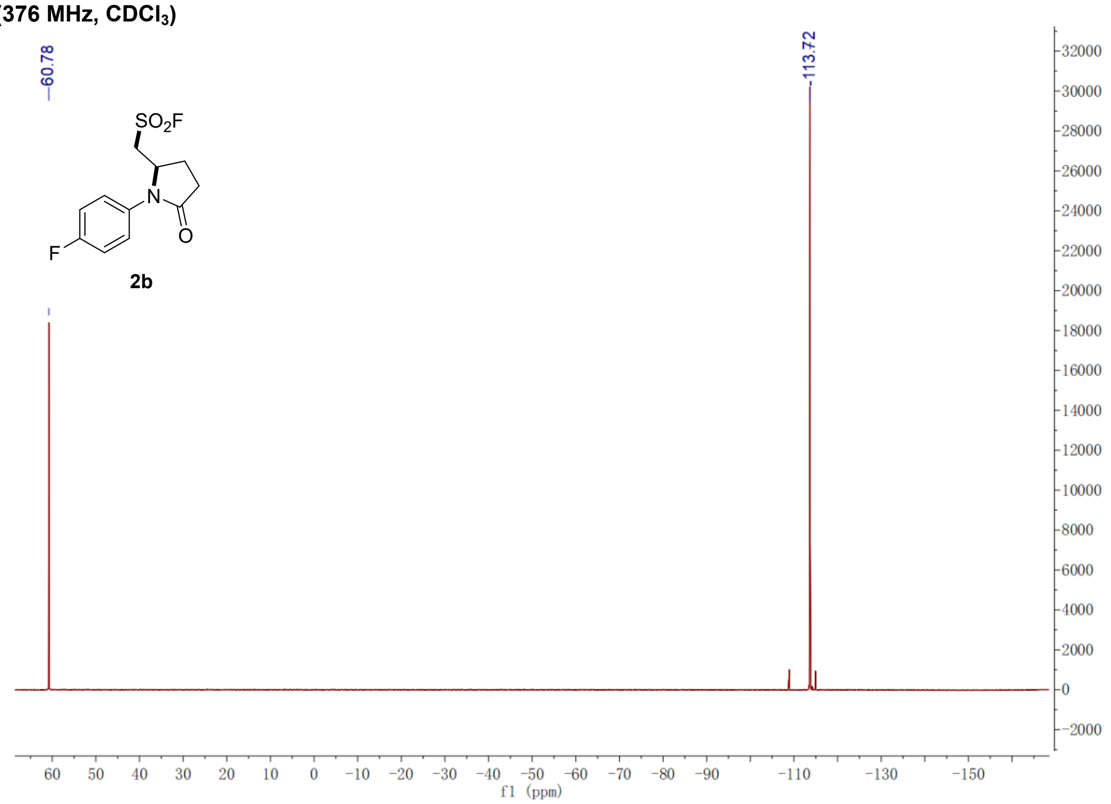

^{1}H-NMR (400 MHz, $CDCl_3$)

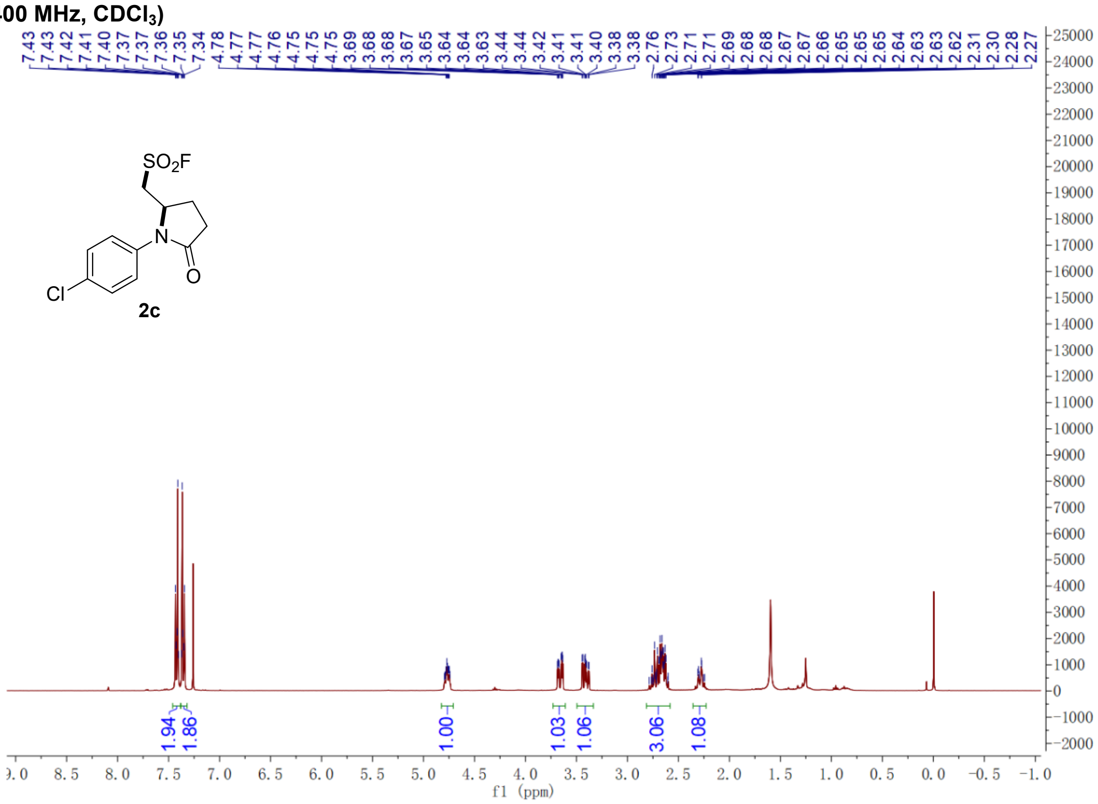

^{13}C-NMR (101 MHz, $CDCl_3$)

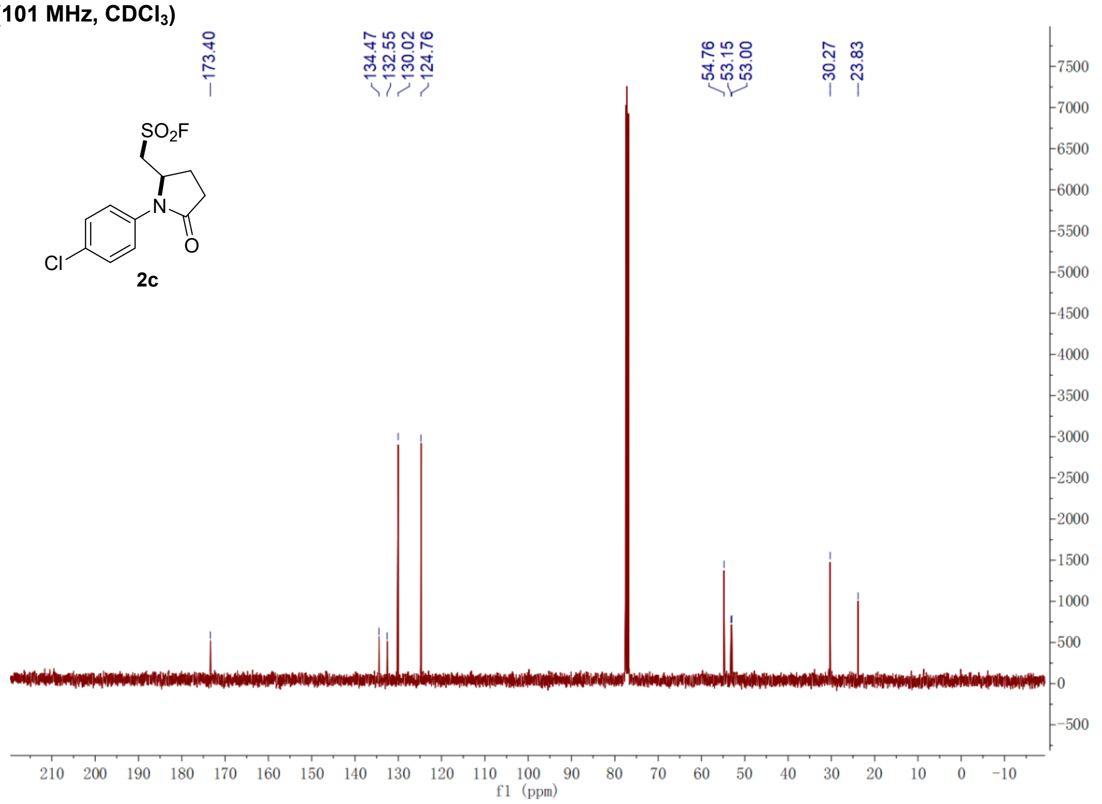

^{19}F NMR (376 MHz, $CDCl_3$)

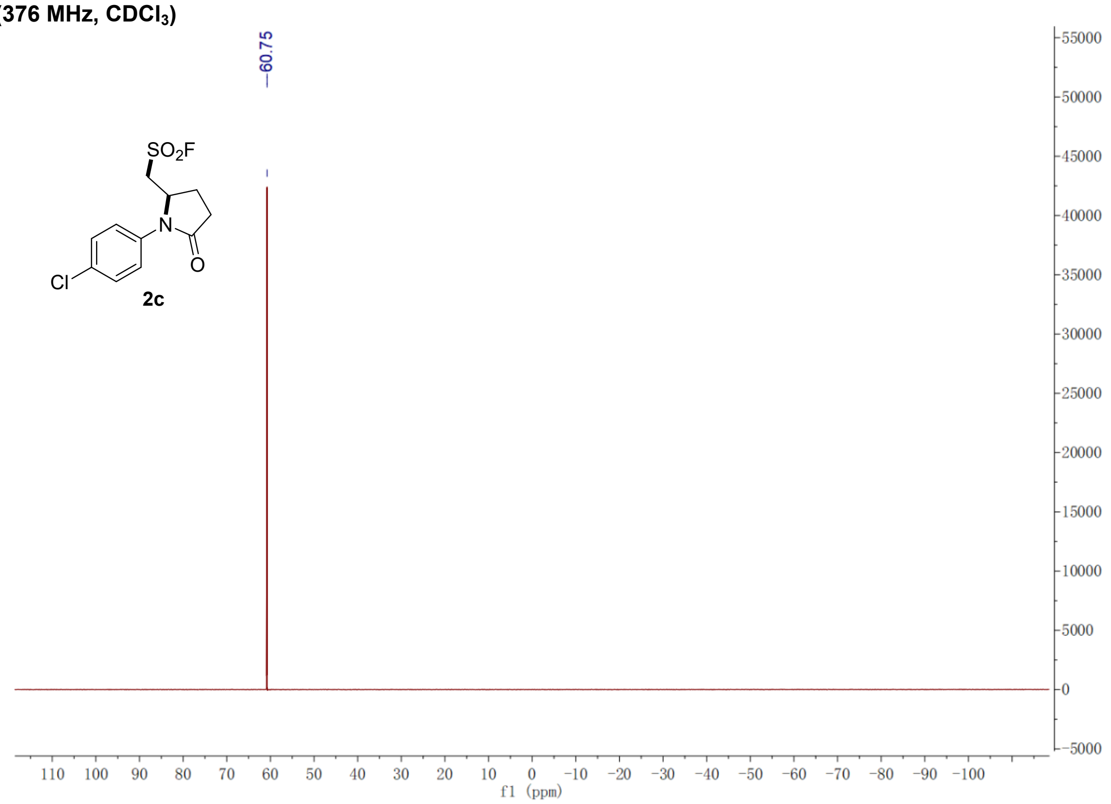

^{1}H-NMR (400 MHz, $CDCl_3$)

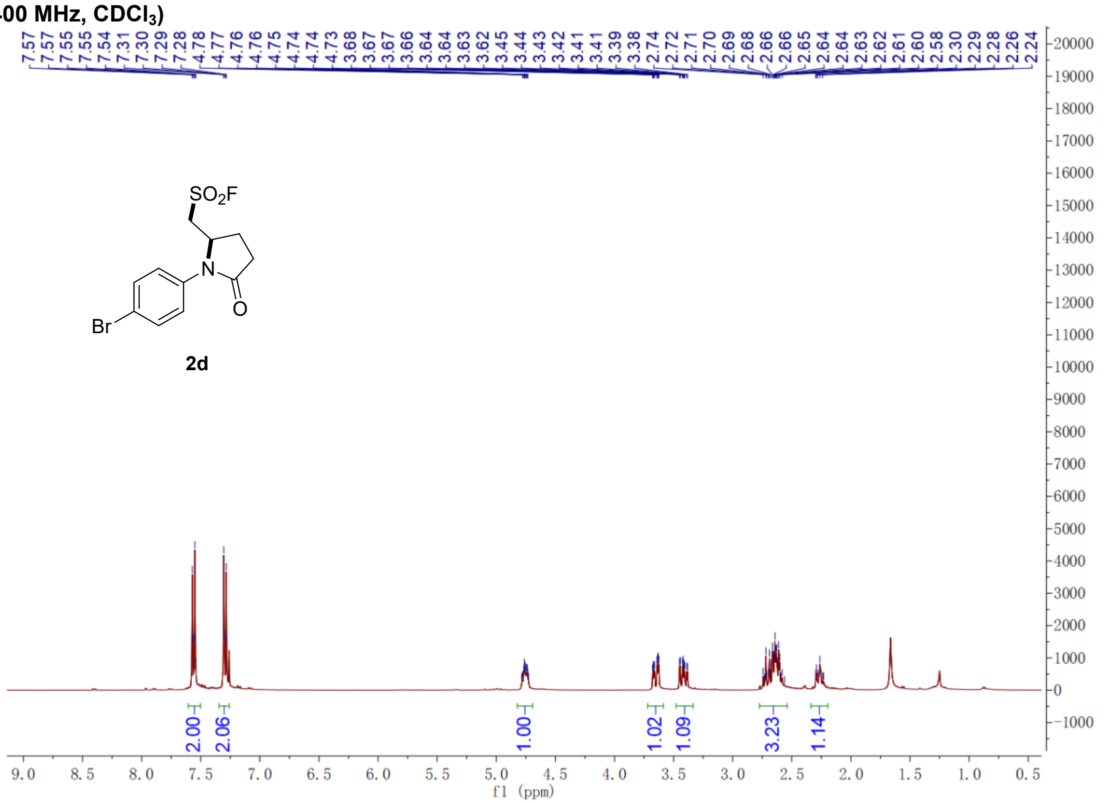

^{13}C-NMR (126 MHz, CDCl$_3$)

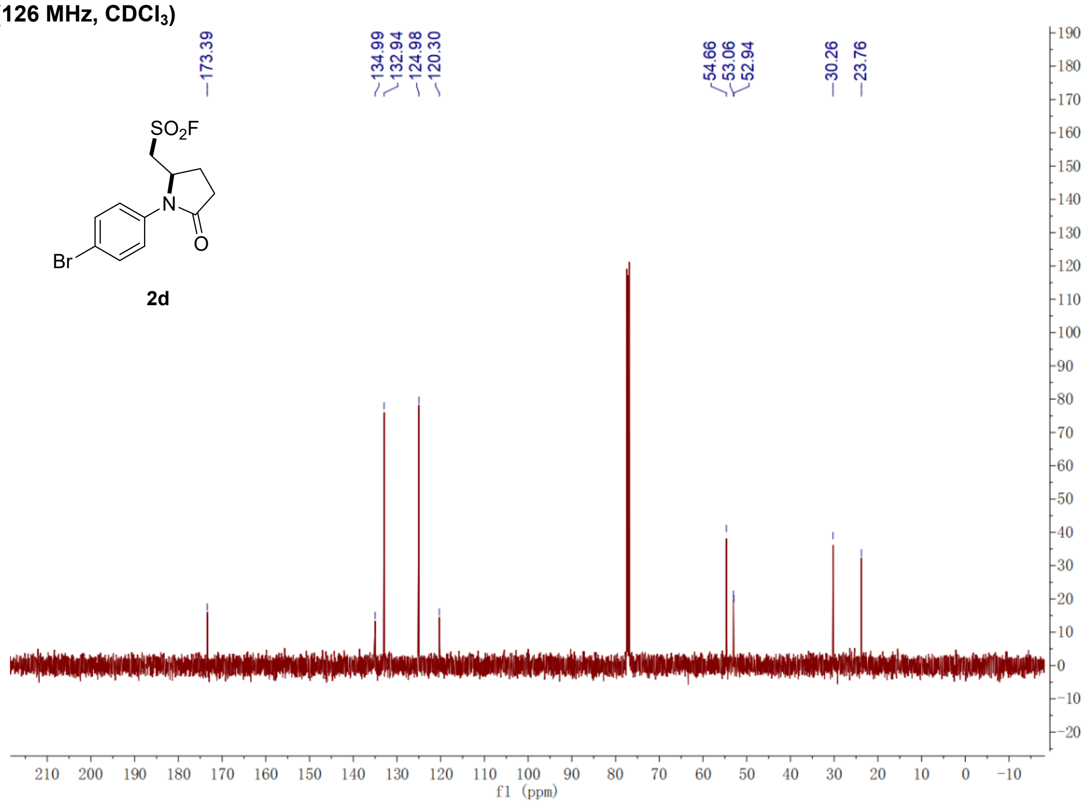

^{19}F NMR (376 MHz, CDCl$_3$)

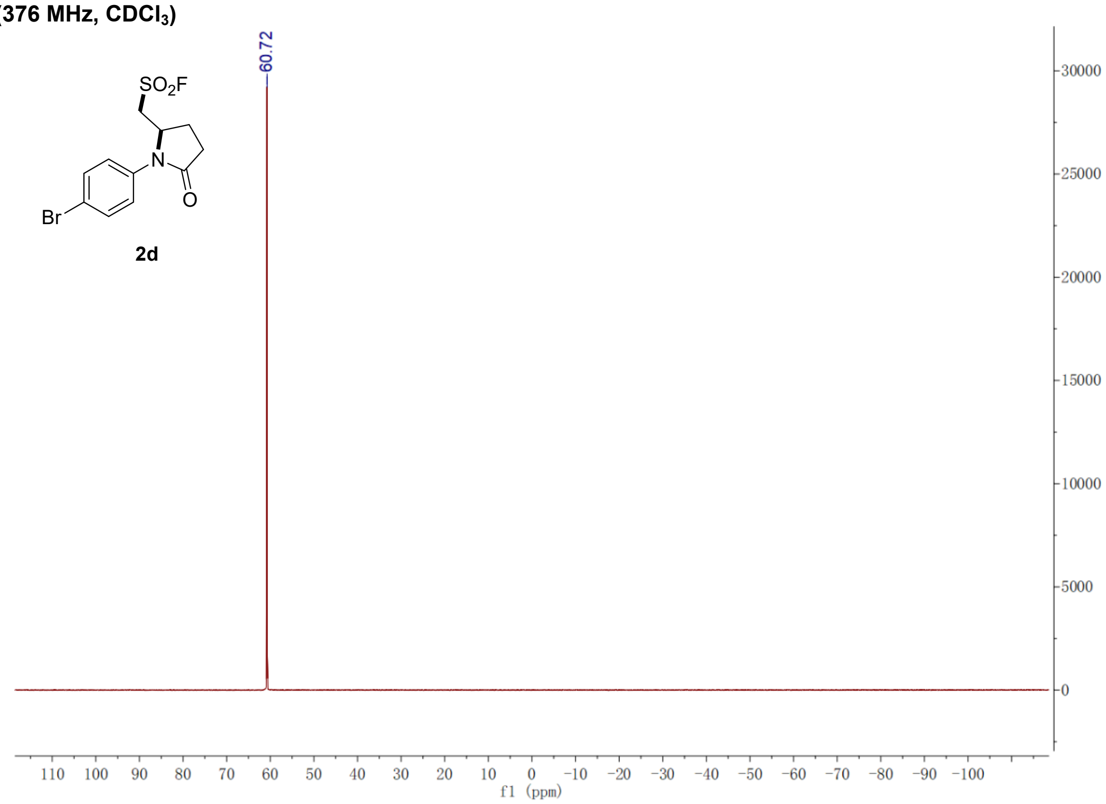

^{1}H-NMR (400 MHz, CDCl$_3$)

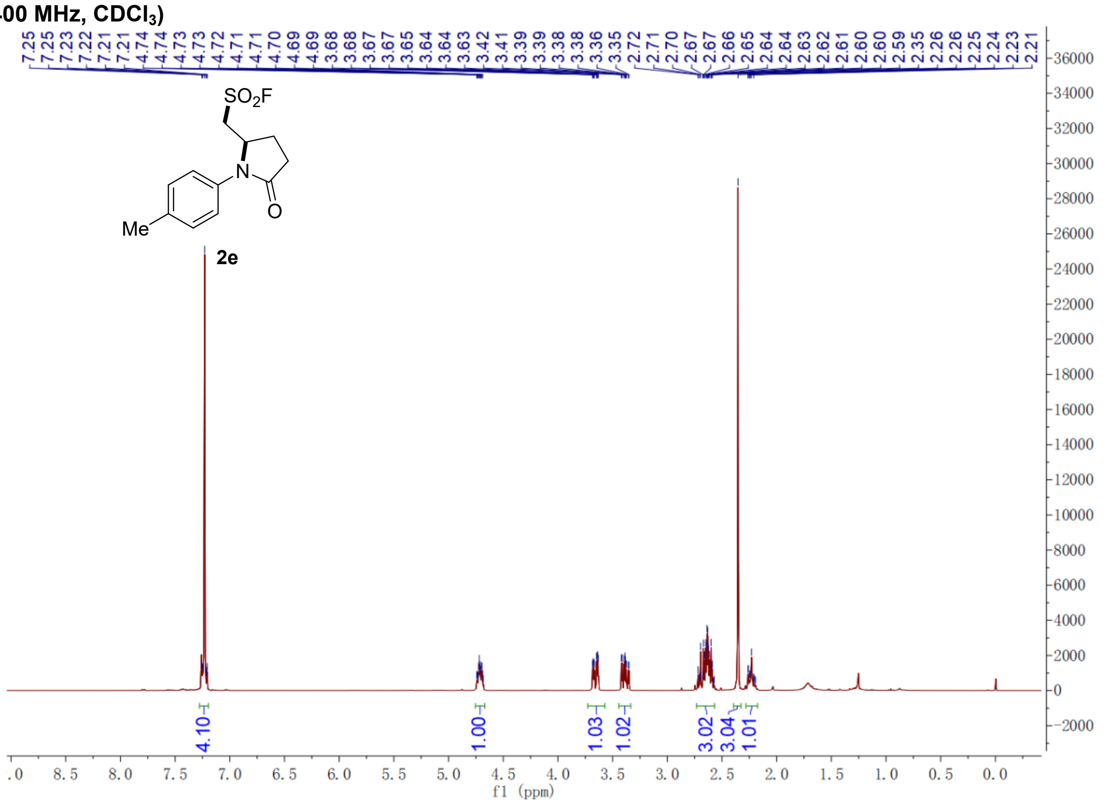

^{13}C-NMR (101 MHz, CDCl$_3$)

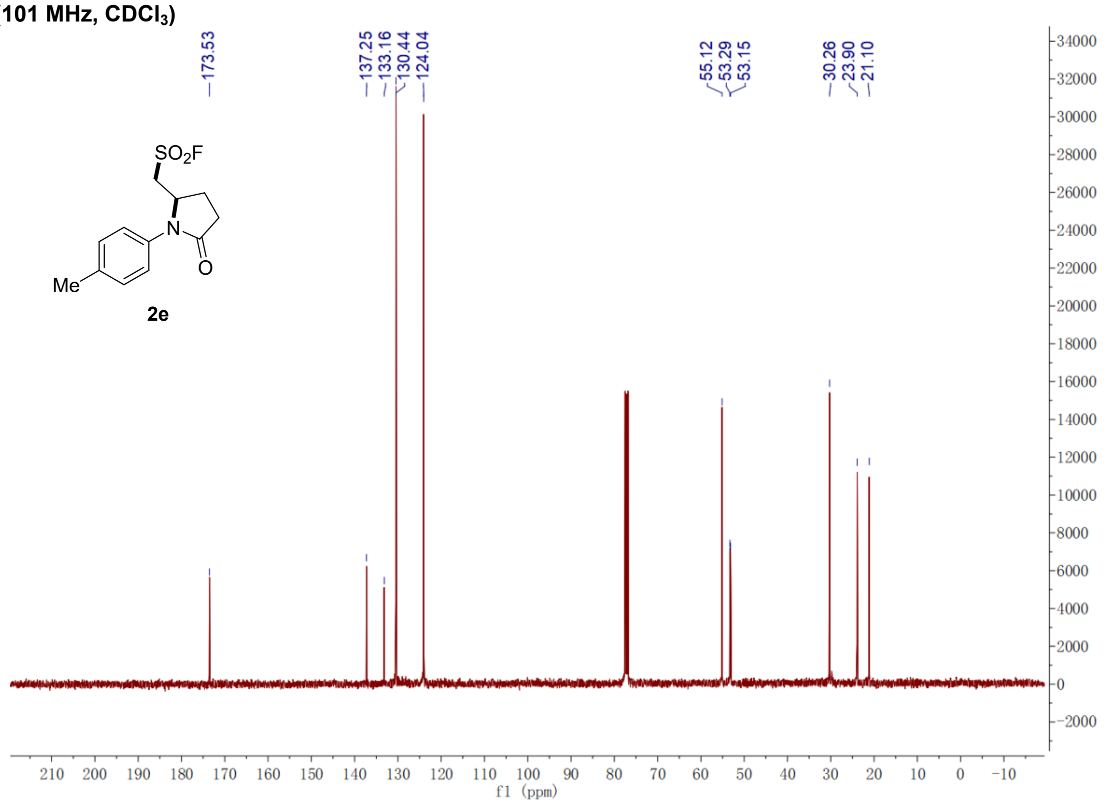

^{19}F NMR (376 MHz, $CDCl_3$)

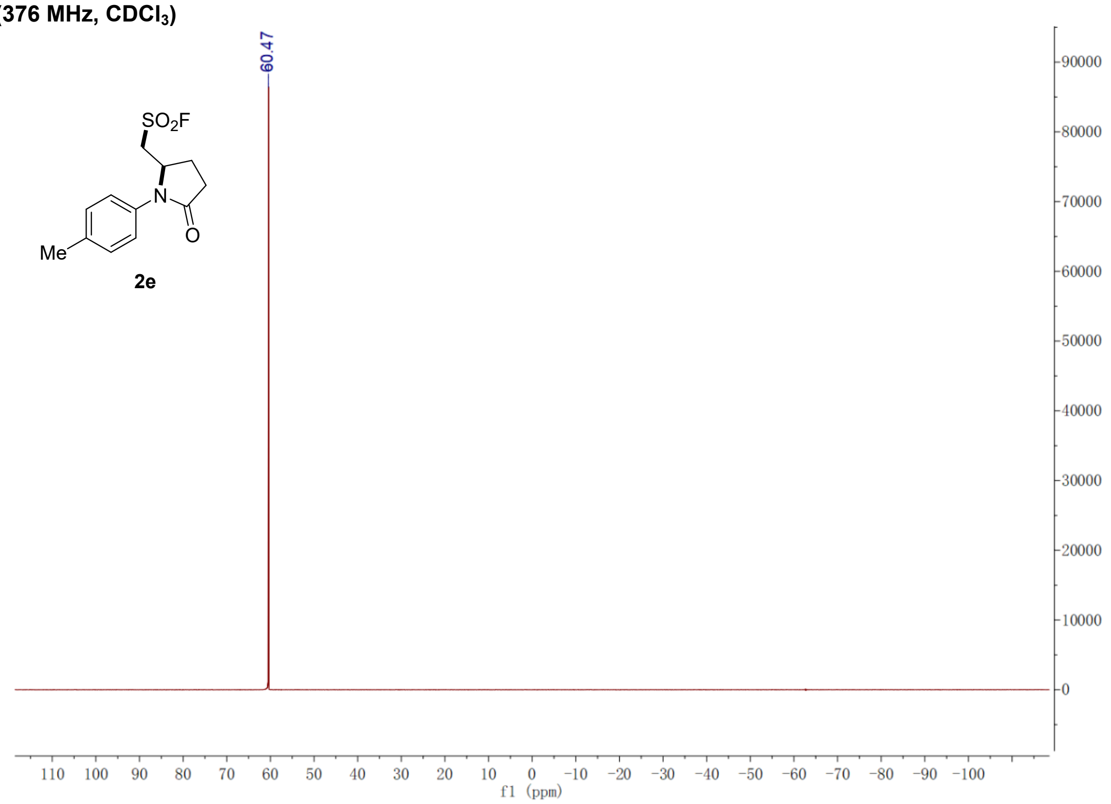

2e

^{1}H-NMR (400 MHz, $CDCl_3$)

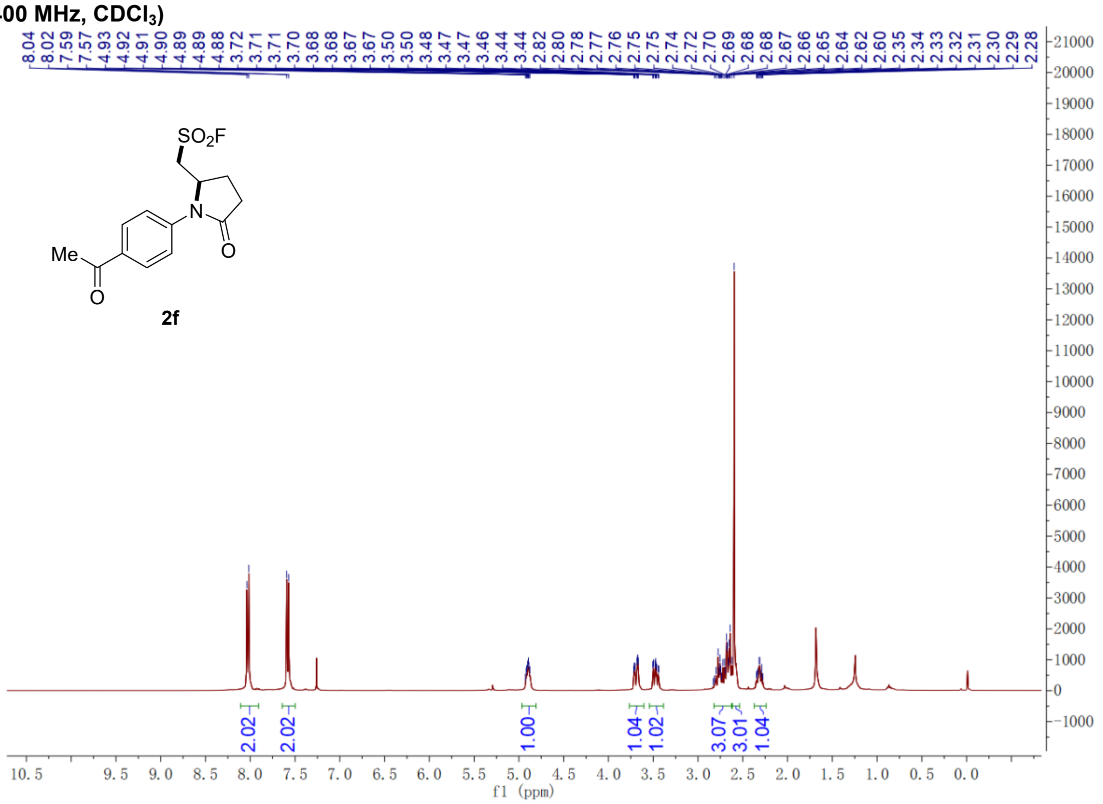

2f

^{13}C-NMR (126 MHz, $CDCl_3$)

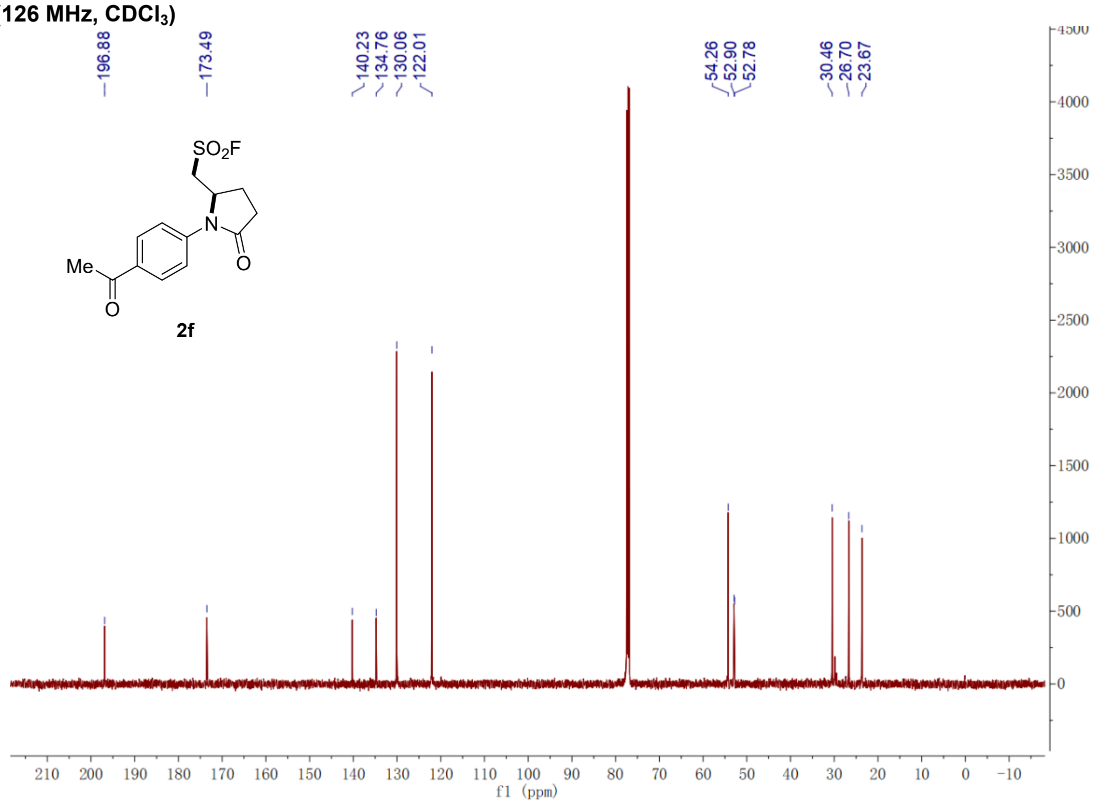

^{19}F NMR (376 MHz, $CDCl_3$)

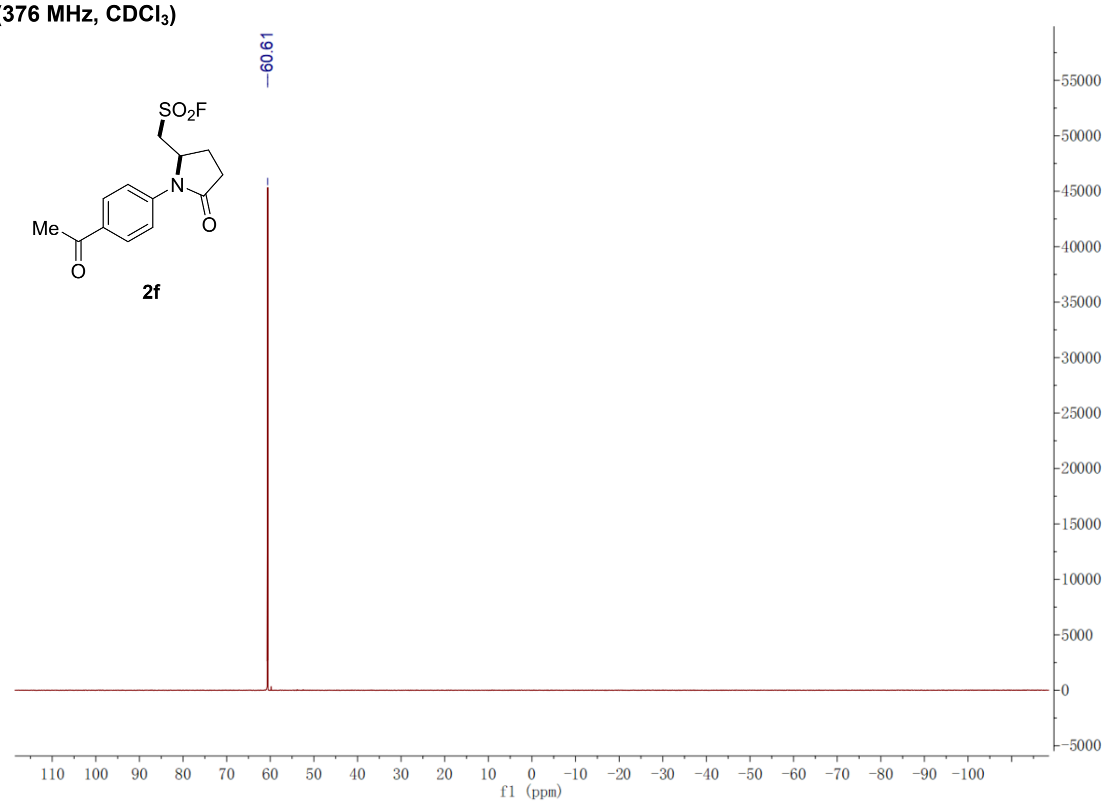

^{1}H-NMR (400 MHz, $CDCl_3$)

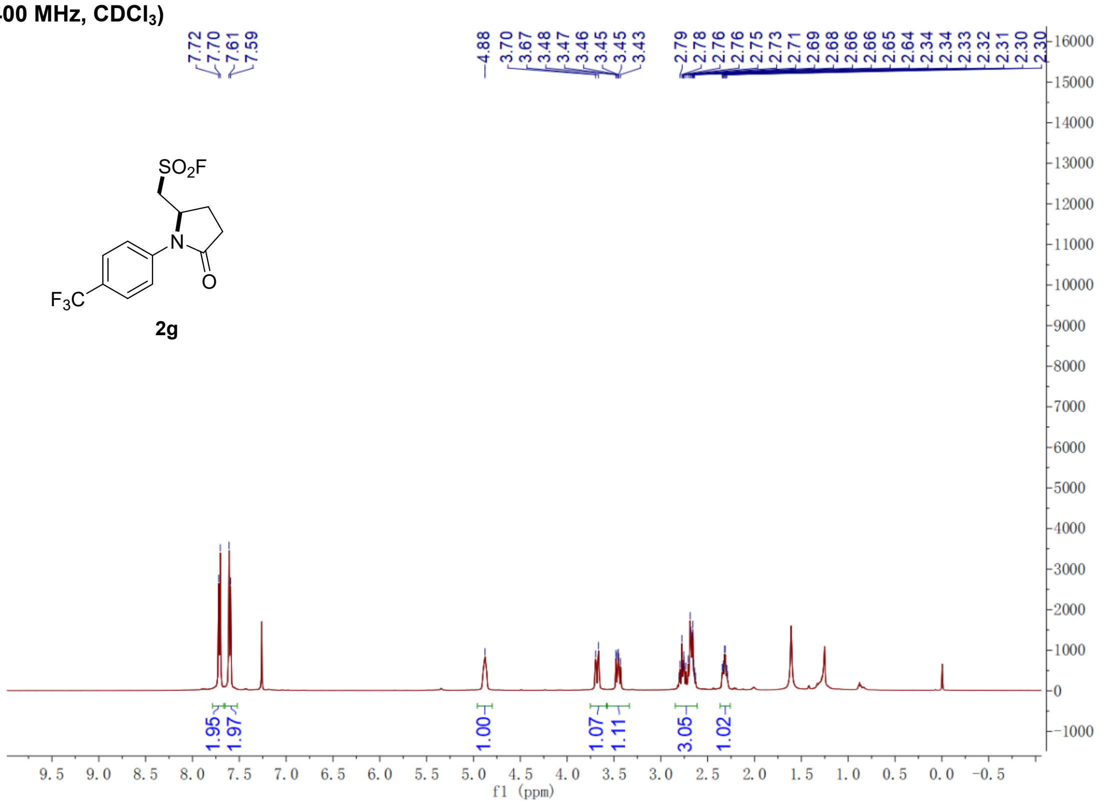

^{13}C-NMR (101 MHz, $CDCl_3$)

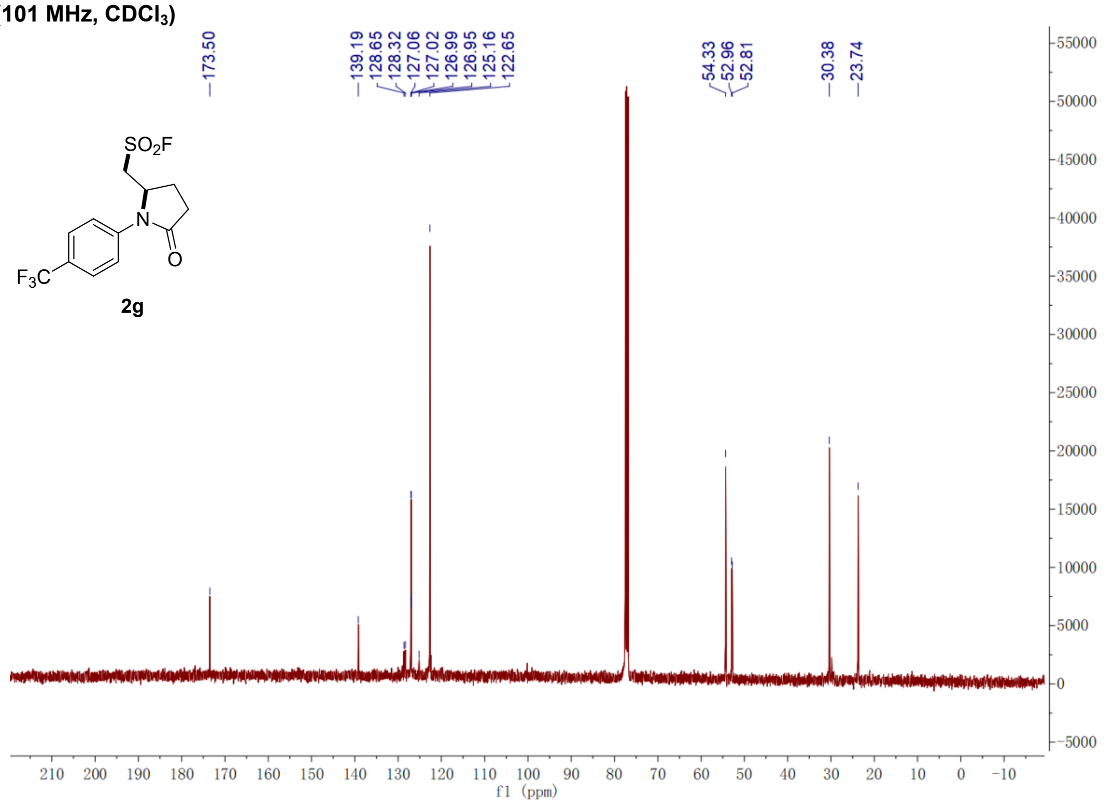

^{19}F NMR (376 MHz, CDCl$_3$)

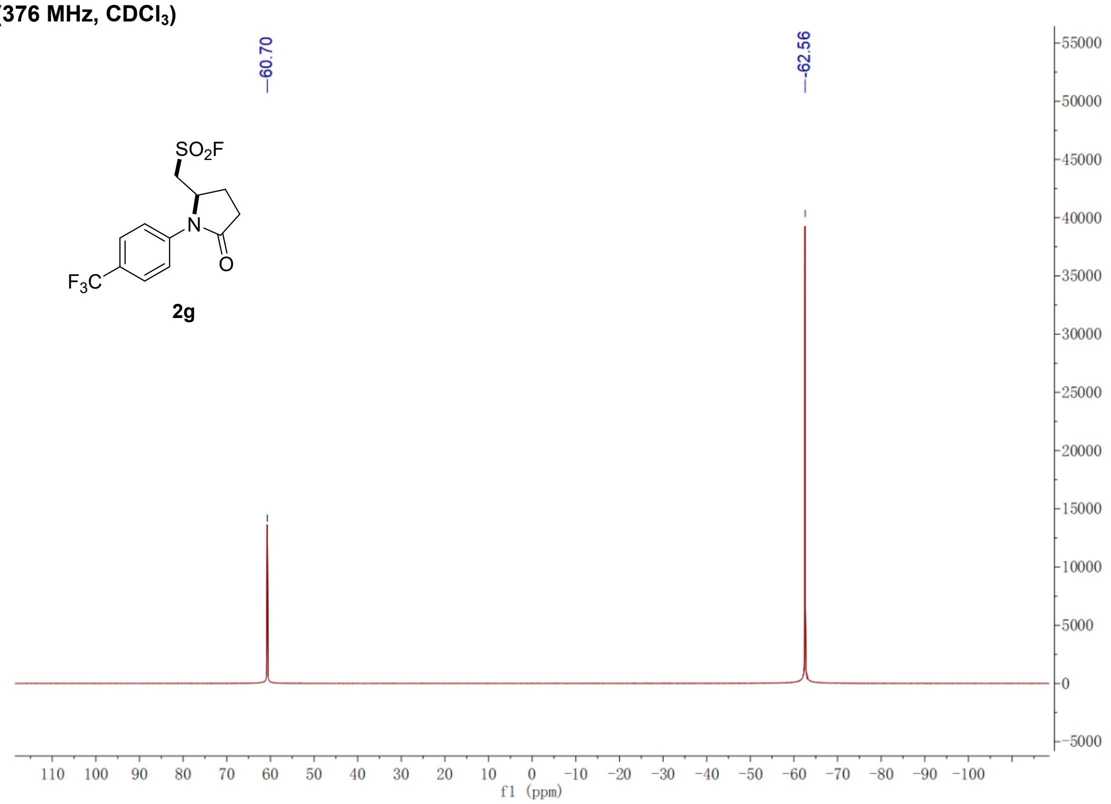

^{1}H-NMR (400 MHz, CDCl$_3$)

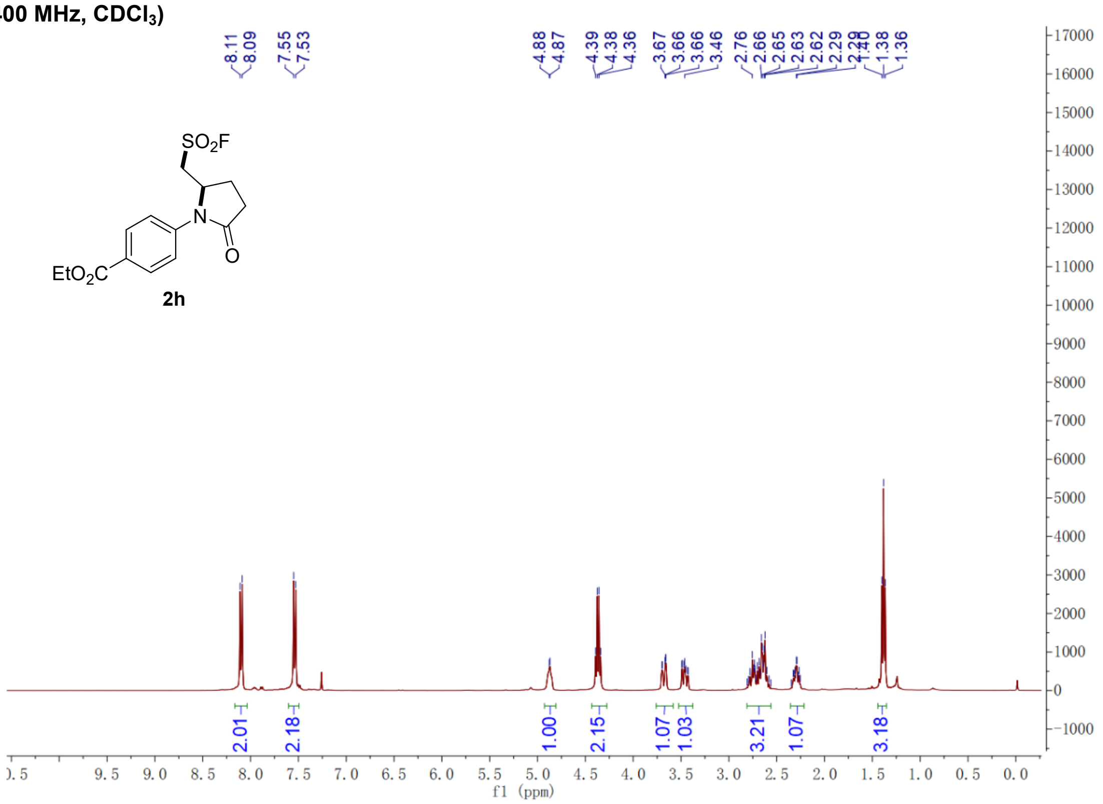

^{13}C-NMR (101 MHz, $CDCl_3$)

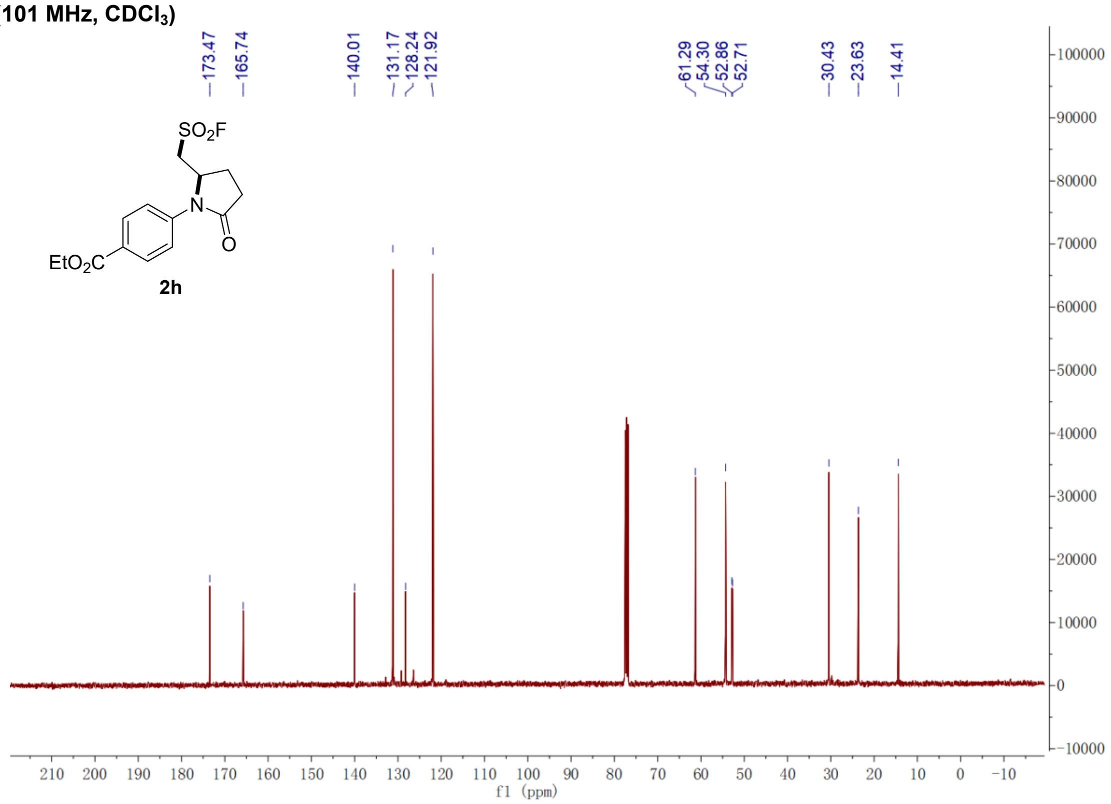

^{19}F NMR (376 MHz, $CDCl_3$)

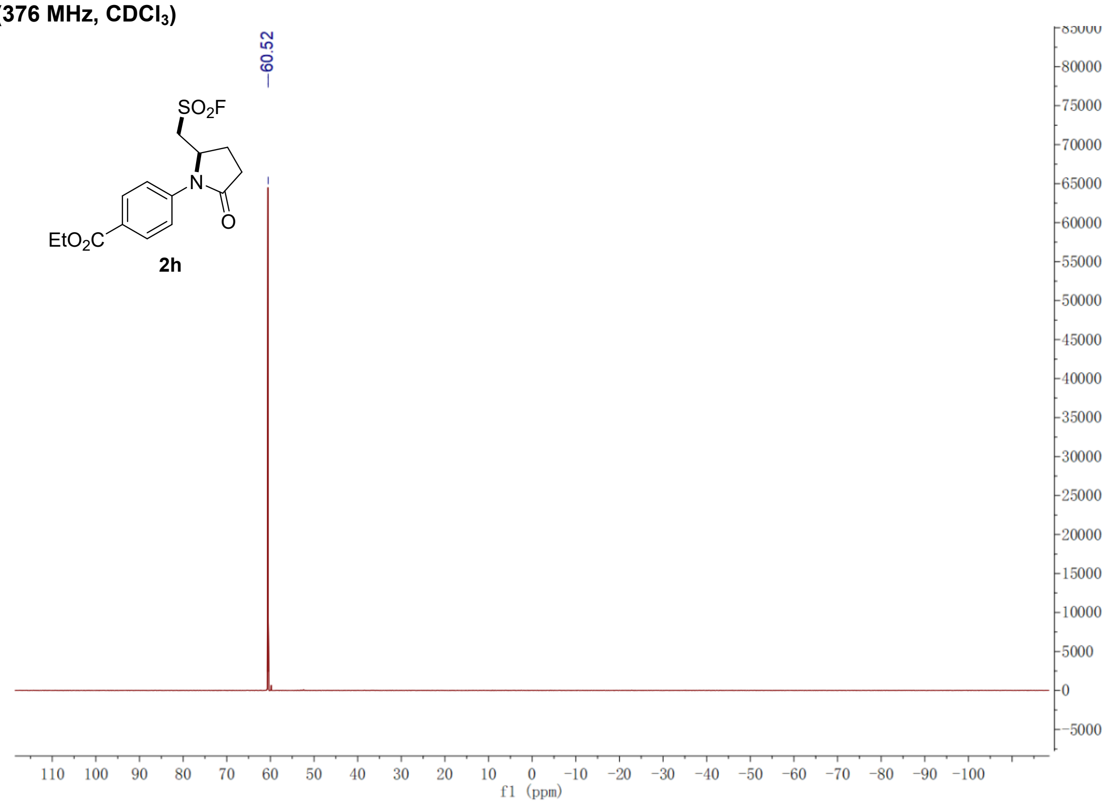

^{1}H-NMR (400 MHz, DMSO-d_6)

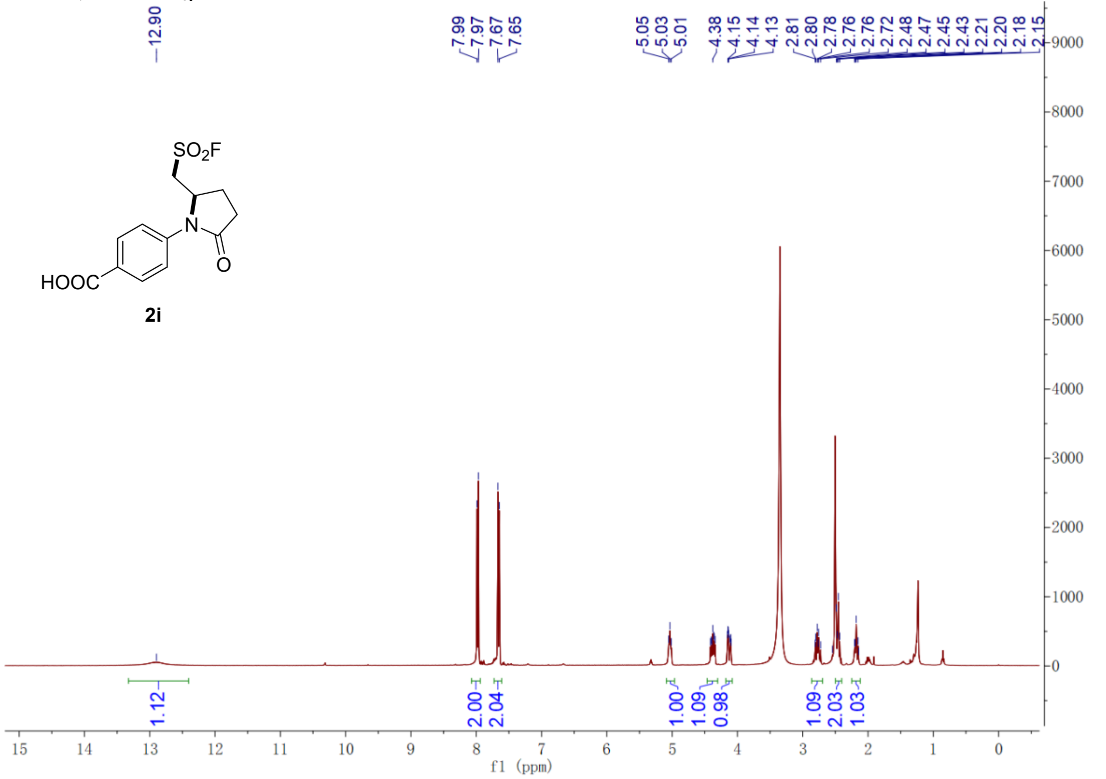

^{13}C-NMR (101 MHz, DMSO-d_6)

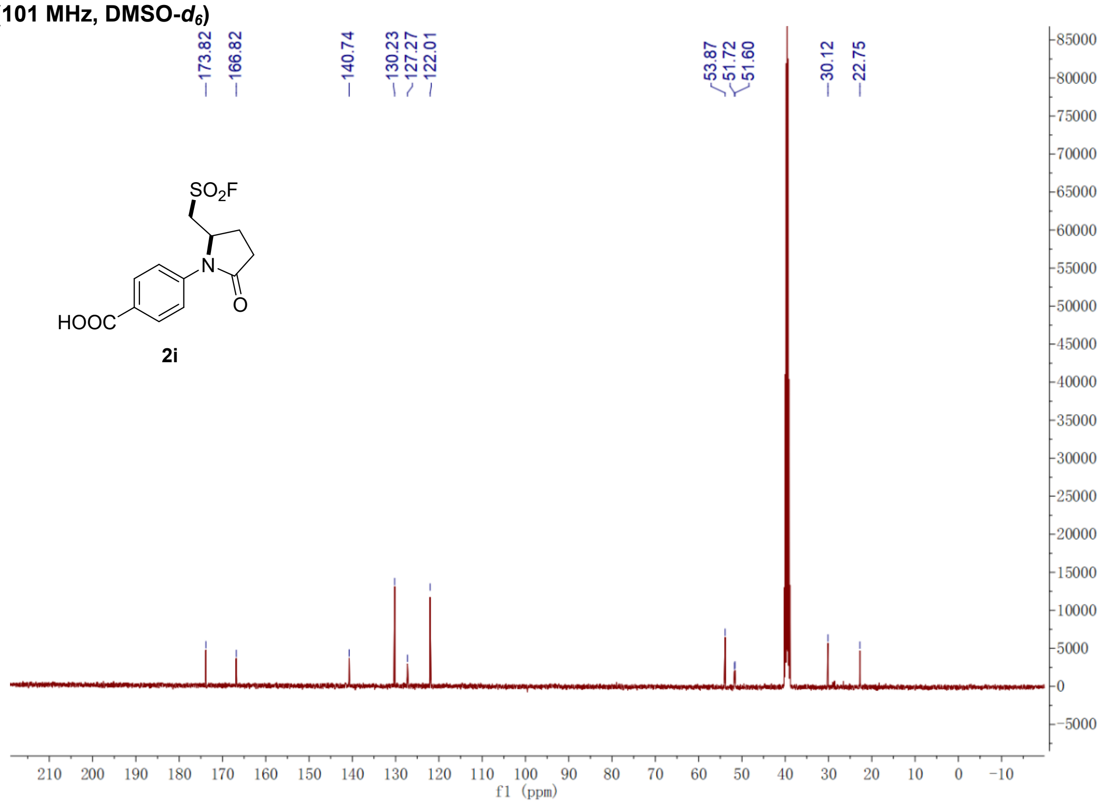

^{19}F NMR (376 MHz, DMSO-d_6)

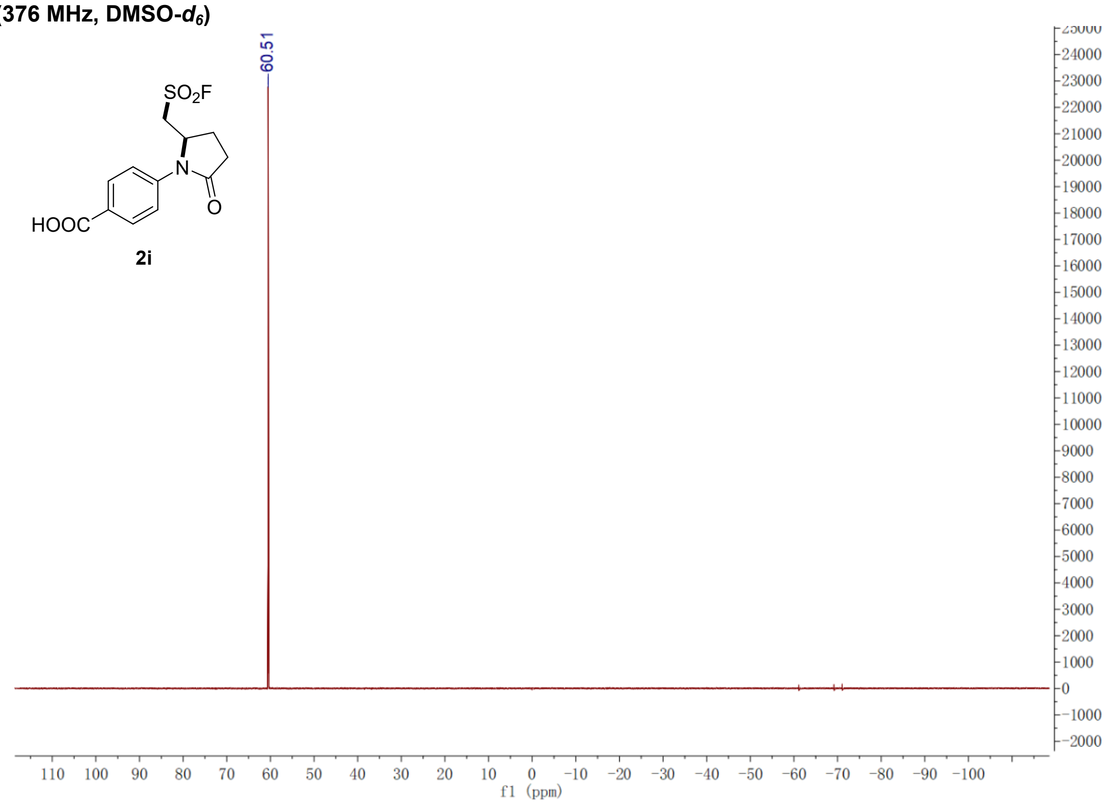

^{1}H-NMR (400 MHz, $CDCl_3$)

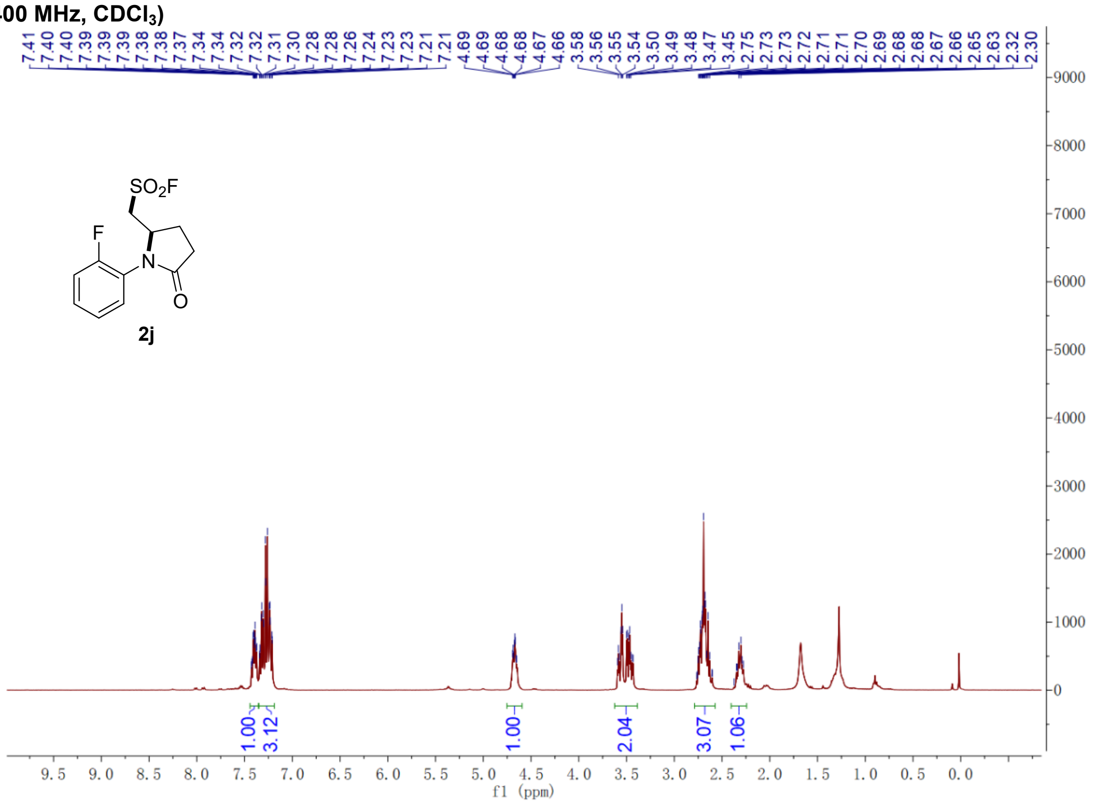

^{13}C-NMR (101 MHz, $CDCl_3$)

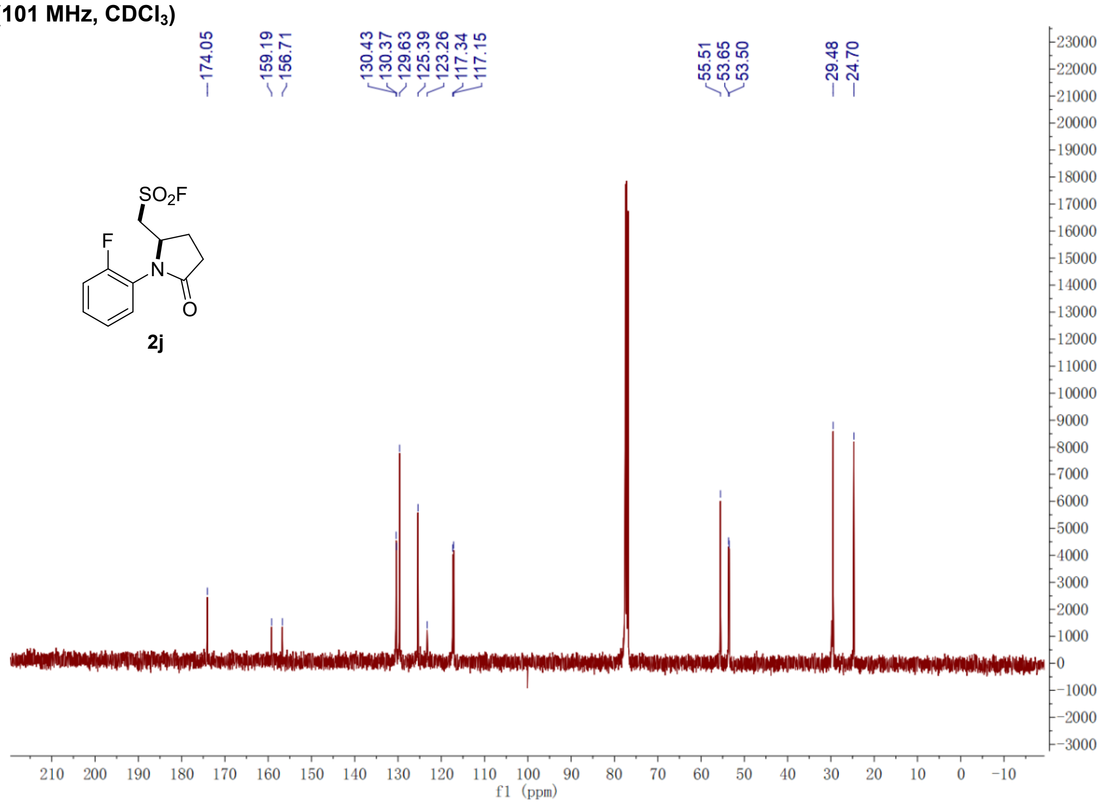

^{19}F NMR (376 MHz, $CDCl_3$)

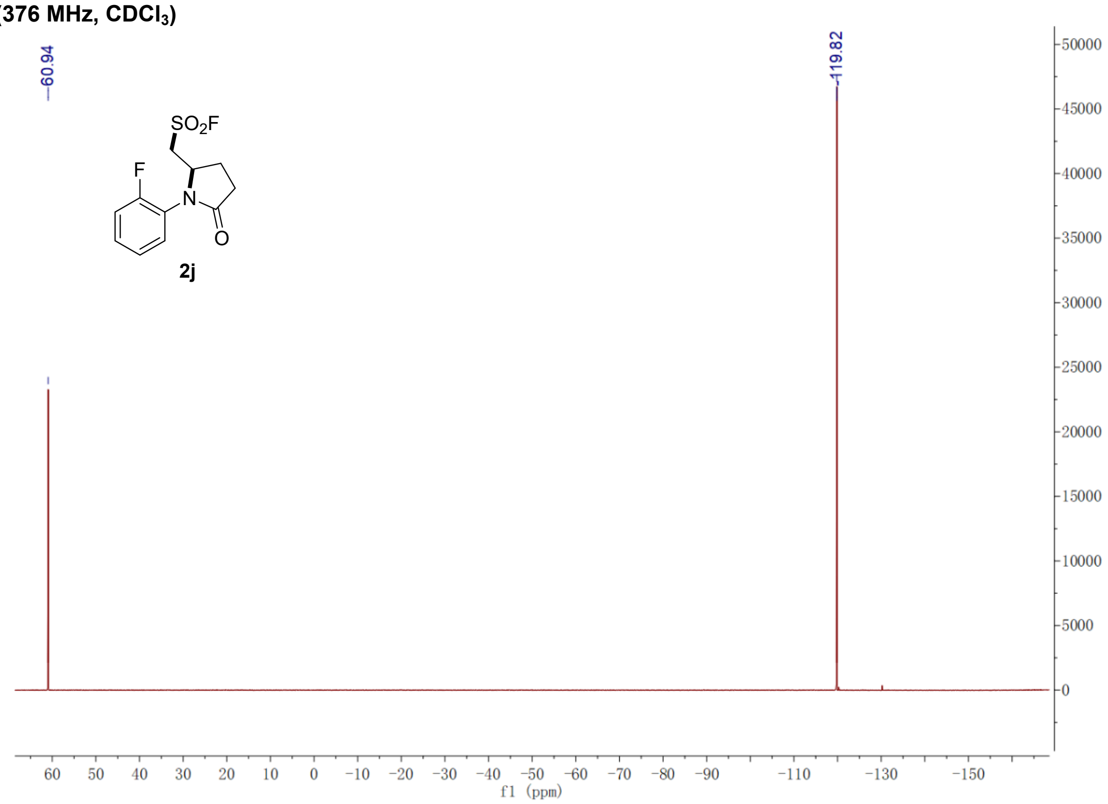

^1H-NMR (400 MHz, $CDCl_3$)

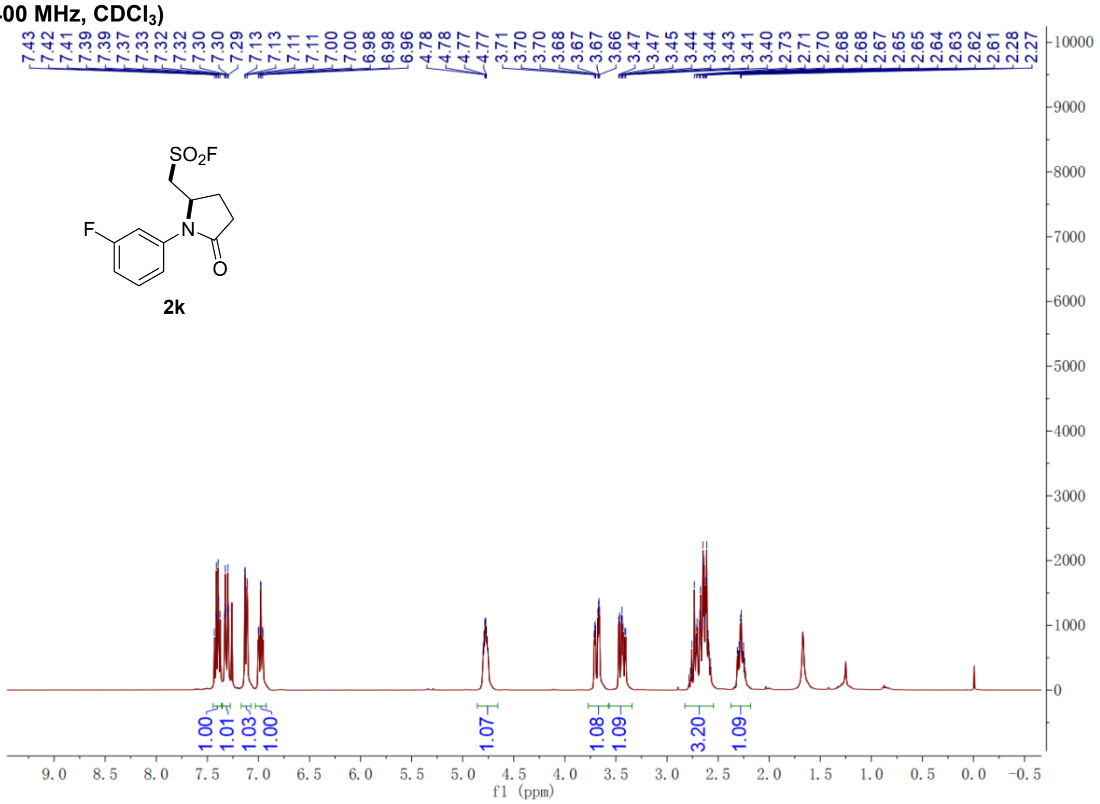

^{13}C-NMR (101 MHz, $CDCl_3$)

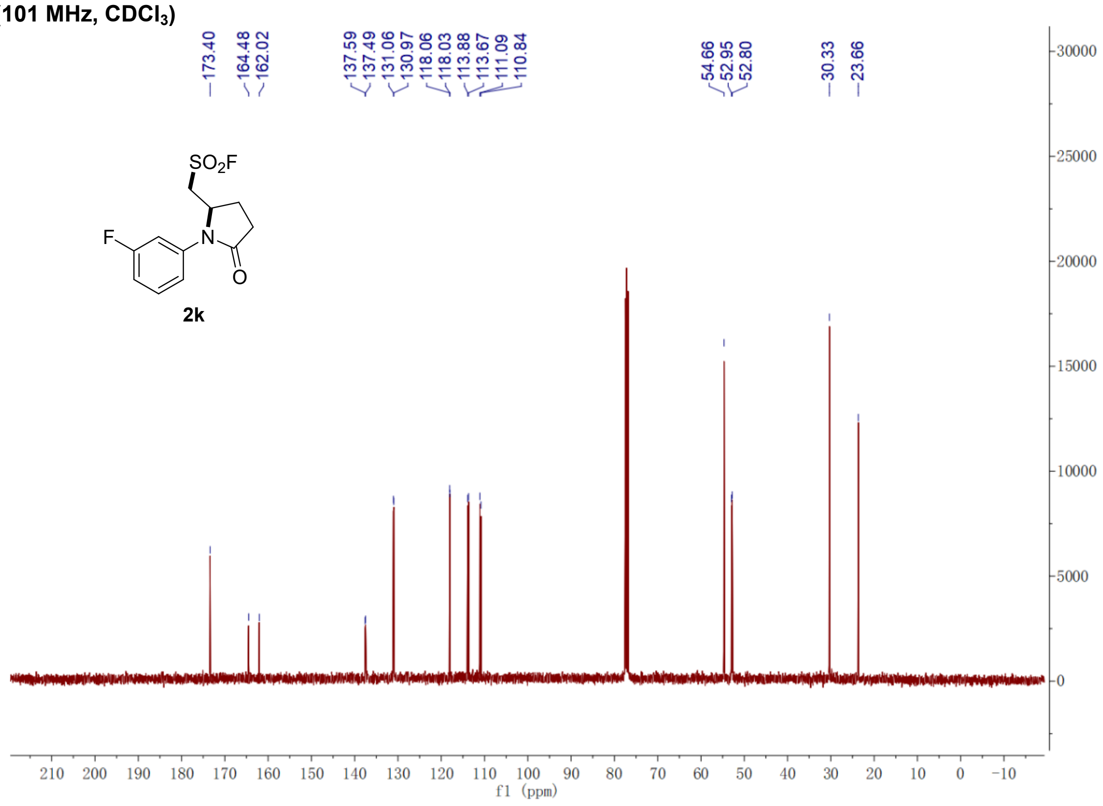

^{19}F NMR (376 MHz, $CDCl_3$)

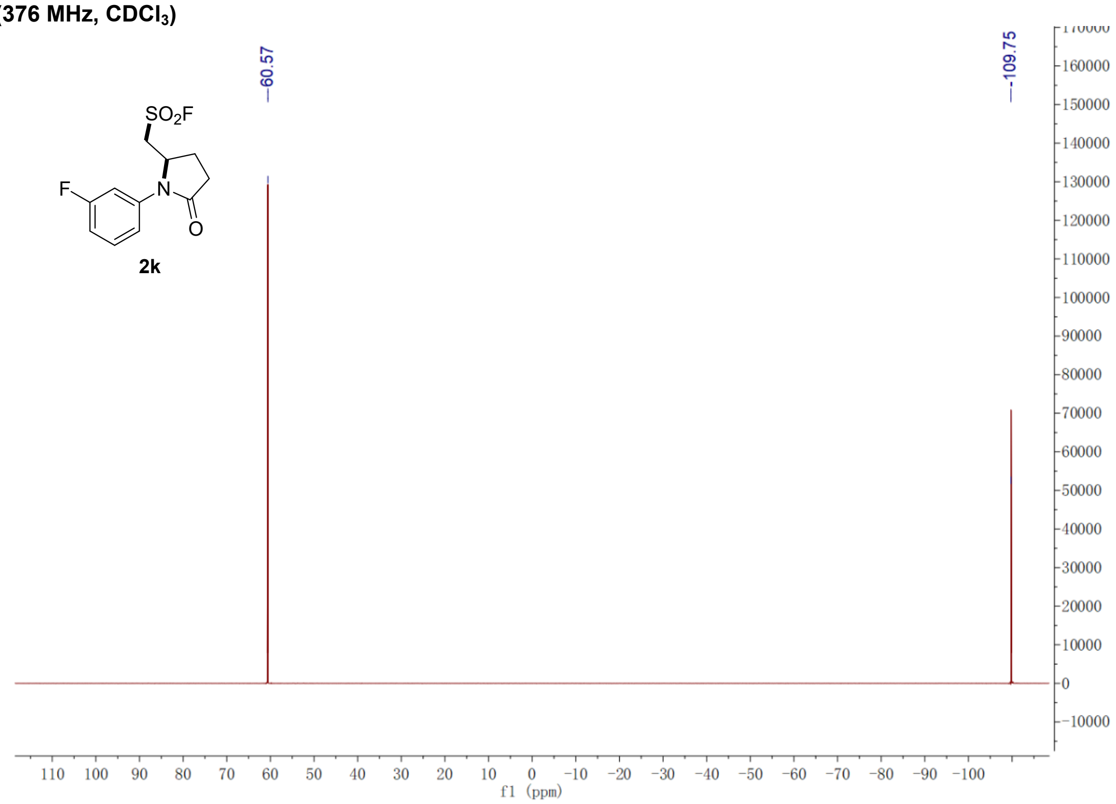

^{1}H-NMR (400 MHz, $CDCl_3$)

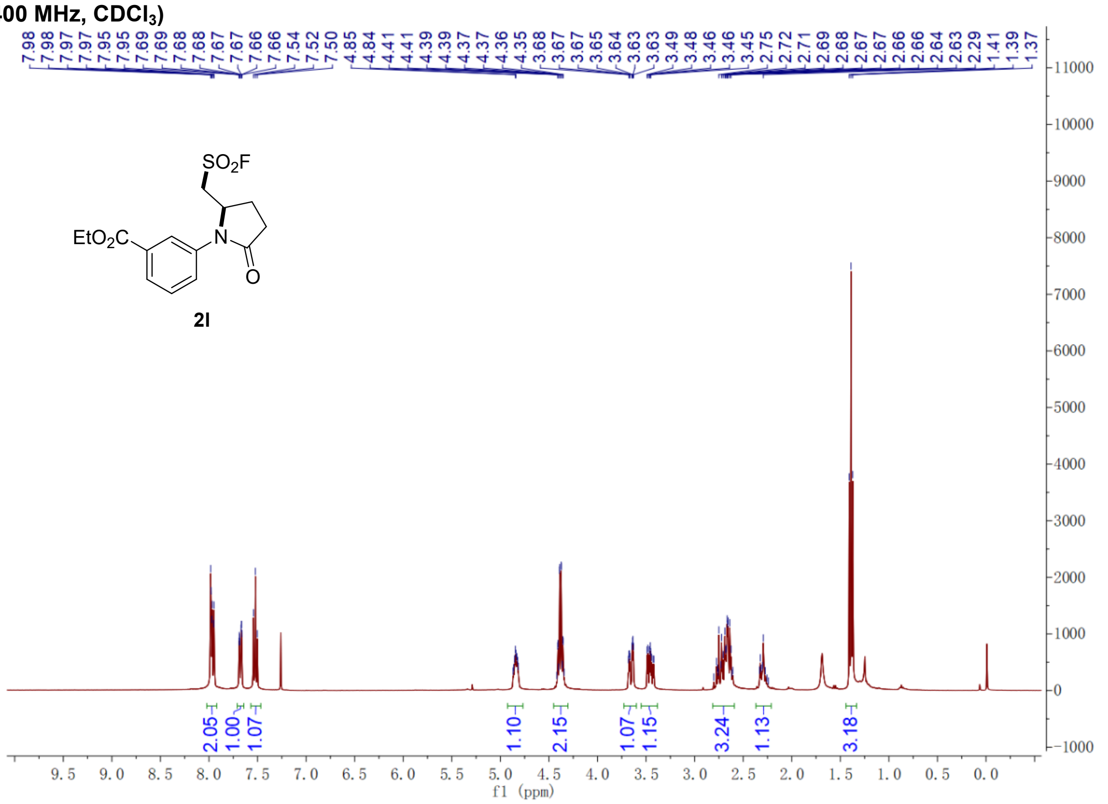

^{13}C-NMR (101 MHz, CDCl$_3$)

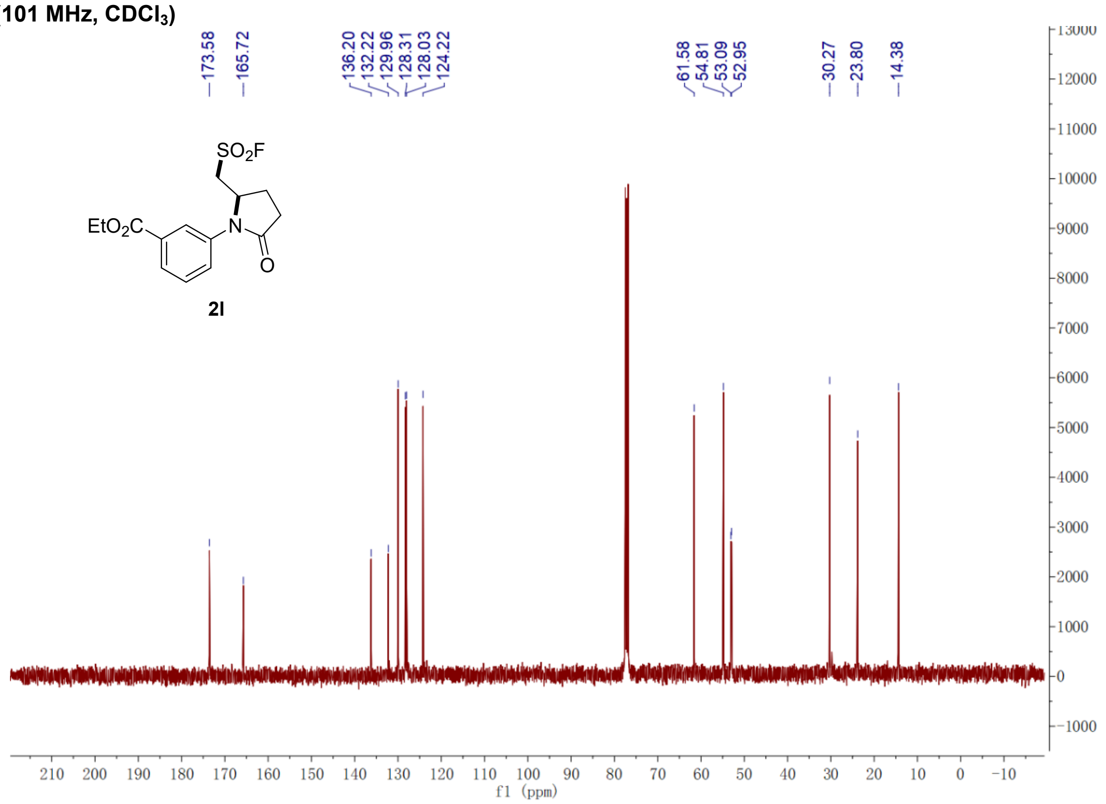

^{19}F NMR (376 MHz, CDCl$_3$)

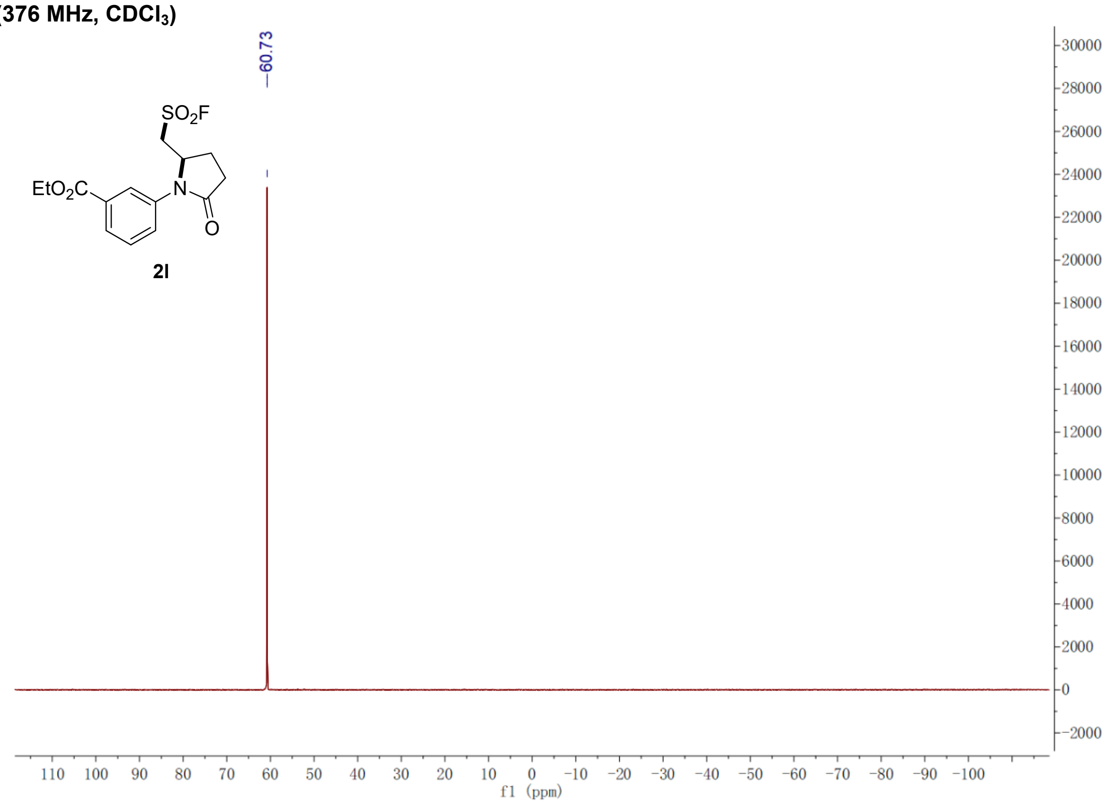

^{1}H-NMR (400 MHz, $CDCl_3$)

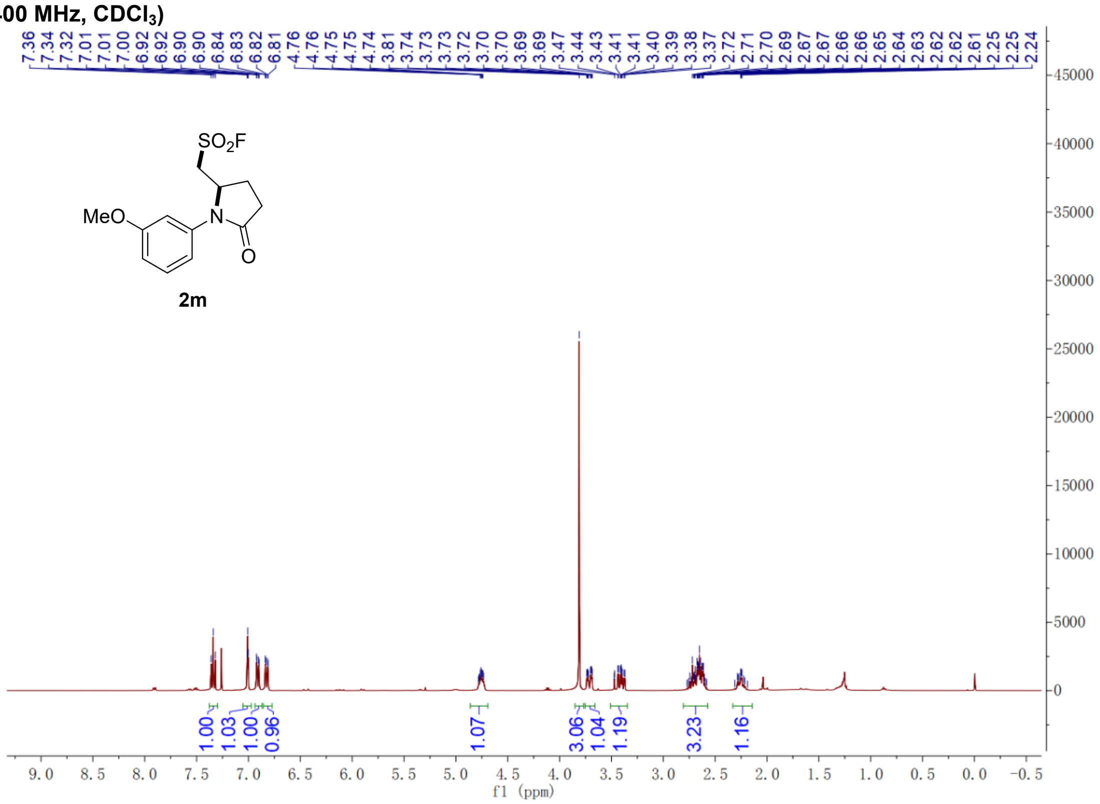

^{13}C-NMR (101 MHz, $CDCl_3$)

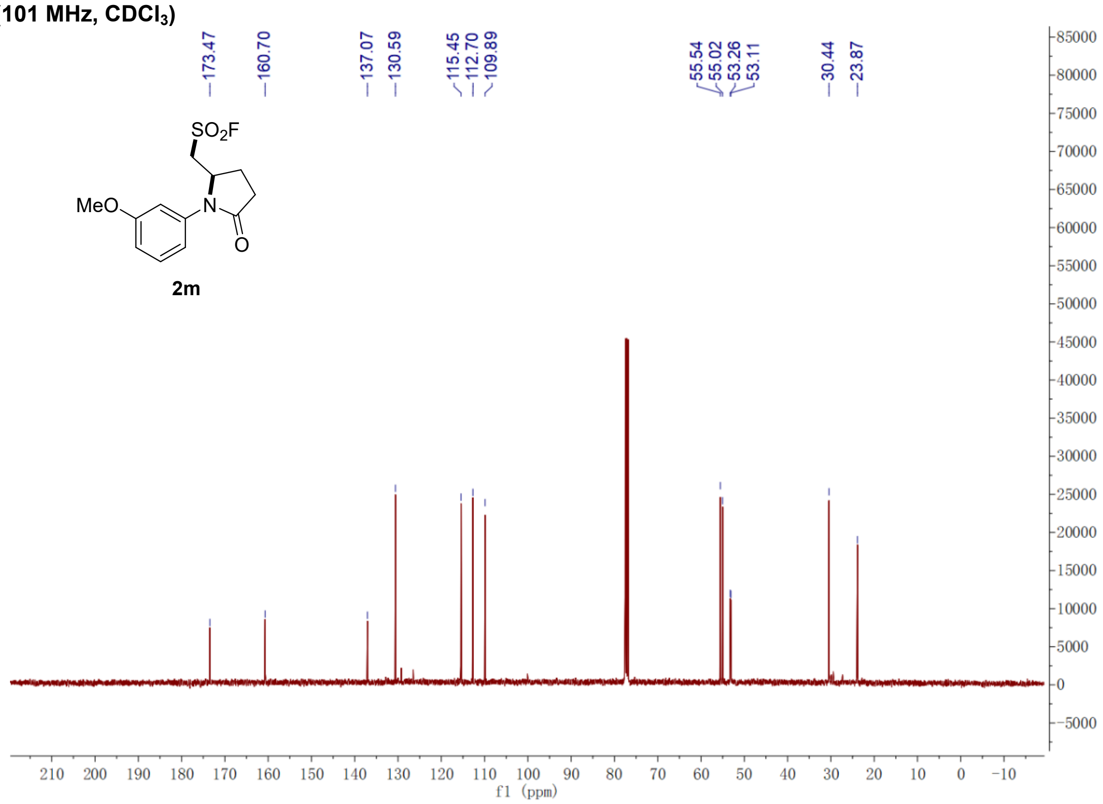

^{19}F NMR (376 MHz, CDCl$_3$)

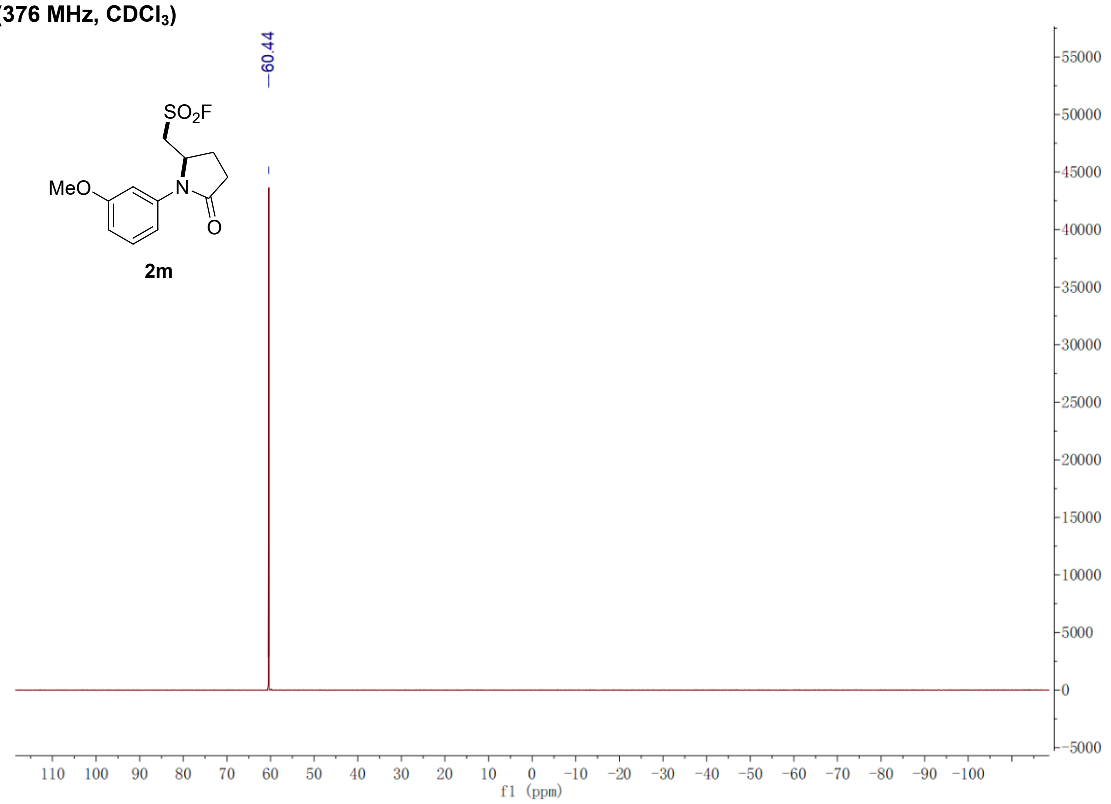

^{1}H-NMR (400 MHz, CDCl$_3$)

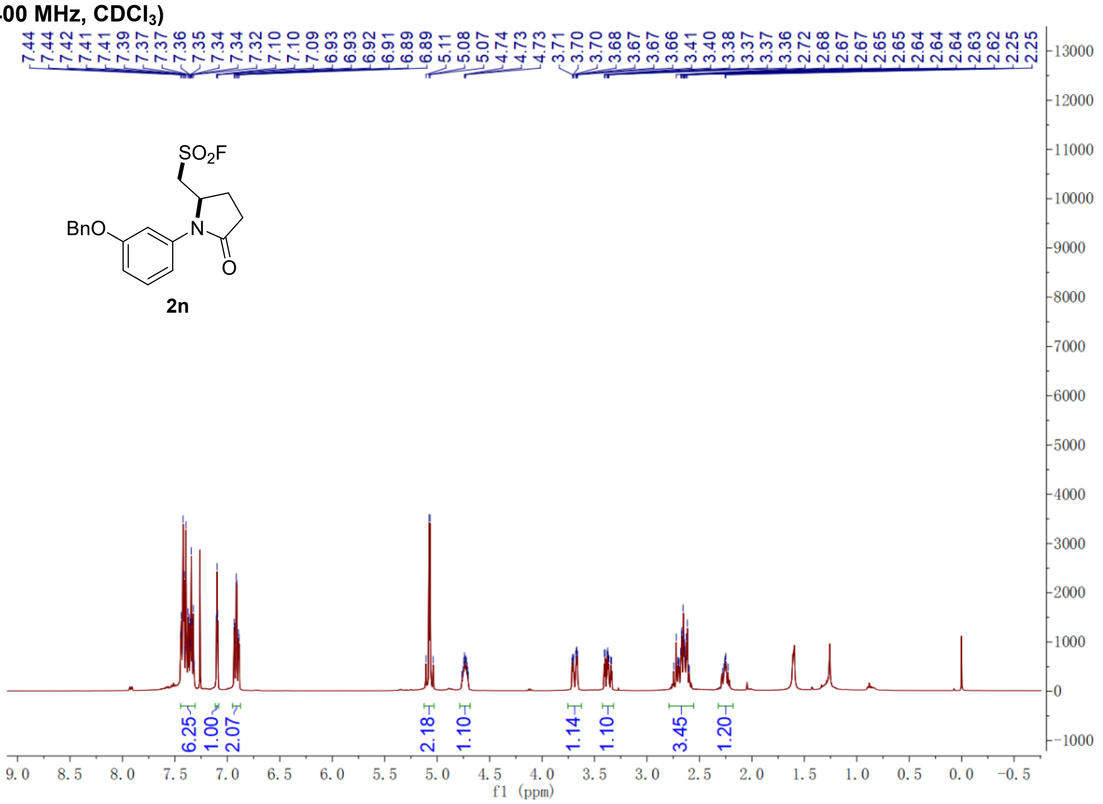

^{13}C-NMR (101 MHz, $CDCl_3$)

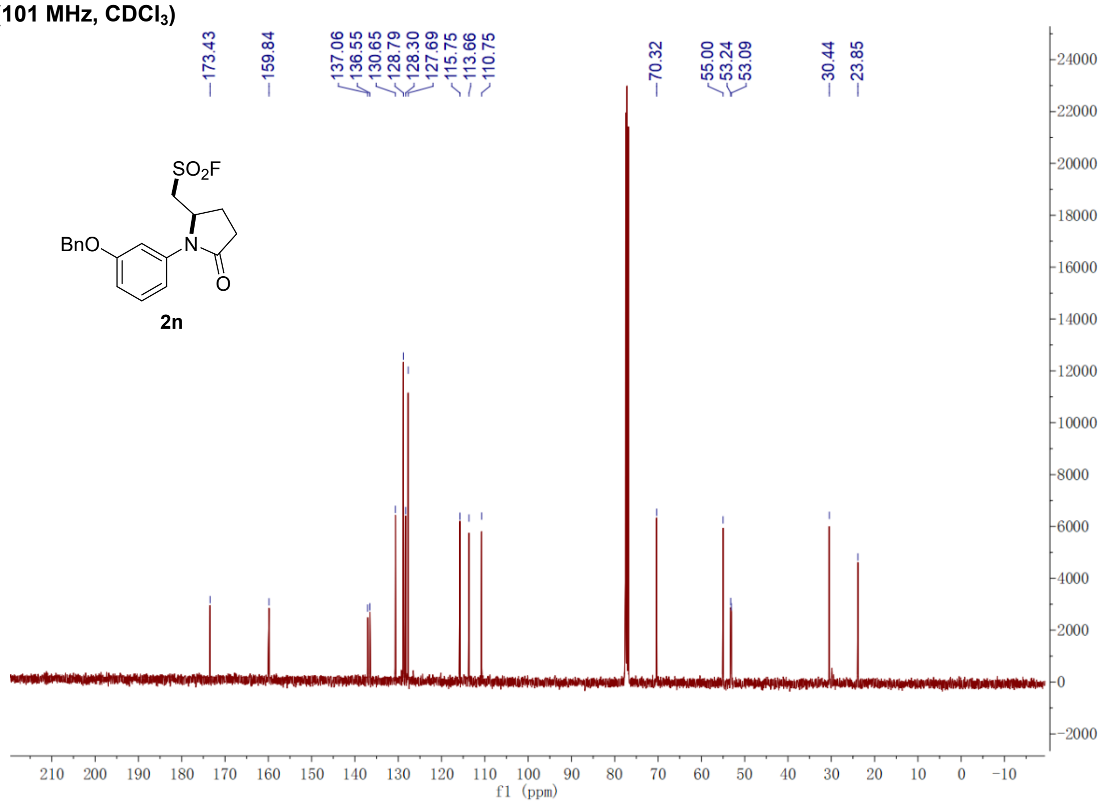

^{19}F NMR (376 MHz, $CDCl_3$)

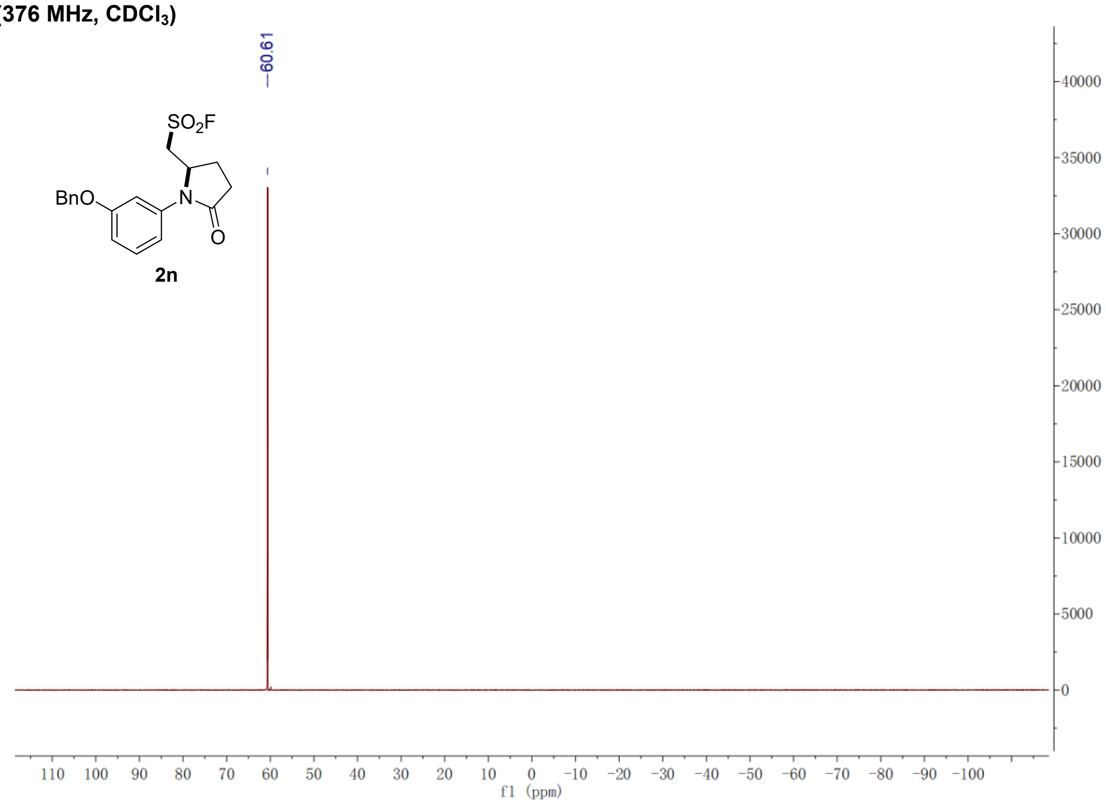

^{1}H-NMR (400 MHz, $CDCl_3$)

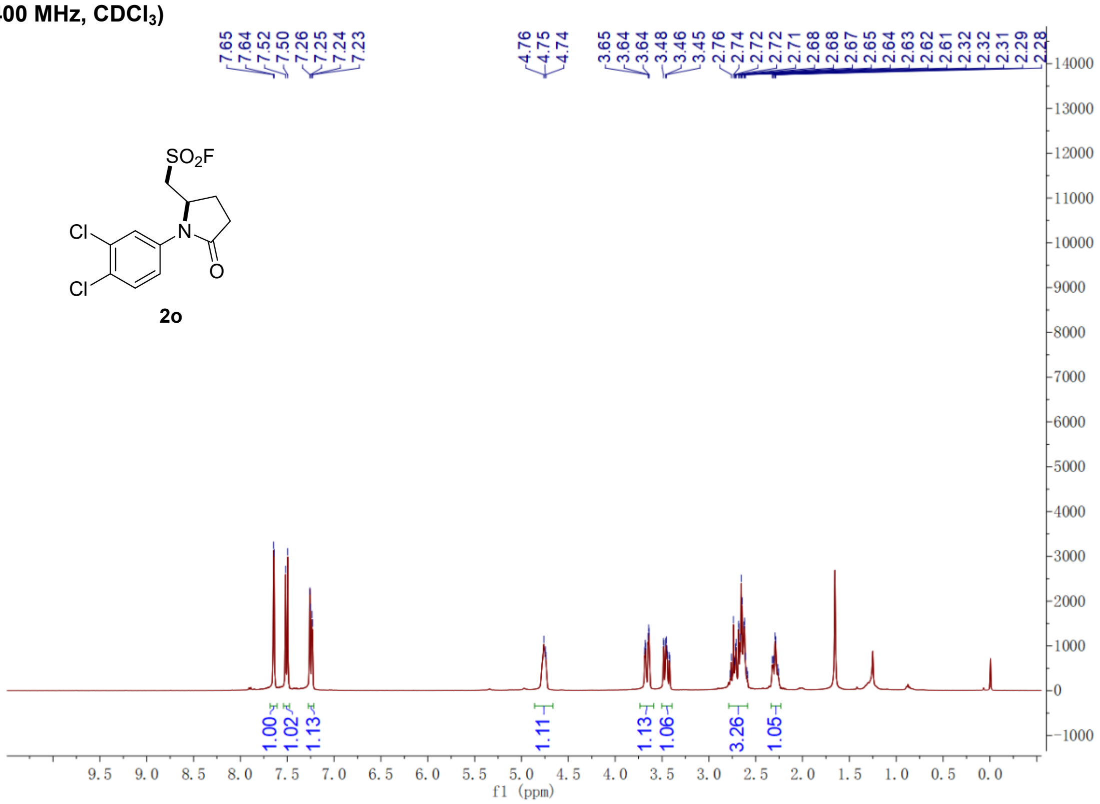

^{13}C-NMR (126 MHz, $CDCl_3$)

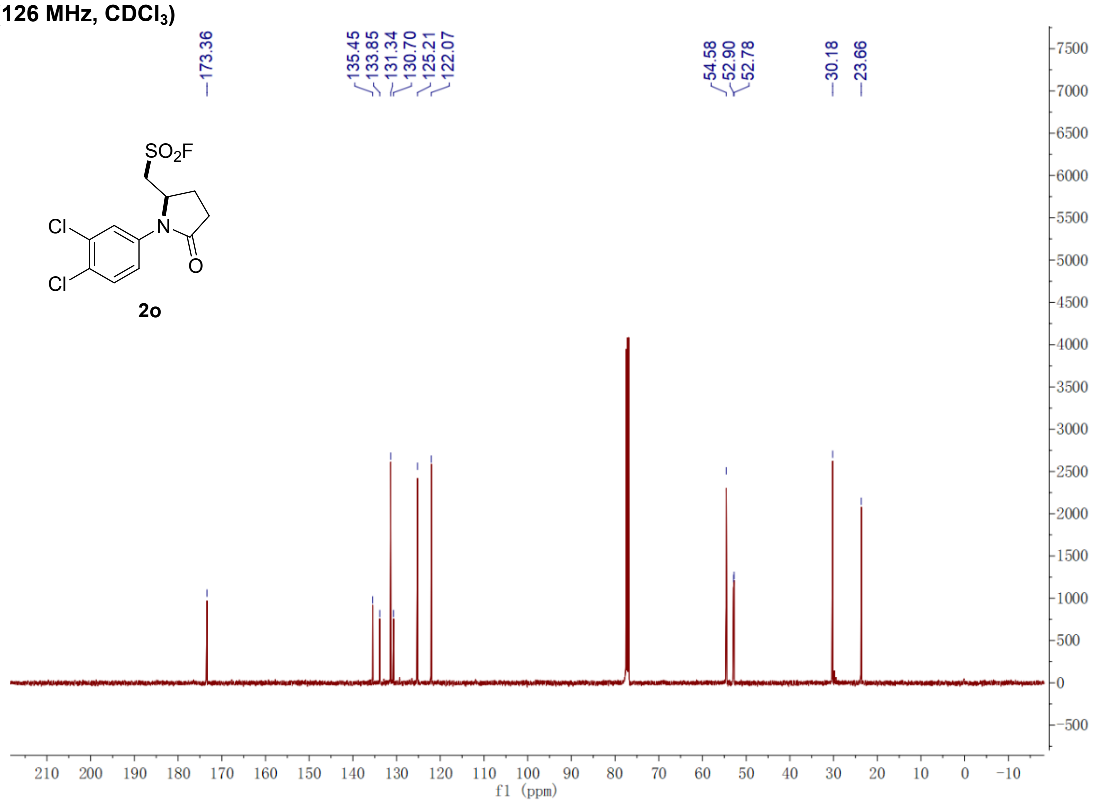

^{19}F NMR (376 MHz, $CDCl_3$)

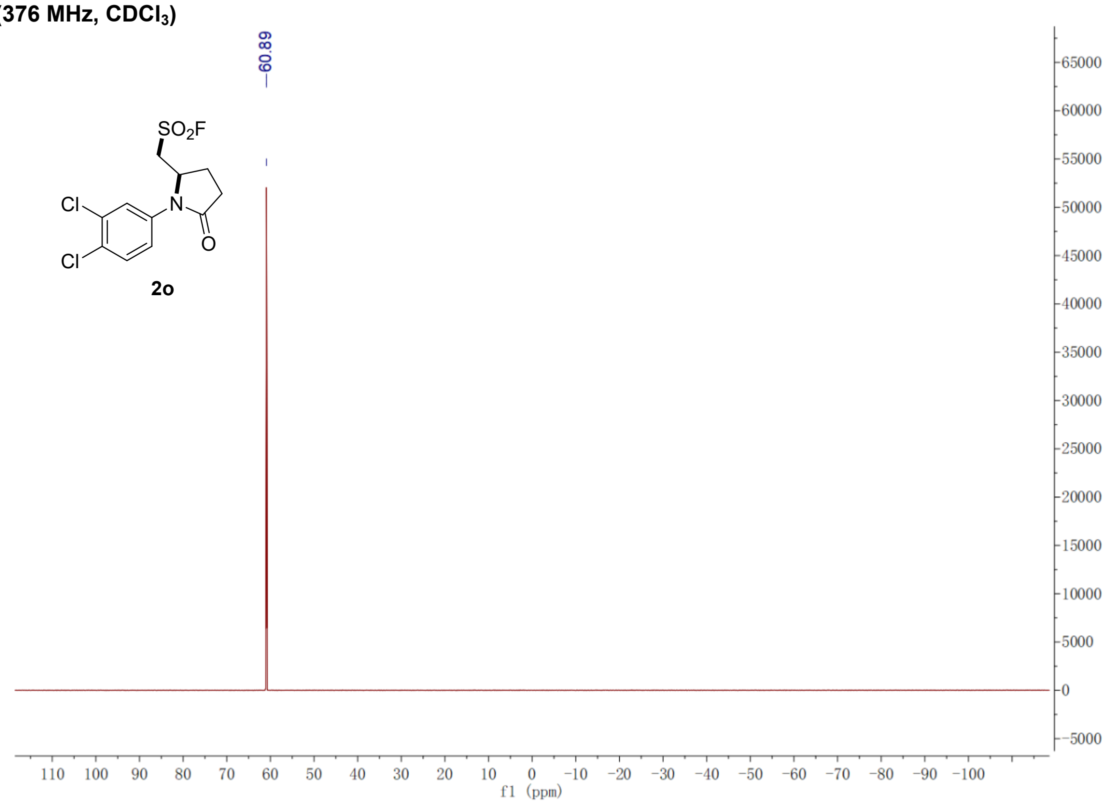

^{1}H-NMR (400 MHz, DMSO-d_6)

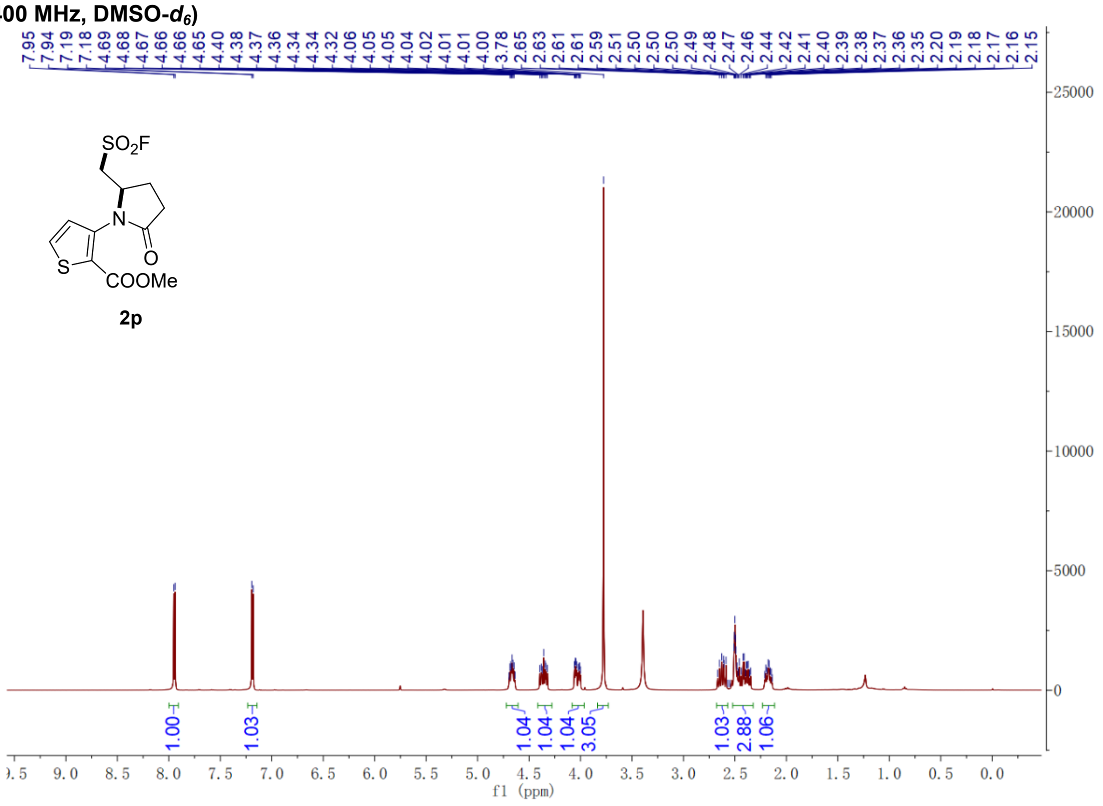

^{13}C-NMR (126 MHz, DMSO-d_6)

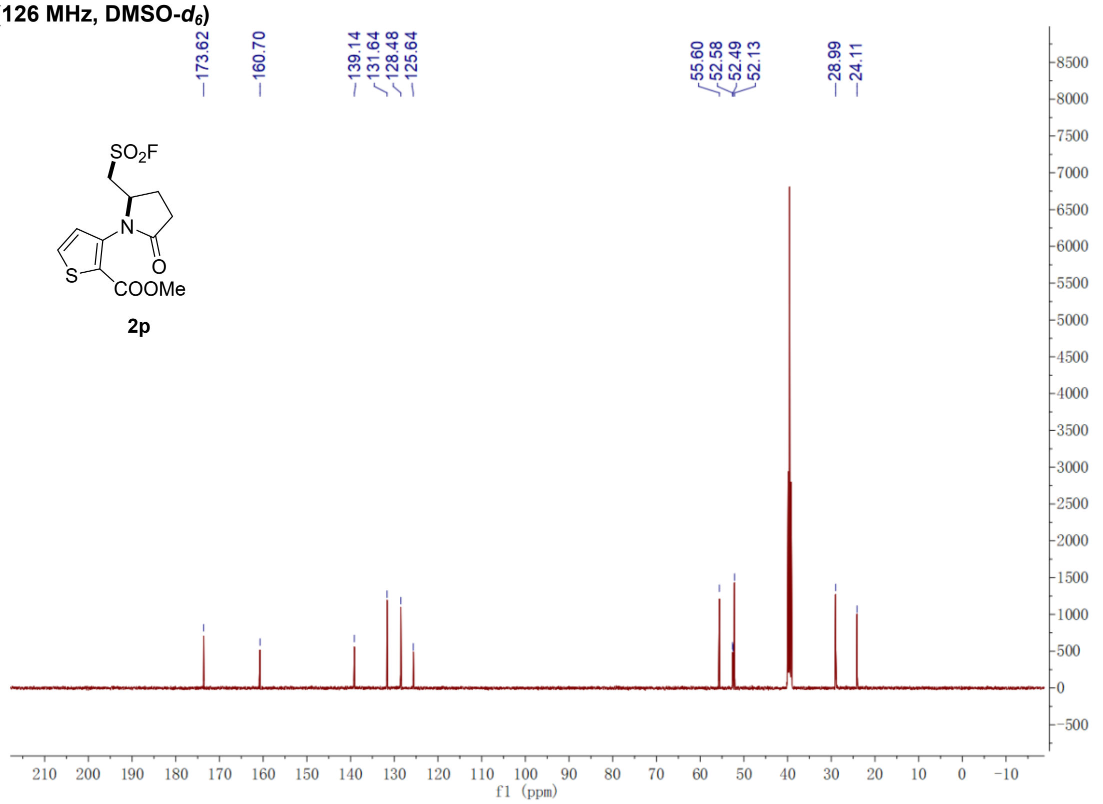

^{19}F NMR (376 MHz, DMSO-d_6)

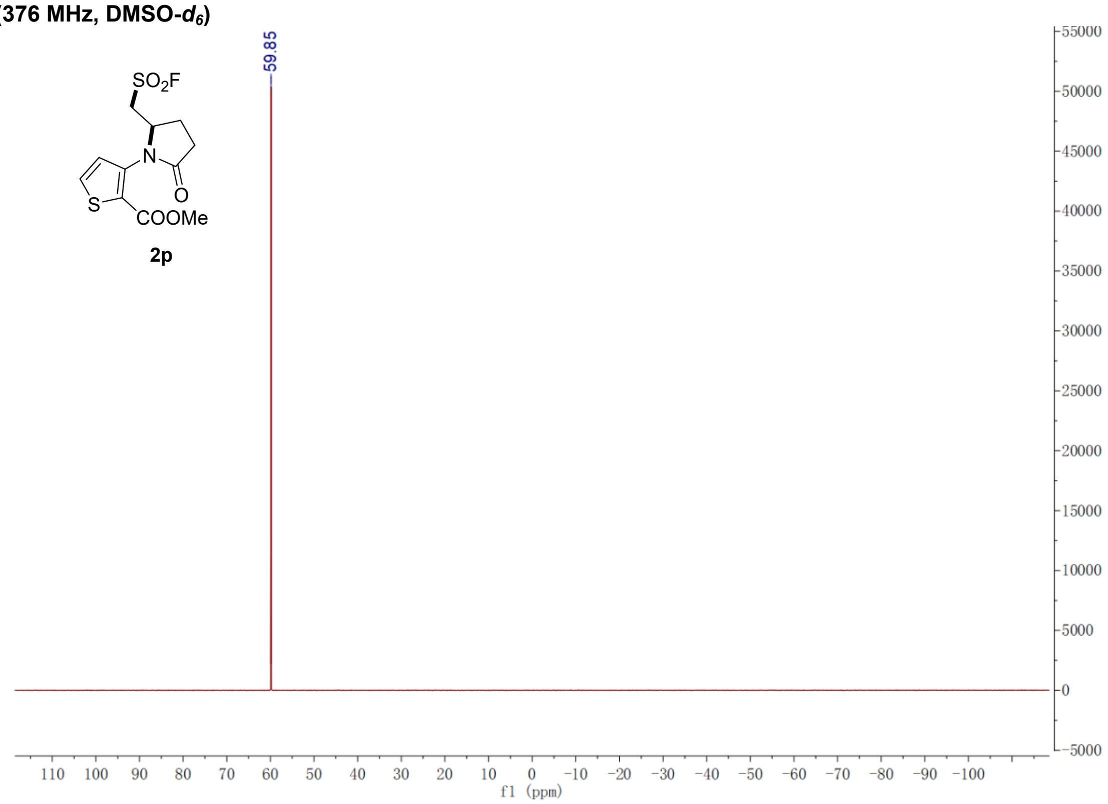

^{1}H-NMR (400 MHz, DMSO-d_6)

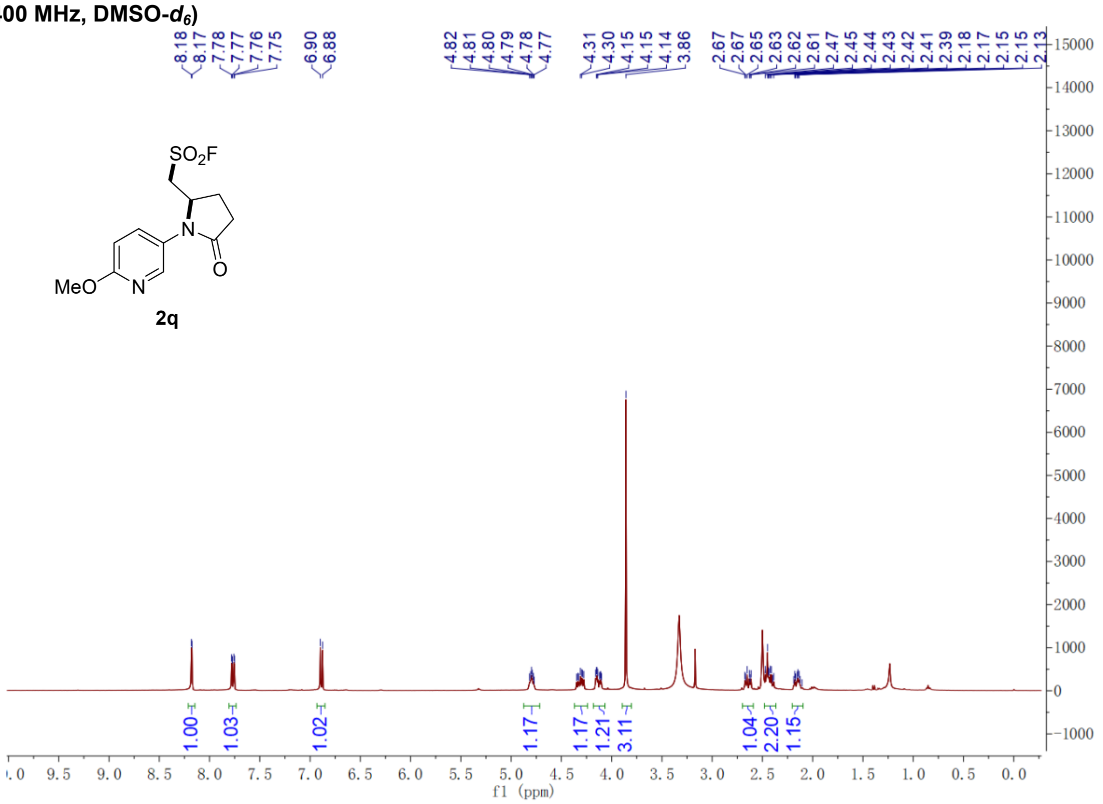

^{13}C-NMR (101 MHz, DMSO-d_6)

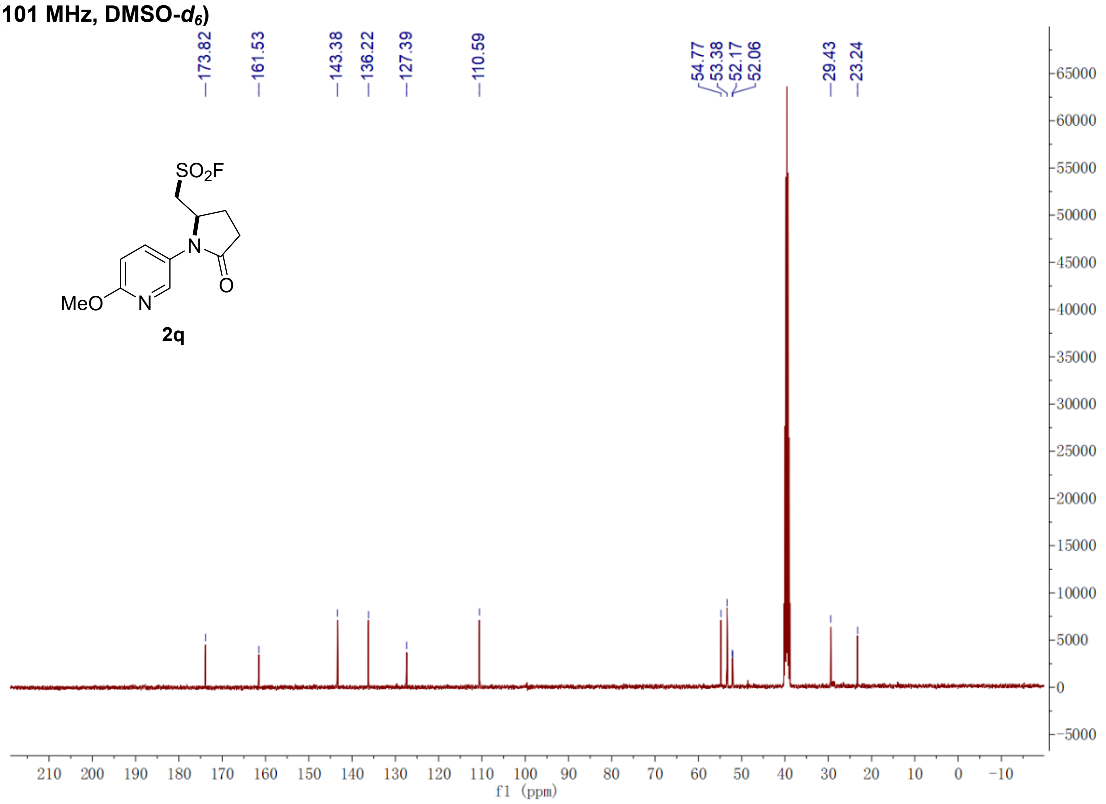

^{19}F NMR (376 MHz, DMSO-d_6)

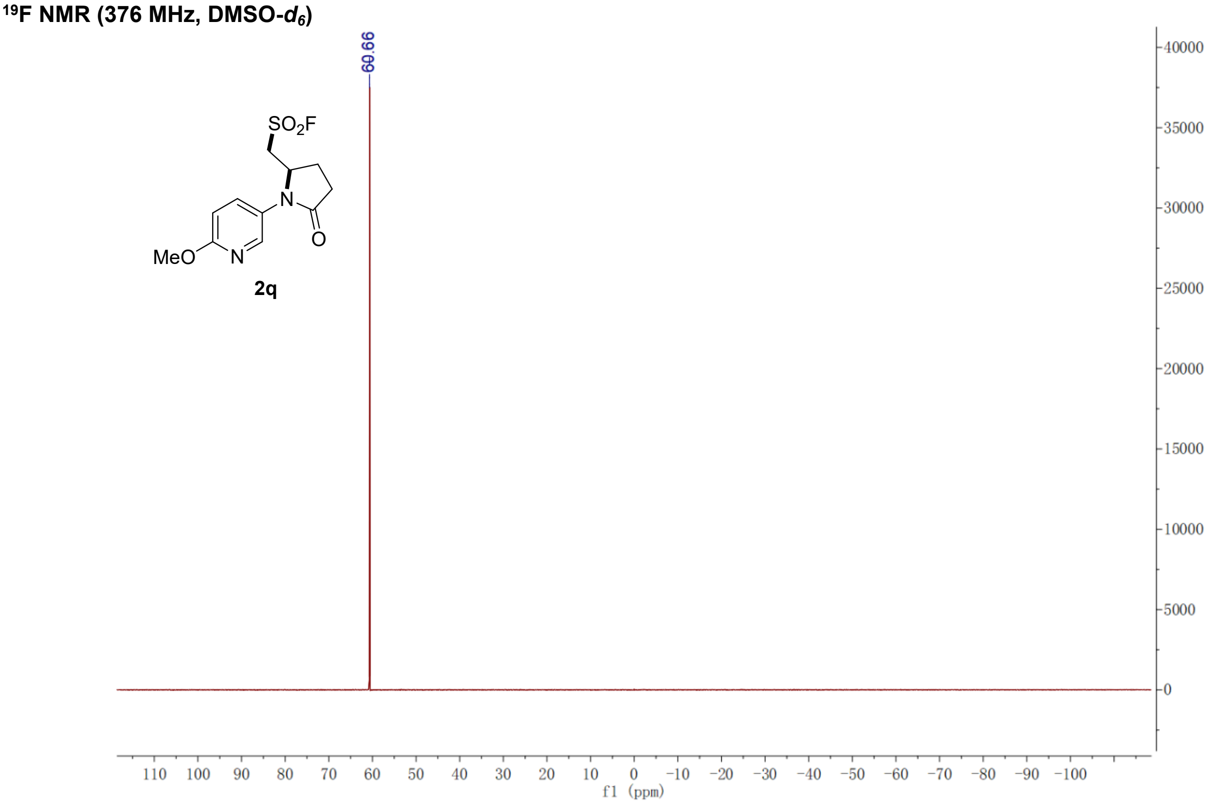

^{1}H-NMR (400 MHz, CDCl$_3$)

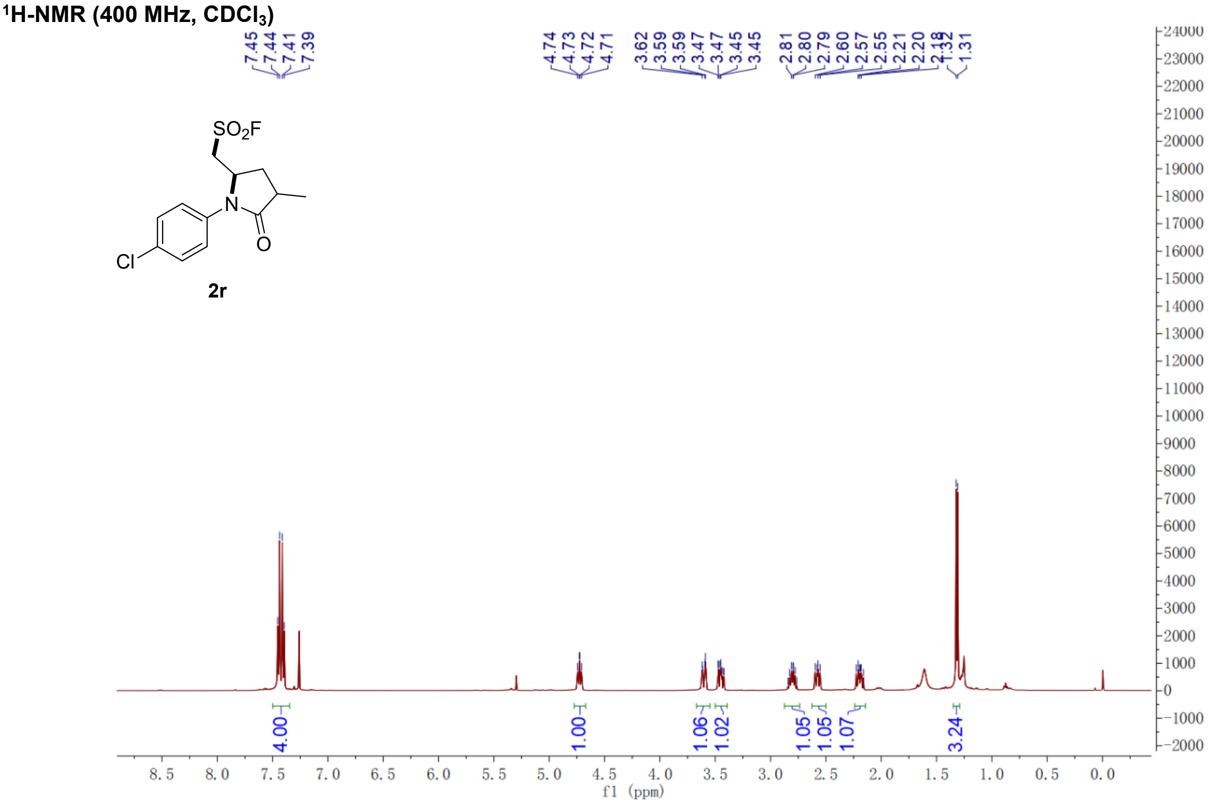

^{13}C-NMR (101 MHz, $CDCl_3$)

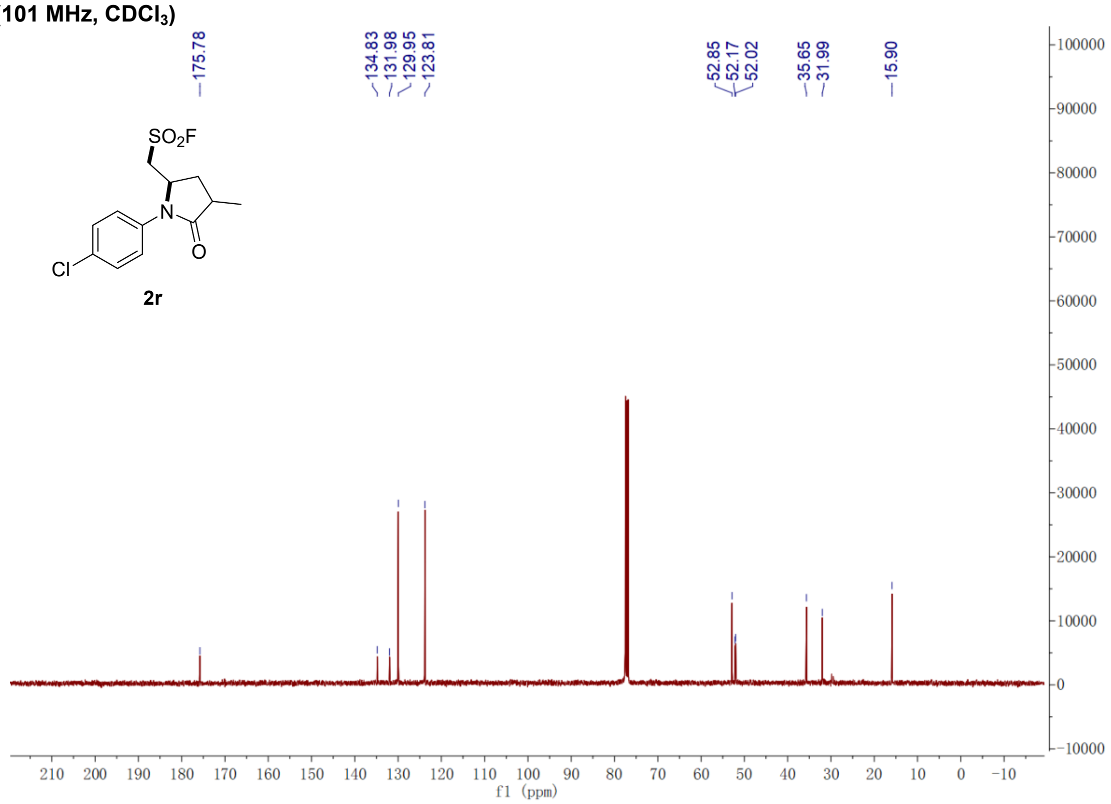

^{19}F NMR (376 MHz, $CDCl_3$)

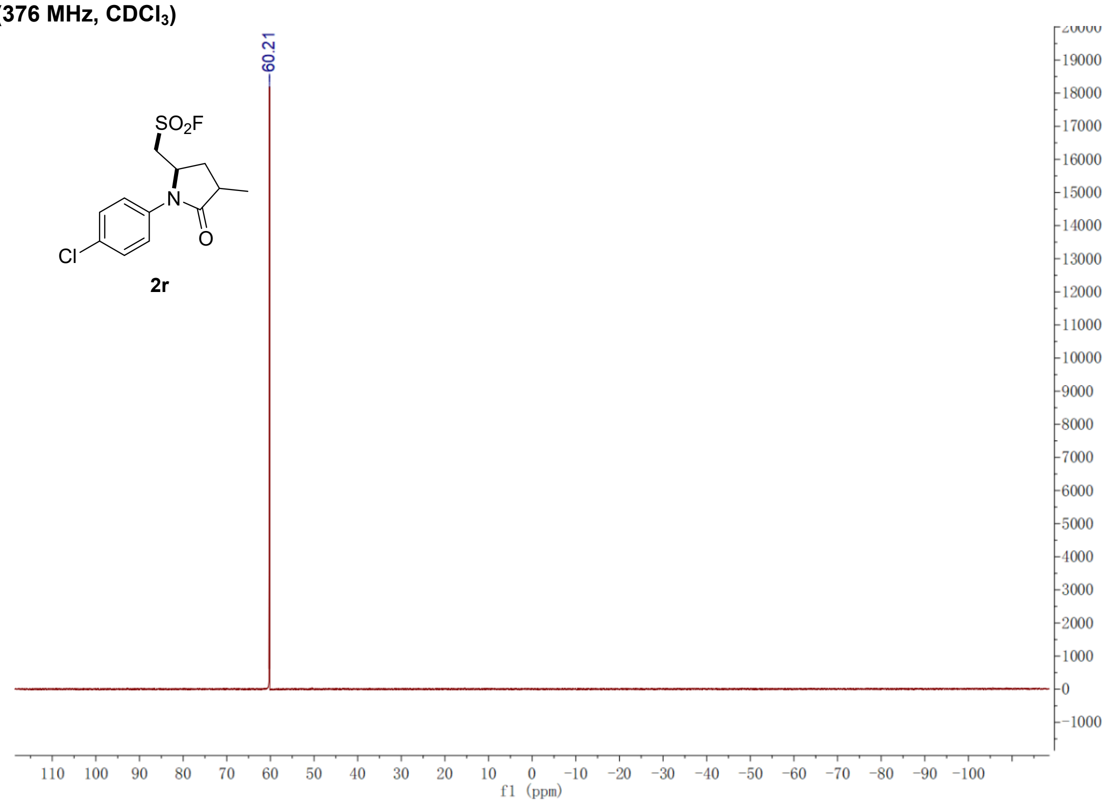

^{1}H-NMR (400 MHz, $CDCl_3$)

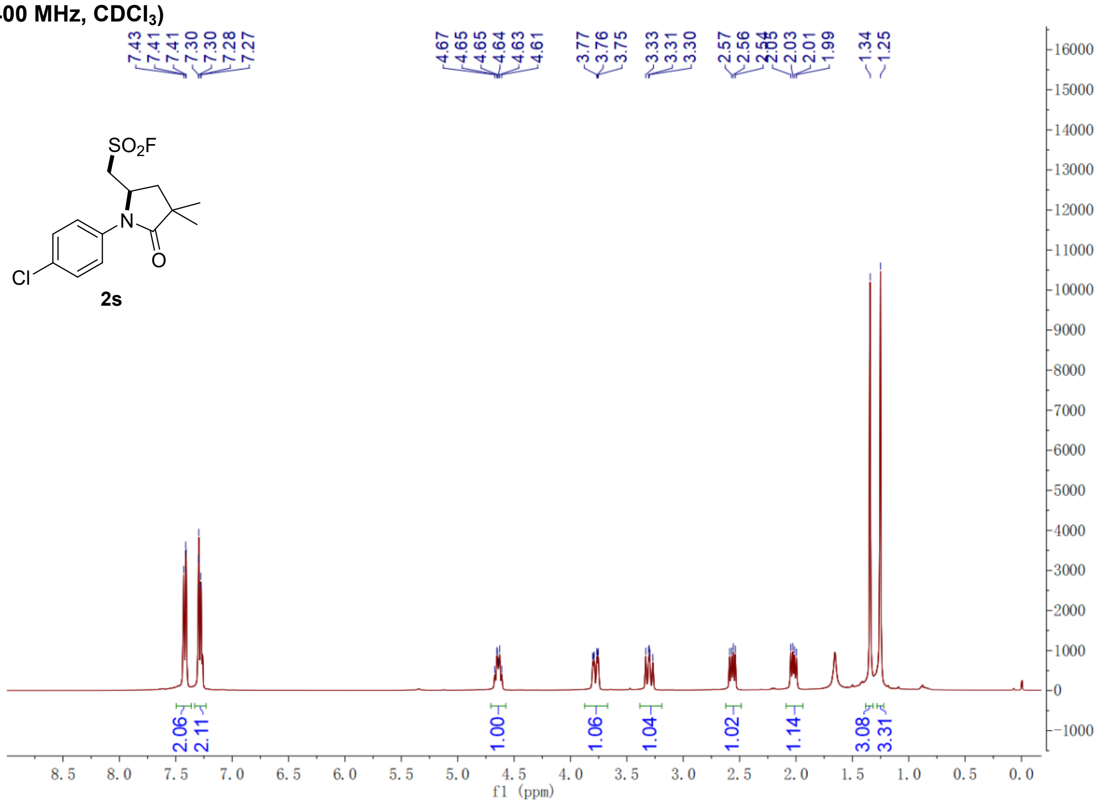

^{13}C-NMR (101 MHz, $CDCl_3$)

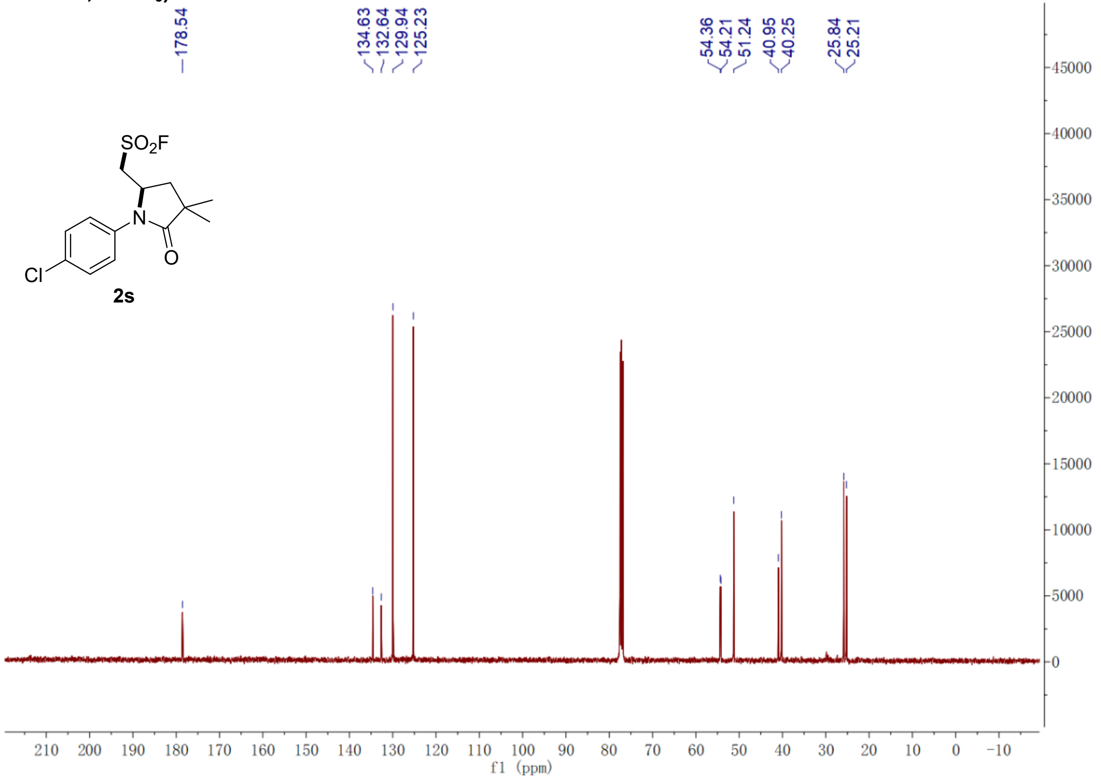

^{19}F NMR (376 MHz, $CDCl_3$)

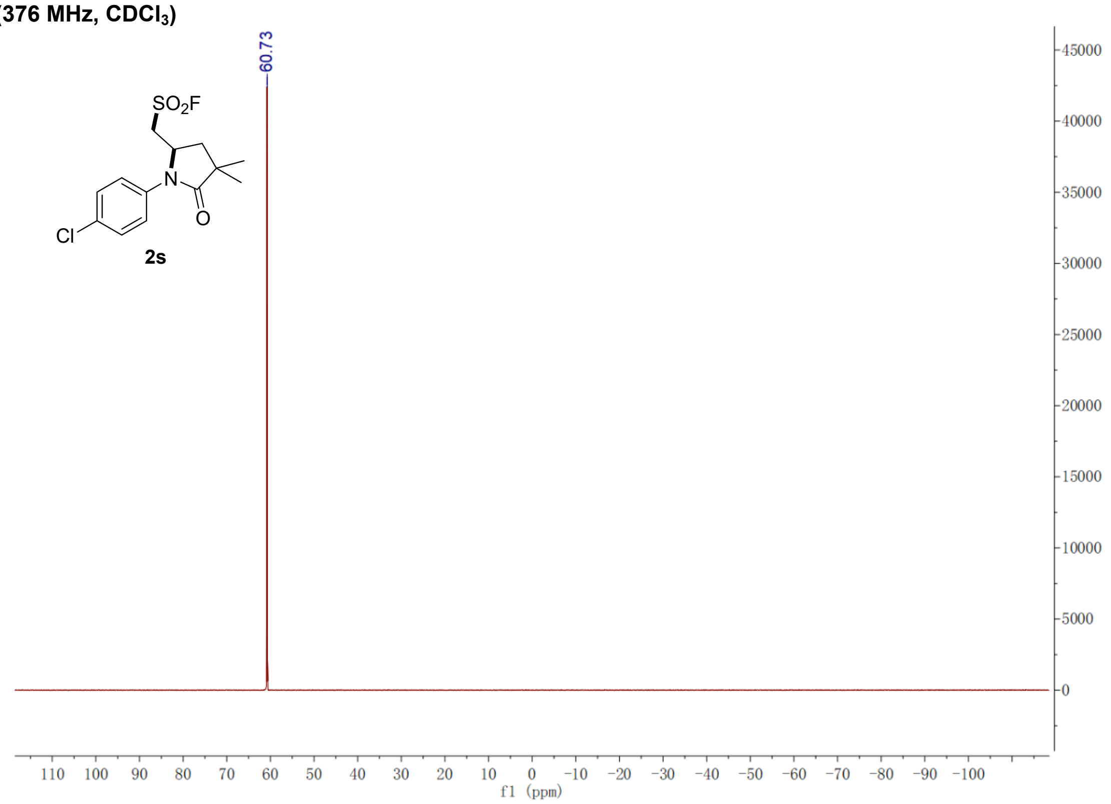

^{1}H-NMR (400 MHz, $CDCl_3$)

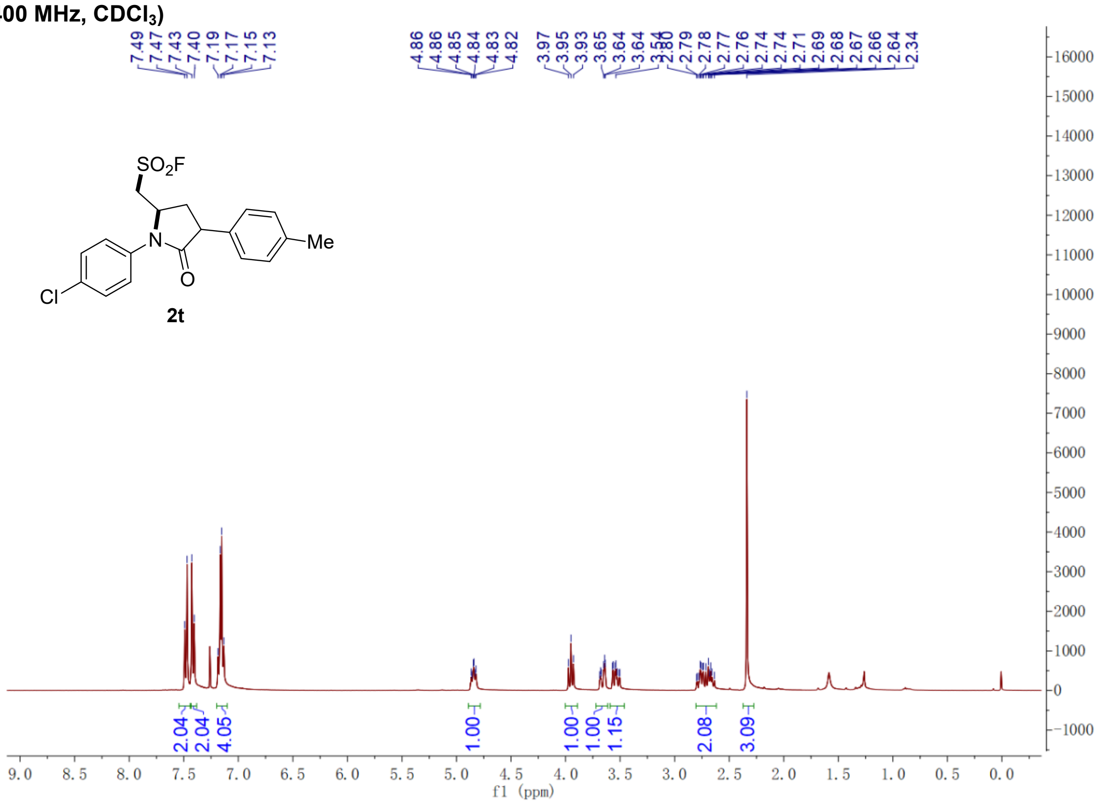

^{13}C-NMR (101 MHz, $CDCl_3$)

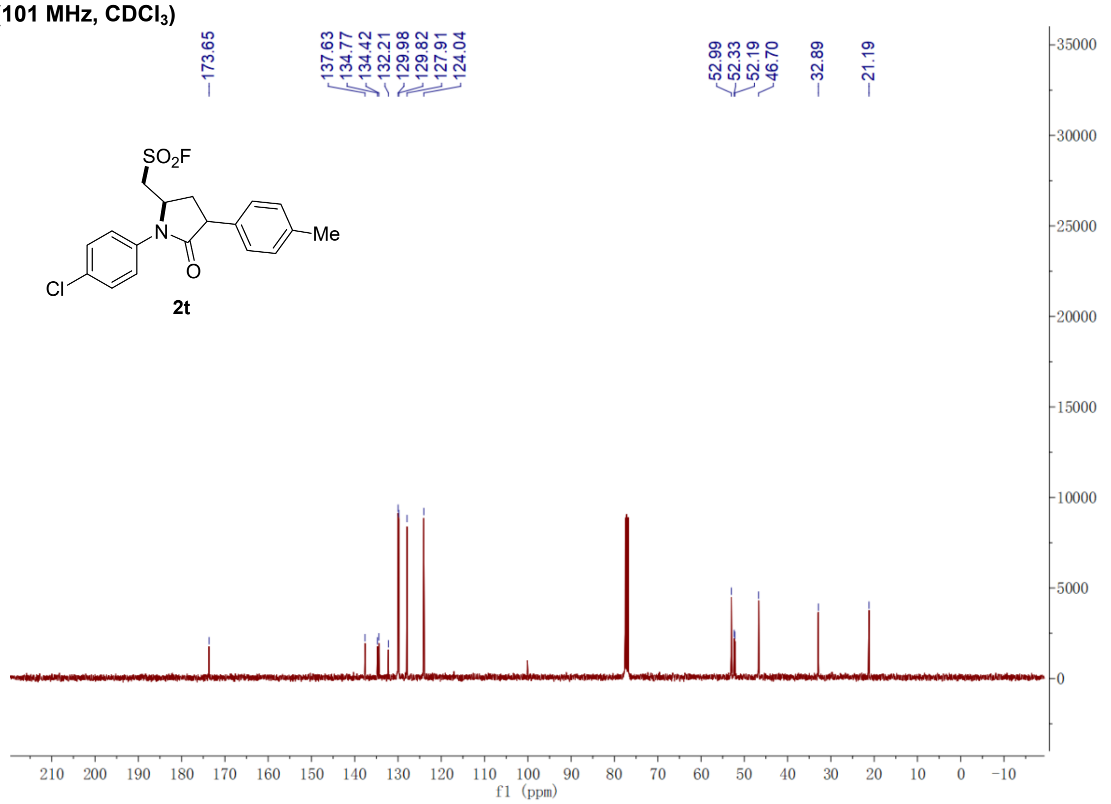

^{19}F NMR (376 MHz, $CDCl_3$)

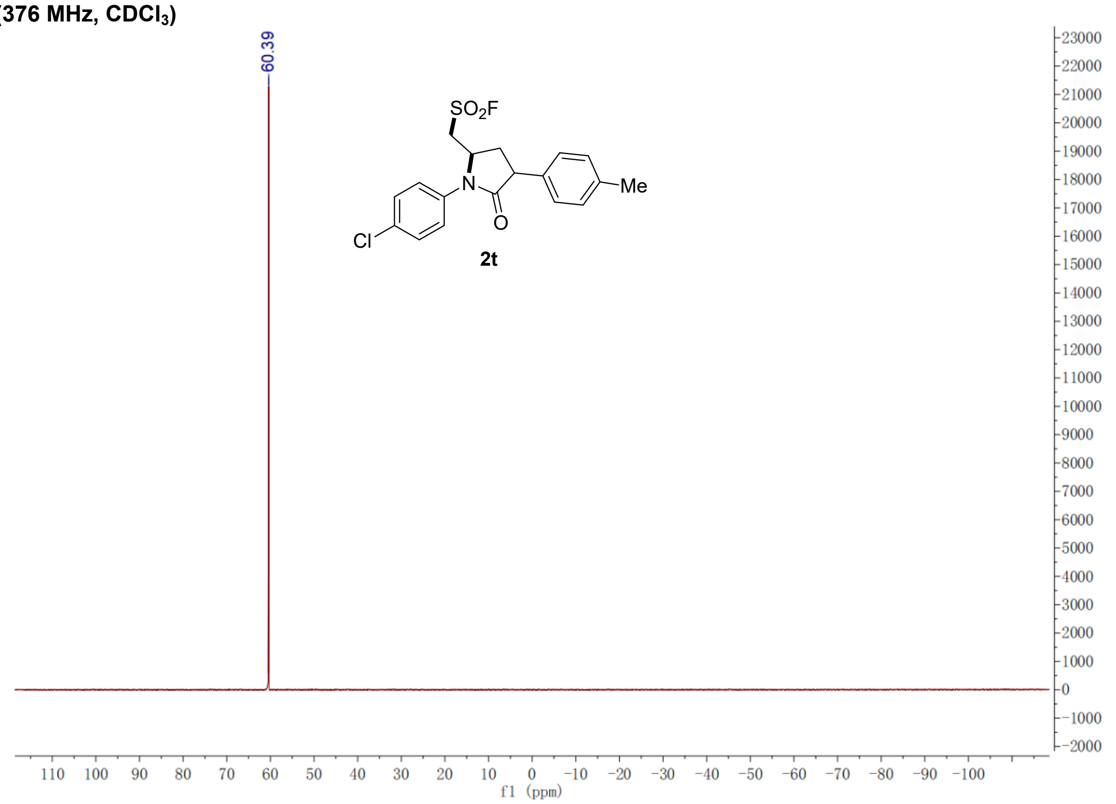

^{1}H-NMR (400 MHz, $CDCl_3$)

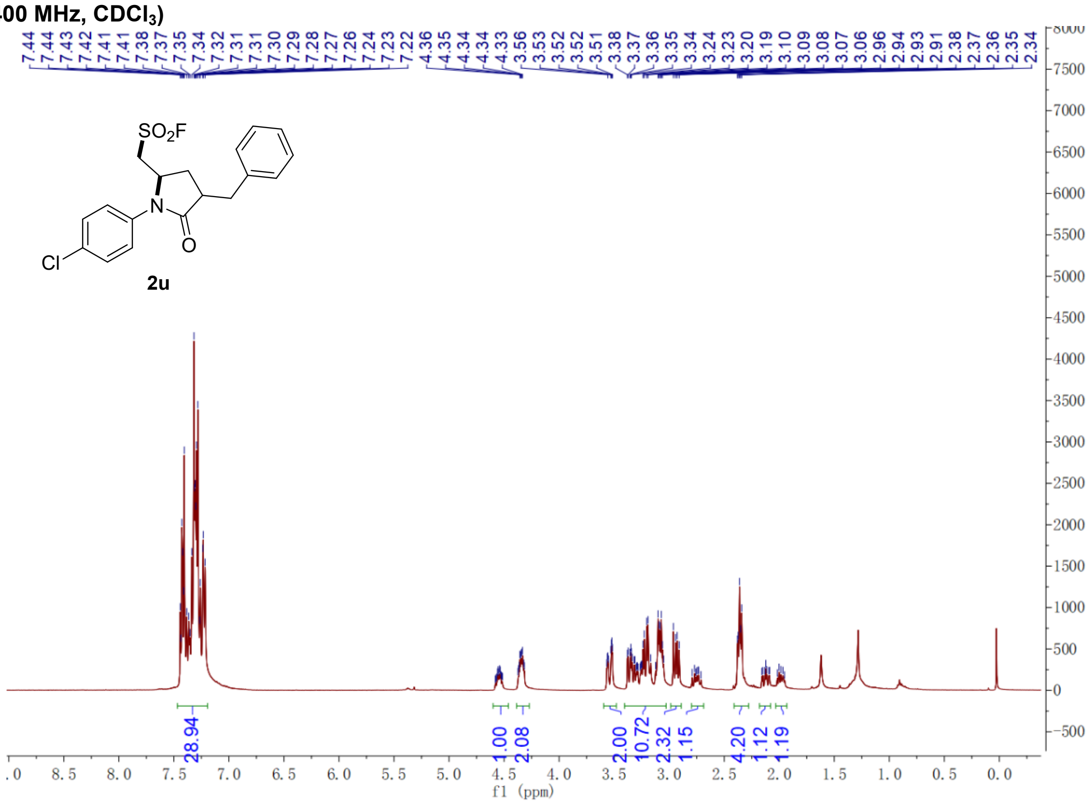

^{13}C-NMR (126 MHz, $CDCl_3$)

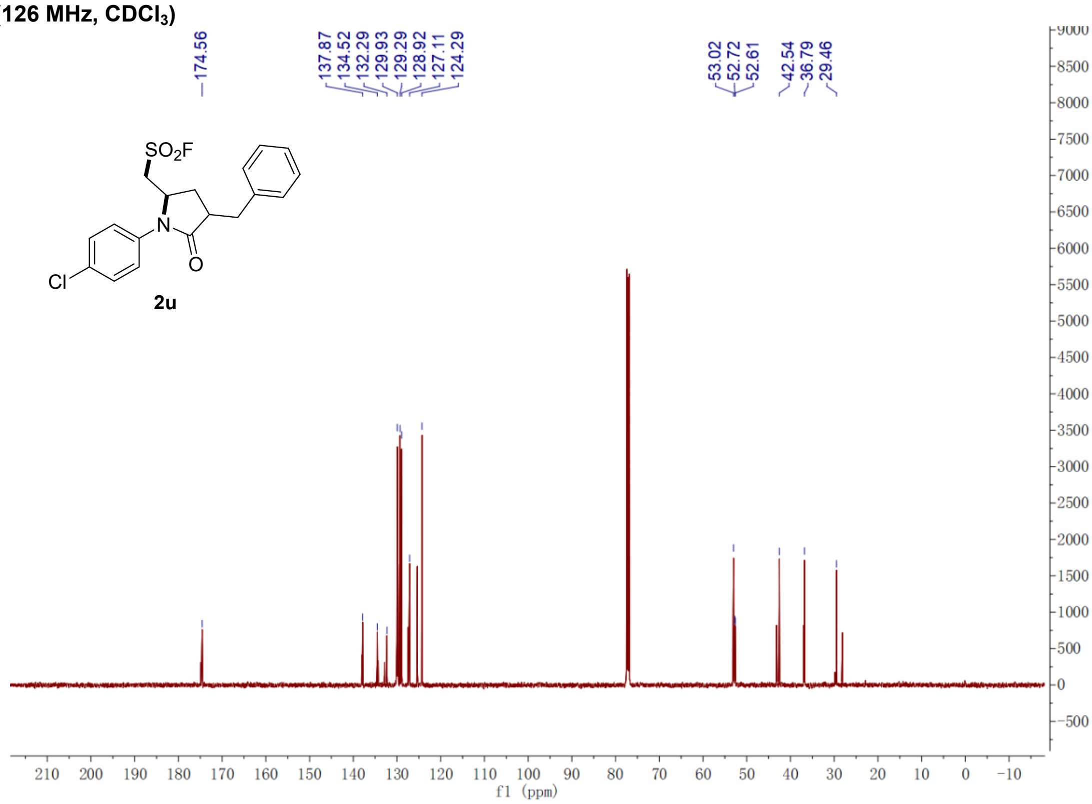

^{19}F NMR (376 MHz, $CDCl_3$)

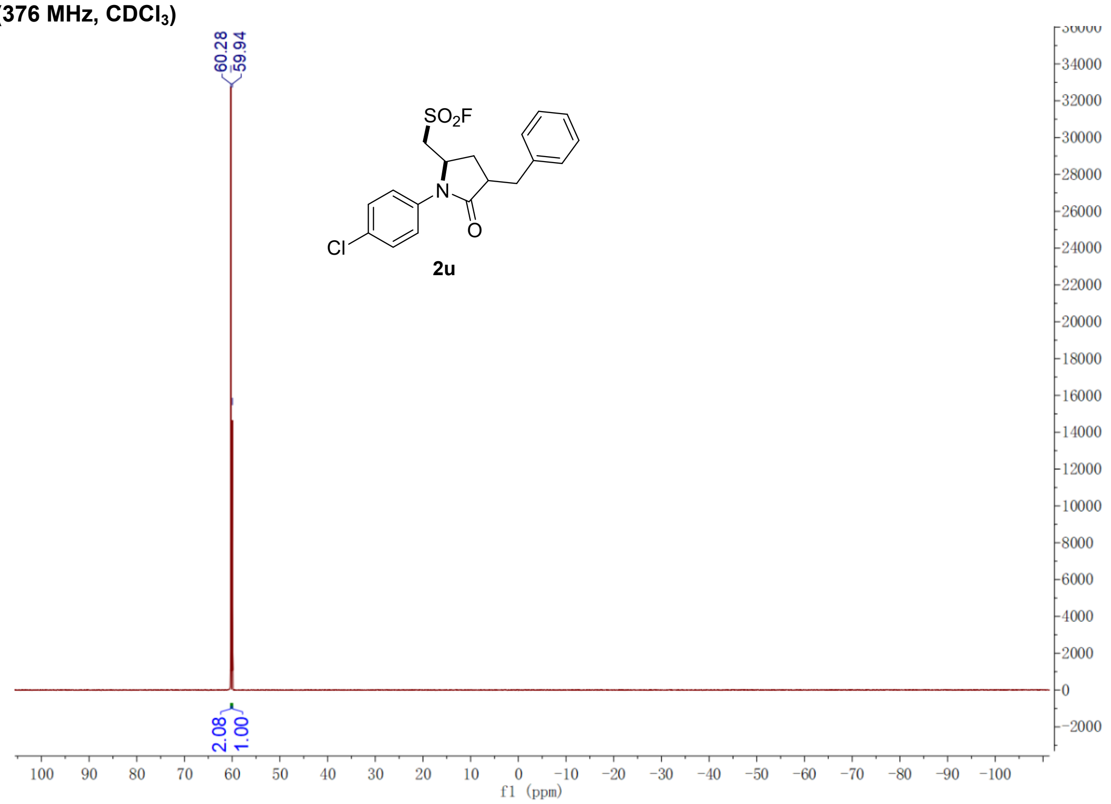

^{1}H-NMR (400 MHz, $CDCl_3$)

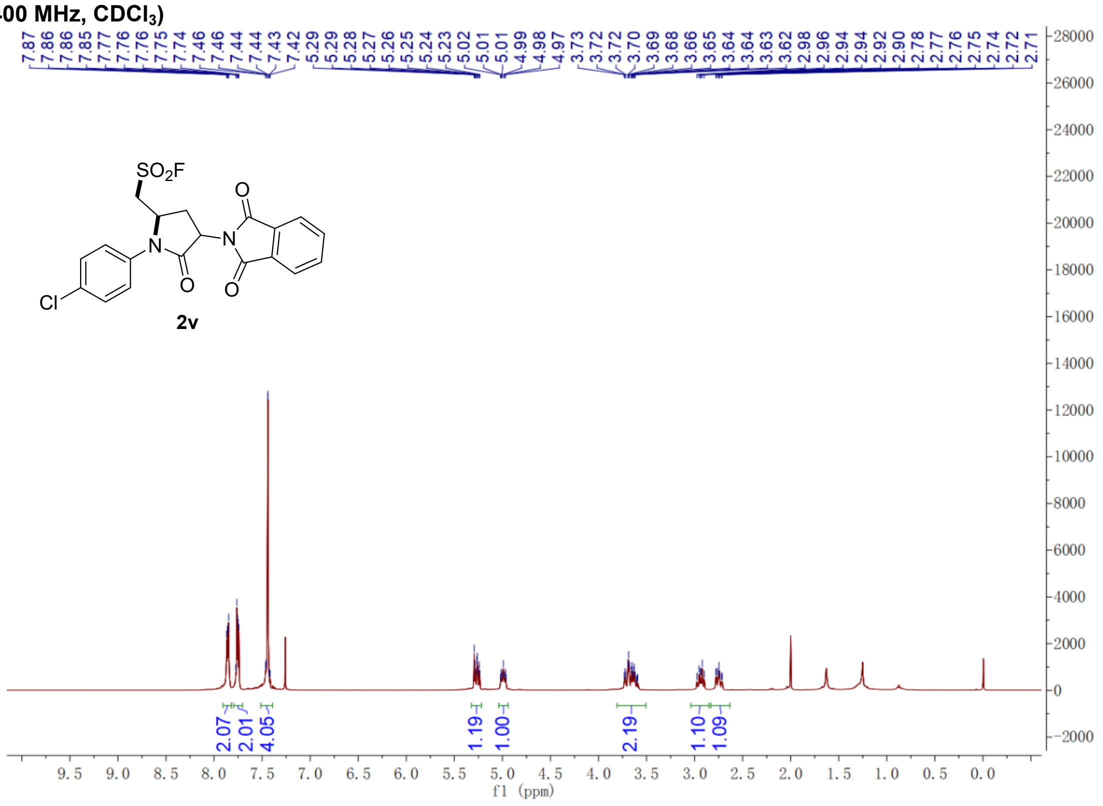

^{13}C-NMR (101 MHz, $CDCl_3$)

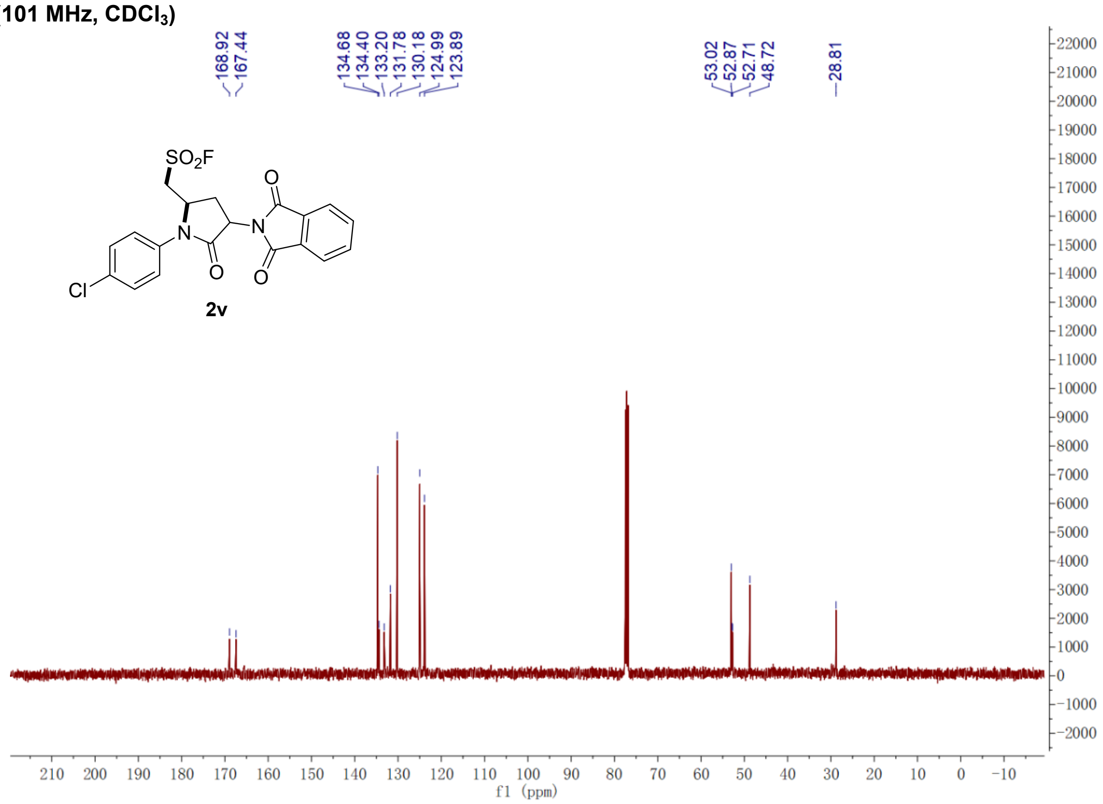

^{19}F NMR (376 MHz, $CDCl_3$)

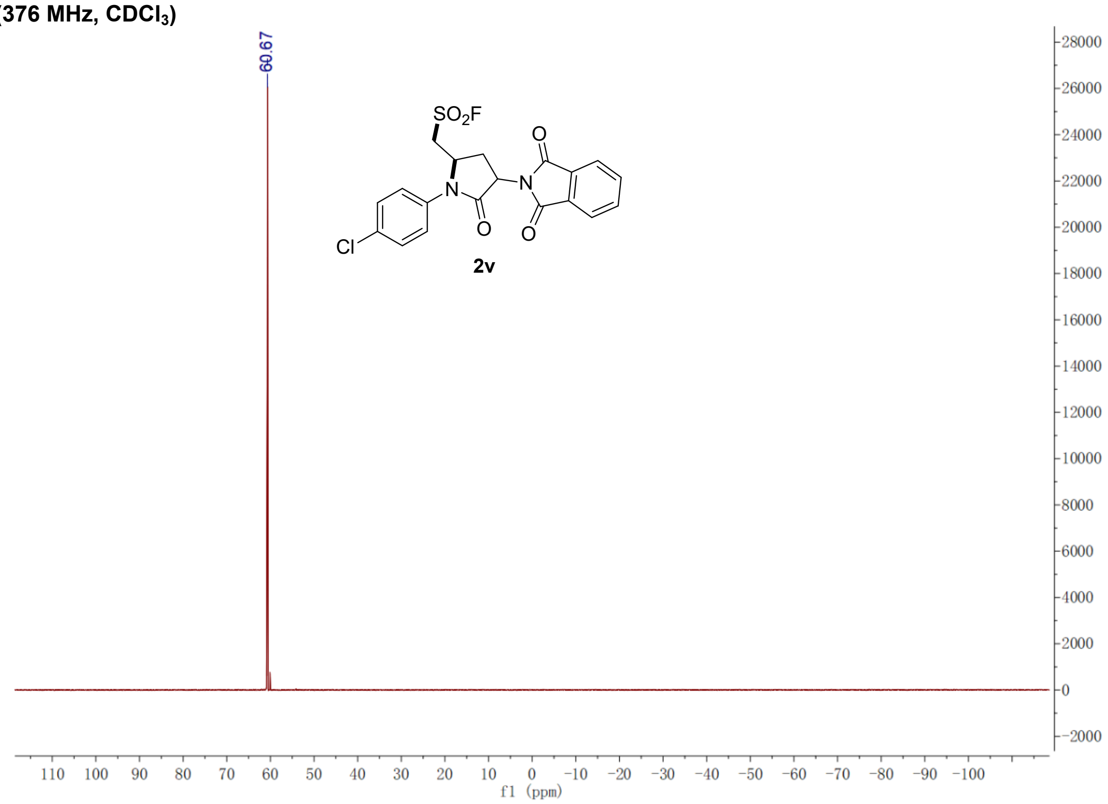

^{1}H-NMR (400 MHz, $CDCl_3$)

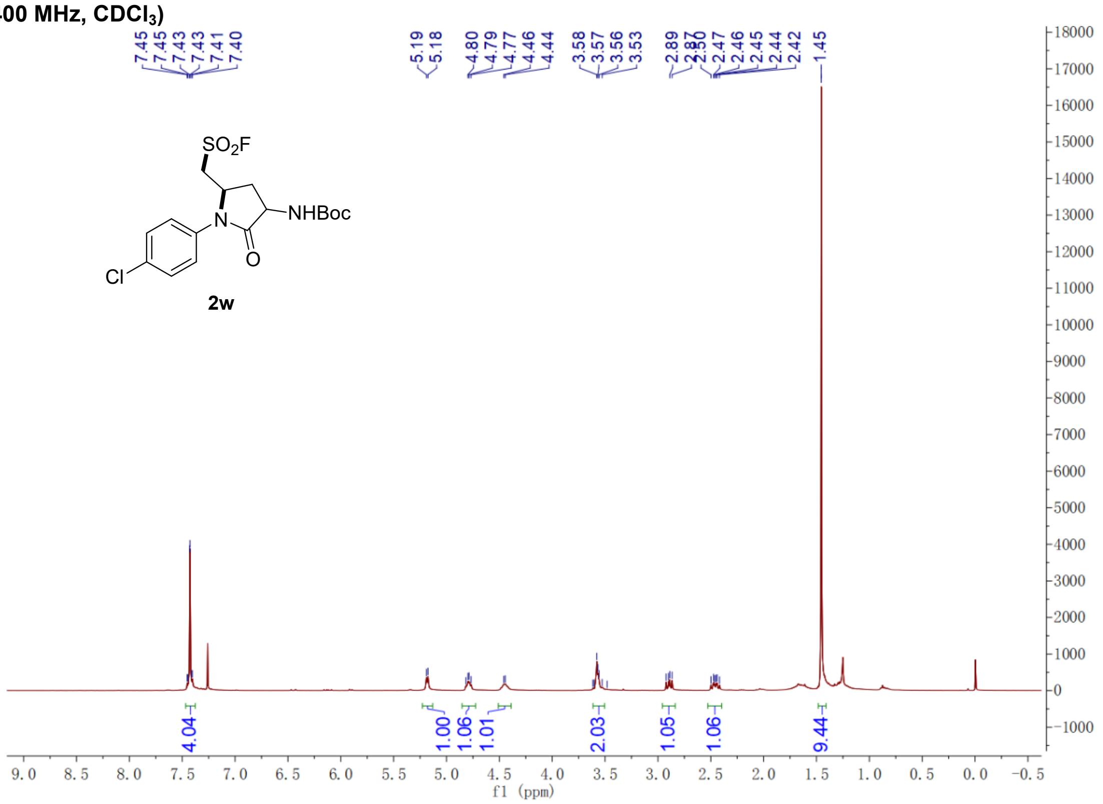

^{13}C-NMR (126 MHz, $CDCl_3$)

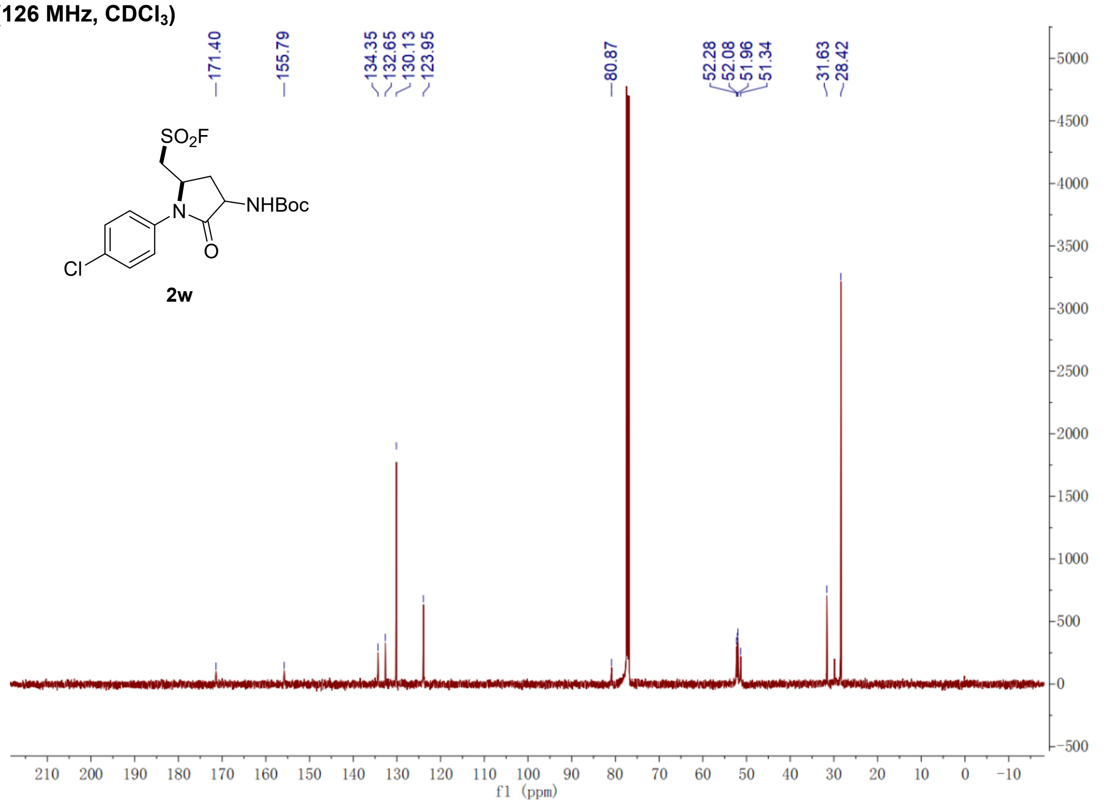

^{19}F NMR (376 MHz, $CDCl_3$)

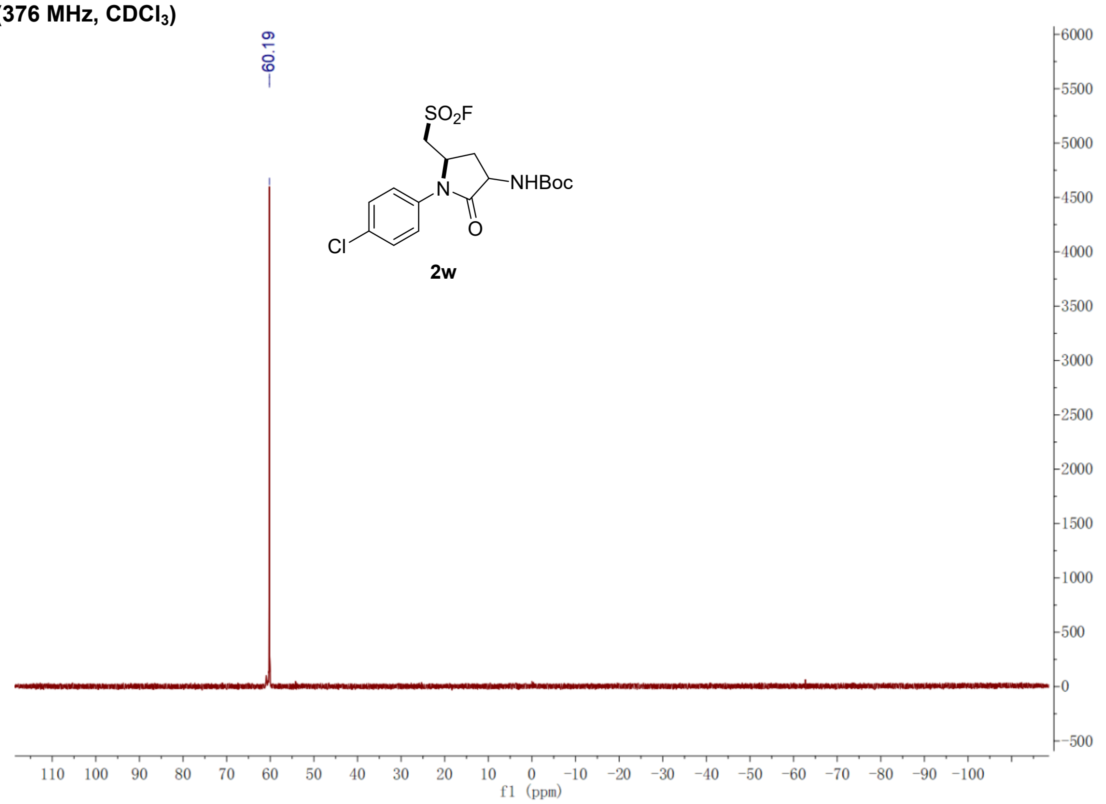

2w

^{1}H-NMR (500 MHz, DMSO-d_6)

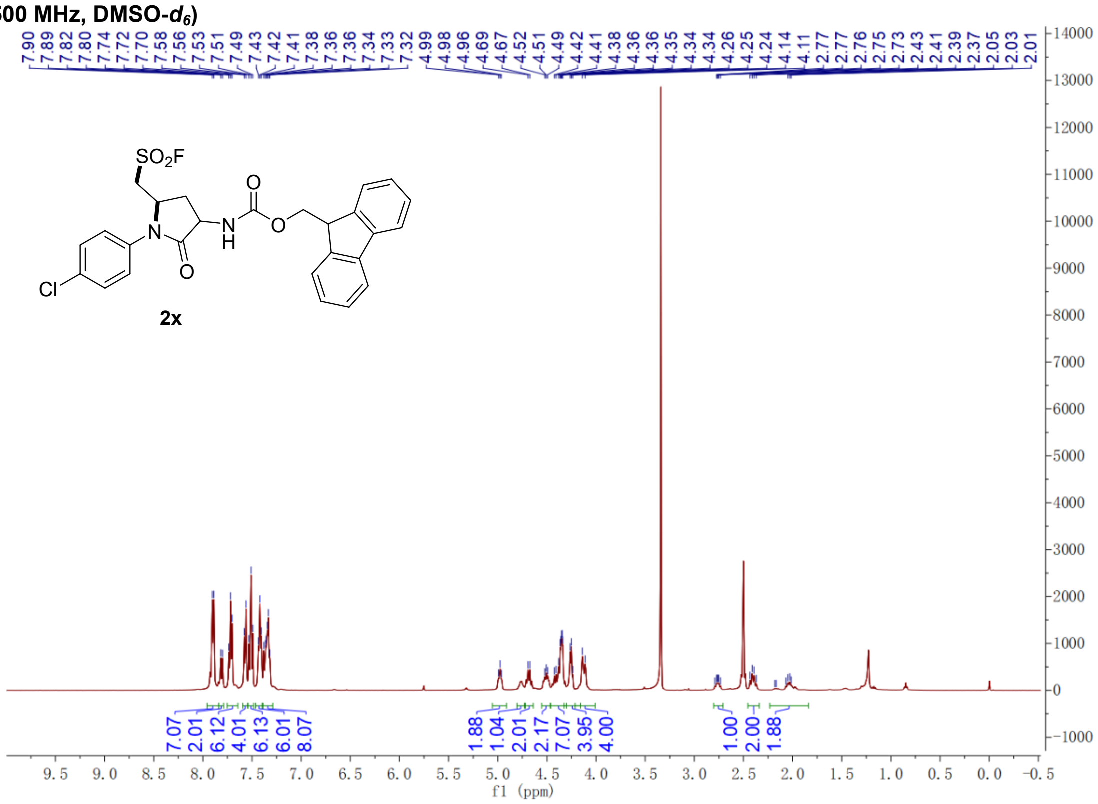

2x

^{13}C-NMR (126 MHz, DMSO-d_6)

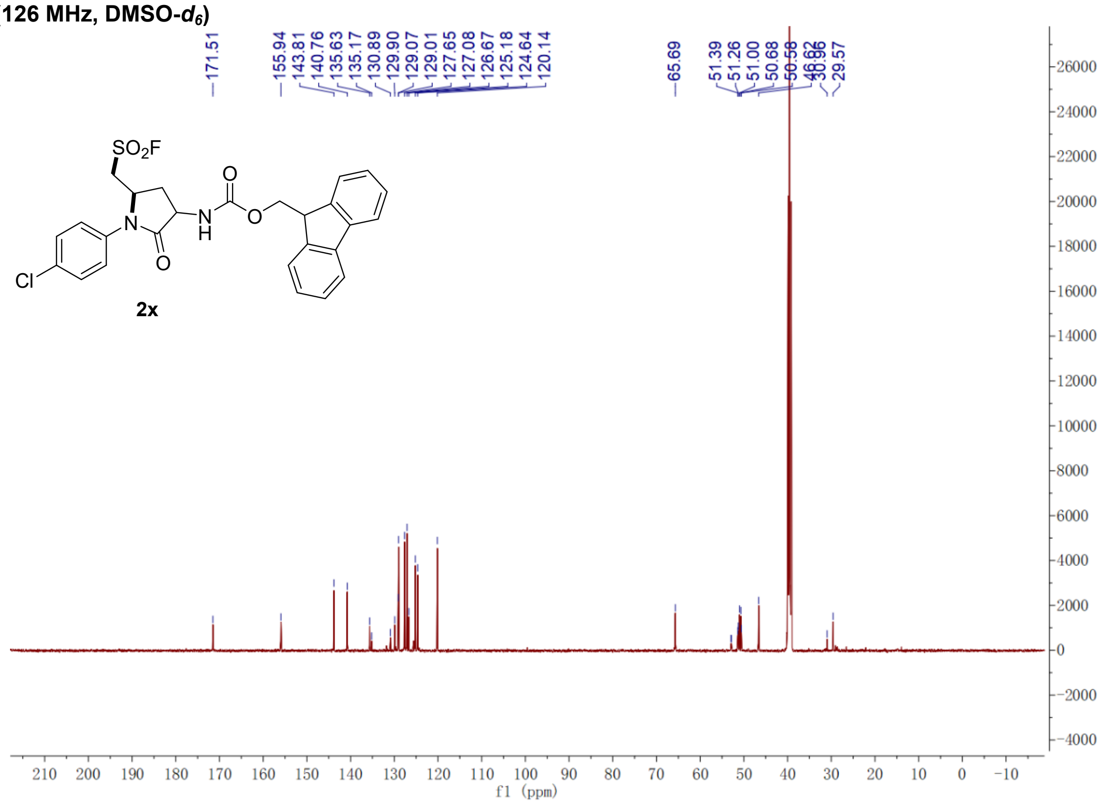

2x

^{19}F NMR (376 MHz, DMSO-d_6)

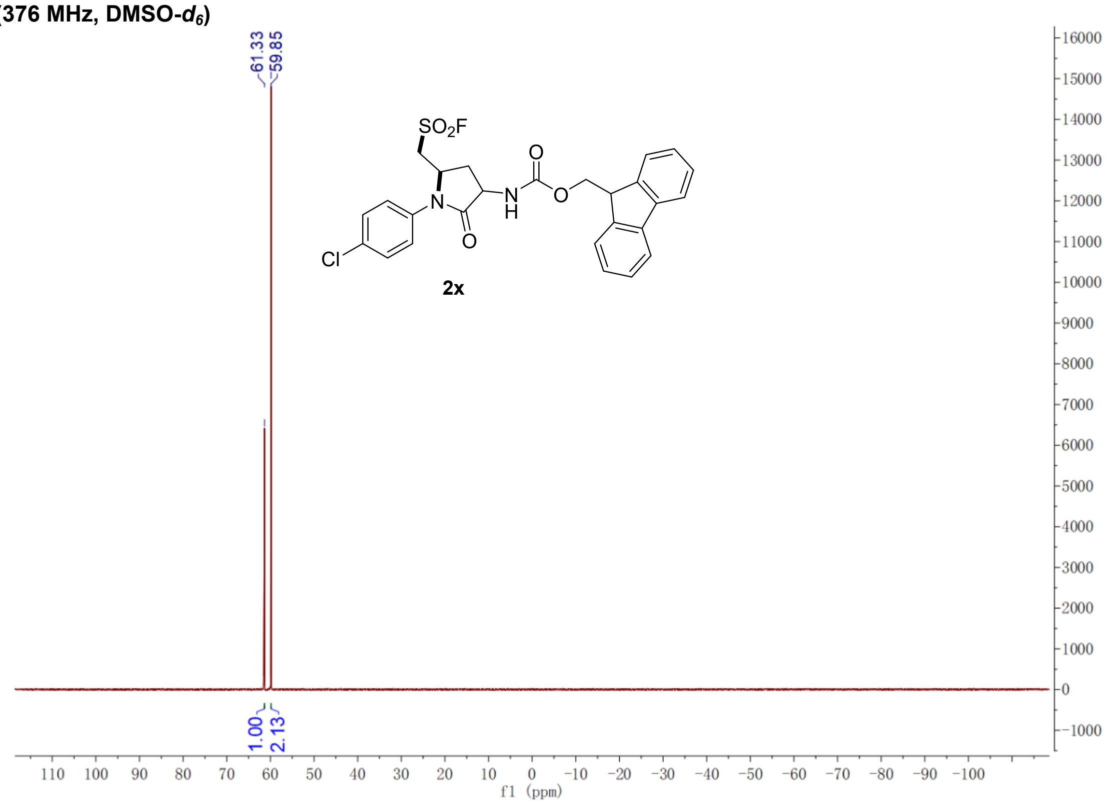

2x

^{1}H-NMR (400 MHz, $CDCl_3$)

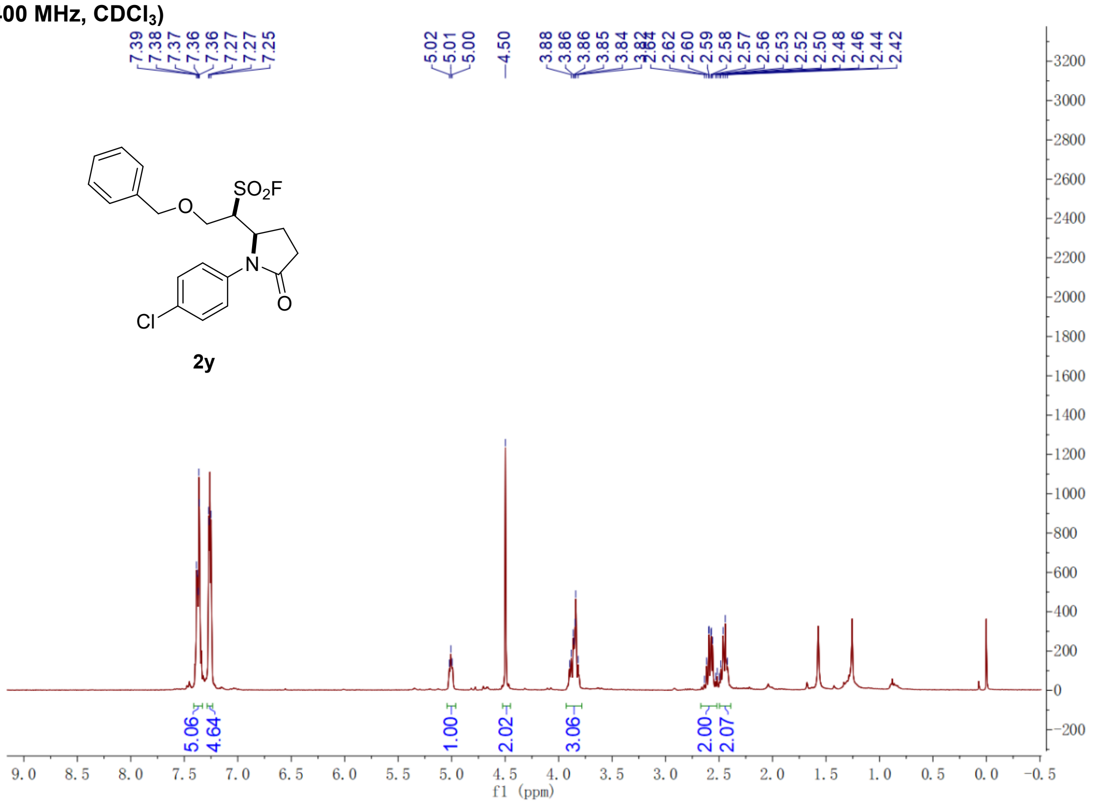

^{13}C-NMR (101 MHz, $CDCl_3$)

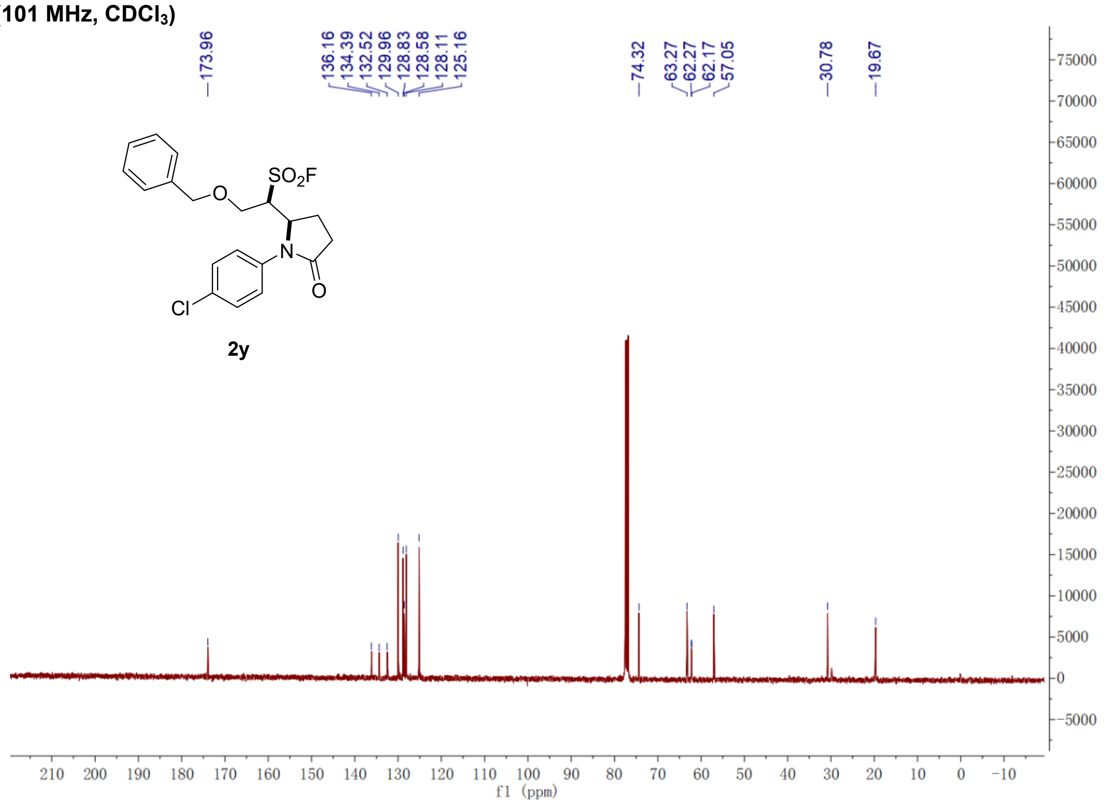

^{19}F NMR (376 MHz, $CDCl_3$)

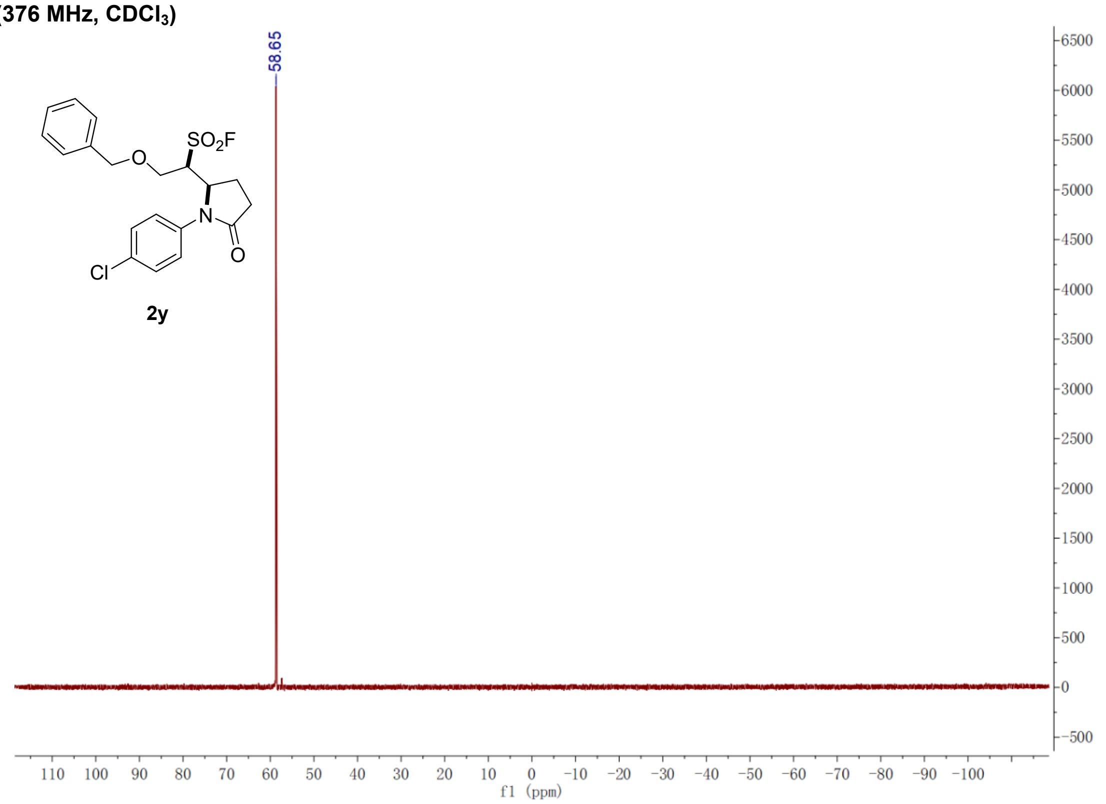

^{1}H-NMR (400 MHz, $CDCl_3$)

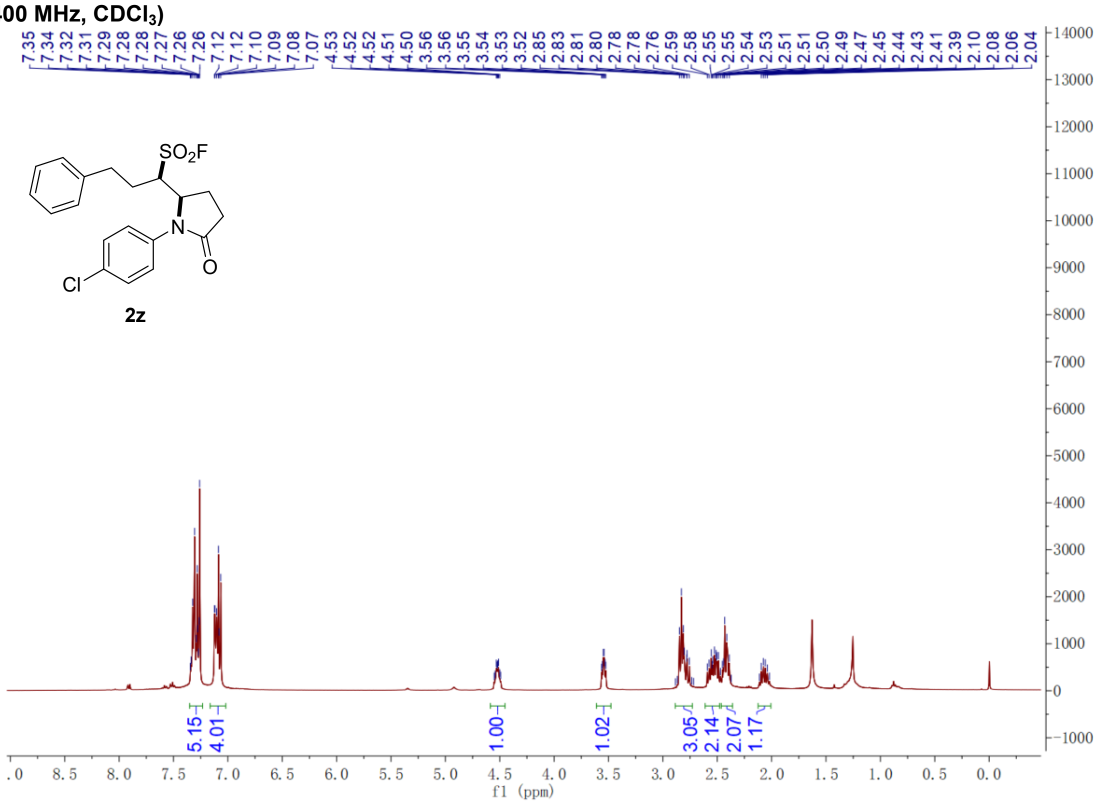

^{13}C-NMR (101 MHz, $CDCl_3$)

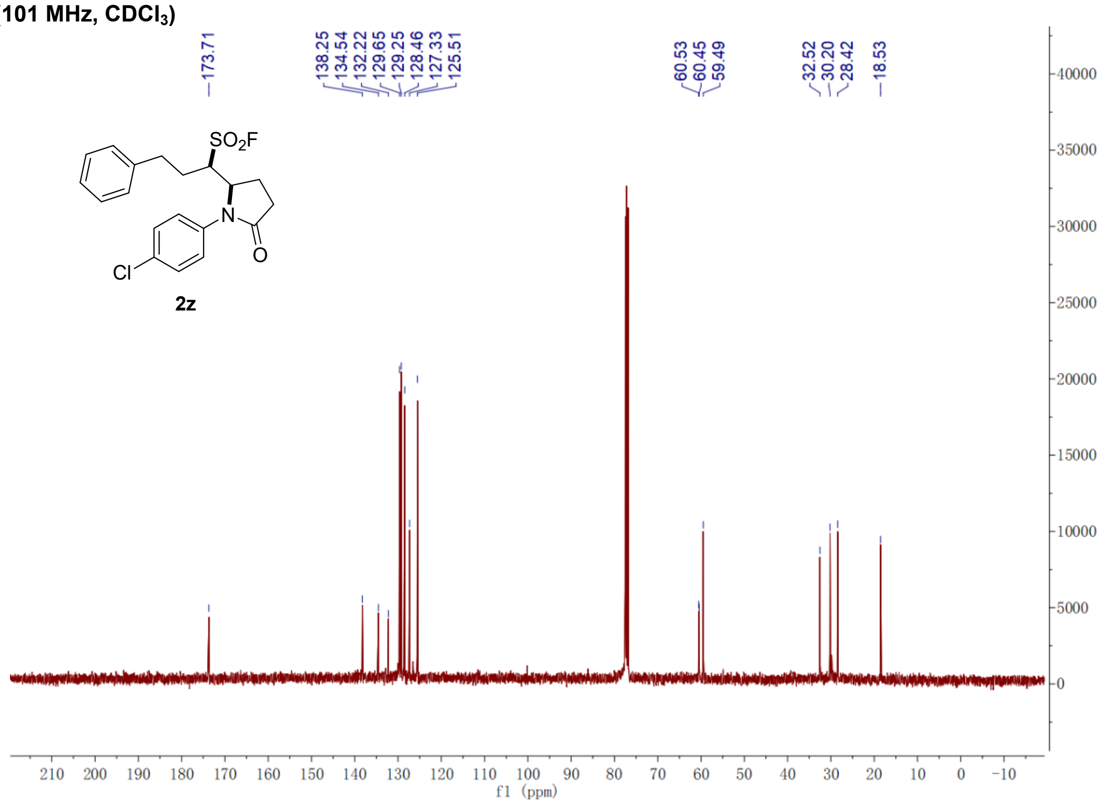

^{19}F NMR (376 MHz, $CDCl_3$)

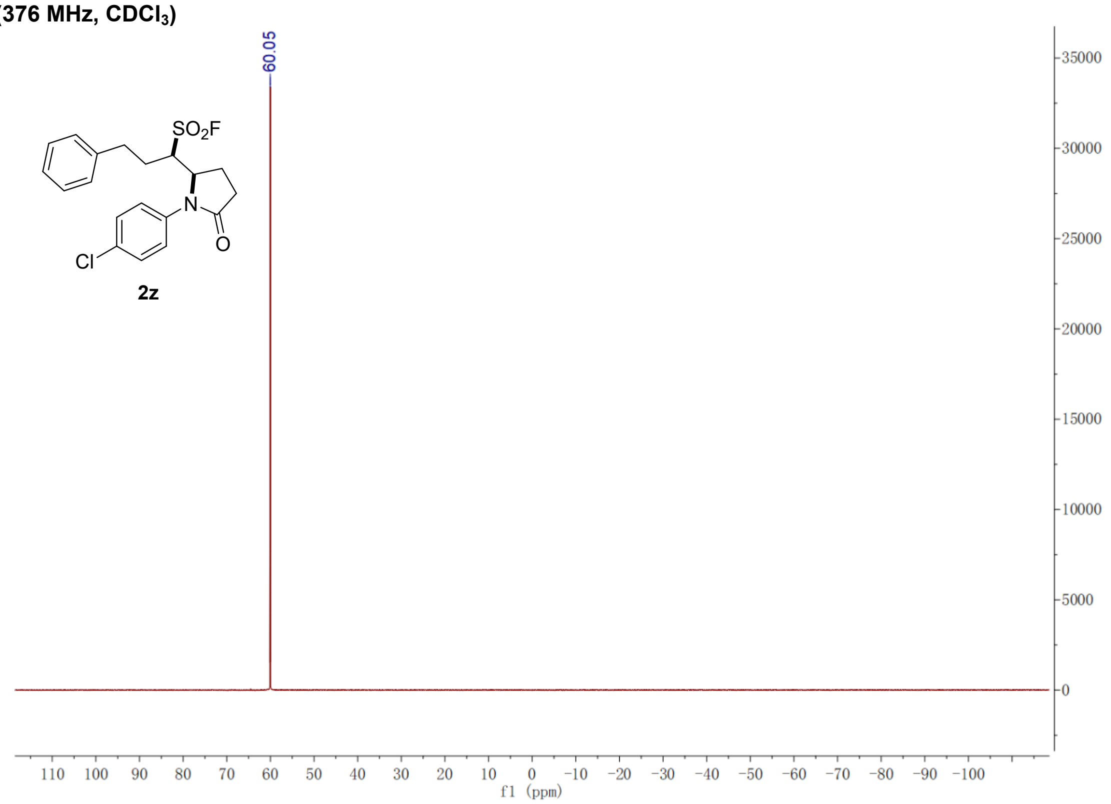

^{1}H-NMR (400 MHz, $CDCl_3$)

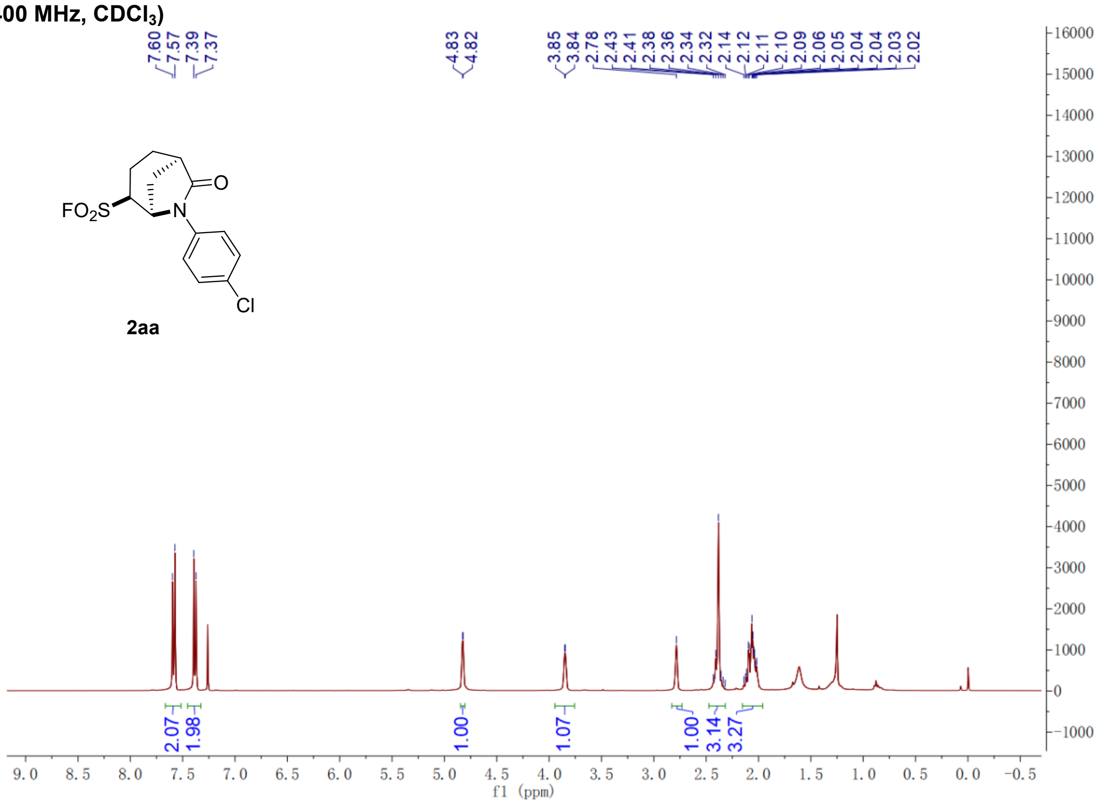

^{13}C-NMR (101 MHz, $CDCl_3$)

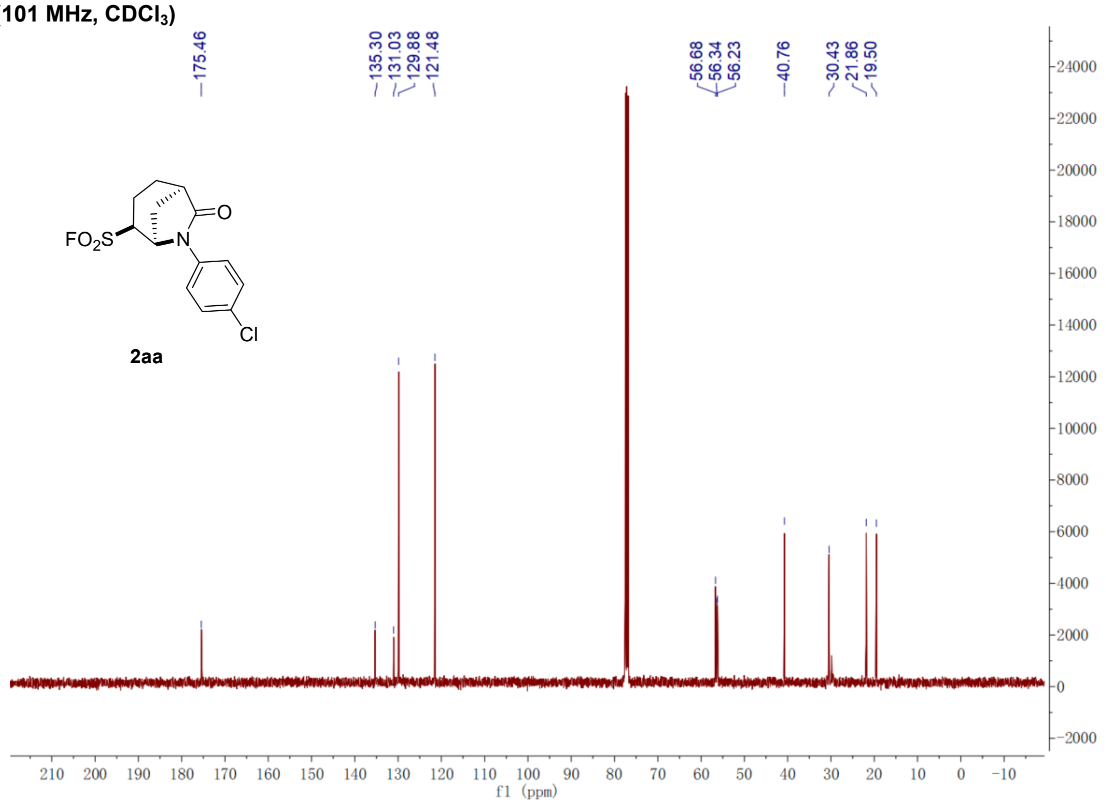

^{19}F NMR (376 MHz, $CDCl_3$)

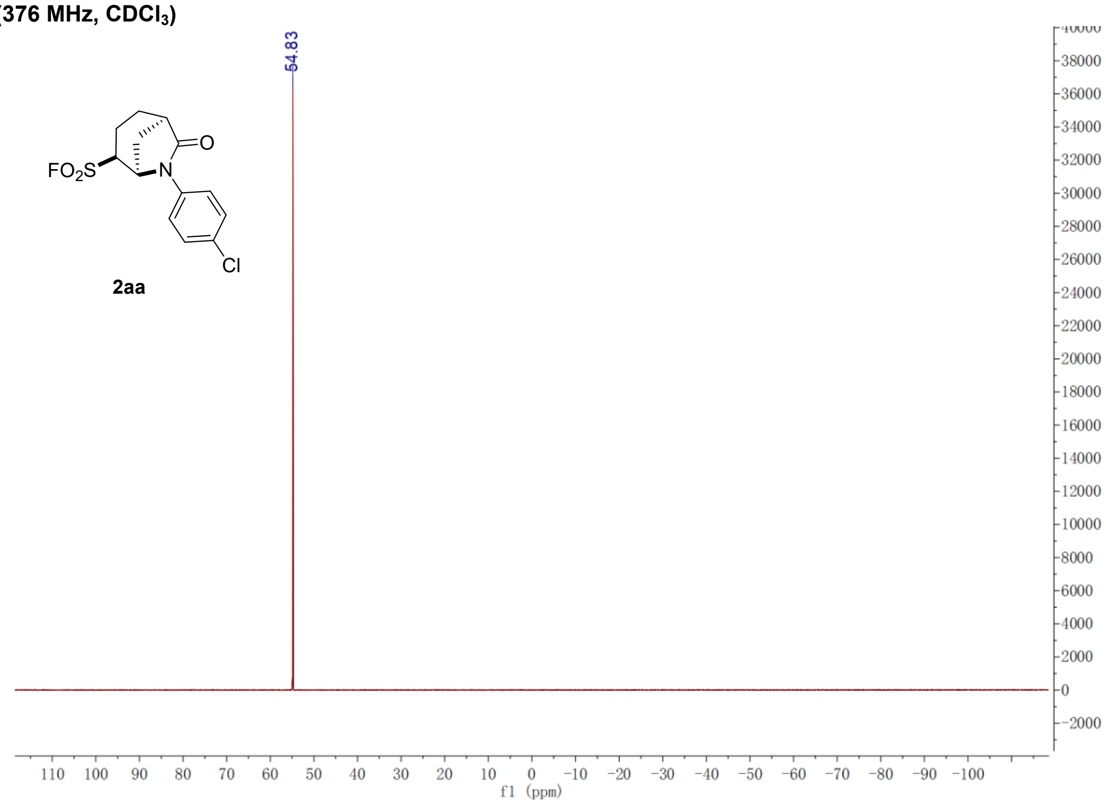

2aa

^{1}H-NMR (400 MHz, $CDCl_3$)

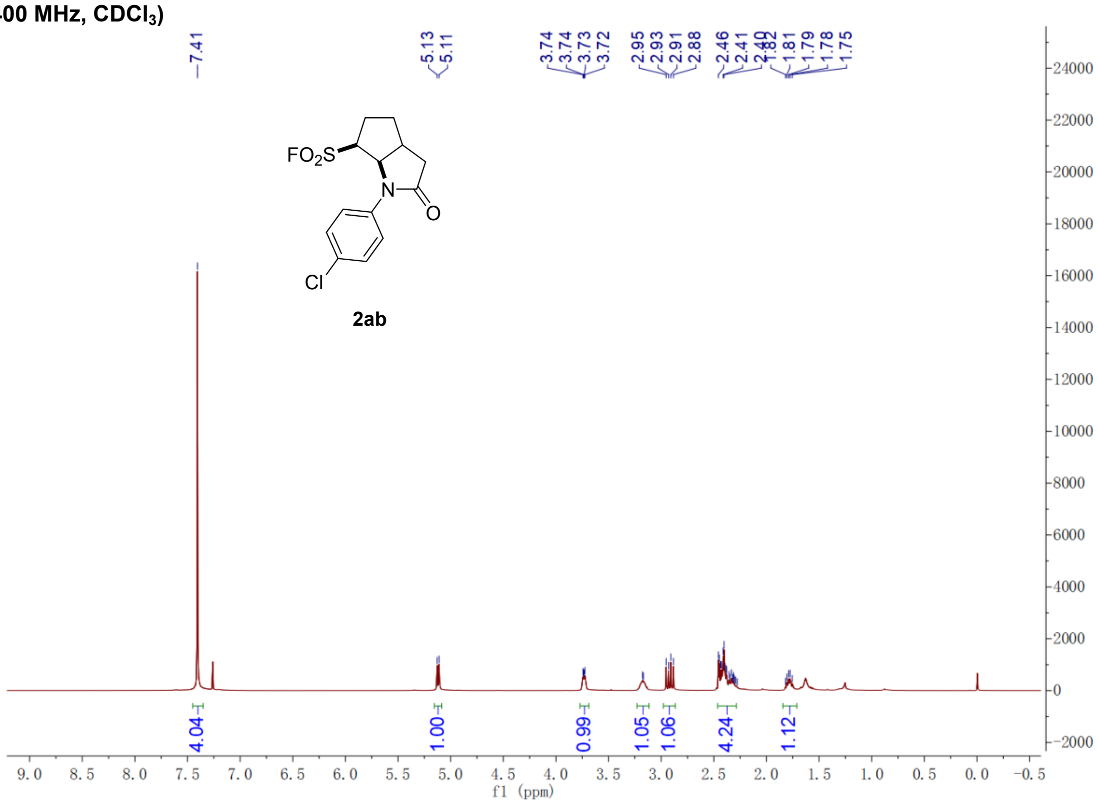

2ab

^{13}C-NMR (101 MHz, $CDCl_3$)

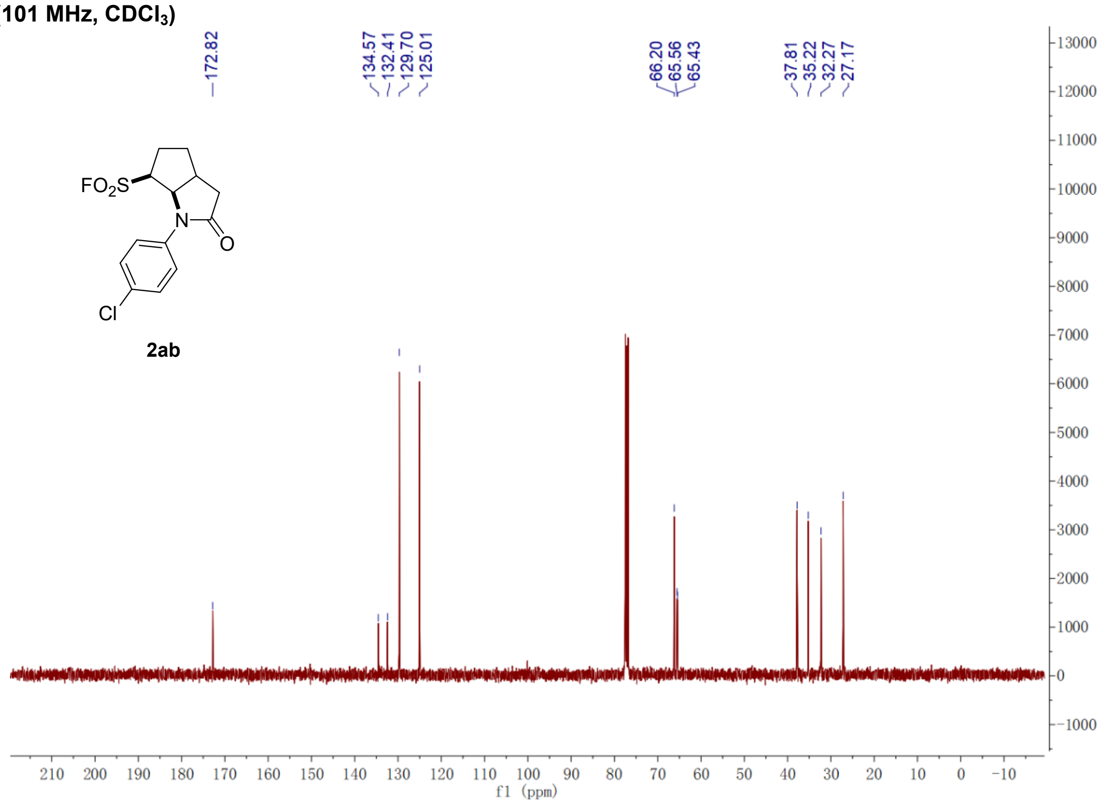

^{19}F NMR (376 MHz, $CDCl_3$)

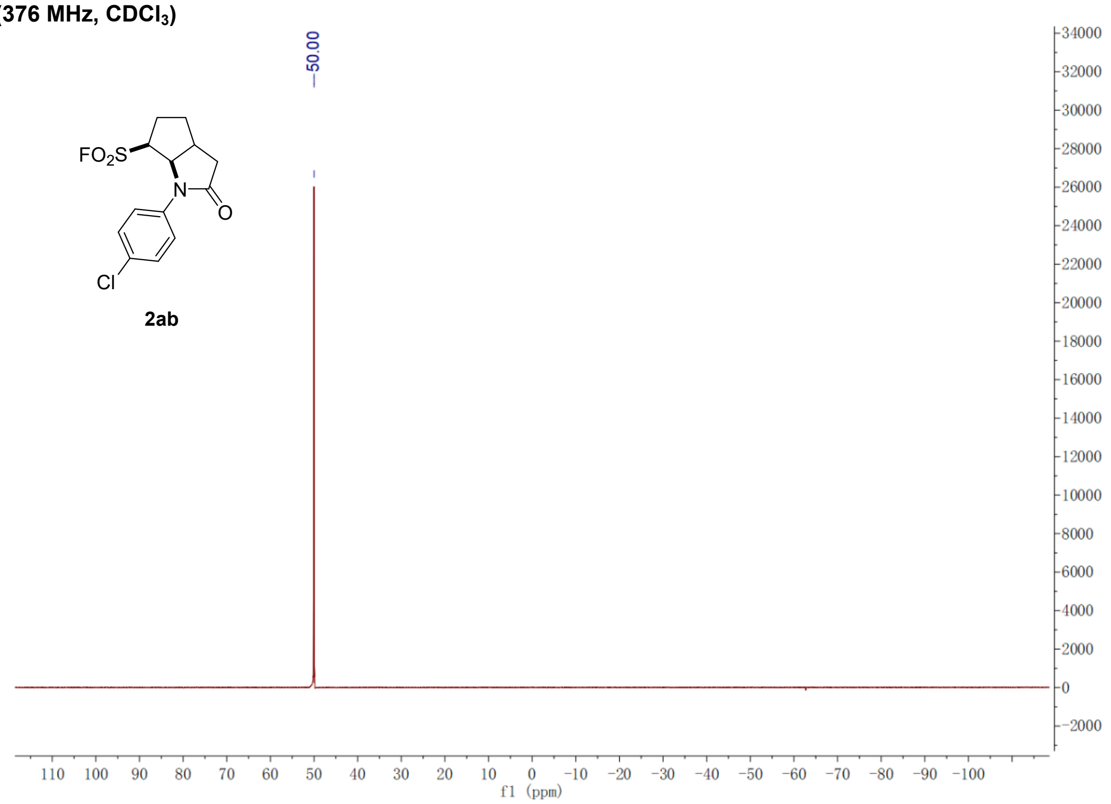

^{1}H-NMR (400 MHz, $CDCl_3$)

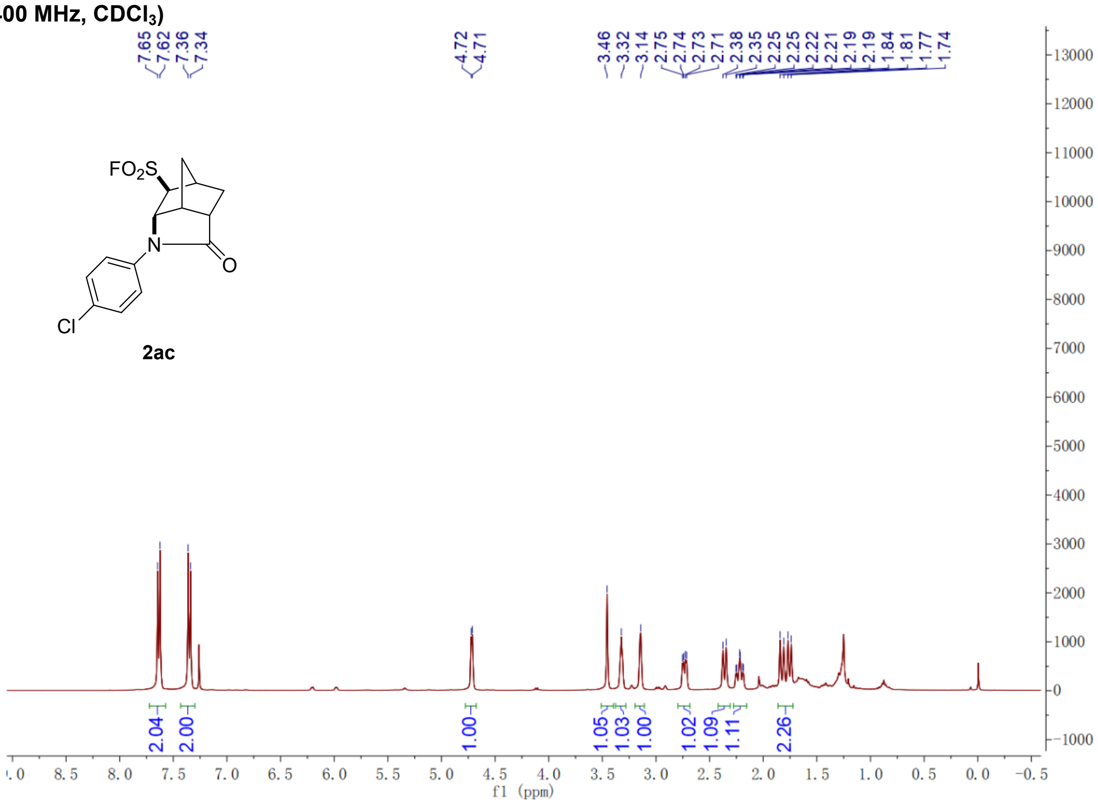

^{13}C-NMR (126 MHz, $CDCl_3$)

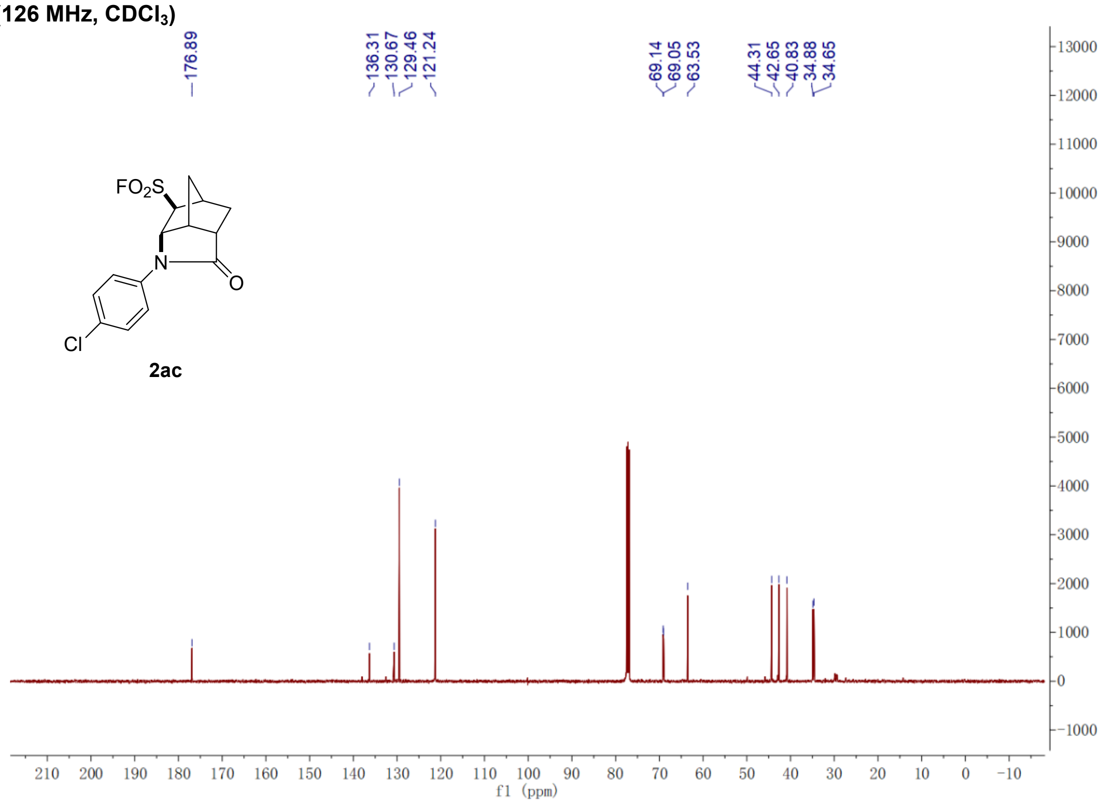

^{19}F NMR (376 MHz, $CDCl_3$)

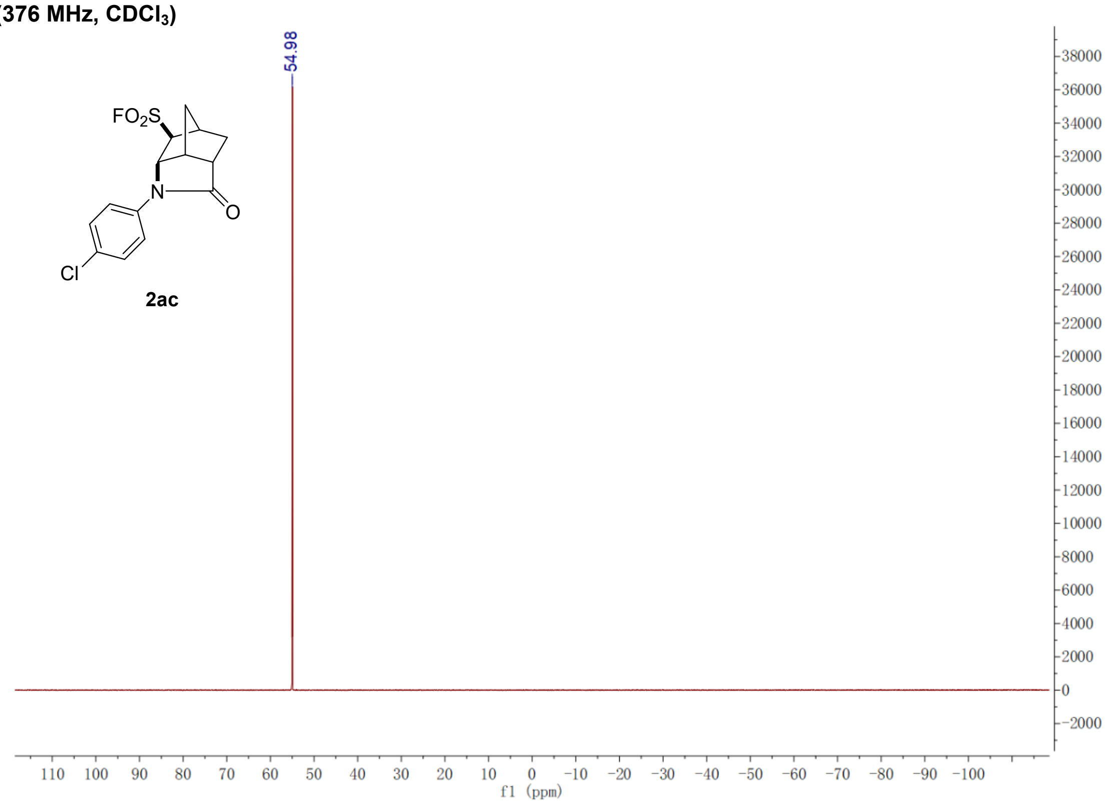

^{1}H-NMR (400 MHz, $CDCl_3$)

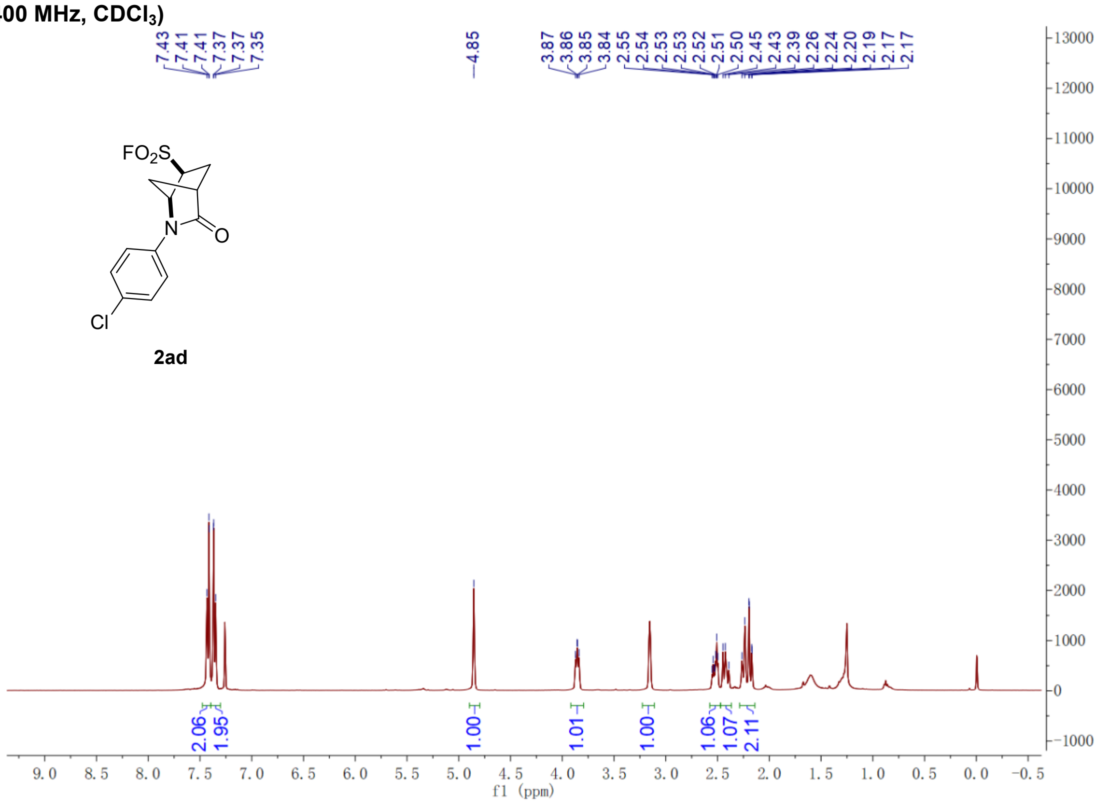

^{13}C-NMR (101 MHz, $CDCl_3$)

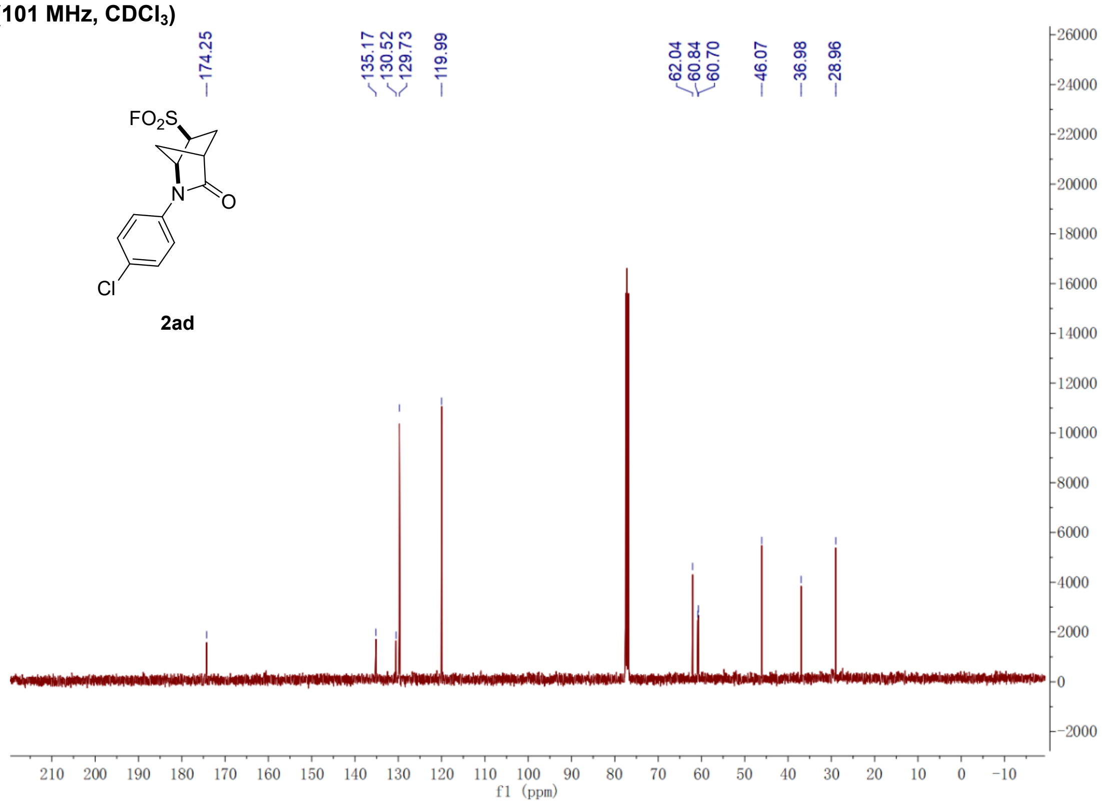

^{19}F NMR (376 MHz, $CDCl_3$)

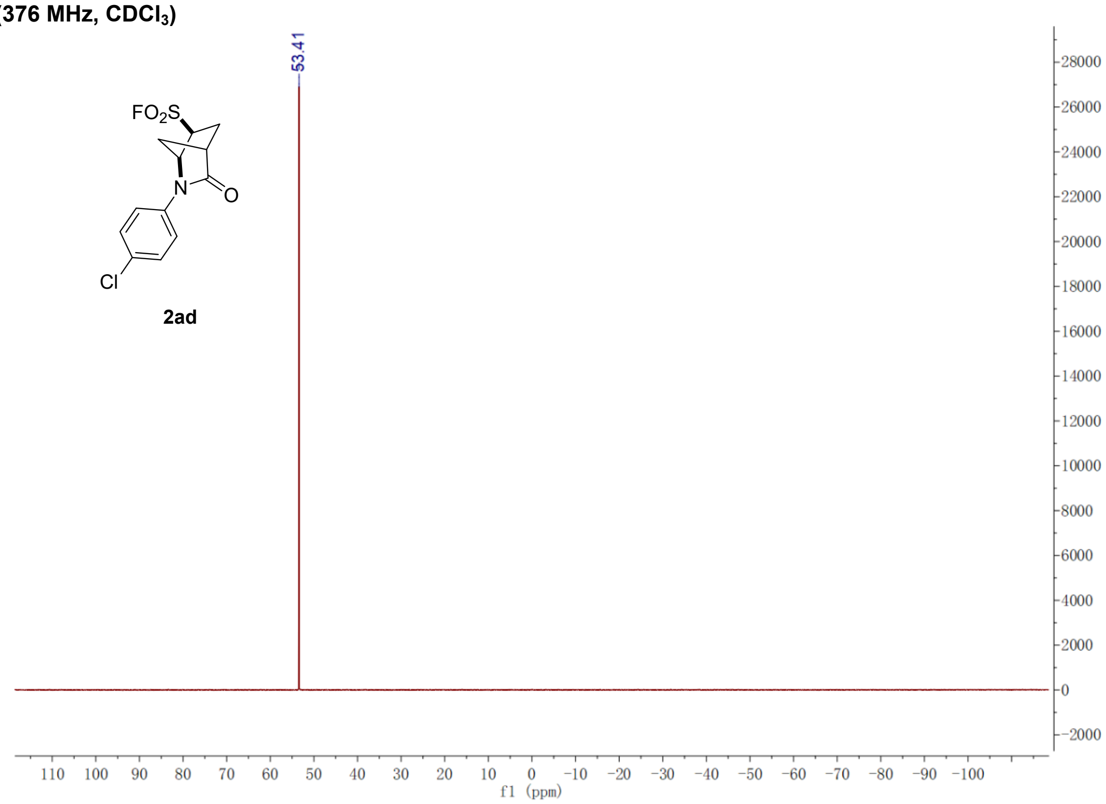

^1H-NMR (400 MHz, $CDCl_3$)

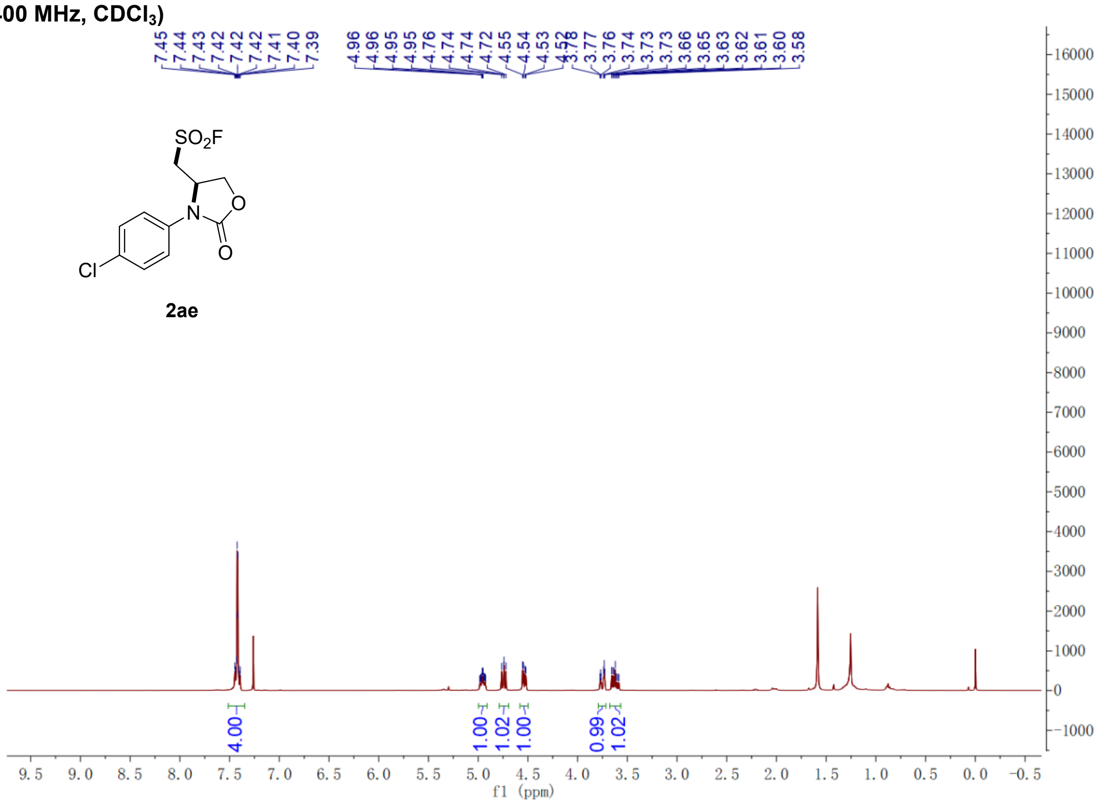

2ae

^{13}C-NMR (101 MHz, $CDCl_3$)

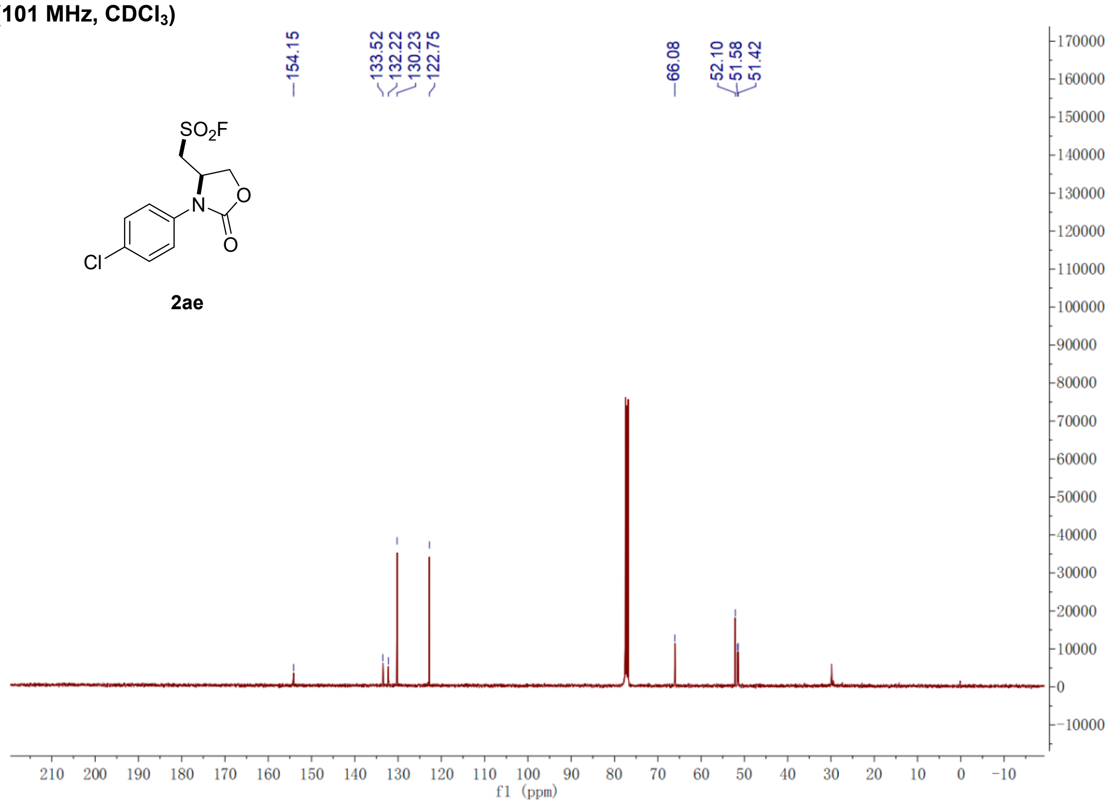

2ae

^{19}F NMR (376 MHz, $CDCl_3$)

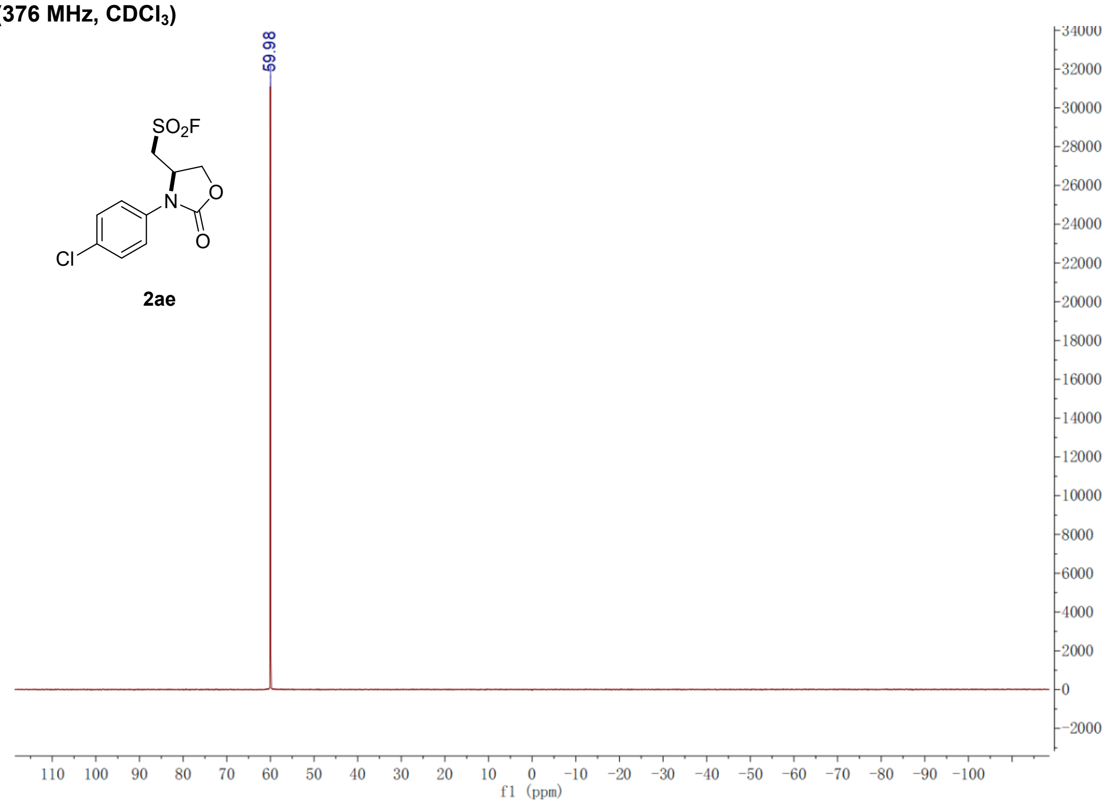

^{1}H-NMR (400 MHz, $CDCl_3$)

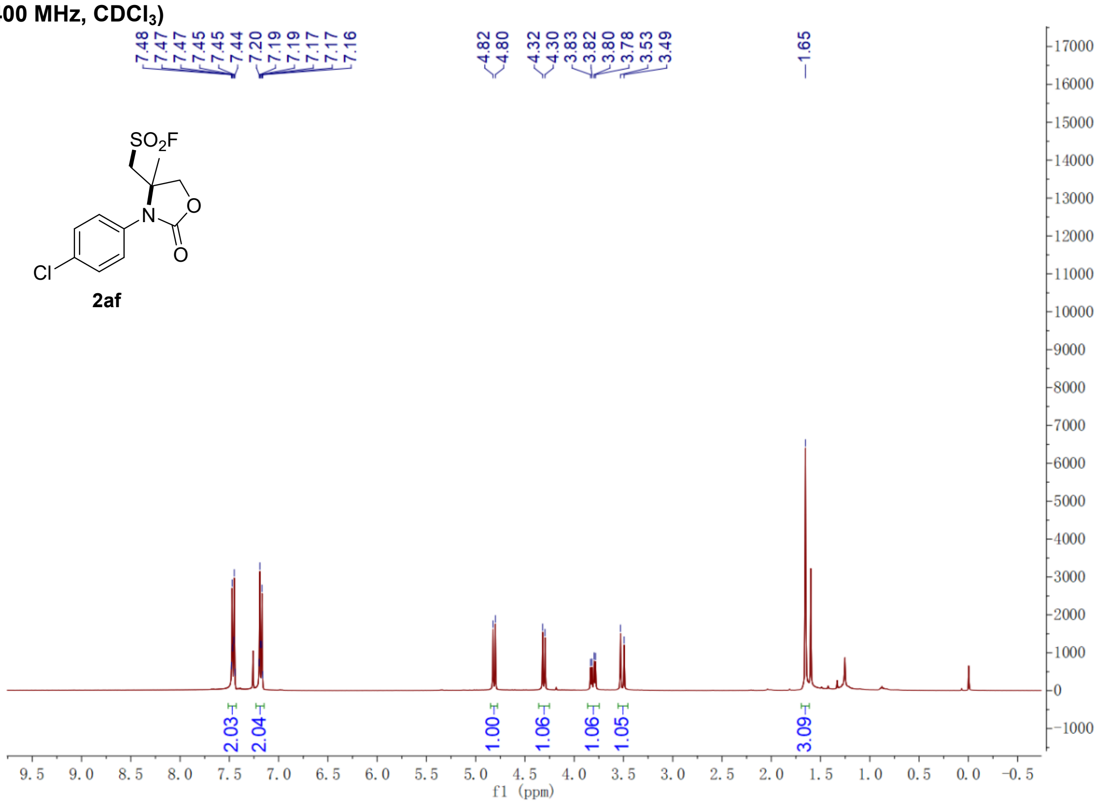

^{13}C-NMR (126 MHz, $CDCl_3$)

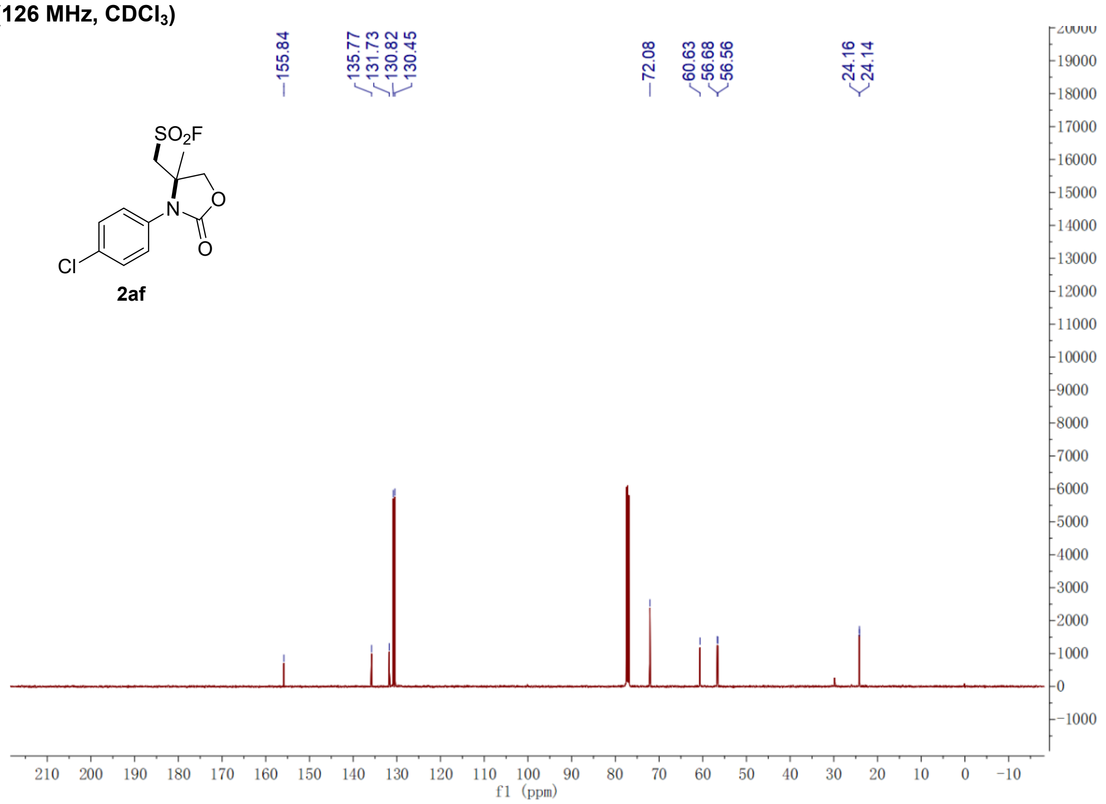

^{19}F NMR (376 MHz, $CDCl_3$)

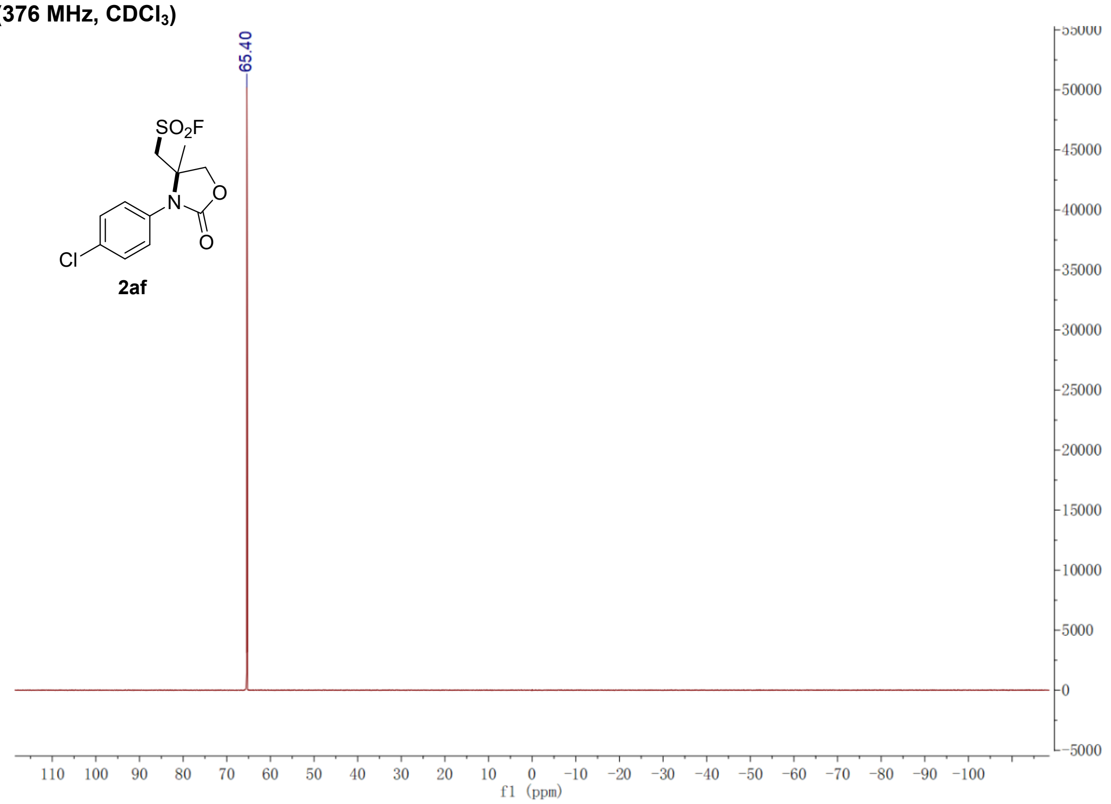

^{1}H-NMR (400 MHz, $CDCl_3$)

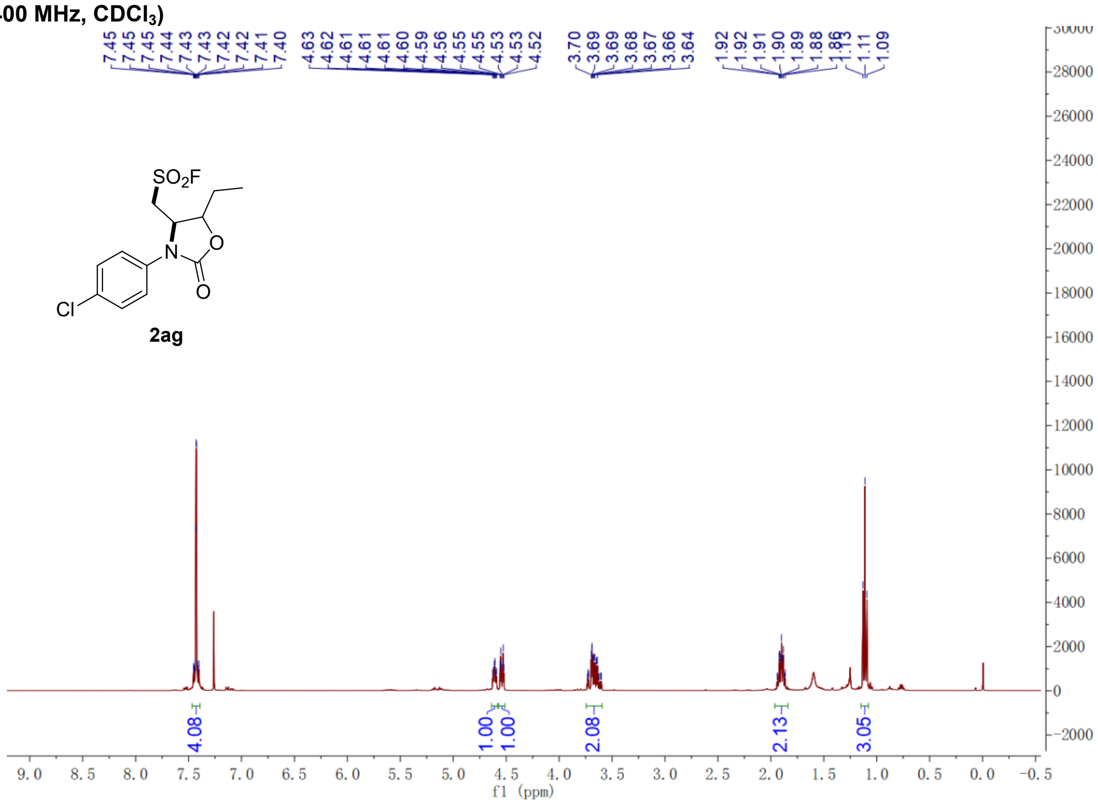

^{13}C-NMR (101 MHz, $CDCl_3$)

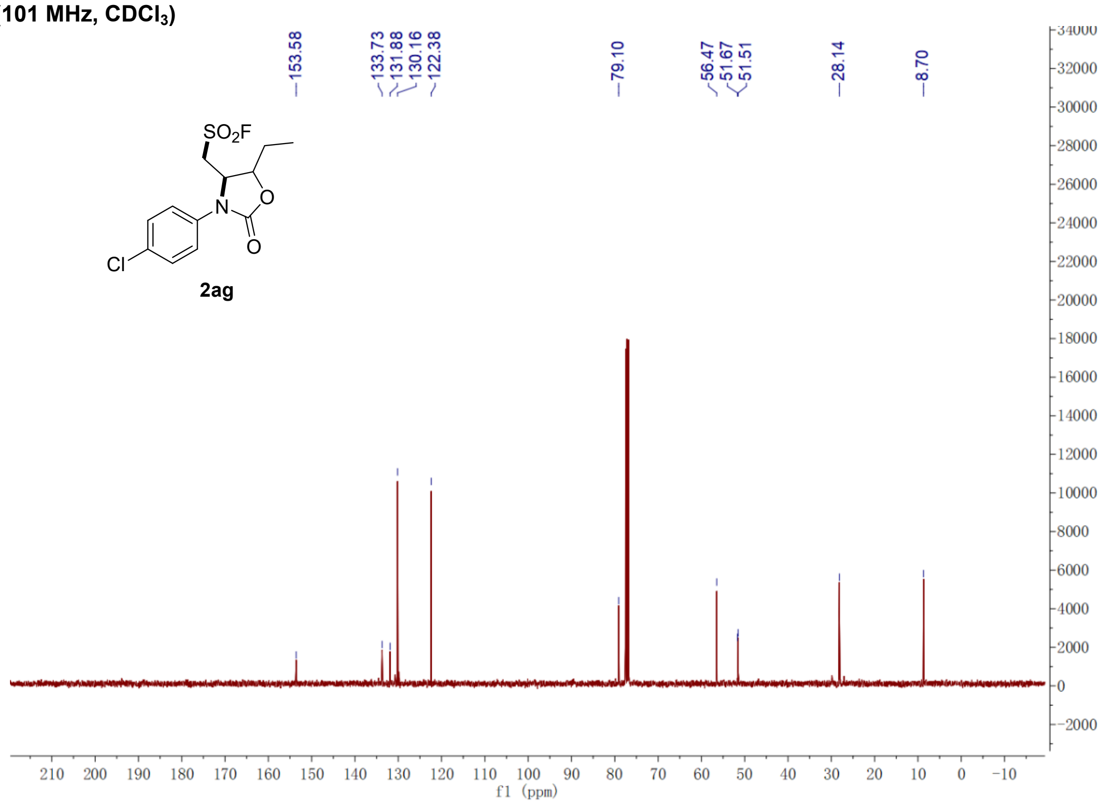

^{19}F NMR (376 MHz, $CDCl_3$)

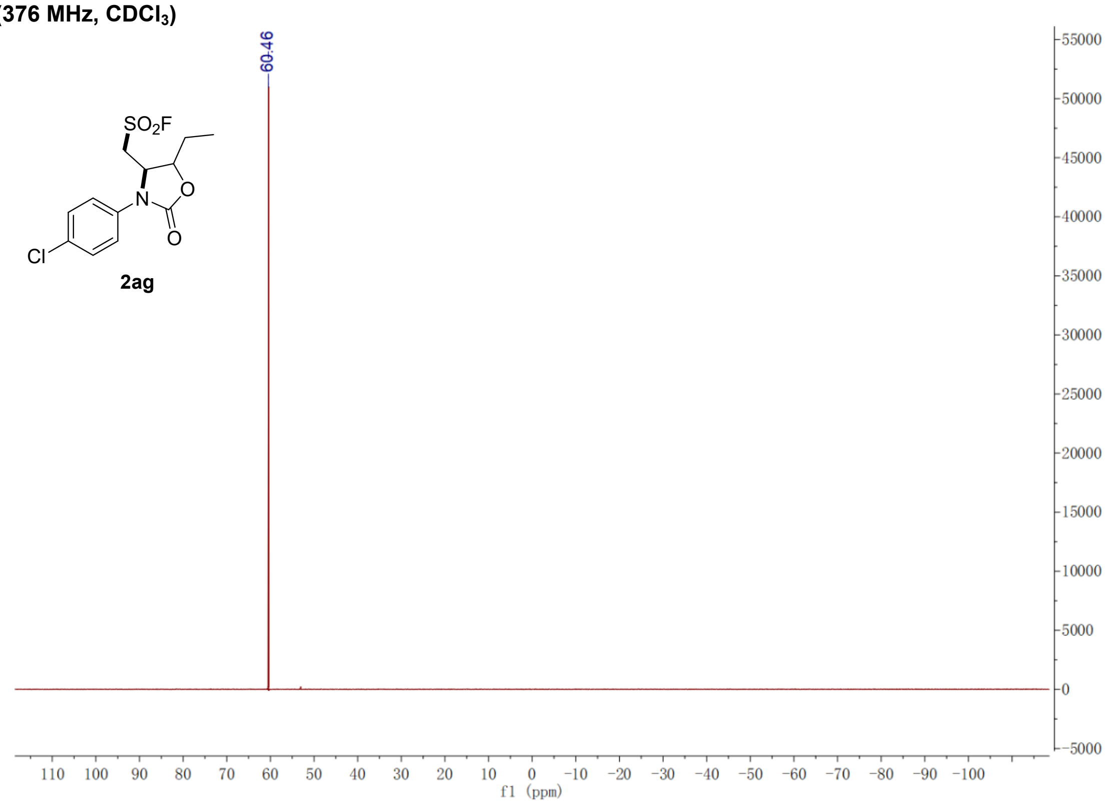

^{1}H-NMR (400 MHz, $CDCl_3$)

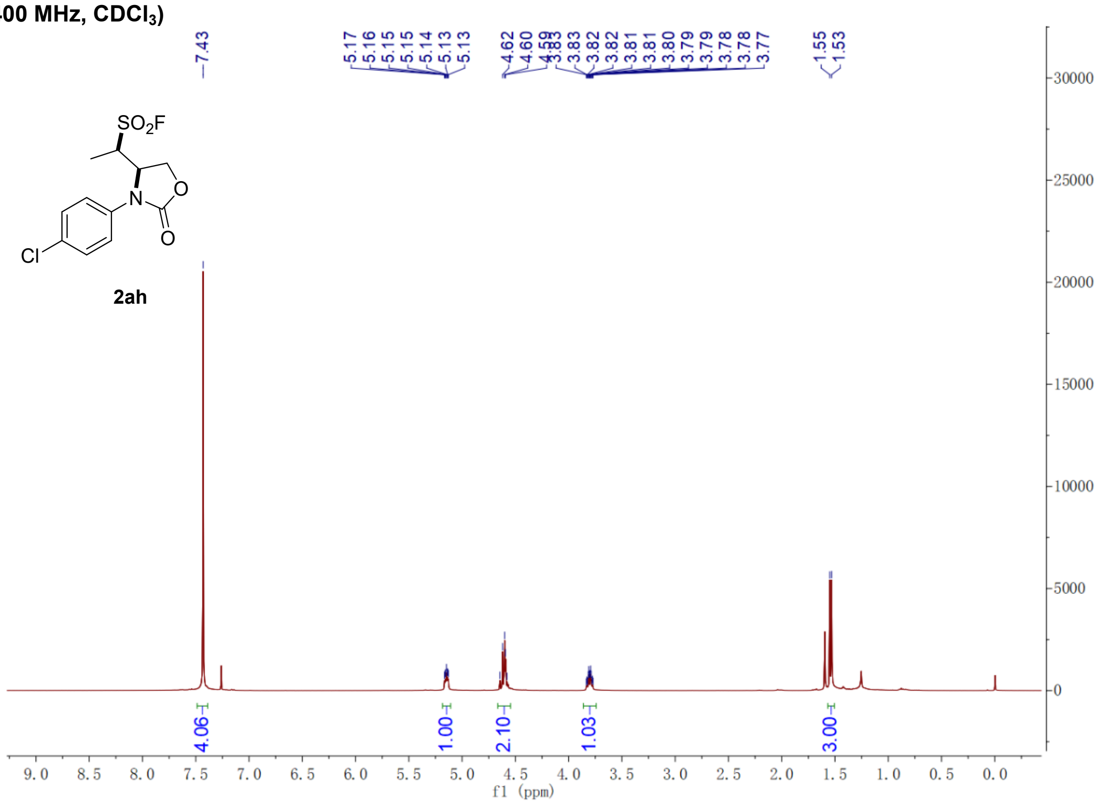

^{13}C-NMR (126 MHz, CDCl$_3$)

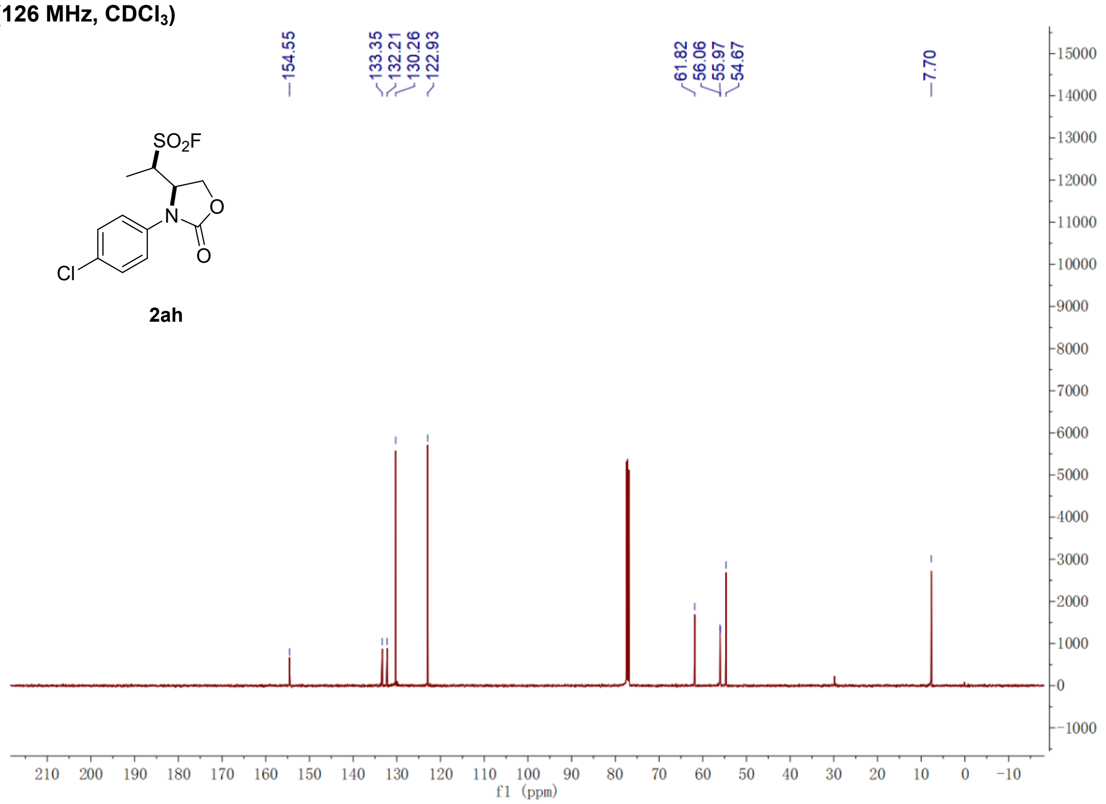

^{19}F NMR (376 MHz, CDCl$_3$)

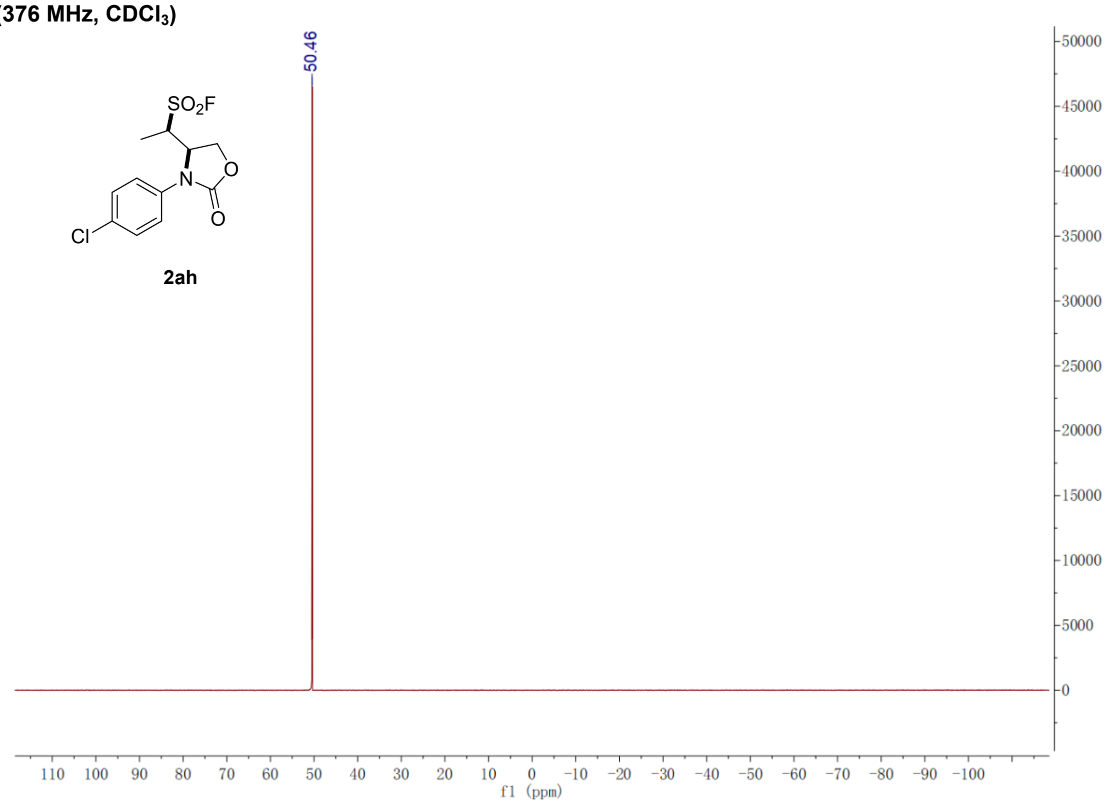

^{1}H-NMR (400 MHz, $CDCl_3$)

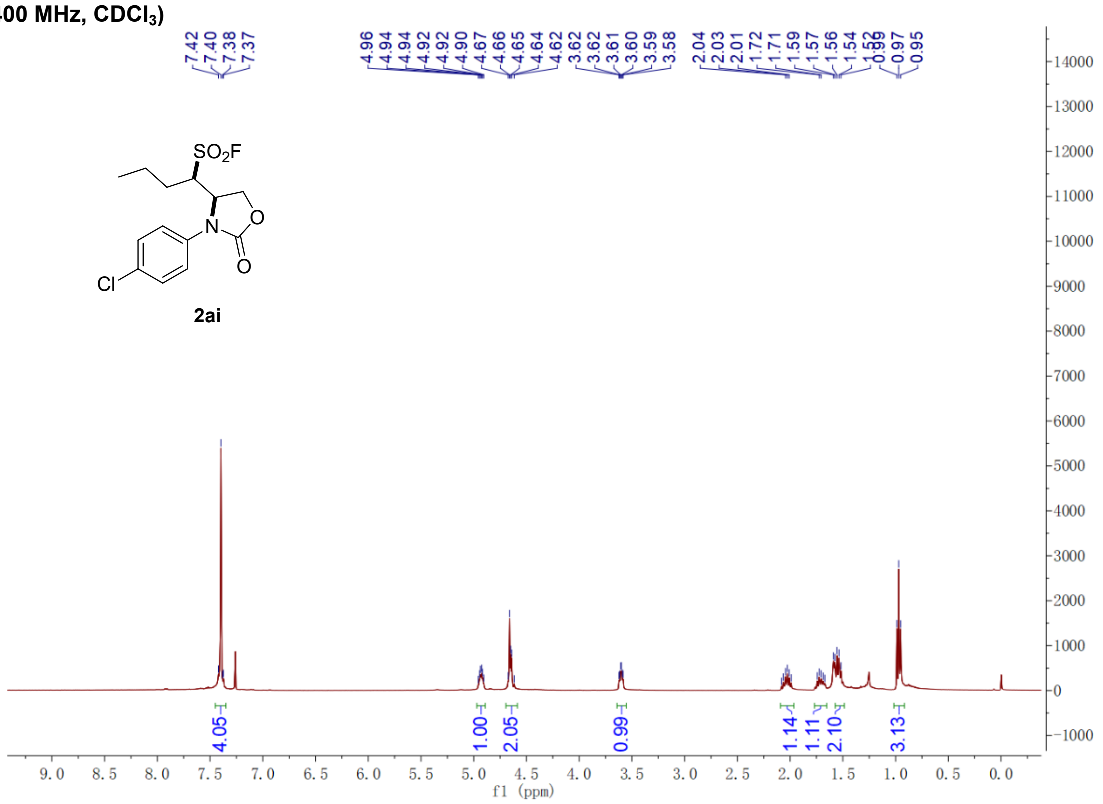

^{13}C-NMR (101 MHz, $CDCl_3$)

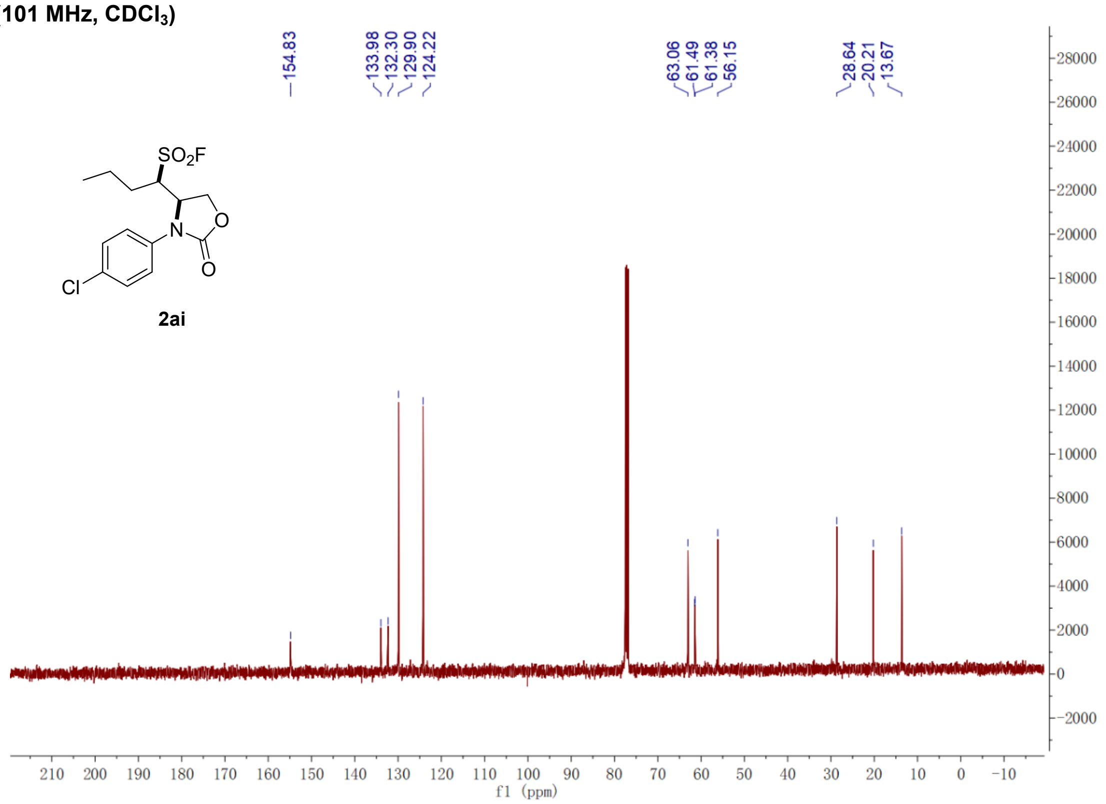

^{19}F NMR (376 MHz, $CDCl_3$)

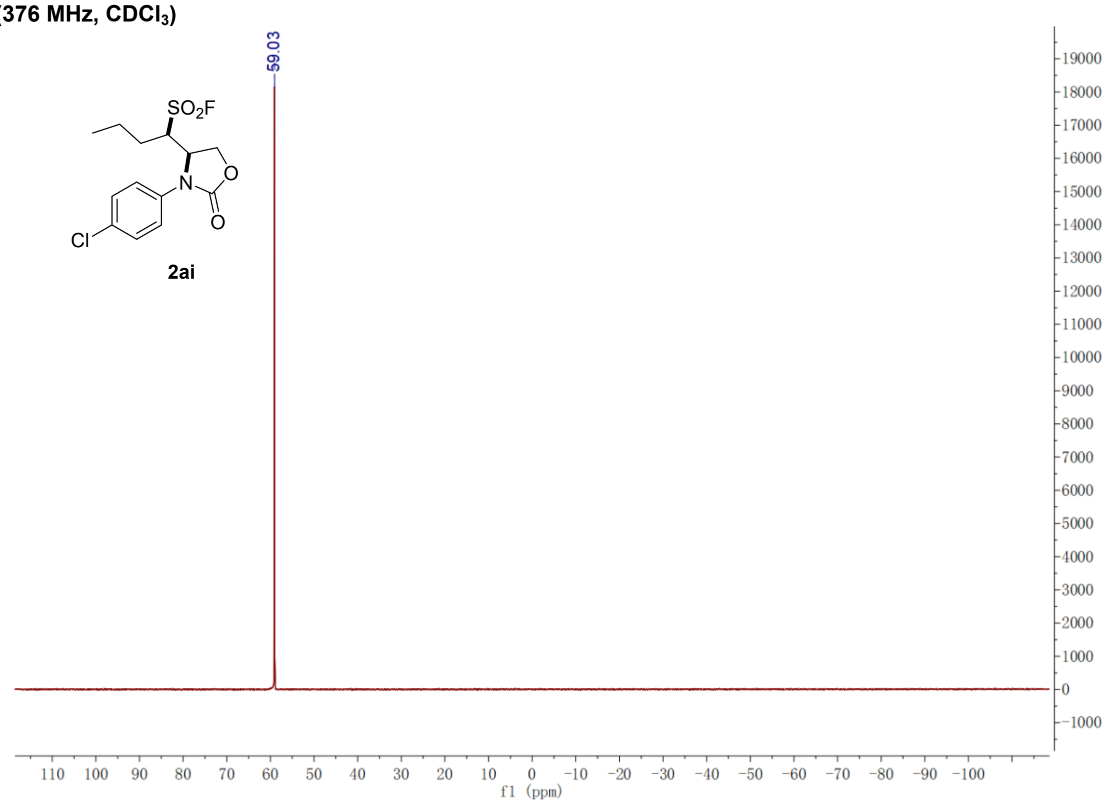

^{1}H-NMR (400 MHz, $CDCl_3$)

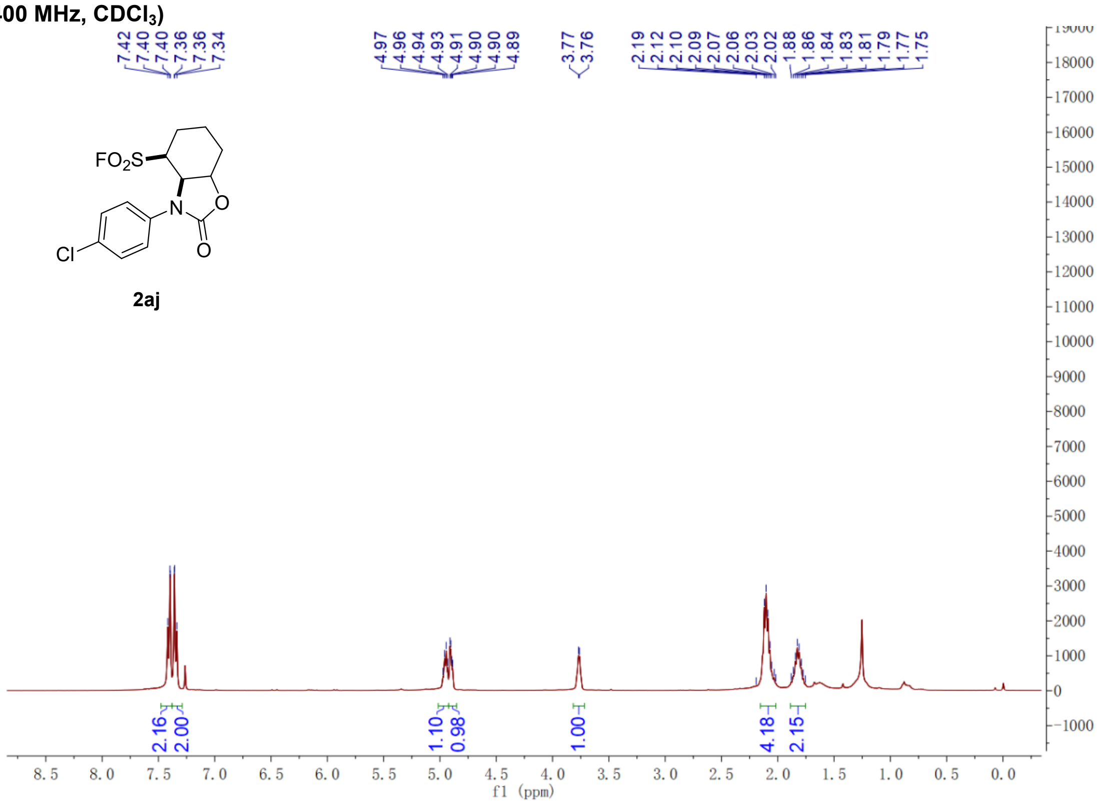

^{13}C-NMR (101 MHz, $CDCl_3$)

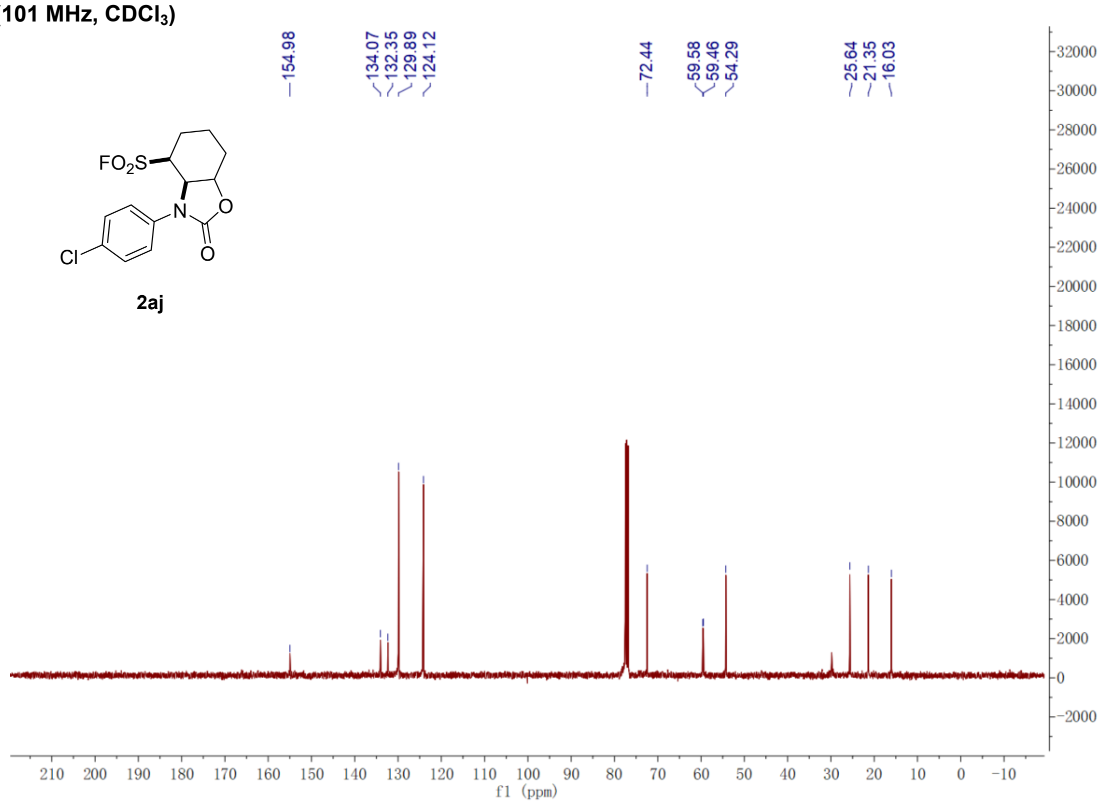

^{19}F NMR (376 MHz, $CDCl_3$)

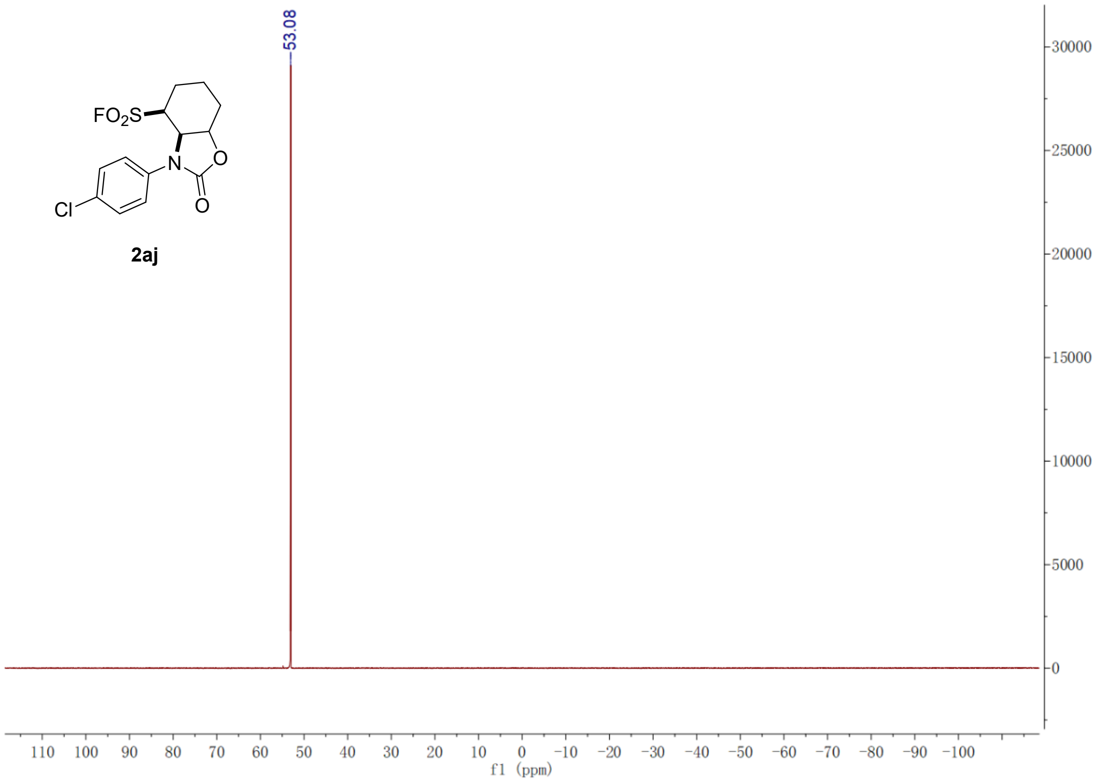

^{1}H-NMR (400 MHz, DMSO-d_6)

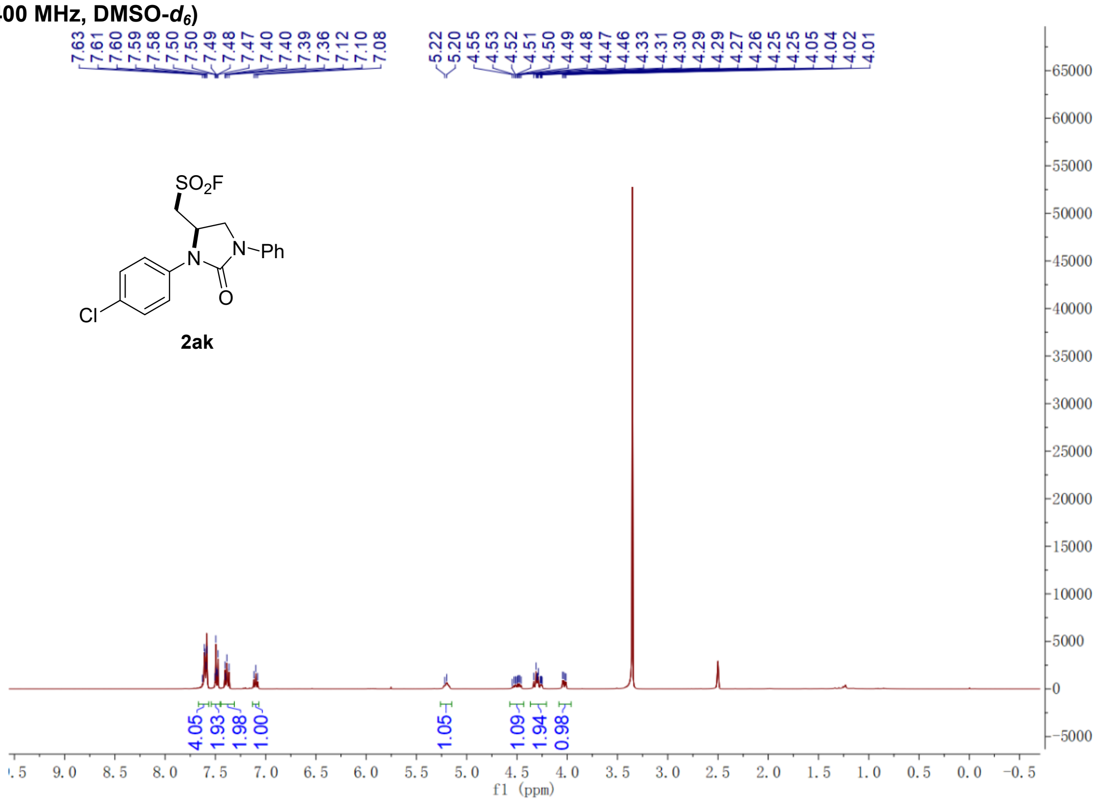

^{13}C-NMR (101 MHz, DMSO-d_6)

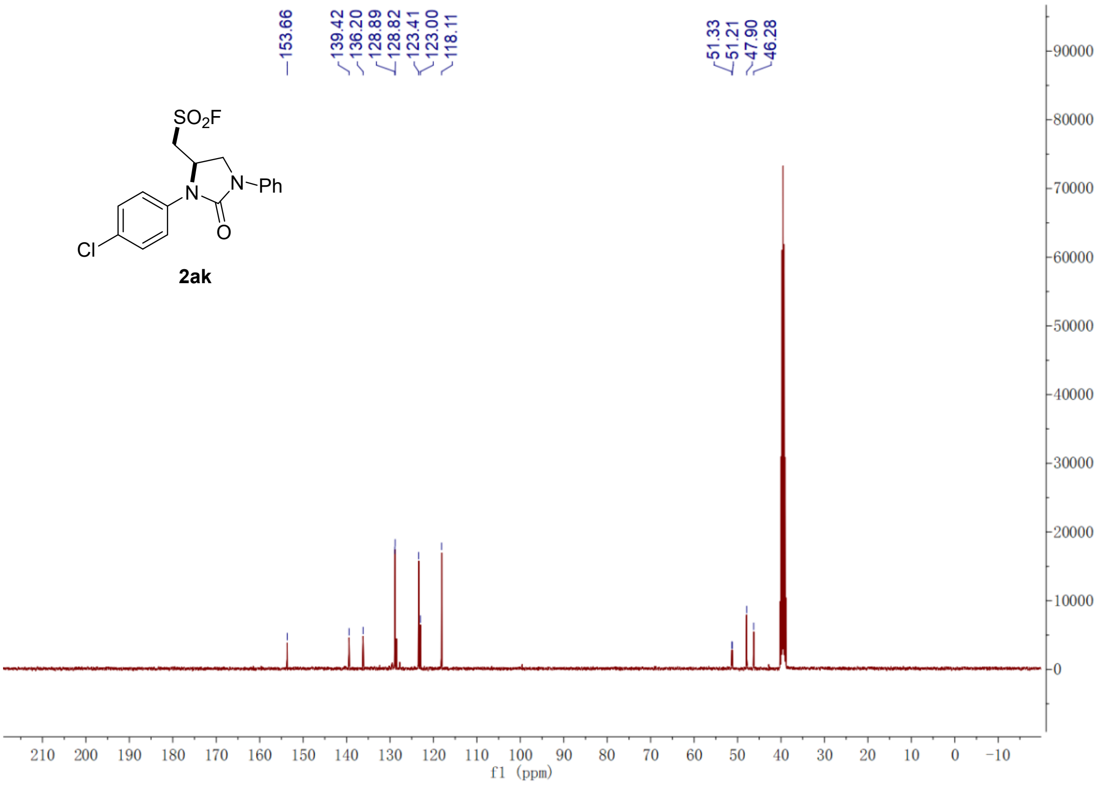

^{19}F NMR (376 MHz, DMSO-d_6)

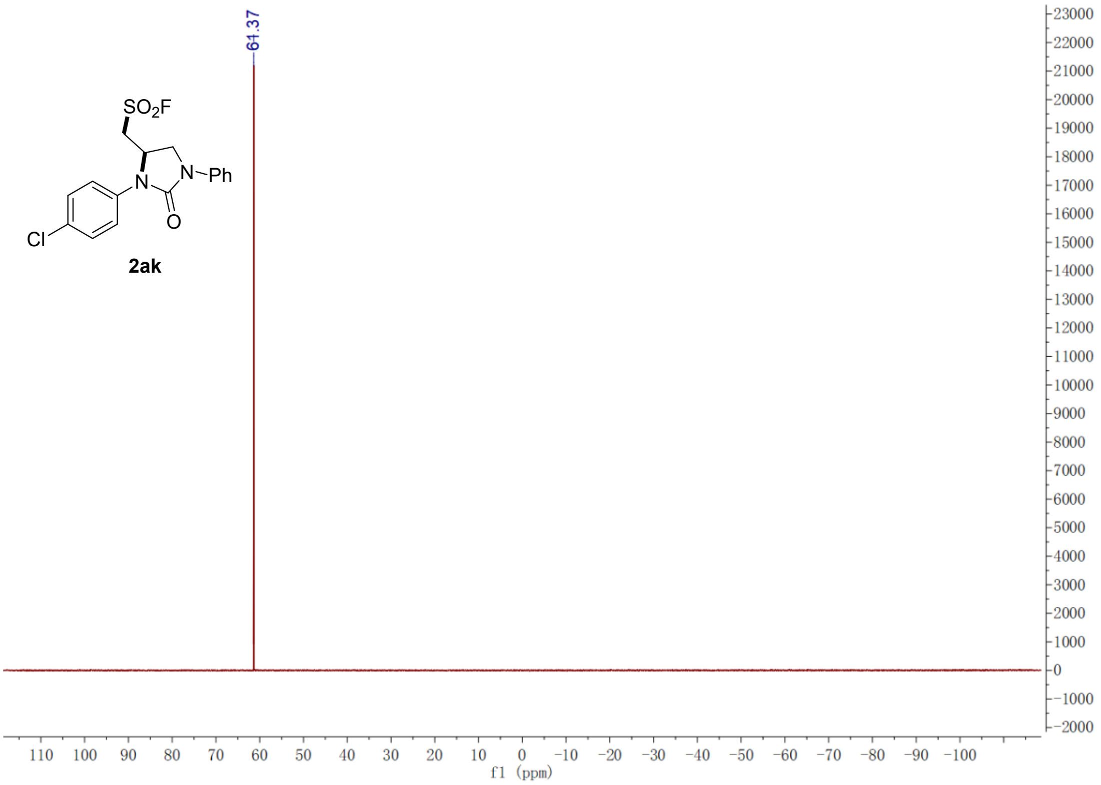

2ak

^{1}H-NMR (500 MHz, DMSO-d_6)

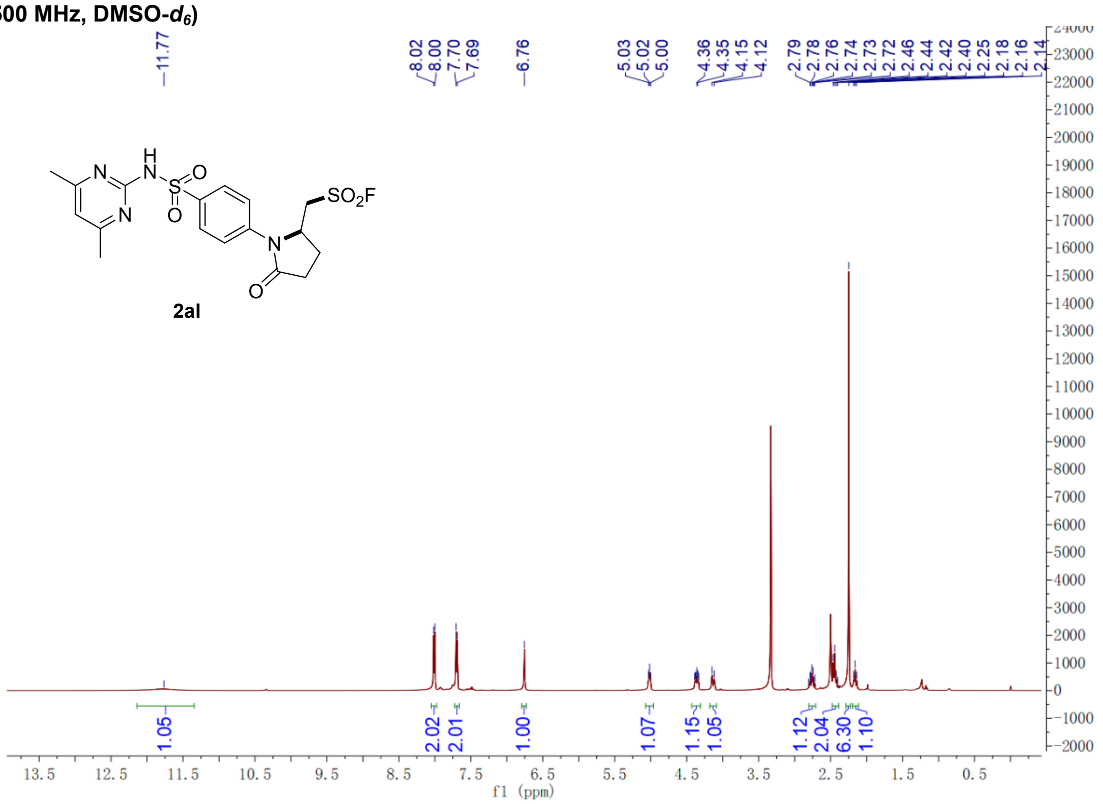

2al

^{13}C-NMR (126 MHz, DMSO-d_6)

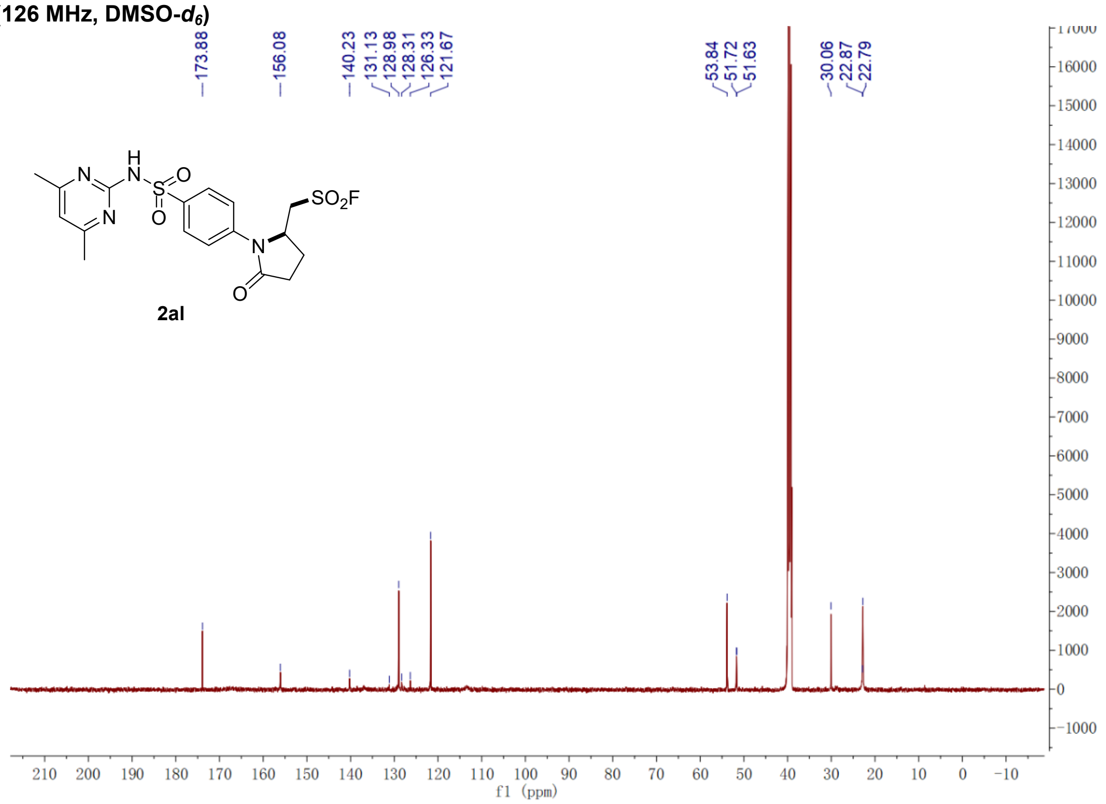

^{19}F NMR (376 MHz, DMSO-d_6)

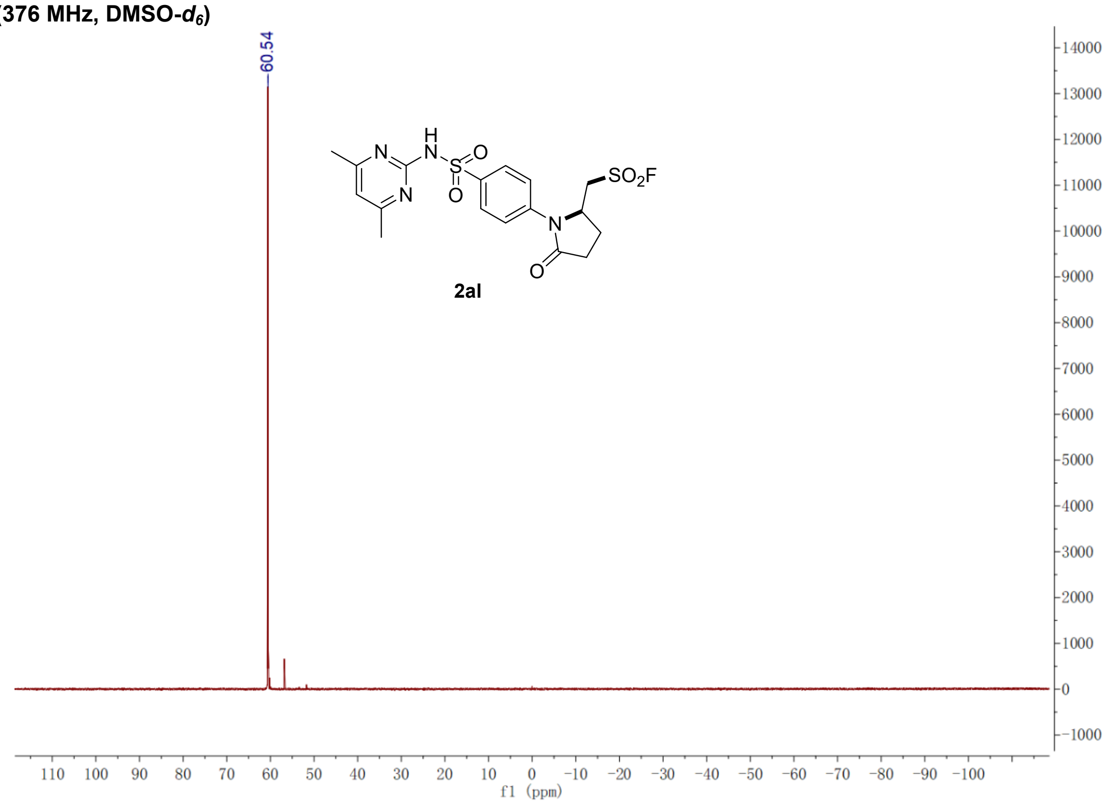

^{1}H-NMR (400 MHz, DMSO-d_6)

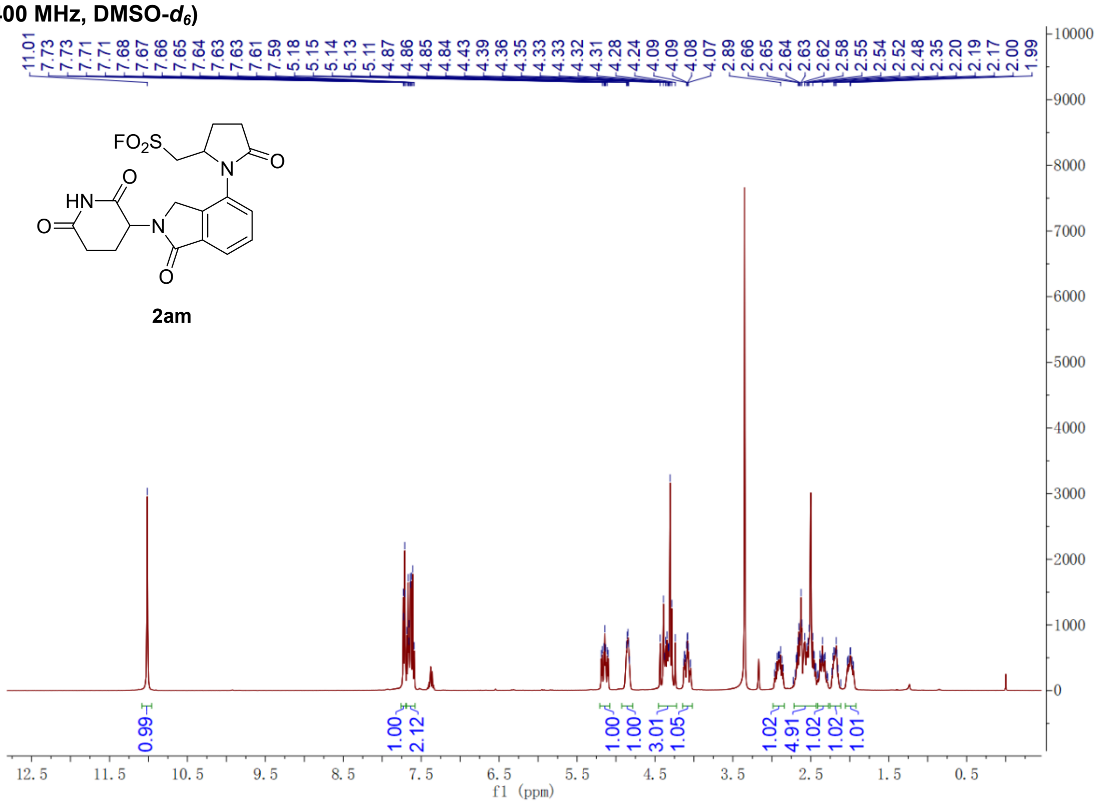

^{13}C-NMR (101 MHz, DMSO-d_6)

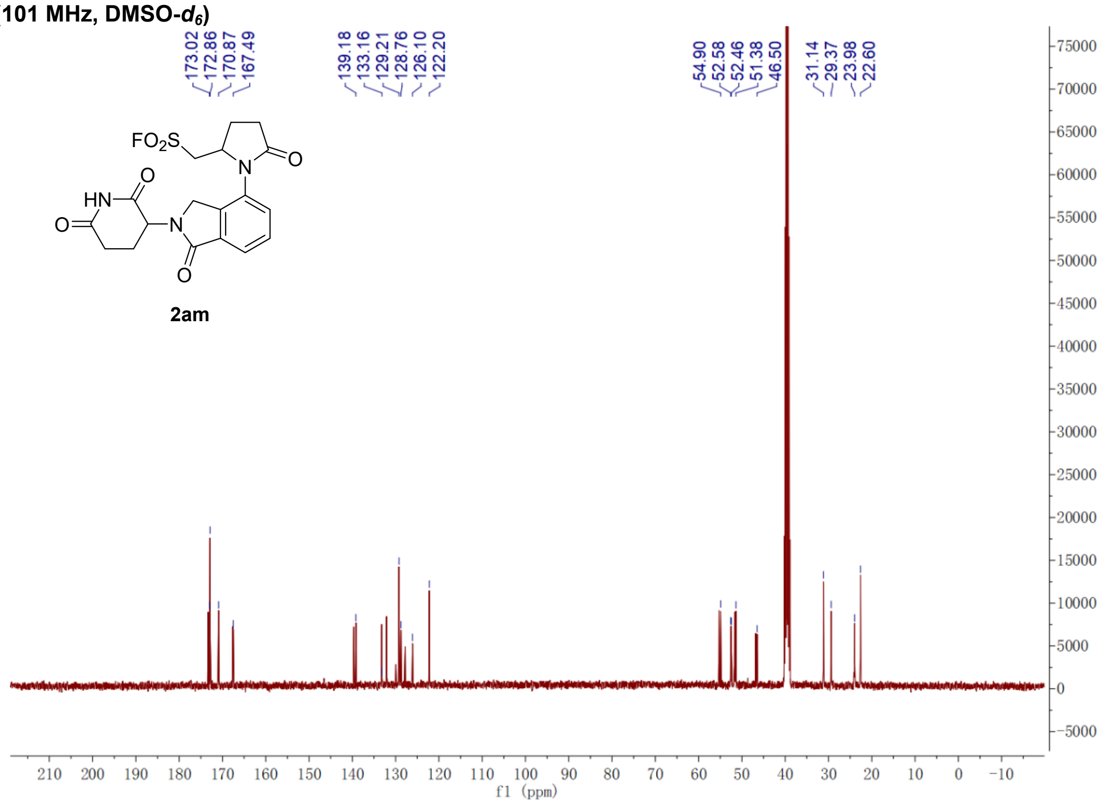

^{19}F NMR (376 MHz, DMSO-d_6)

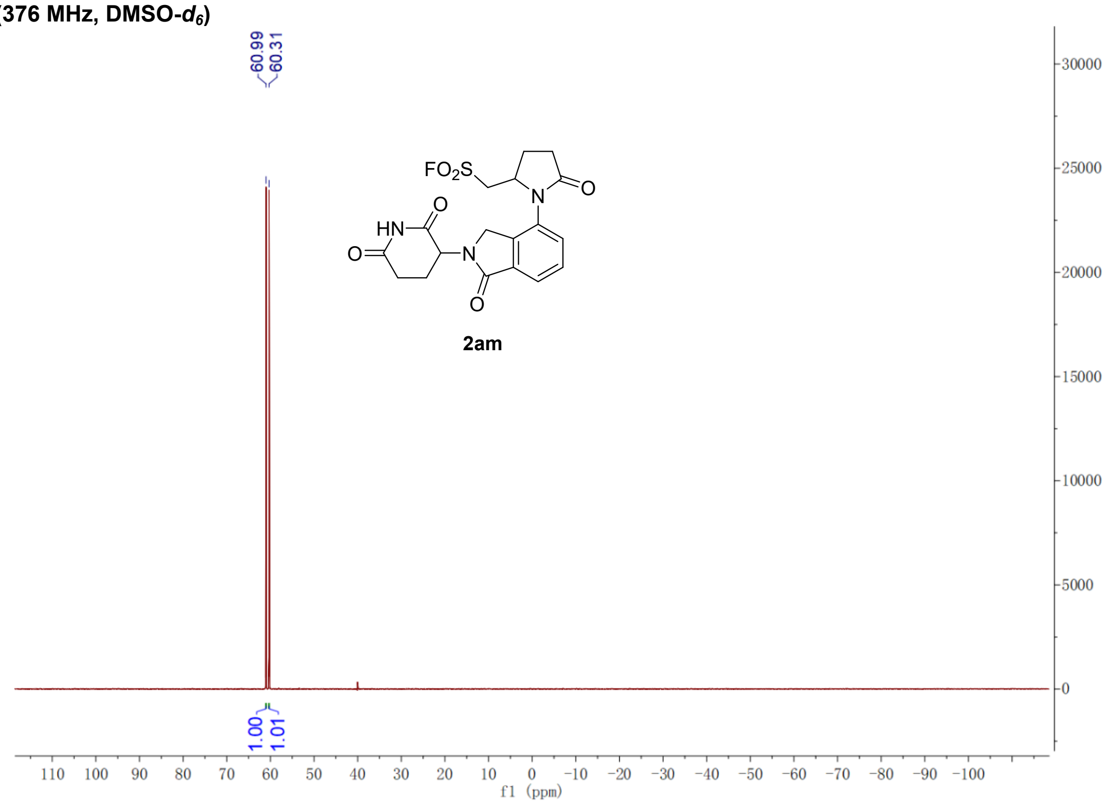

^{1}H-NMR (400 MHz, CDCl$_3$)

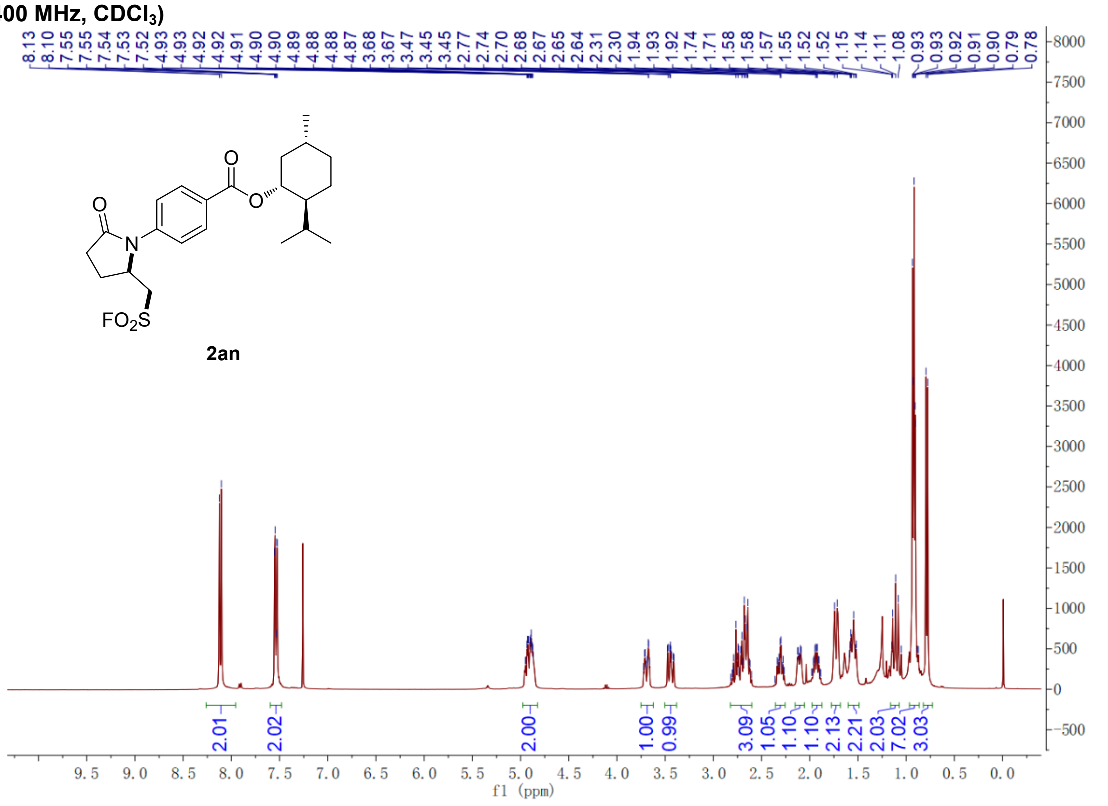

^{13}C-NMR (101 MHz, CDCl$_3$)

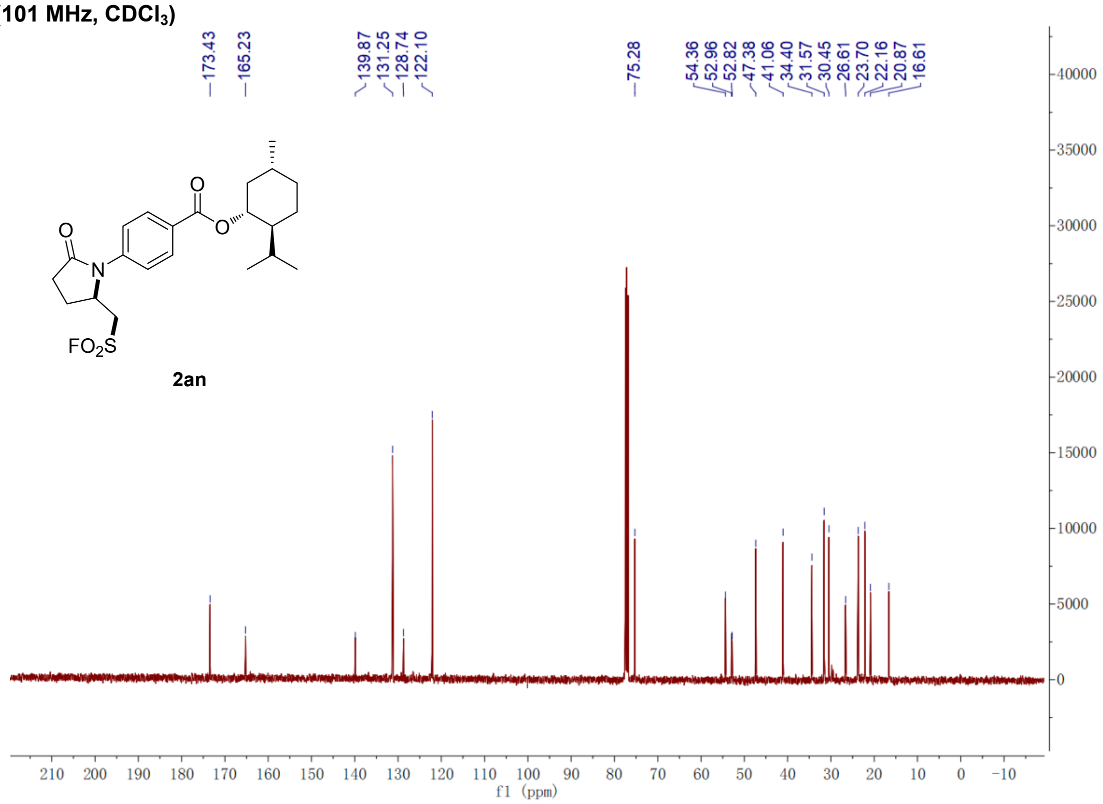

^{19}F NMR (376 MHz, CDCl$_3$)

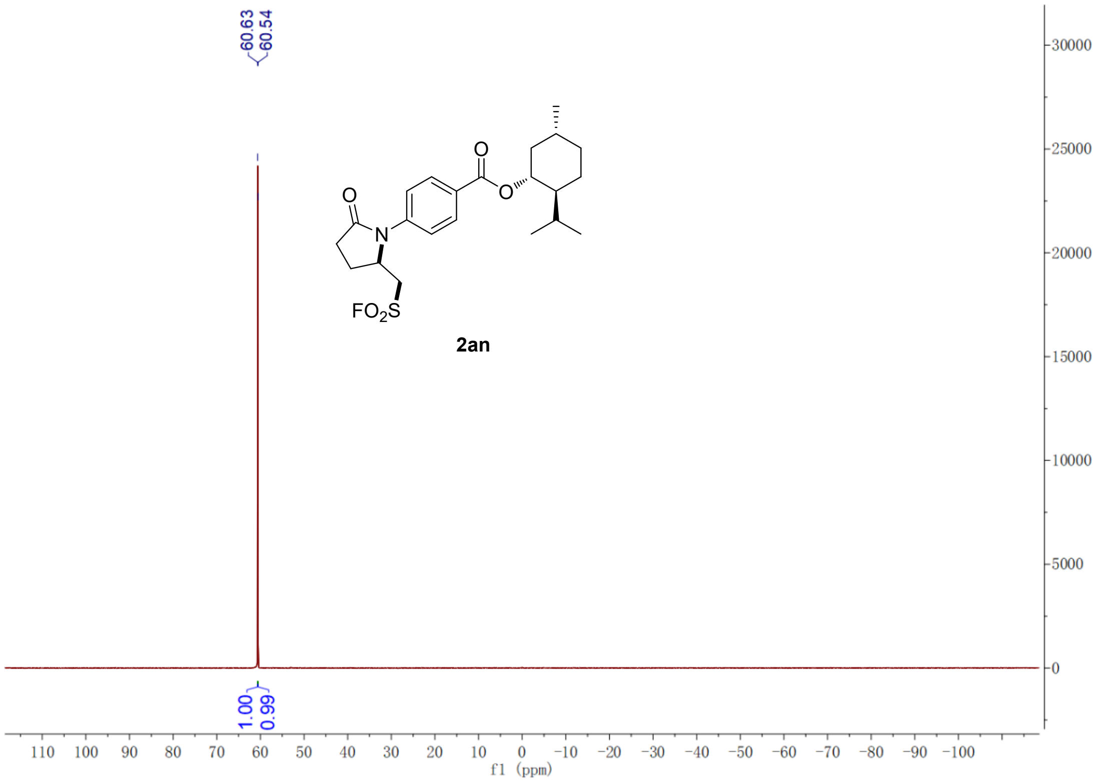

^{1}H-NMR (400 MHz, $CDCl_3$)

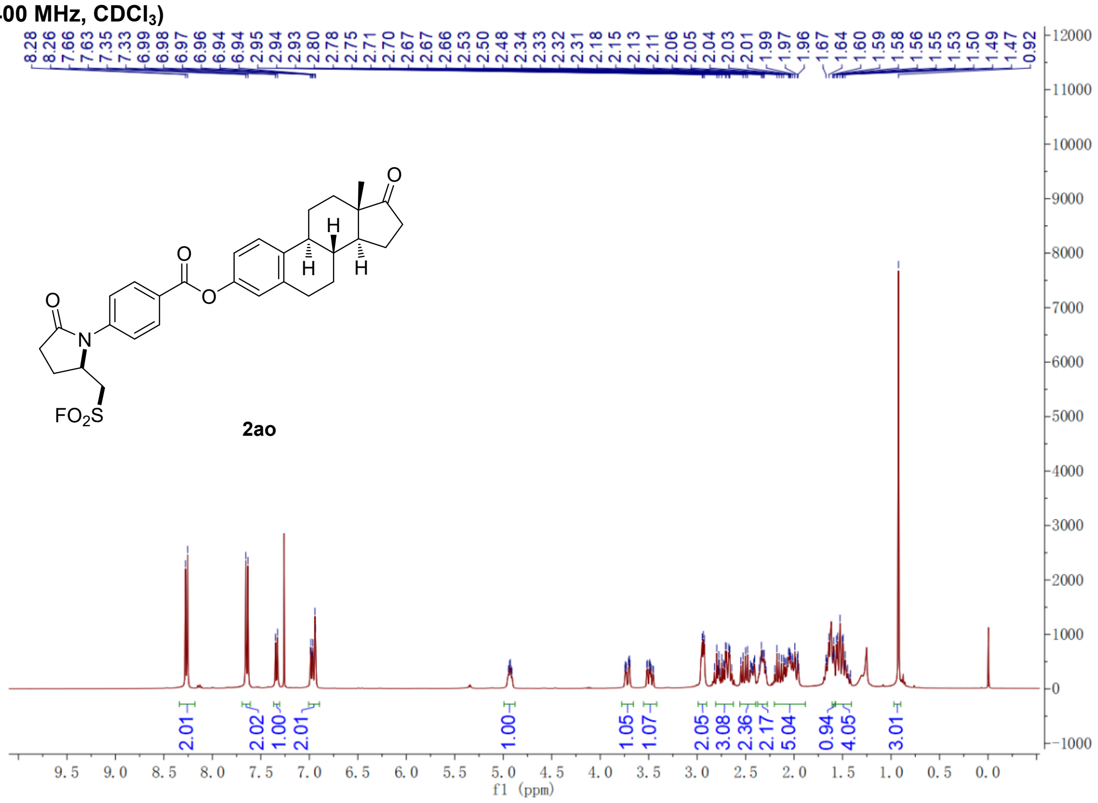

^{13}C-NMR (126 MHz, $CDCl_3$)

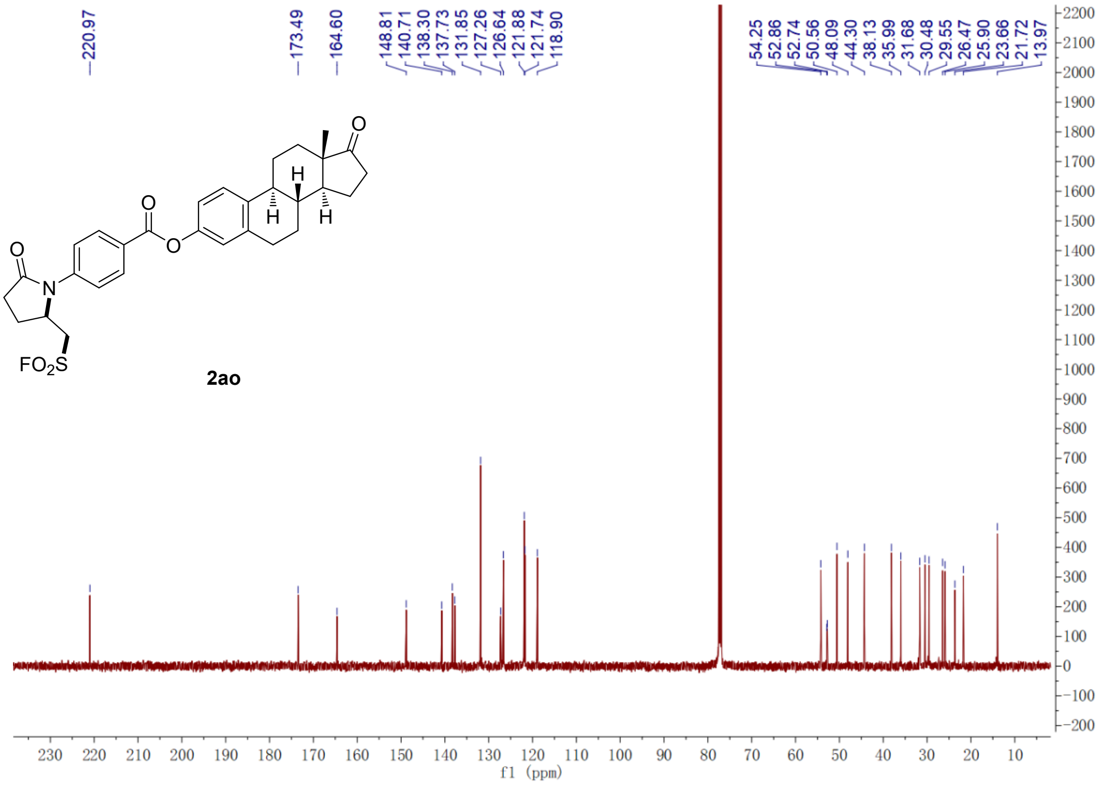

^{19}F NMR (376 MHz, $CDCl_3$)

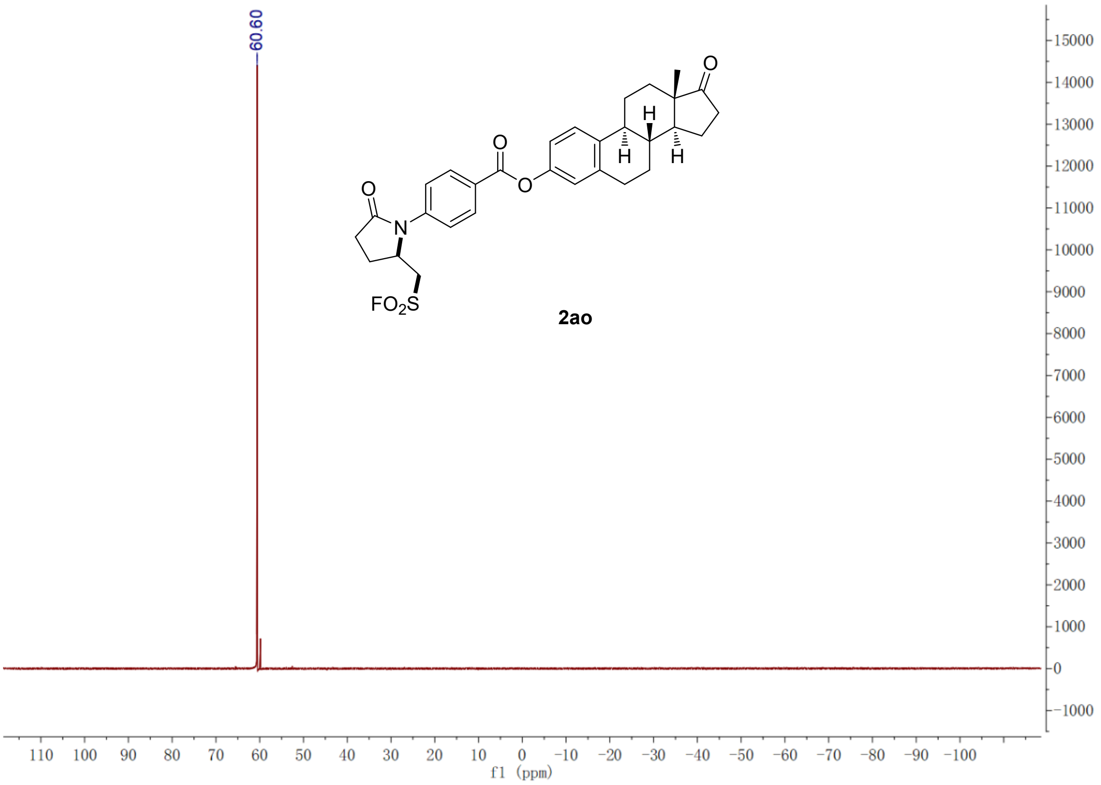

^{1}H-NMR (400 MHz, $CDCl_3$)

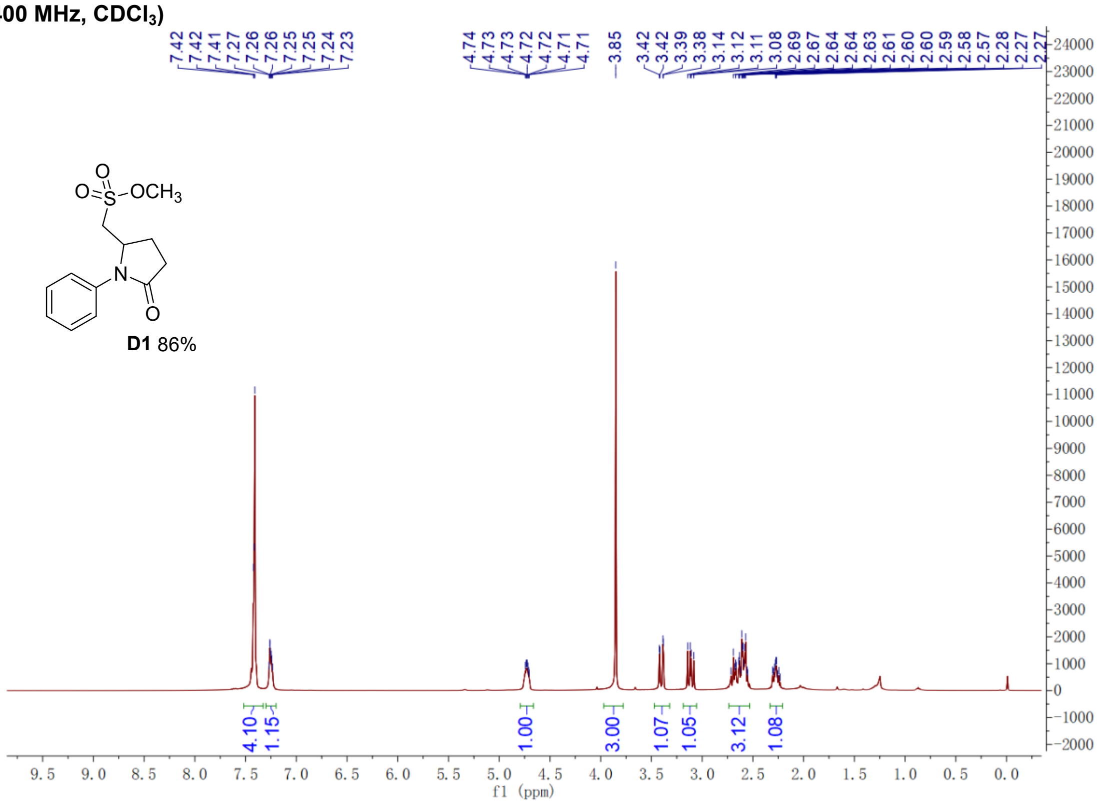

^{13}C-NMR (101 MHz, $CDCl_3$)

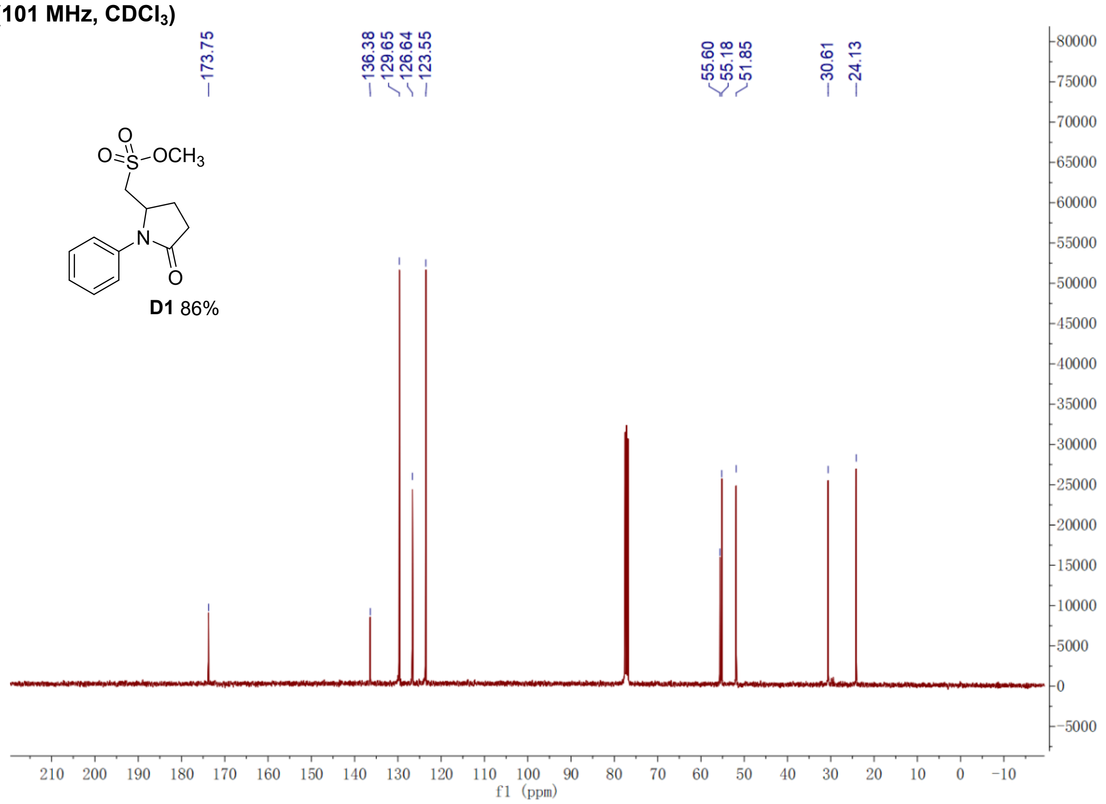

^{1}H-NMR (400 MHz, $CDCl_3$)

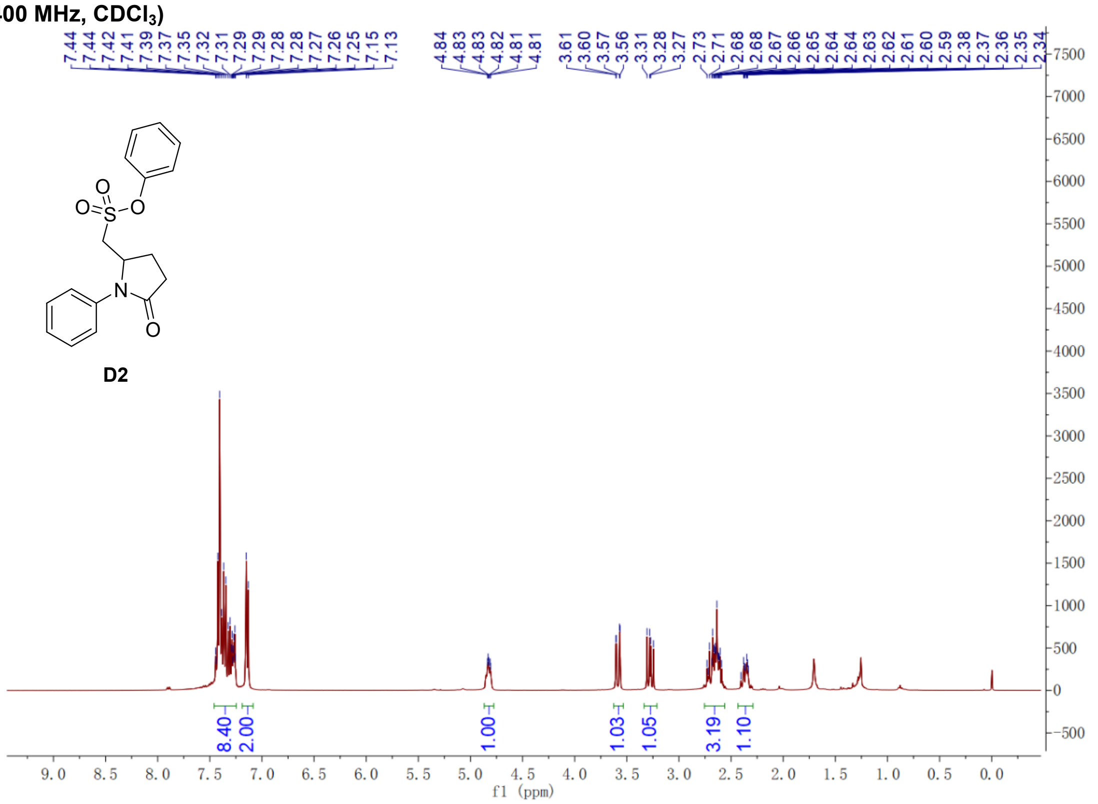

^{13}C-NMR (101 MHz, $CDCl_3$)

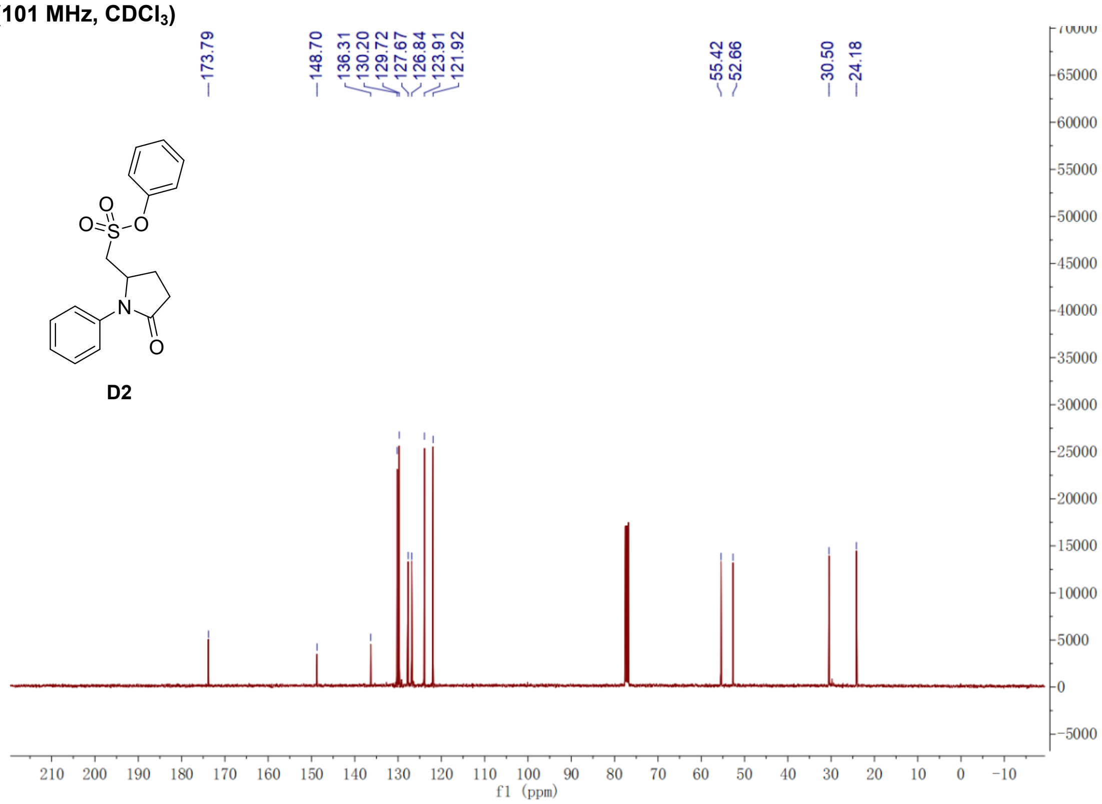

^{1}H-NMR (400 MHz, $CDCl_3$)

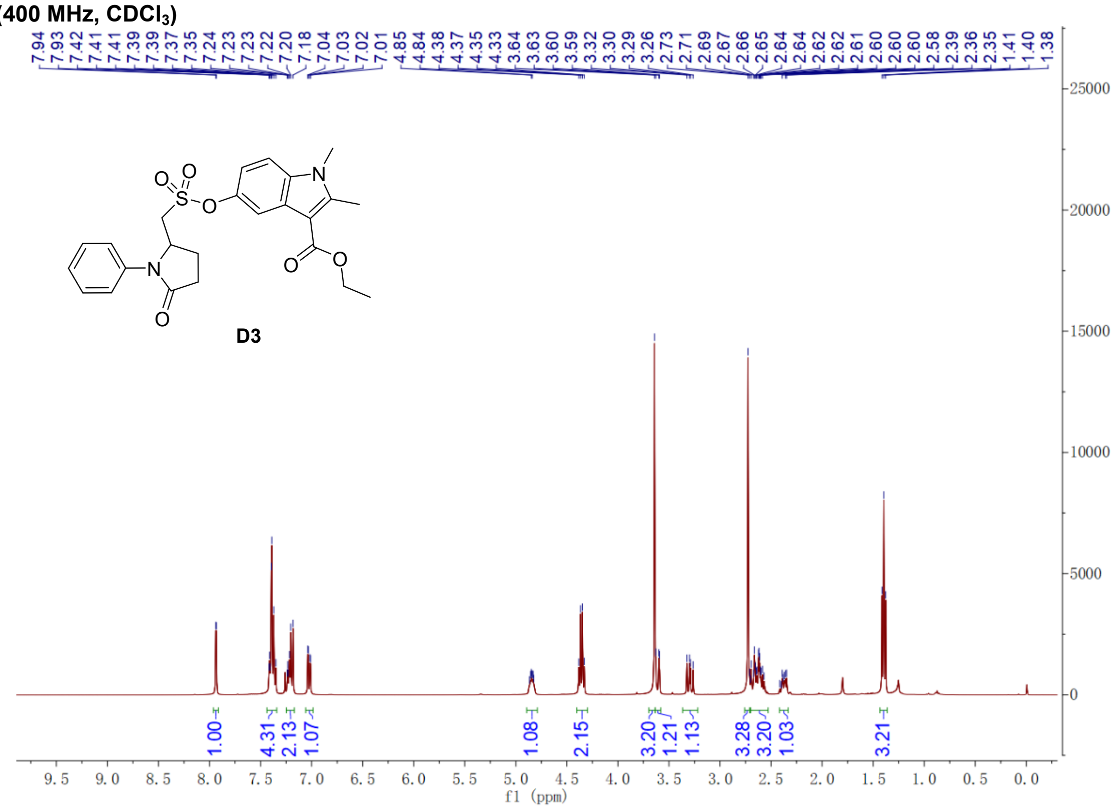

^{13}C-NMR (101 MHz, $CDCl_3$)

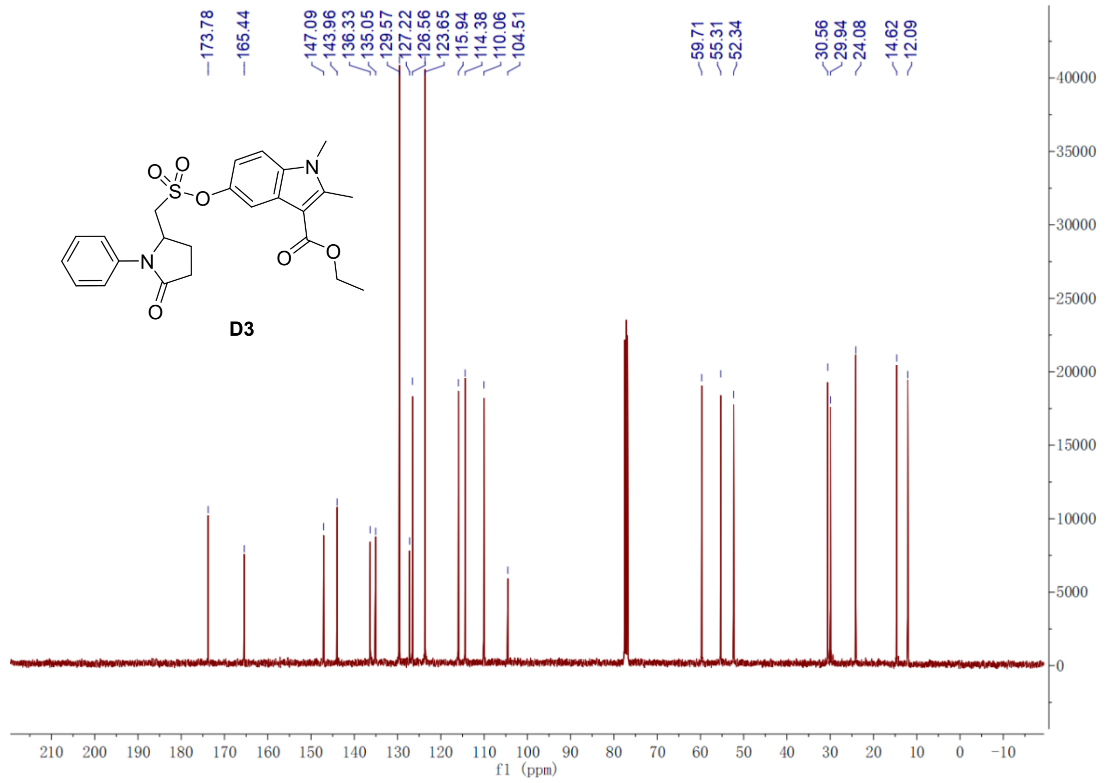

^{1}H-NMR (400 MHz, CDCl$_3$)

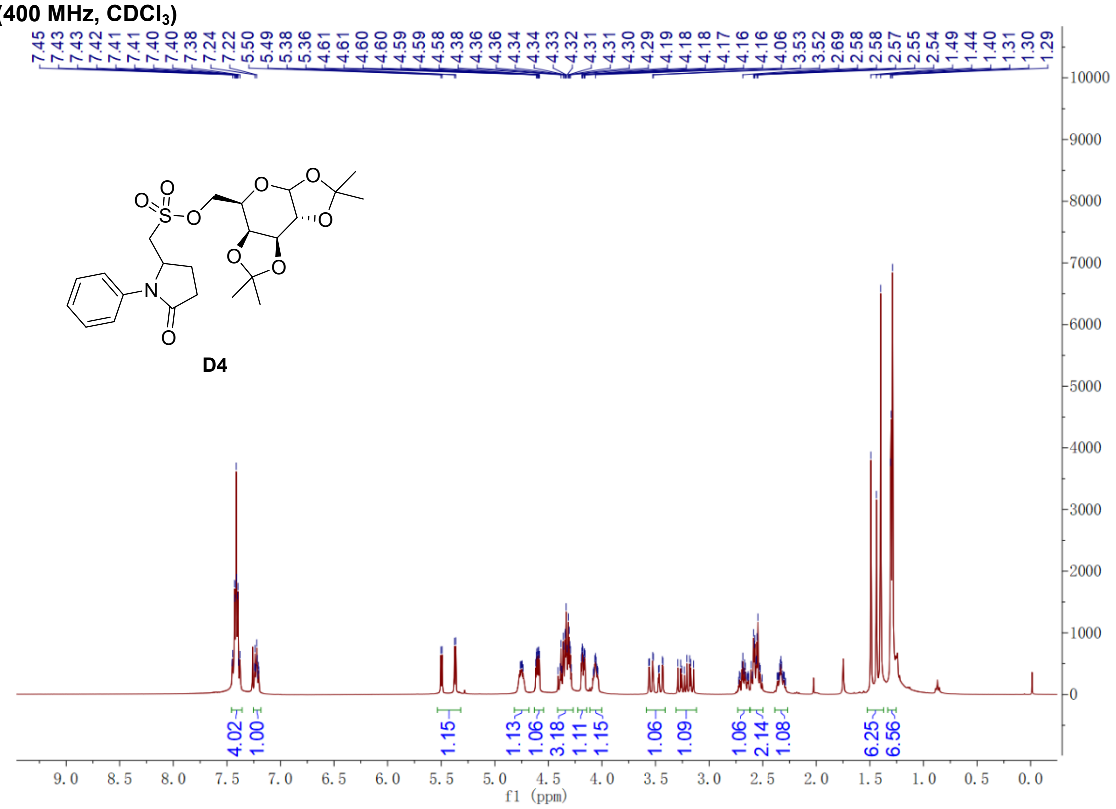

^{13}C-NMR (126 MHz, CDCl$_3$)

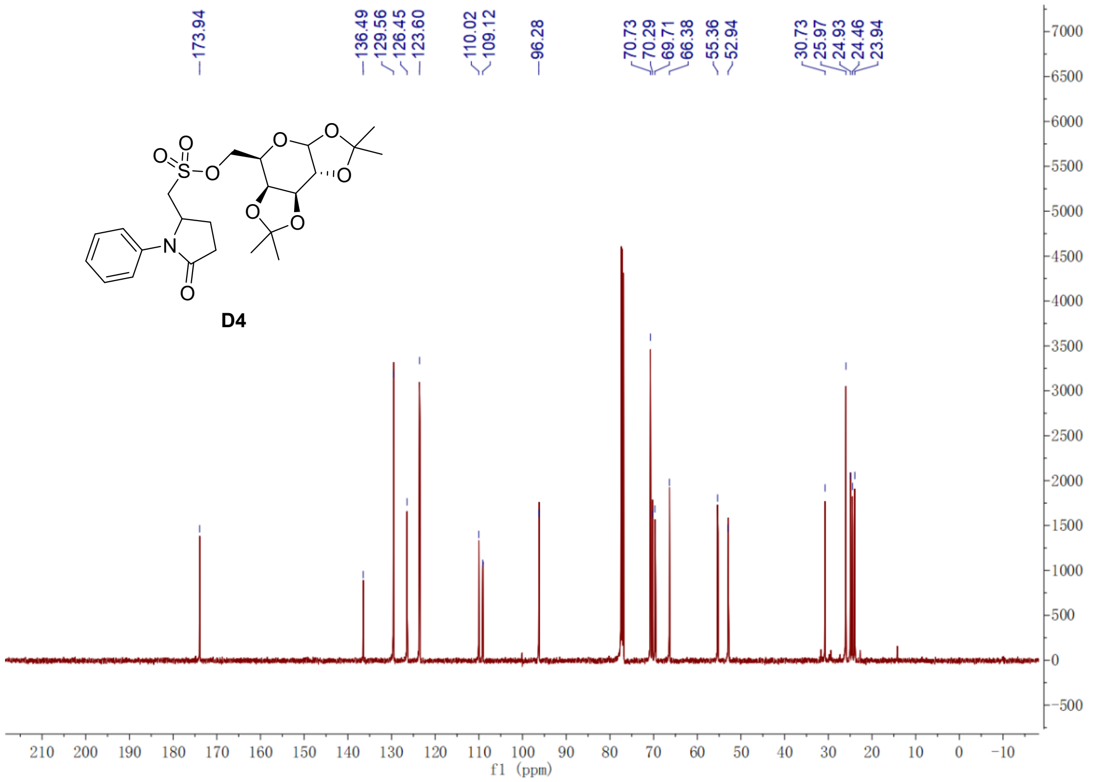

^{1}H-NMR (400 MHz, DMSO-d_6)

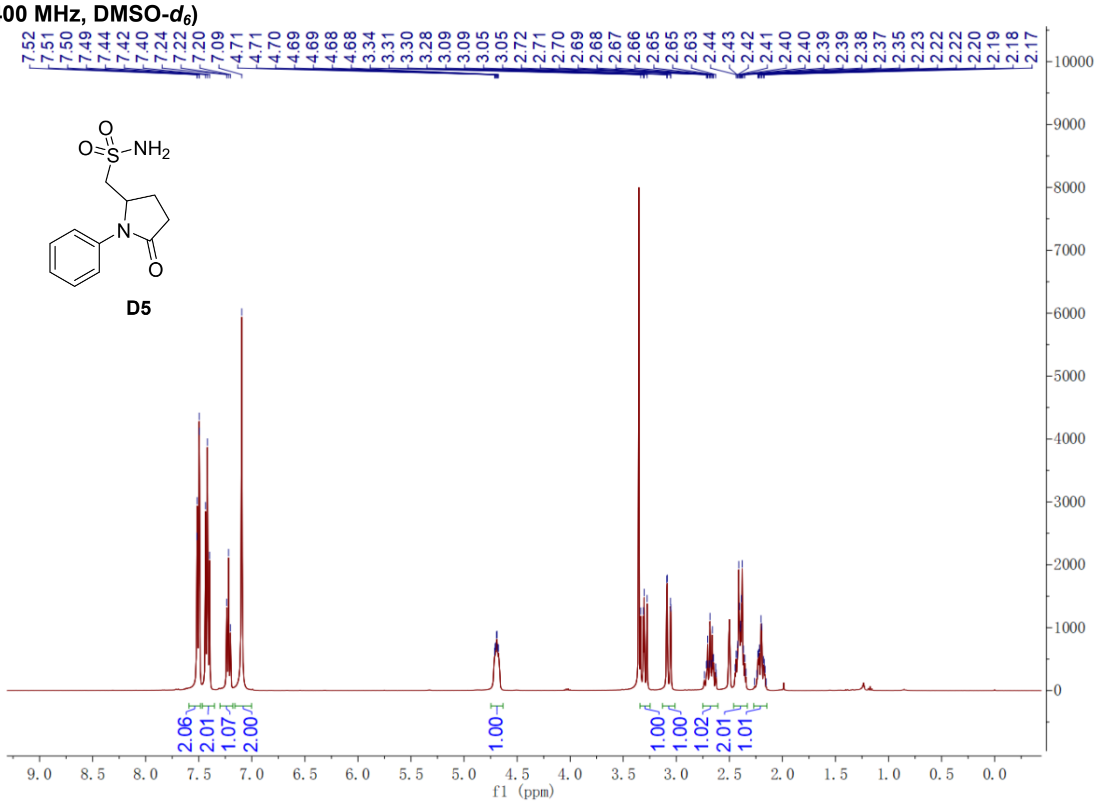

^{13}C-NMR (101 MHz, DMSO-d_6)

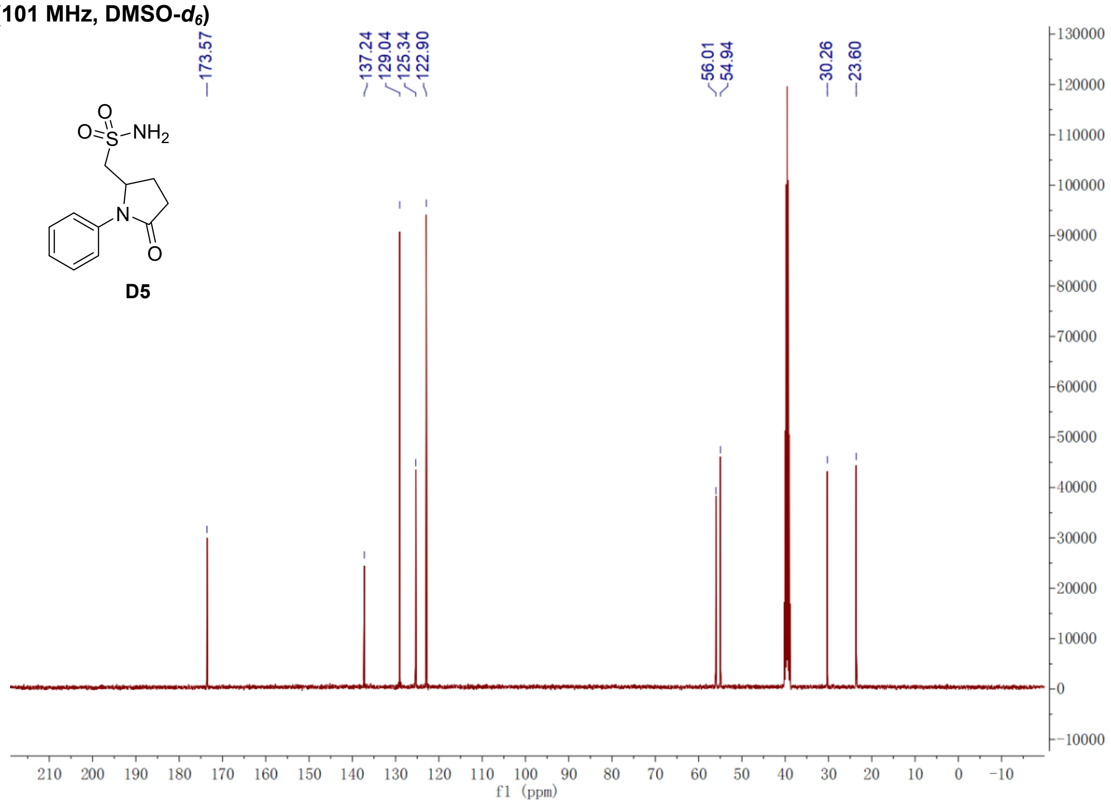

^{1}H-NMR (400 MHz, $CDCl_3$)

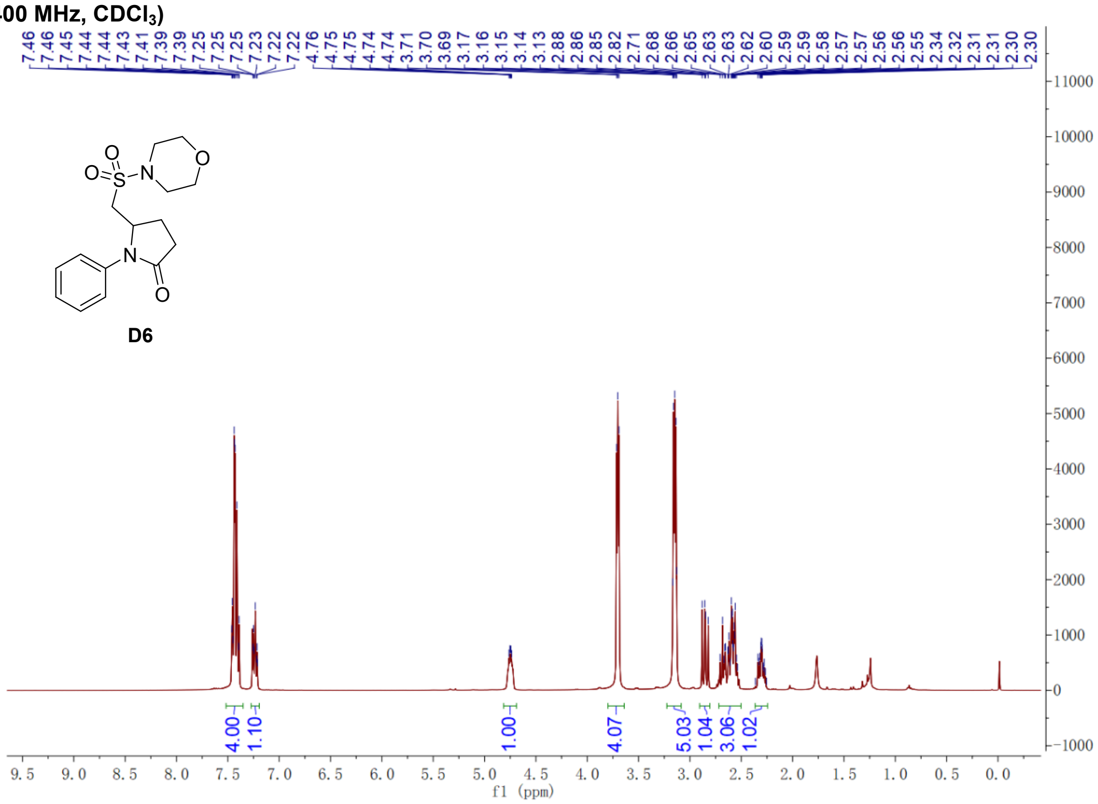

^{13}C-NMR (101 MHz, $CDCl_3$)

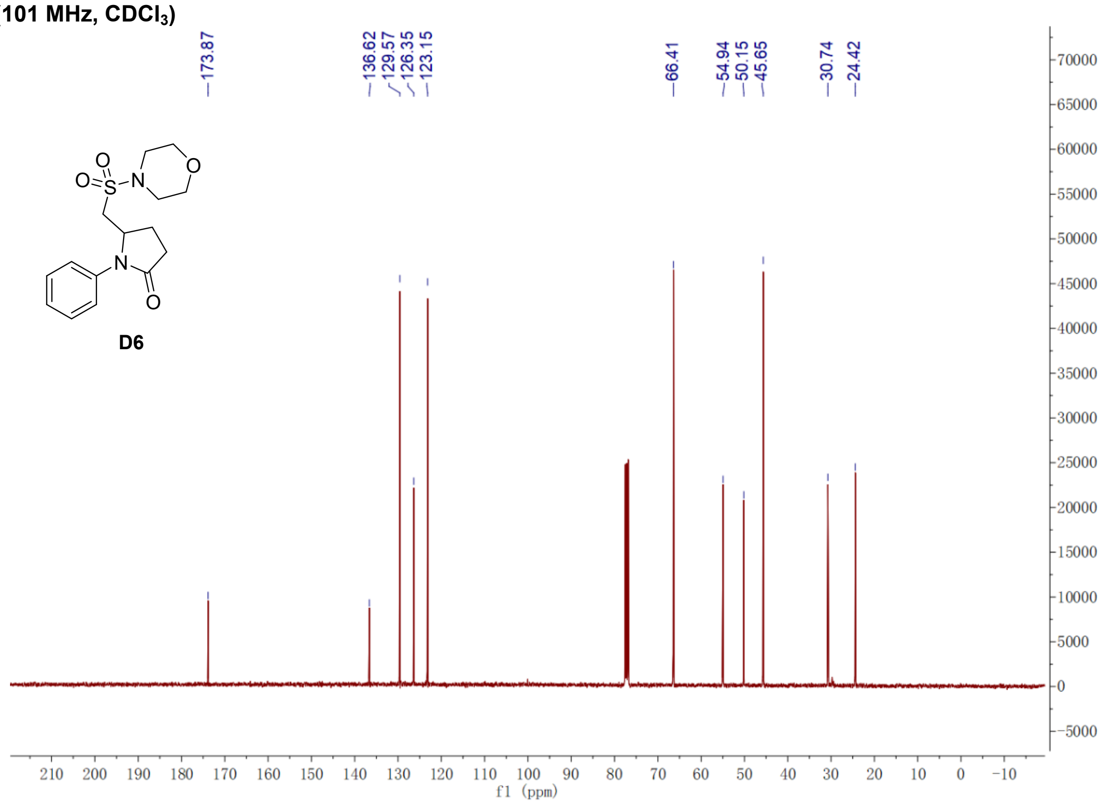

^{1}H-NMR (400 MHz, $CDCl_3$)

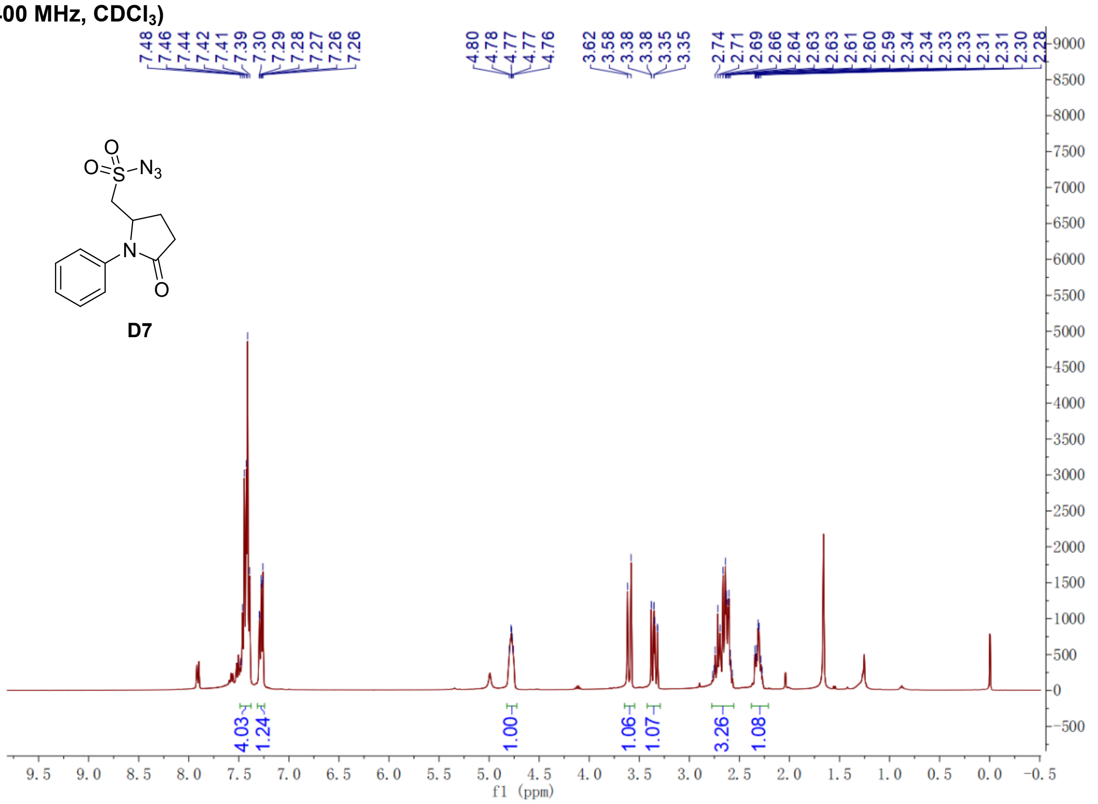

^{13}C-NMR (101 MHz, $CDCl_3$)

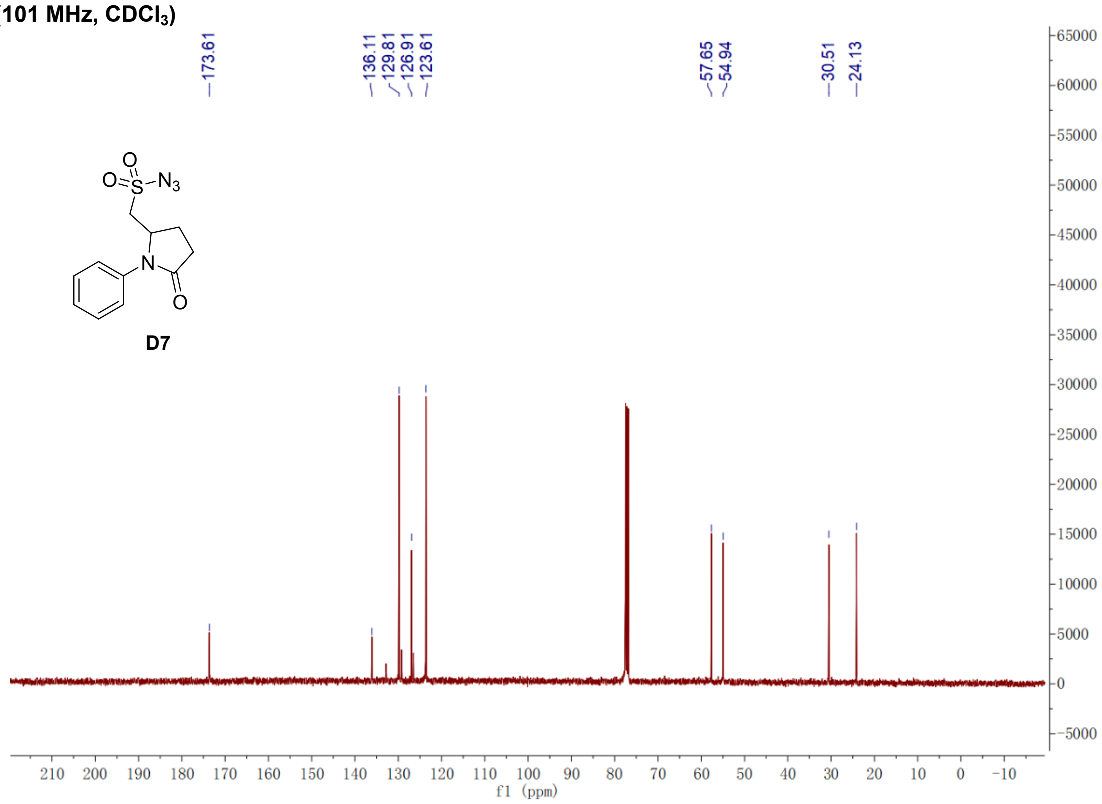

^{1}H-NMR (400 MHz, $CDCl_3$)

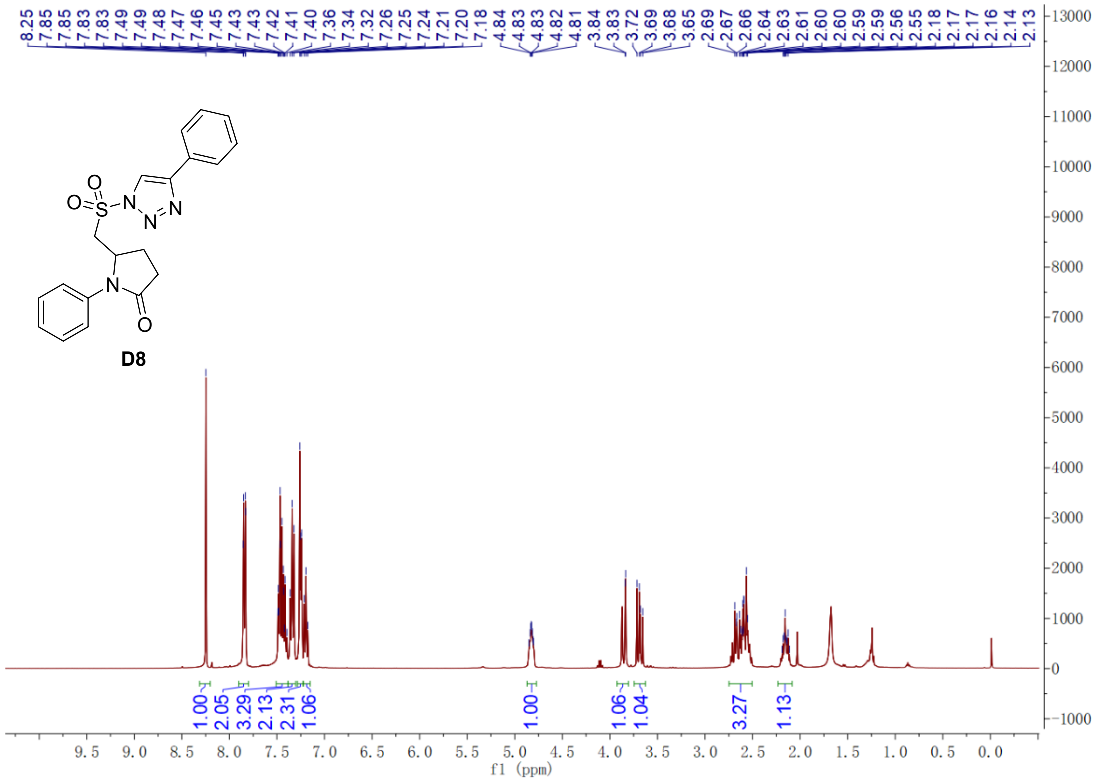

^{13}C-NMR (101 MHz, $CDCl_3$)

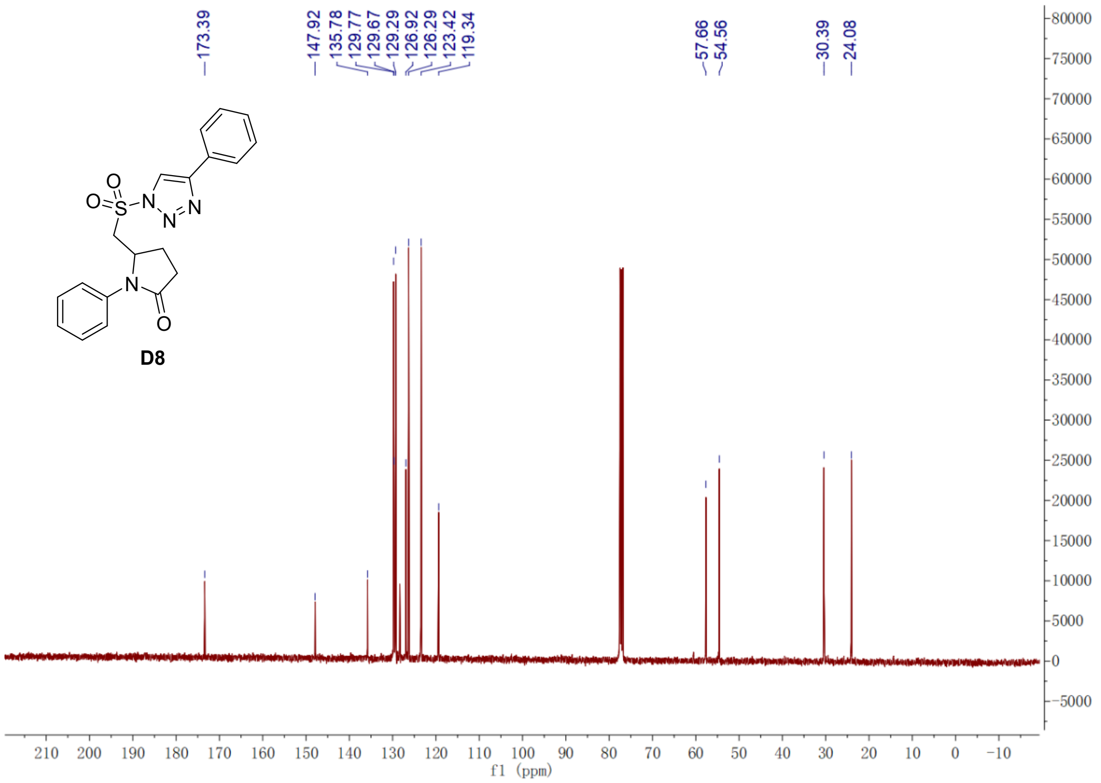

^{1}H-NMR (400 MHz, $CDCl_3$)

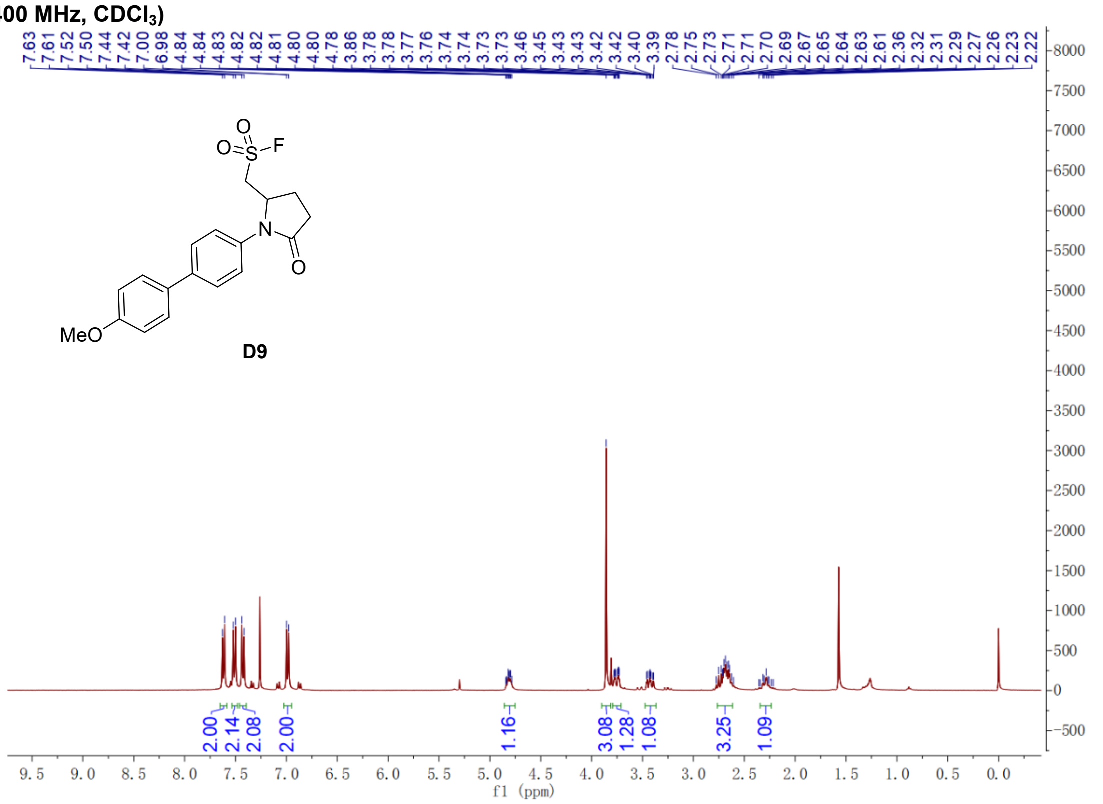

^{13}C-NMR (101 MHz, $CDCl_3$)

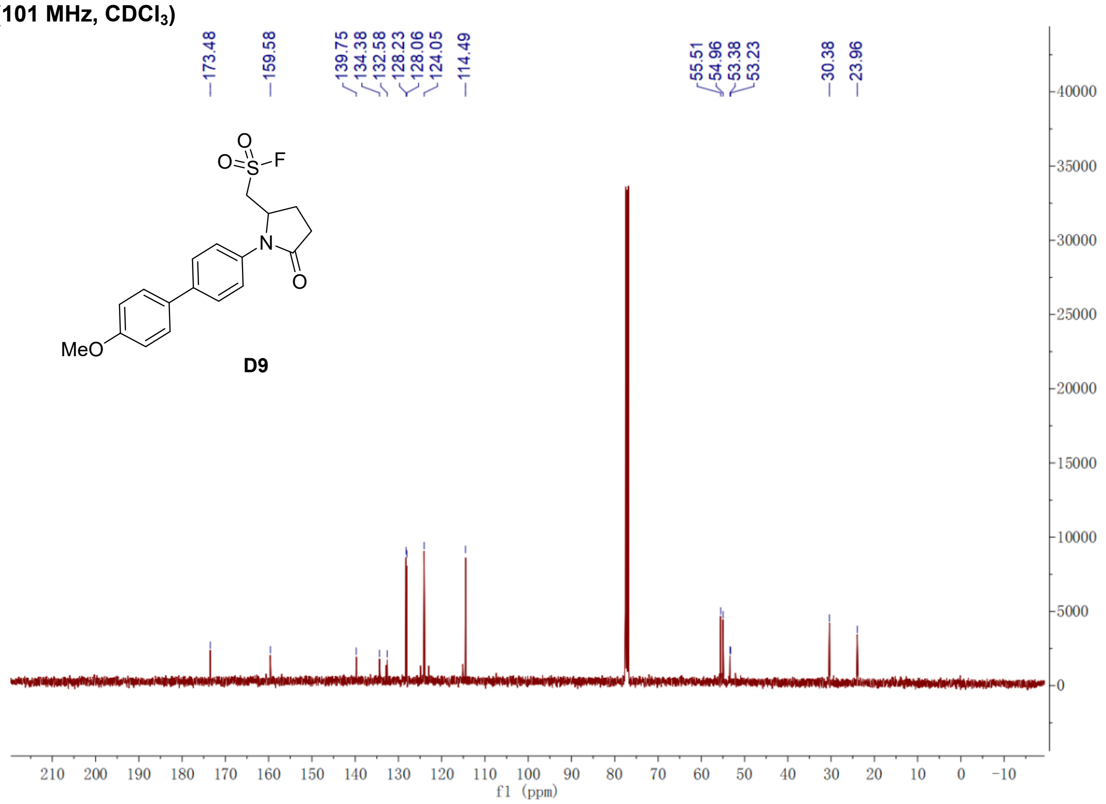

^{19}F NMR (376 MHz, CDCl$_3$)

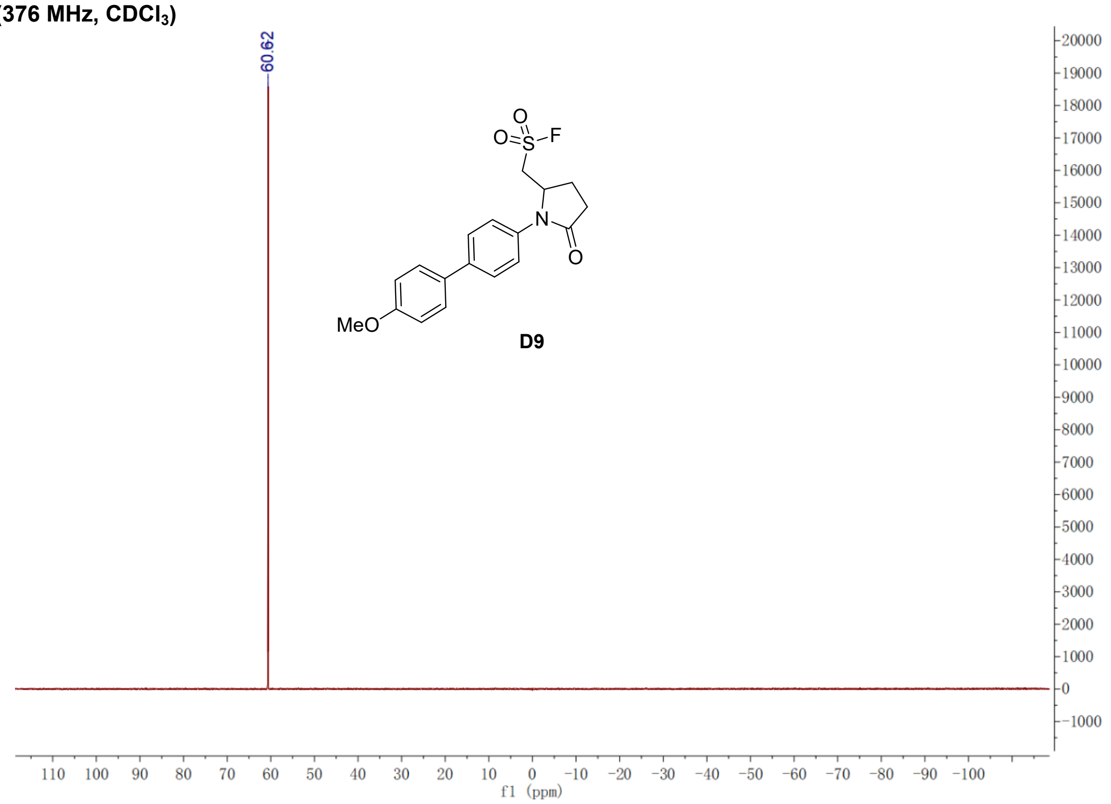

^{1}H-NMR (400 MHz, CDCl$_3$)

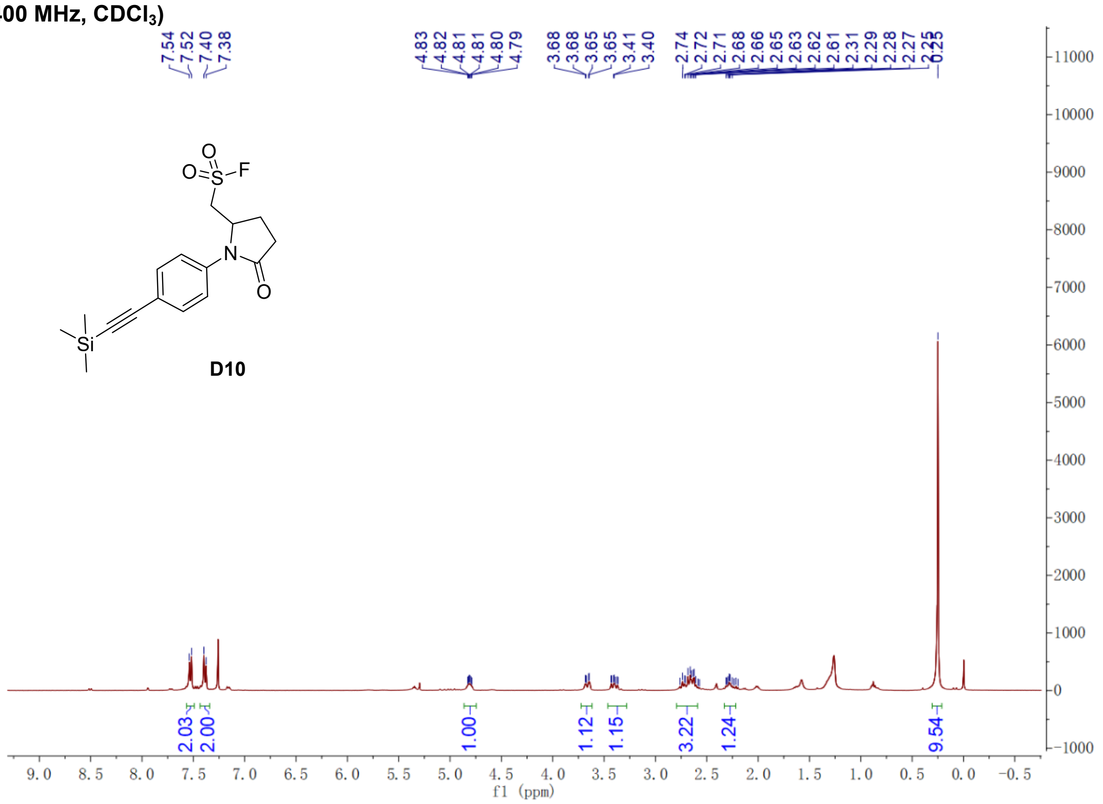

^{13}C-NMR (126 MHz, CDCl$_3$)

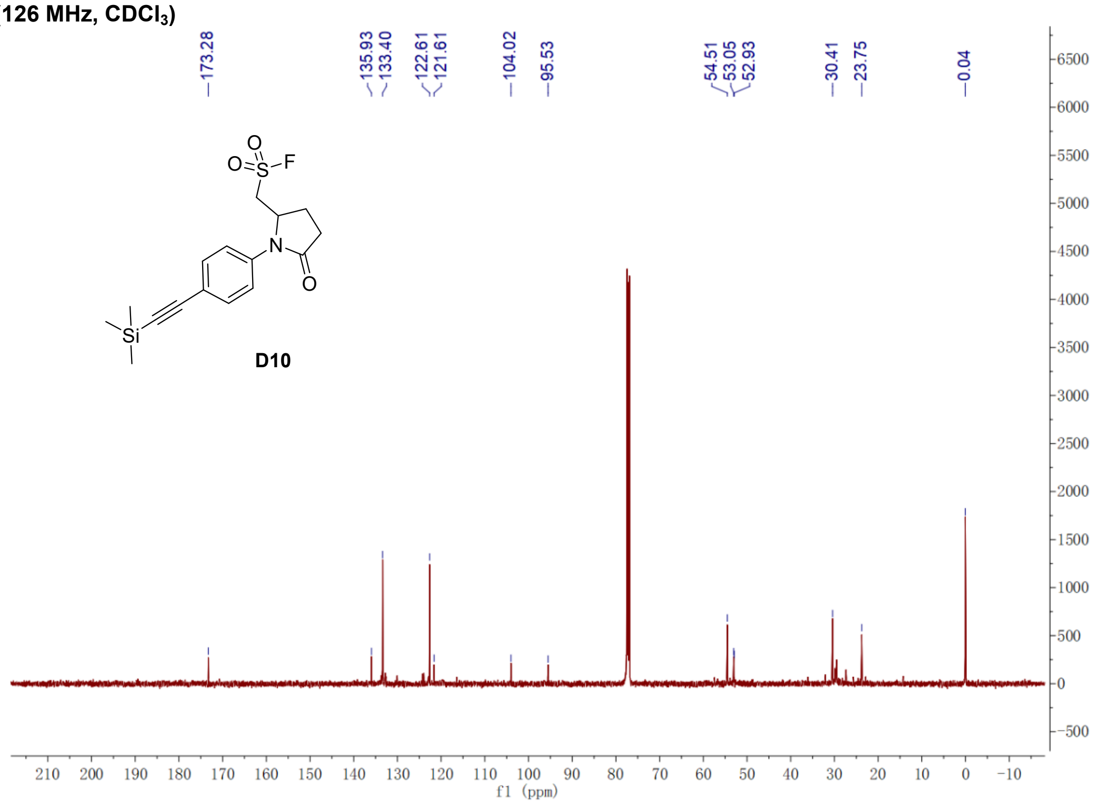

^{19}F NMR (376 MHz, CDCl$_3$)

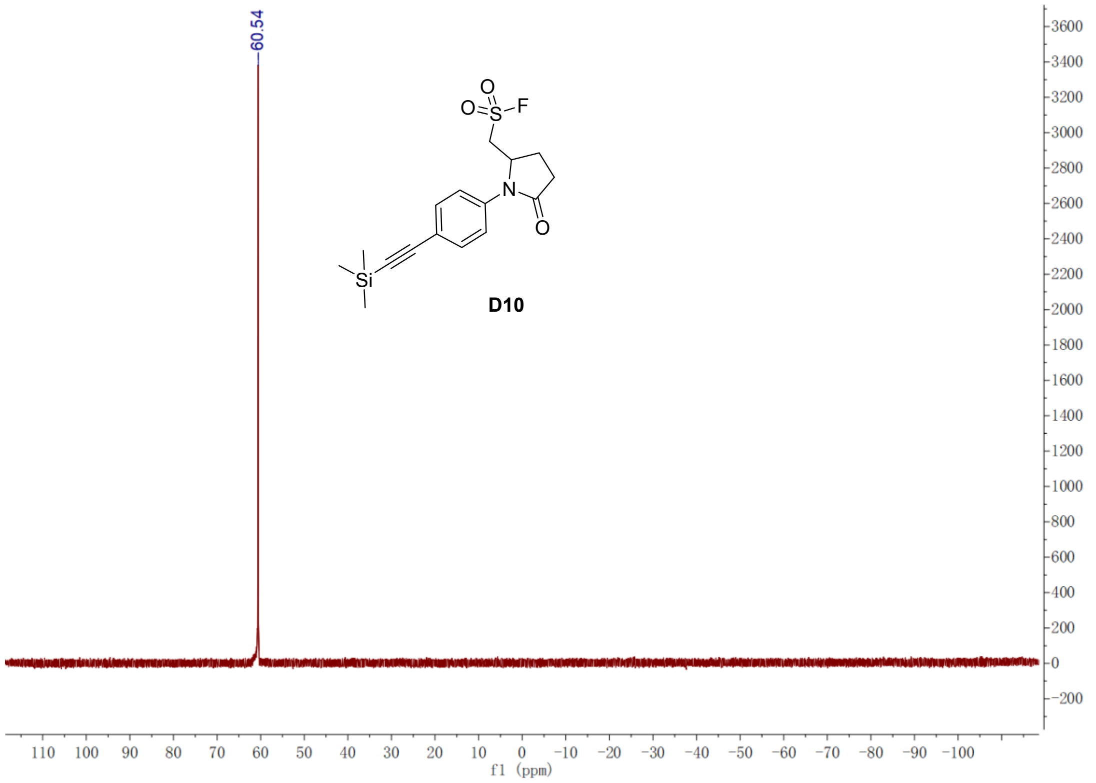

^{1}H-NMR (400 MHz, $CDCl_3$)

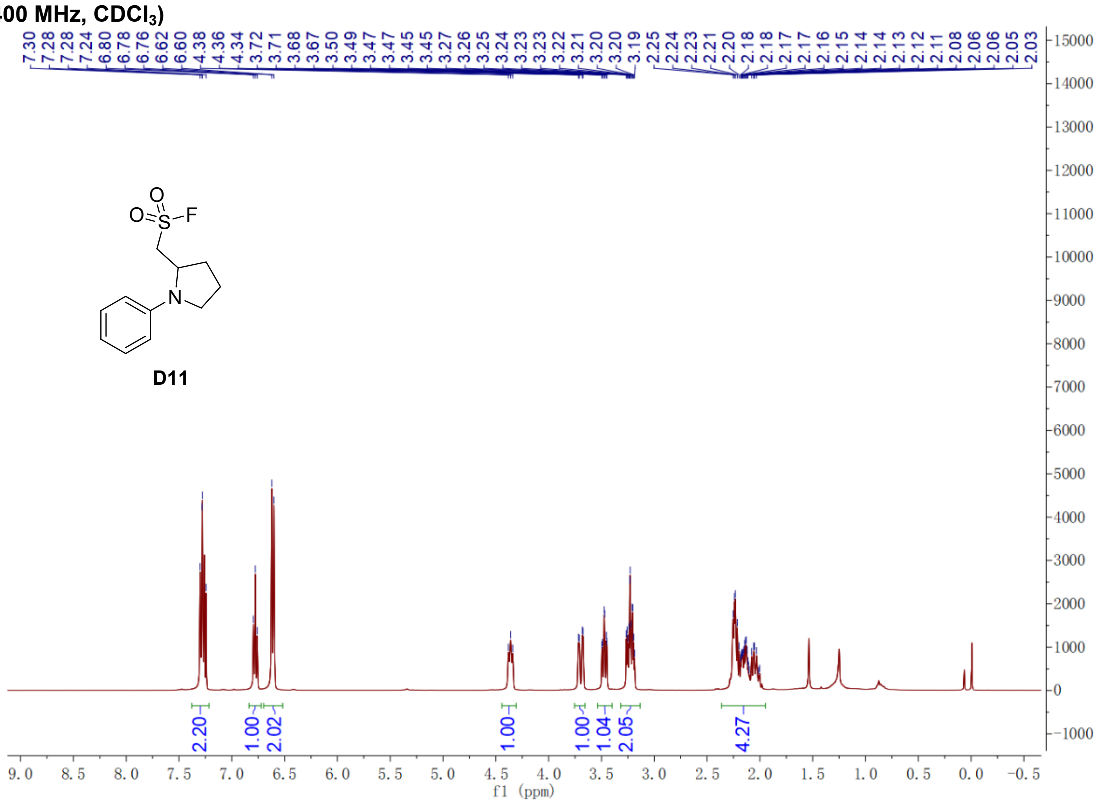

^{13}C-NMR (101 MHz, $CDCl_3$)

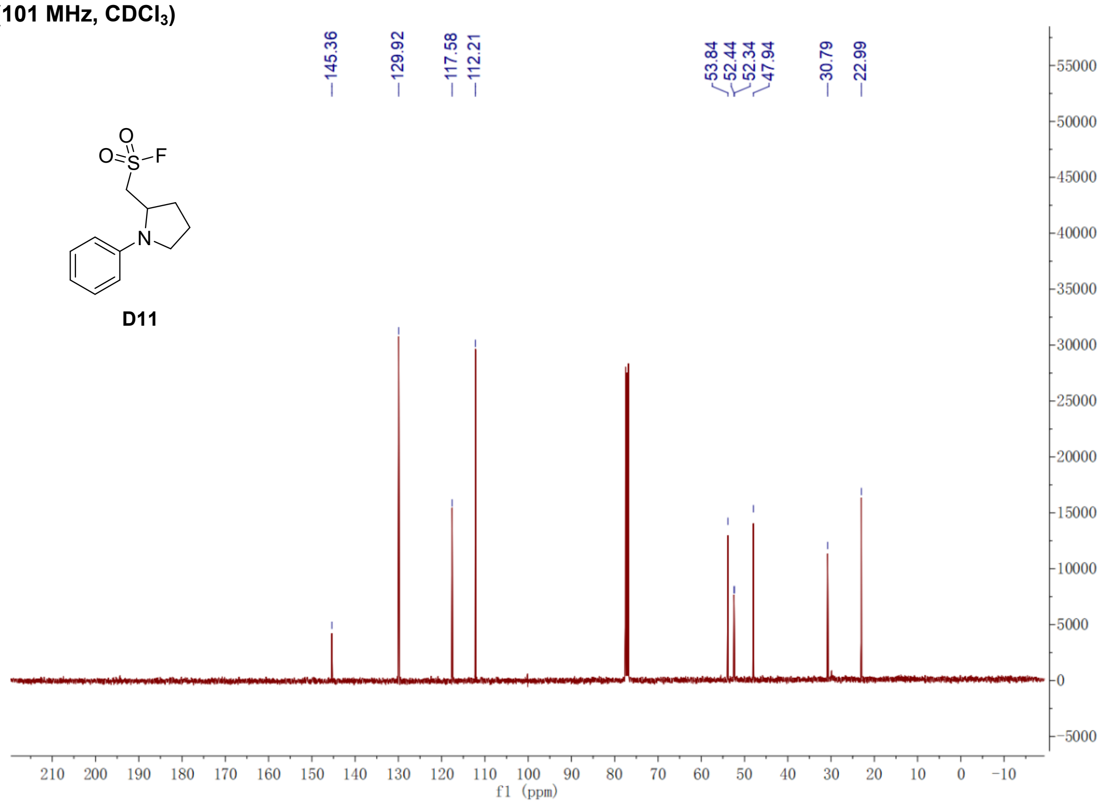

^{19}F NMR (376 MHz, $CDCl_3$)

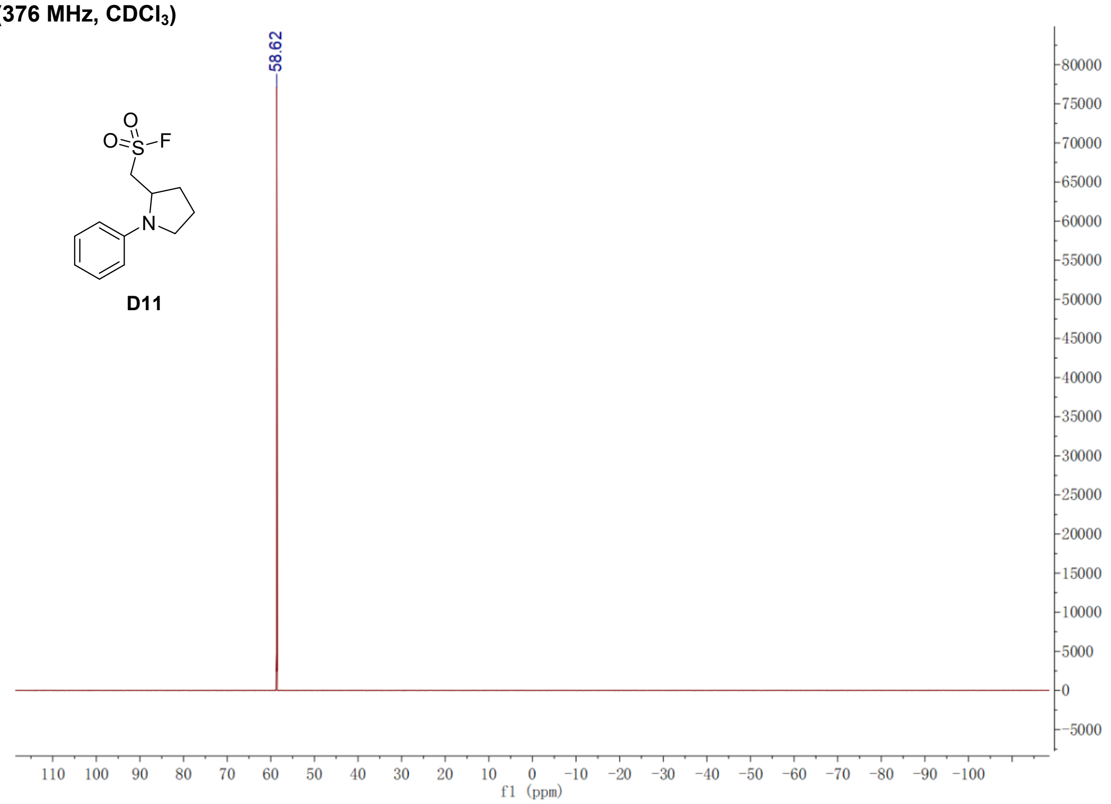

^{1}H-NMR (400 MHz, $CDCl_3$)

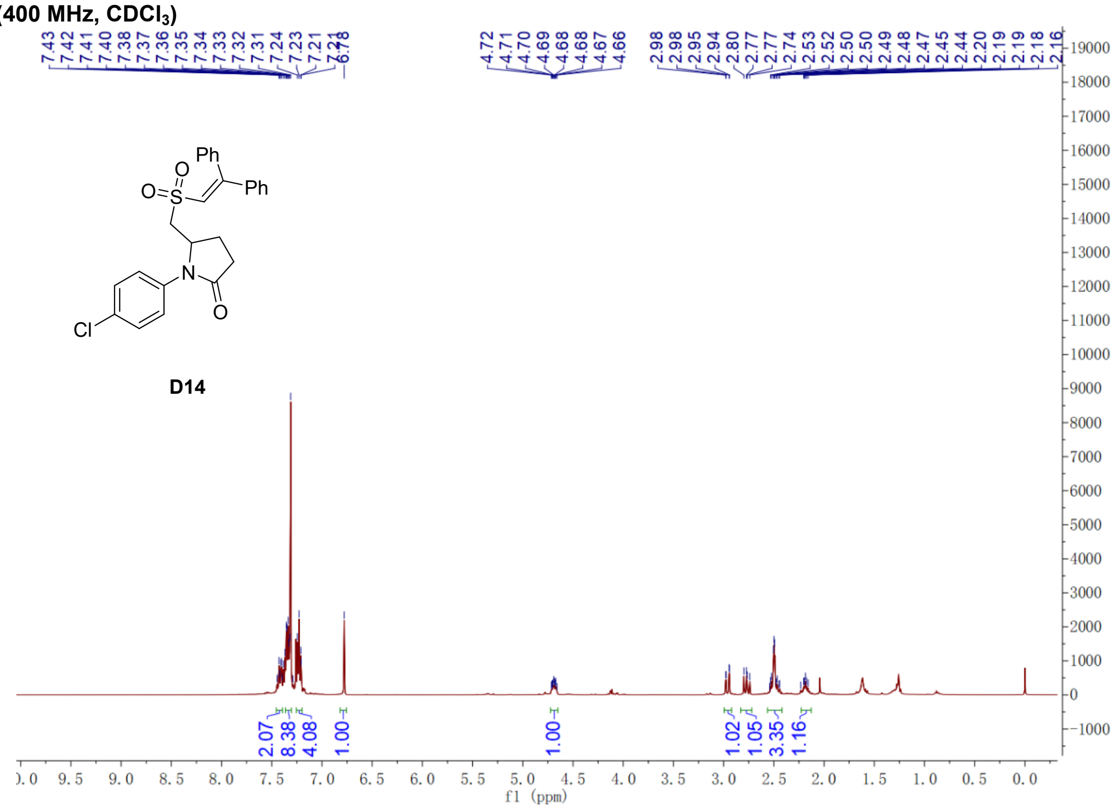

^{13}C-NMR (101 MHz, CDCl$_3$)

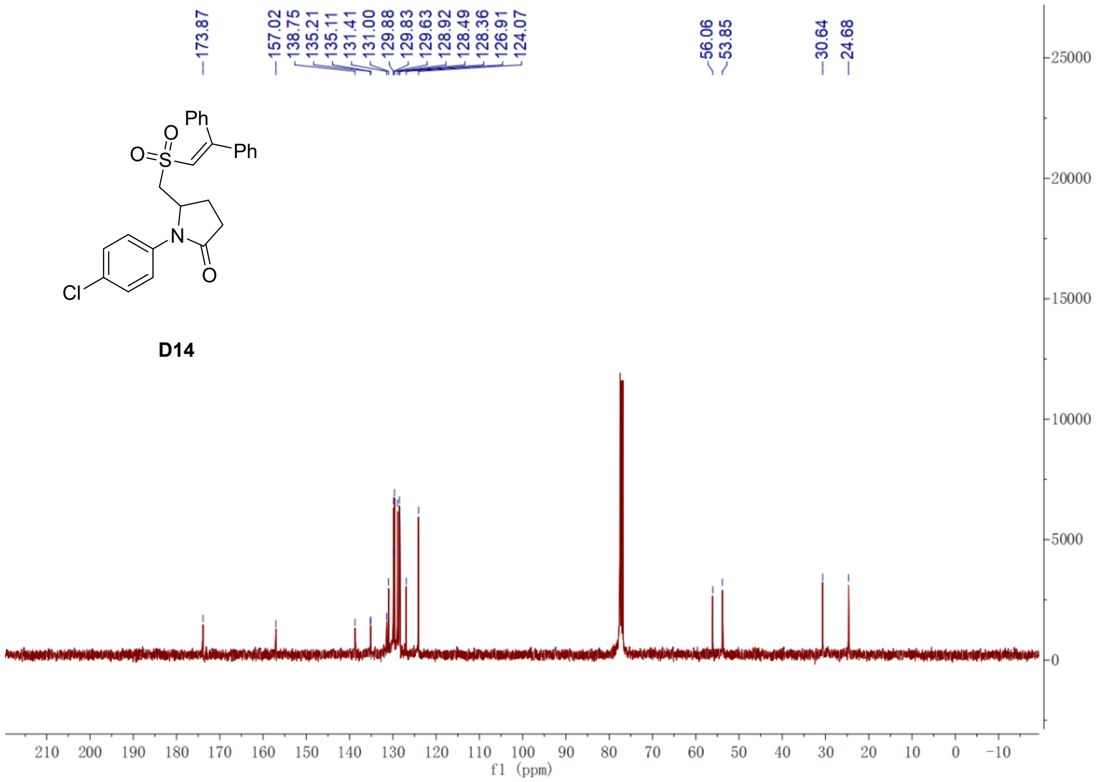

D14

^{1}H-NMR (500 MHz, CDCl$_3$)

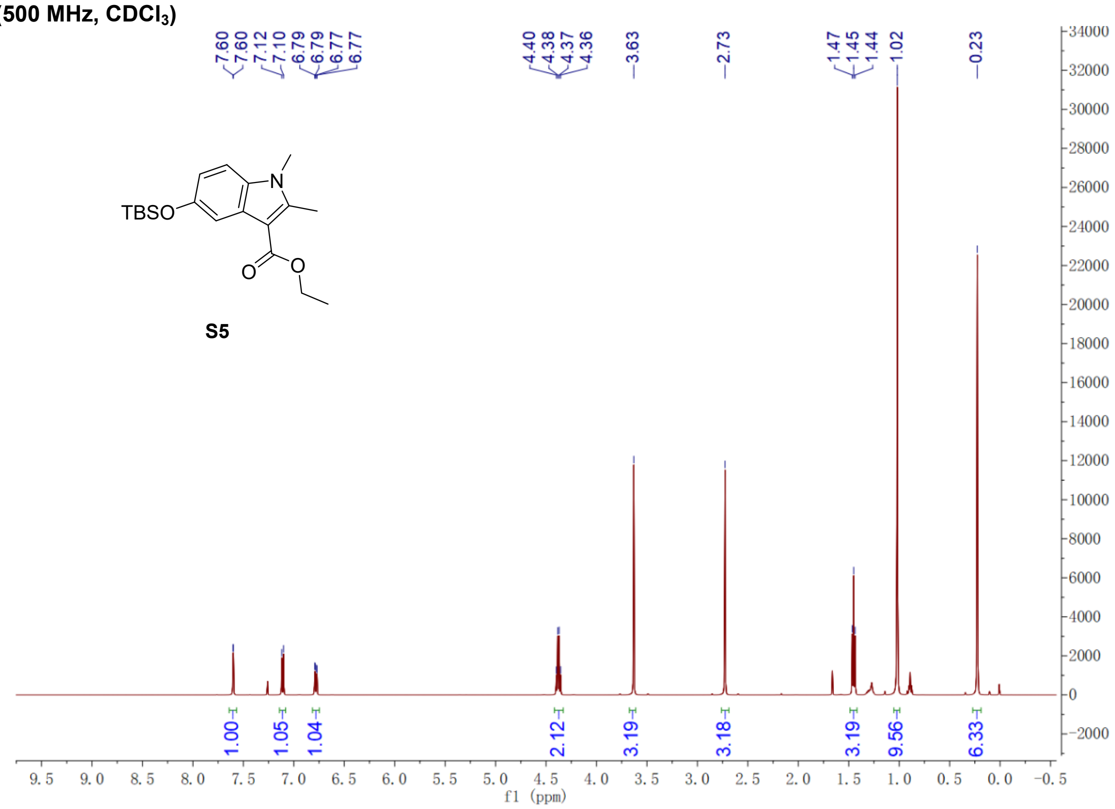

S5

^{13}C-NMR (126 MHz, $CDCl_3$)

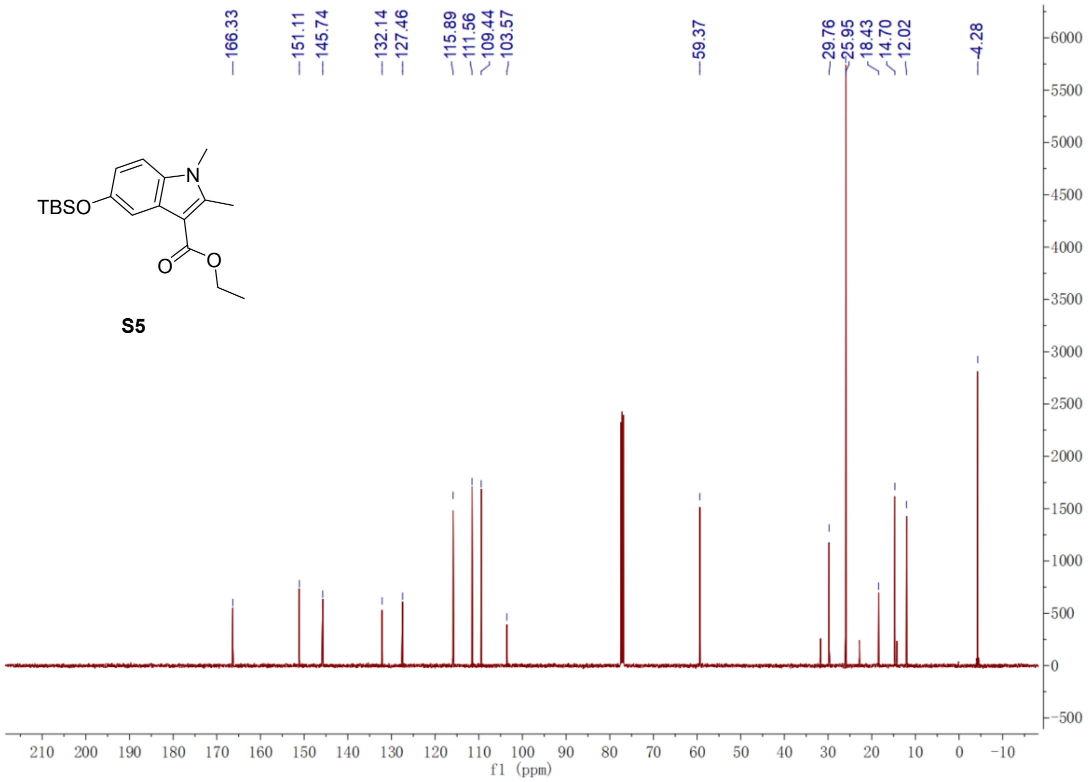

Supplement: SC-012-D1SC02503A-s001 [file SC-012-D1SC02503A-s001.pdf]
